# Oxoammonium salts are catalysing efficient and selective halogenation of olefins, alkynes and aromatics

**DOI:** 10.1038/s41467-021-24174-w

**Published:** 2021-06-23

**Authors:** Weijin Wang, Xinyao Li, Xiaoxue Yang, Lingsheng Ai, Zhiwen Gong, Ning Jiao, Song Song

**Affiliations:** 1 State Key Laboratory of Natural and Biomimetic Drugs, Peking University, Beijing, China; 2 State Key Laboratory of Organometallic Chemistry, Chinese Academy of Sciences, Shanghai, China

**Keywords:** Homogeneous catalysis, Synthetic chemistry methodology

## Abstract

Electrophilic halogenation reactions have been a reliable approach to accessing organohalides. During the past decades, various catalytic systems have been developed for the activation of haleniums. However, there is still a short of effective catalysts, which could cover various halogenation reactions and broad scope of unsaturated compounds. Herein, TEMPO (2,2,6,6-tetramethylpiperidine nitroxide) and its derivatives are disclosed as active catalysts for electrophilic halogenation of olefins, alkynes, and aromatics. These catalysts are stable, readily available, and reactive enough to activate haleniums including Br^+^, I^+^ and even Cl^+^ reagents. This catalytic system is applicable to various halogenations including haloarylation of olefins or dibromination of alkynes, which were rarely realized in previous Lewis base catalysis or Lewis acid catalysis. The high catalytic ability is attributed to a synergistic activation model of electrophilic halogenating reagents, where the carbonyl group and the halogen atom are both activated by present TEMPO catalysis.

## Abstract (web summary)

Organohalides are widely used as synthetic precursors and target products, but for various halogenation reactions there is a need for effective catalysts to activate commercially available haleniums. Here, the authors report that TEMPO and its derivatives are active catalysts for electrophilic halogenation of olefins, alkynes and aromatics, under mild reaction conditions and with good functional group tolerance.

## Introduction

**Fig. 1:**
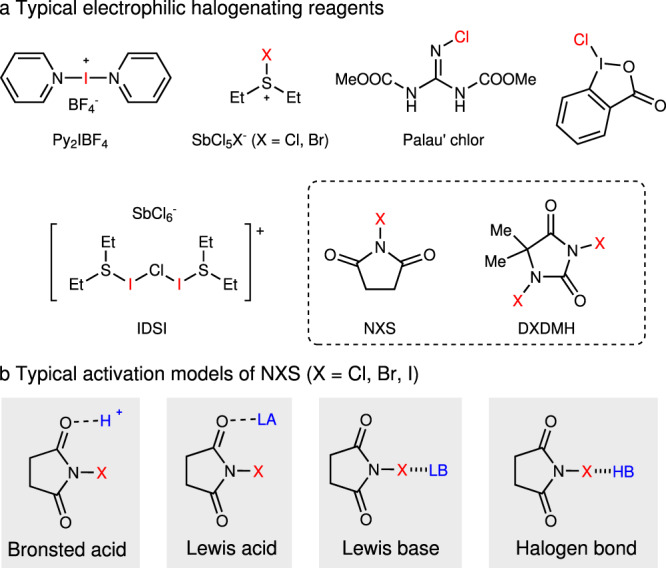
Typical electrophilic halogenating reagents and activation models. **a** Typical electrophilic halogenating reagents. **b** Typical activation models of NXS.

Organohalides are undoubtedly important chemicals, and have been widely used as the synthetic precursors as well as the target products^1–3^. The carbon–halogen bonds play as a key functional regulator in agrochemicals, materials, natural products, and pharmaceuticals^4–6^. In order to overcome the limitations of electrophilic halogenation with molecular halogen (X_2_, X = Cl, Br, I)^7^, various electrophilic halogenating reagents have been developed (Fig. 1a)^8–10^. Especially, Py_2_IBF_4_^11–14^, Et_2_SX•SbCl_5_X (X = Br or Cl)^15–18^, and Palau’chlor^19^ show high efficiency in the construction of C–X bonds. In contrast, the very common and readily available *N*-halosuccinimides (NXS, X = Cl, Br, I) and dihalo-dimethylhydantoin (DXDMH, X = Cl, Br, I) are practical, stable, and inexpensive, but generally with relative lower reactivity and selectivity (Fig. 1a)^20,21^. For example, Baran et al.^19^ tested the chlorination of anisole with *N*-chlorosuccinimide (NCS), and found that NCS could only chlorinate anisole under the addition of other activators. Therefore, in the past decades, various strategies have been developed to activate NXS or DXDMH for halogenations of unsaturated compounds^22^.

**Fig. 2:**
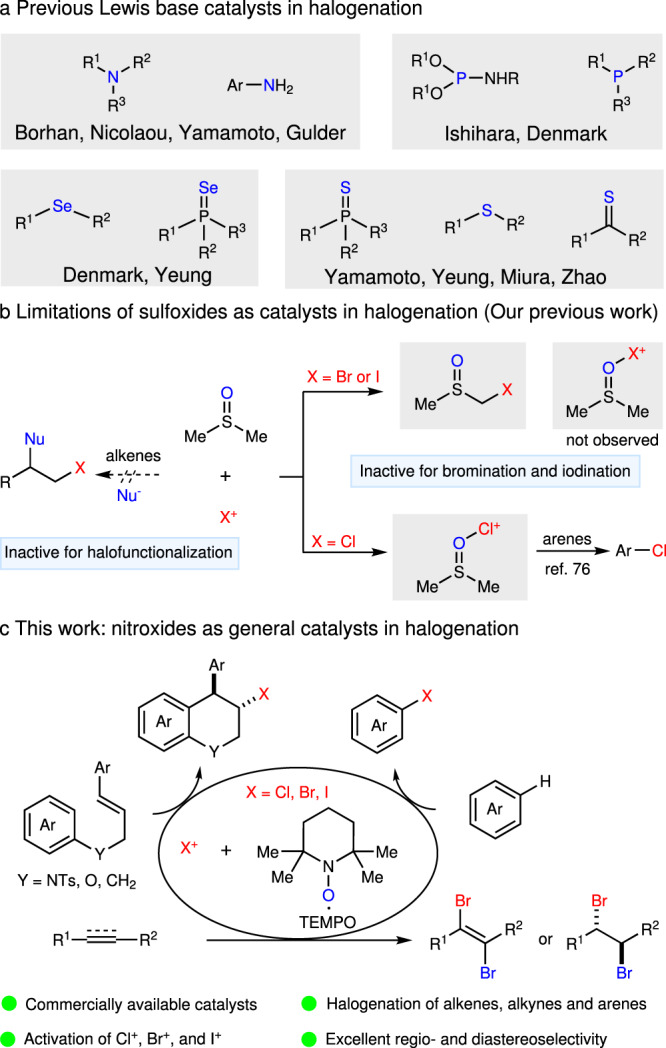
Overview of previous and current work. **a** Previous Lewis base catalysts in halogenation. **b** Limitations of DMSO as catalysts in halogenation. **c** This work: nitroxides as general catalysts in halogenation.

To the best of our knowledge, there are four typical activating strategies to enhance the reactivity of haleniums (Fig. 1b, with NXS as the example). The protonation^23–26^ of carbonyl group in NXS is a widely applied strategy. The Lewis acid^27–33^ could also coordinate with carbonyl group, thus realizing the activation of NXS. The halogen bond reagents could promote the N–X bond cleavage^34,35^, which is beneficial to the electrophilicity of haleniums. Encouraged by Denmark’s work^36–39^, Lewis bases, which always bear a donor heteroatom, have been widely employed in activation of haleniums. Up to date, *N*-centered^40–52^, *S*-centered^53–63^, *Se*-centered^64–70^, and *P*-centered^71,72^ Lewis bases showed high efficiency in halogenation reactions (Fig. 2a). For example, Borhan^44–48^ and Nicolaou^49^ employed (DHQD)_2_PHAL as an efficient catalyst in enantioselective chlorofunctionalization reactions. Yamamoto et al.^50^ reported 2,4,6-trimethylaniline catalyzed aromatic halogenation. Besides, morpholine could act as an efficient promoter in haliranium-induced polyene cyclization reported by Gulder et al.^51^. Significantly, the enantioselective halo-polyene cyclization was developed by Ishihara et al.^72^ with phosphoramidites as chiral promoters. Later on, a catalytic asymmetric approach was established by Yamamoto et al.^57^ utilizing a dual-role Lewis base/Brønsted acid catalyst. Denmark et al.^64–69^ disclosed *Se*-centered catalysts for enantioselective halo- and sulfenofunctionalization reactions. Besides, bifunctional sulfide and selenide catalysis were developed by Zhao et al. for enantioselective halogenation reactions^56^. Yeung et al. found that thioethers^58^, thiocarbamates^59–61^, and selenides^70^ were efficient Lewis base catalysts for enantioselective halofunctionaization reactions. Recently, triptycenyl sulfide showed high catalytic reactivity in electrophilic aromatic halogenation by Nishii and Miura^62^.

Despite the significance of these catalysts, obvious limitations still restricted the wide application of these methods: (1) some challenging halofunctionalization reactions such as intramolecular haloarylation of olefins cannot be achieved with these Lewis base catalysts. (2) Usually, each catalyst can only be compatible with one type of nucleophilic substrates or activate one type of haleniums. There is a short of catalytic method with both broad substrate scope and reaction scope. Compared with above Lewis bases, organic oxides^73,74^ were previously considered inactive for halofunctionalization of unsaturated compounds^75^. Recently, our group reported dimethyl sulfoxide (DMSO) as an efficient catalyst to catalyze the aromatic chlorination reactions^76^. However, the reactive methyl groups^77^ and strong nucleophilicity of DMSO results in the failure to catalyze both bromination or iodination of arenes and halofunctionalization of olefins (Fig. 2b)^78–81^. Thus, the development of effective and diverse catalysts to activate commercially available haleniums, which could cover various halogenation reactions (bromination, iodination, and even chlorination) and broad scope of unsaturated compounds (olefins, arenes, and alkynes) to overcome the existing limitations in the halogenation field, is very urgent. Our inspiration came from the formation enthalpies of the iodine complexes with Lewis bases^82^. The absolute values of enthalpies paralleled with their catalytic activities in the halofunctionalization reactions^75^. Some nitroxides showed quite high enthalpies, therefore, we speculated that nitroxides could activate electrophilic halogenating reagents.

In this work, TEMPO^+^ and its derivatives, which could be generated in situ from commercially available TEMPO^83–86^ and electrophilic halogenating reagents, were developed as diverse and efficient catalysts in various halogenation reactions. Besides bromonium and iodonium, the chloronium was also strongly activated by catalytic amount of TEMPO^+^. The strong activating ability and low nucleophilicity of TEMPO make the tolerance of olefins, alkynes, and arenes possible, thus overcoming the inherent limitations of previous sulfoxide catalysis^76^. Based on the high reactivity, the intramolecular haloarylation of olefins, dibromination of olefins, and alkynes, as well as aromatic halogenation of arenes were realized with good selectivity and efficiency (Fig. 2c).

## Results and discussion

### TEMPO-catalyzed intramolecular haloarylation of olefins

**Fig. 3:**
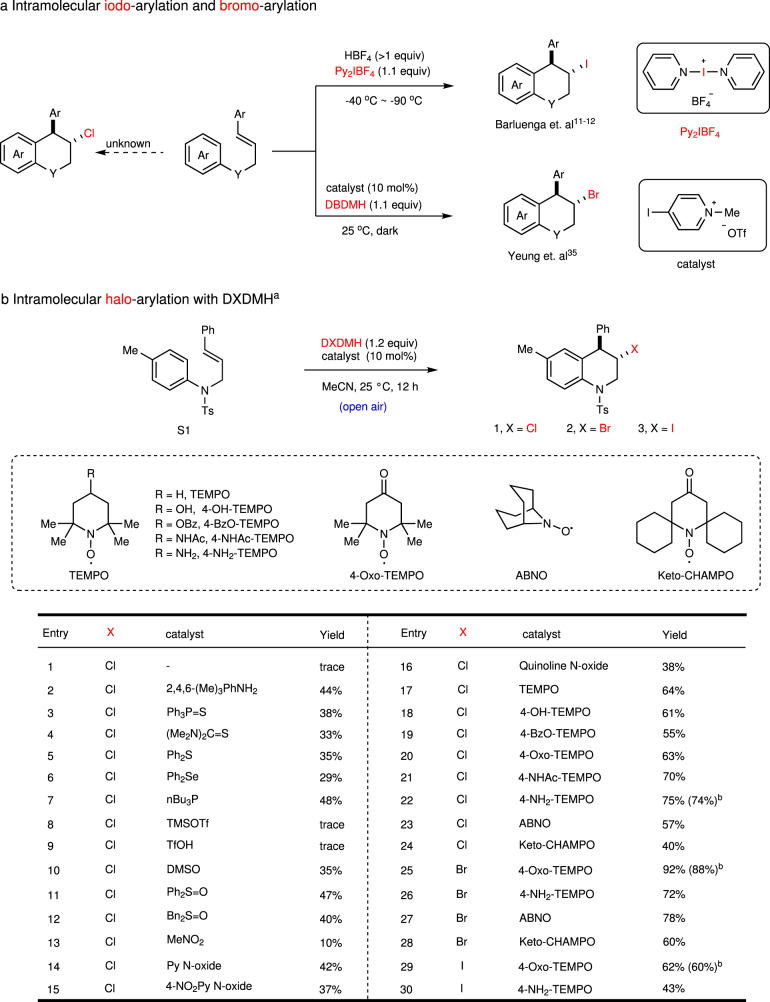
Optimization of reaction conditions. **a** Previous reports on intramolecular haloarylation reactions. **b** Optimization of reaction conditions. ^a^Reactions were carried out with **S1** (0.10 mmol), catalyst (0.01 mmol) and DXDMH (0.12 mmol) in MeCN (1.0 ml) for 12 h at 25 °C. Yields were determined by ^1^H-NMR using 1,1,2,2-tetrachloroethane as the internal standard. ^b^Isolated yields of **1**–**3** for the reaction at 0.20 mmol scale. TsOH *p*-toluenesulfonic acid, TMSOTf trimethylsilyl trifluoromethylsulfonate.

The intramolecular haloarylation of olefins provides a convenient protocol to valuable synthetic intermediates^87,88^. The iodo- and bromoarylation of olefins have been well realized by Barluenga^11,12^ and Yeung^35^. However, the chloroarylation was more challenging and rarely realized based on two reasons: (1) the unstability of chloriranium ion makes it more likely to form a free carbocation, resulting in low stereoselectivity or decomposition; (2) the chlorenium ion is more reactive so that it tends to react with nucleophiles before forming a chloriranium ion (Fig. 3a). Therefore, developing a versatile synthetic system for all chloro-, bromo- and iodo-arylation under mild reaction conditions is still in great need. Thus, the chloroarylation of **S1** was initially investigated in MeCN at 25 ^°^C with DCDMH as the chloro-source (Fig. 3b). In the absence of catalyst, DCDMH was not active enough to promote the cyclization, and only trace amount of **1** was detected (Entry 1). The classical Lewis base catalysts, including 2,4,6-trimethylaniline, Ph_3_P = S, thiourea, Ph_2_S, Ph_2_Se, *n*Bu_3_P showed low reactivity (Entries 2–7). Lewis acid TMSOTf or Brønsted acid TsOH could not catalyze the transformation from **S1** to **1** (Entries 8–9). The sulfoxides including DMSO, Ph_2_S = O, and Bn_2_S = O could not catalyze this reaction efficiently (Entries 10–12). Then, a series of reagents bearing N–O bond(s) were tested (Entries 13–16). Nitromethane, which promoted the fluorination efficiently^89^, failed to catalyze this reaction (Entry 13). The *N*-oxides of pyridine, 4-nitropyridine, and quinoline showed similar catalytic activity with sulfoxides (Entries 14–16). To our delight, the yield of **1** increased to 64% with 10 mol% of TEMPO as the catalyst (Entry 17). 4-OH, 4-BzO, or 4-Oxo substituted TEMPO gave similar yields with TEMPO (Entries 18–20), and 4-NH_2_-TEMPO gave the highest 75% yield (Entries 21–22). TEMPO analogs bearing different α-substituents^90^ still promoted the chloroarylation, although with lower yields (Entries 23–24). The TEMPO catalysis was also successfully applied in the bromo- and iodo-arylation of **S1**, and 4-Oxo-TEMPO showed highest catalytic reactivity (88 and 62% yields, Entries 25–30).

**Fig. 4:**
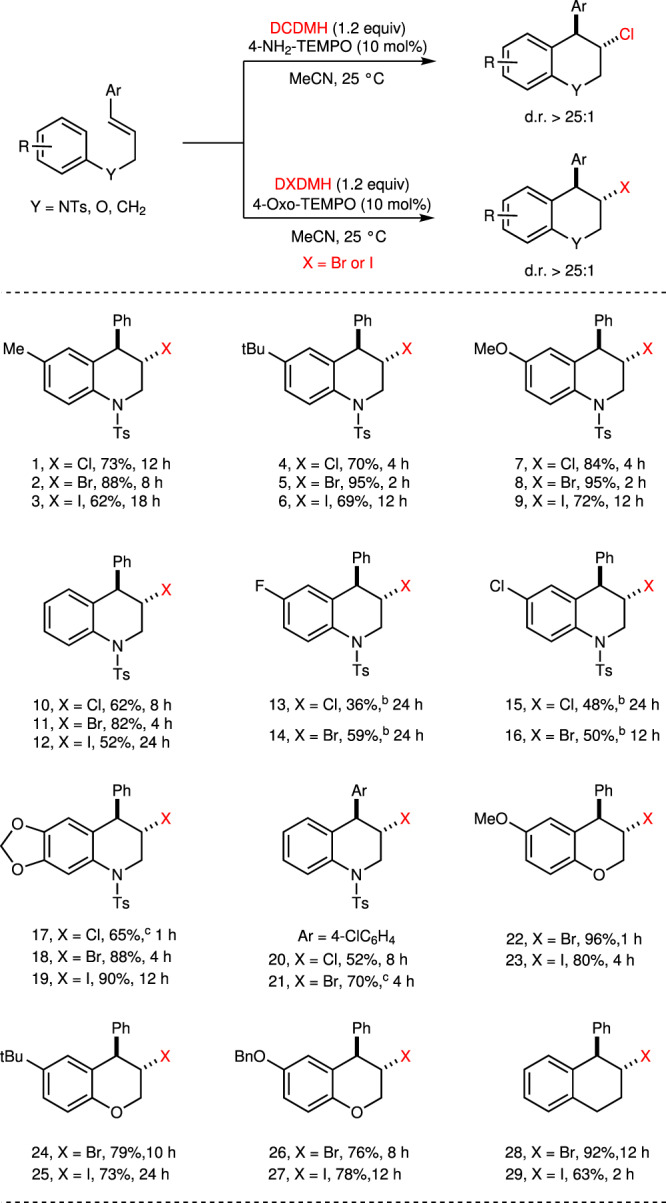
TEMPO-catalyzed haloarylation^a^. ^a^Reactions were carried out with substrate (0.20 mmol), catalyst (0.02 mmol), DXDMH (0.24 mmol), MeCN (2.0 mL) at 25 °C unless specified. Isolated yield. ^b^Reactions were carried out with substrate (0.20 mmol), catalyst (0.04 mmol), DXDMH (0.30 mmol), MeNO_2_ (3.0 ml) at 60 °C under air. ^c^5 mol% of catalyst was used.

With 10 mol% of TEMPO derivatives as the catalysts, the substrate scope of this intramolecular haloarylation was examined (Fig. 4). The present catalytic system exhibited high diastereoseletivity and regioselectivity, and no aromatic halogenated products were detected. Generally, the yields of bromoarylation were higher than that of chloro- and iodo-arylation. The haloarylation of *N*-cinnamyl sulfonamides proceeded smoothly to give the desired tetrahydroquinolines **1–21** in good efficiencies. Electron-donating group substituted substrates are favorable for this transformation and could be smoothly converted into cyclic products in excellent yields. Electron-withdrawing groups such as fluorine and chlorine atoms could also be tolerated in the reaction system under specified conditions, although with moderate yields due to incomplete conversion of the substrates. The bromo- and iodo-arylation of *O*-cinnamyl ethers underwent smoothly to afford chromane building blocks **22–27** in high yields. Exposure of (*E*)-but-1-ene-1,4-diyldibenzene under standard conditions delivered tetrahydronaphthalene **28–29** in good yields.

### TEMPO-catalyzed dibromination of alkenes and alkynes

**Fig. 5:**
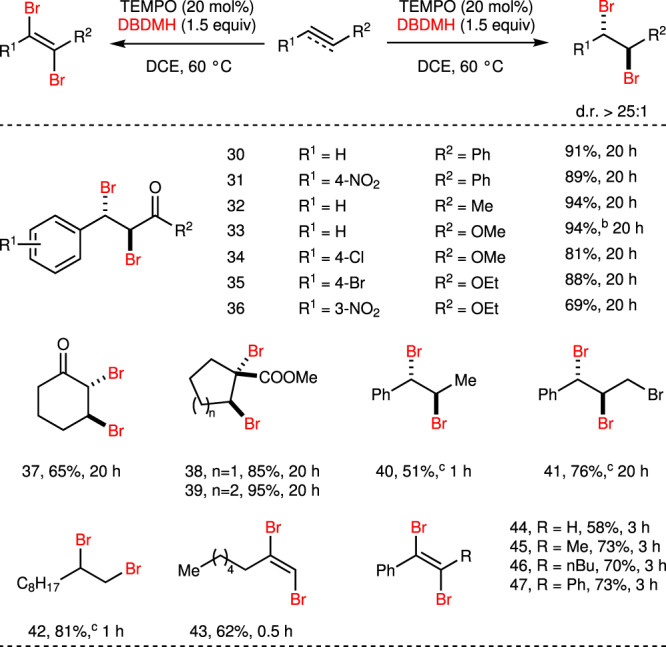
TEMPO-catalyzed dibromination of alkenes and alkynes^a^. ^a^Reactions were carried out with alkene or alkyne (0.50 mmol), TEMPO (0.10 mmol), DBDMH (0.75 mmol), DCE (2.0 ml) at 60 °C under air. Isolated yield. ^b^The catalyst was added twice in total 20 mol%. ^c^Performed at 25 °C.

Meanwhile, we noticed that X^–^ was generated in situ when TEMPO was oxidized by DXDMH. If the Br^–^ rather than arenes acted as the nucleophile (the excess bromide anions might come from in situ hydrolysis and disproportionation, see Supplementary Figs. 10–12), the dibromination of olefins would be realized. In this case, TEMPO not only functioned as an activator, but also a redox catalyst to generate X^–^. With this hypothesis, the dibromination of α,β-unsaturated carbonyl compounds^91–93^, which always afforded products in low diastereoselectivity with molecular Br_2_, was tested with TEMPO as the catalyst (Fig. 5). Chalcone and its derivatives were dibrominated with high yields (**30–31**), and it is noteworthy that the diastereoselectivity of this transformation is very high (>25:1). The dibromination of cinnamates also underwent smoothly to deliver **33–36** with good efficiency. Cyclic olefins were dibrominated to afford anti-products **37–39** bearing tertiary and quaternary C–Br bond in good yields. Besides electron-deficient olefins, the dibromination of styrene derivatives and aliphatic olefins also worked very well (**40–42**) with excellent diastereoselectivity.

Inspired by the above success, the dibromination of alkynes was tested with present TEMPO/DBDMH system to realize the efficient synthesis of (*E*)-dibromoolefins. Aliphatic or aromatic alkynes, internal or terminal alkynes were all dibrominated to afford (*E*)-dibromoolefins in good yields (**43–47**).

### TEMPO-catalyzed halogenation of (hetero)arenes

**Fig. 6:**
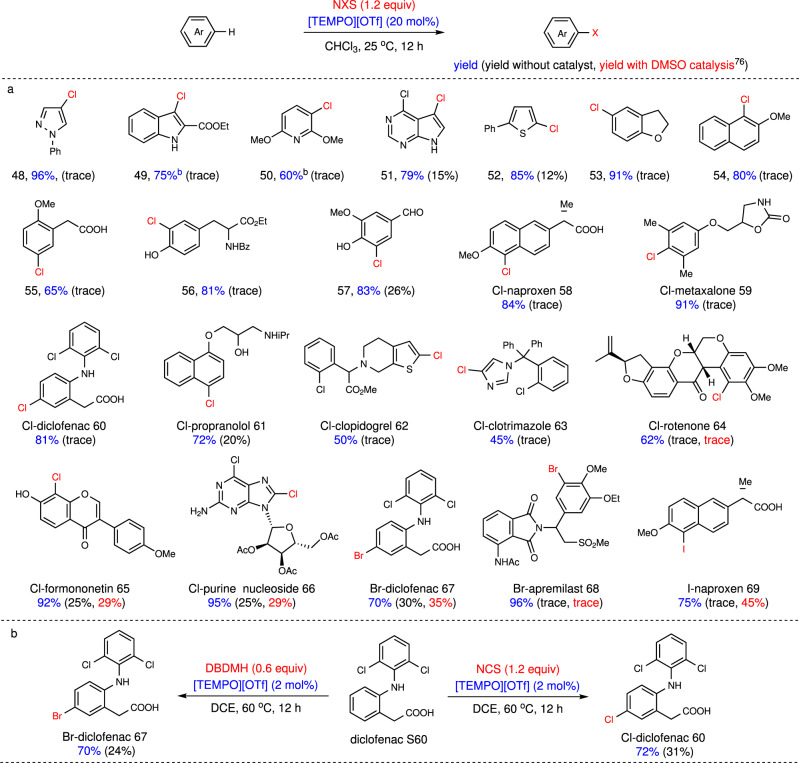
TEMPO-catalyzed halogenation of (hetero)arenes^a^. ^a^Reactions were carried out with arene (0.50 mmol), NXS (0.60–0.75 mmol), and [TEMPO][OTf] (0.10 mmol) in CHCl_3_ (2.0 ml) at 25 °C under air for 12 h. Isolated yield. The black number in parentheses was the yield without catalyst, and the red number in parentheses was the yield with 20 mol% of DMSO as the catalyst. ^b^Performed with NCS (1.0 mmol).

Electrophilic aromatic halogenation is perhaps the most direct approach to afford aryl halides, which is very important in pharmaceutical sciences^6^. Compared with bromination and iodination, the aromatic chlorination of arenes is relatively more challenging. Interestingly, mechanistic studies revealed that TEMPO was in situ oxidized by haleniums to TEMPO^+^, which was the real catalyst to promote haloarylation reactions (see below). Encouraged by the above results, the chlorination of many (hetero)arenes, which could not be chlorinated effectively without a catalyst (yields < 26%, see Supplementary Information), was investigated by [TEMPO][OTf] catalysis with NCS (Fig. 6a). Various aromatics including benzene, indole, pyrazole, pyridine, and thiophene were chlorinated in high yields and excellent regioselectivity at room temperature (**48–57**). The ester, amide, hydroxyl, carboxylic acid, aldehyde groups were all tolerated in the present catalysis.

The mild reaction conditions and excellent functional group tolerance enabled this protocol to be applied in the late-stage halogenation of drugs and natural products (Fig. 6a). Many drugs containing various functional groups which restrict their chlorination, underwent the chlorination smoothly under the present [TEMPO][OTf] catalysis to afford the corresponding products in good yields and selectivities (**58–69**). The chlorination of propranolol, diclofenac, or metaxalone with NCS only afforded trace amount of product, maybe because the amines or hydroxyl groups restricted the chlorination. Gratifyingly, the above drugs were chlorinated in good yields and selectivities in the presence of catalytic [TEMPO][OTf] (**59–61**). Clopidogrel bearing benzene and thiophene cycles was selectively chlorinated at the thiophene position (**62**). The *N*-heterocycle of clotrimazole was chlorinated under the standard conditions (**63**). TEMPO^+^ catalysis showed better functional group tolerance than that of DMSO. The chlorination of rotenone, formononetin, and purine analog did not work in previous DMSO catalysis^76^, but were significantly promoted by TEMPO^+^ with the survival of olefin, ketone, and sugar motif (**64–66**). The TEMPO^+^ catalysis was also successfully applied in the late-stage bromination and iodination of bioactive molecules (**67–69**), which showed very low efficiency in previous DMSO catalysis^76^. It is noteworthy that the present TEMPO^+^ catalysis showed higher efficiency than that of other catalysts for the chlorination of diclofenac (see Supplementary Table 5) and the catalyst loading can be reduced to 2 mol% under 60 °C (Fig. 6b).

### Mechanistic studies

**Fig. 7:**
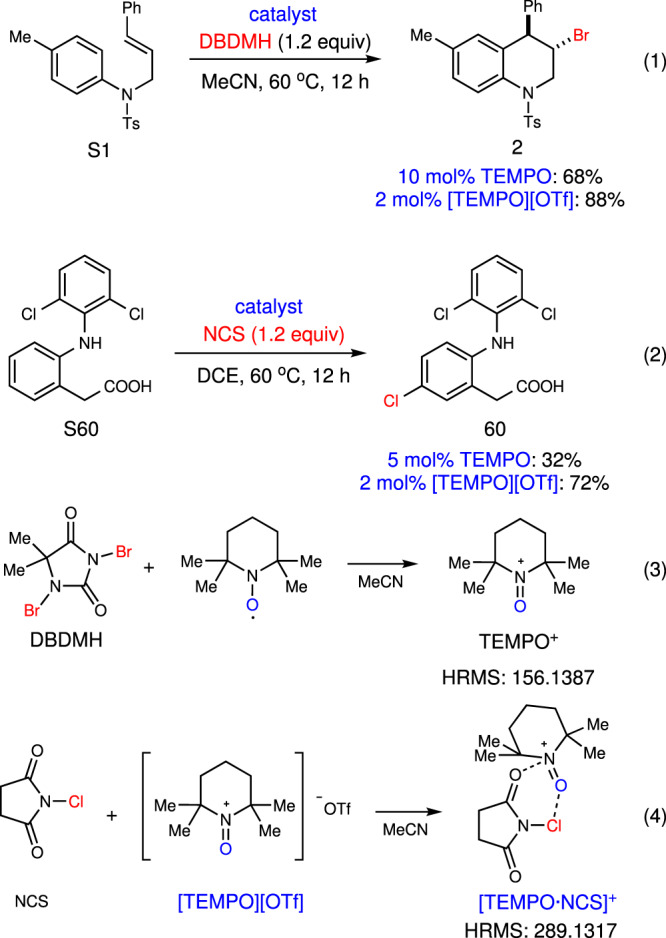
Control experiments. The results in Eqs. (1) and (2) indicate that TEMPO^+^ is the real catalyst to activate haleniums. The formation of TEMPO^+^ and [TEMPO·NCS]^+^ was detected by HRMS as shown in Eqs. (3) and (4).

To investigate the mechanism of TEMPO-catalyzed halogenation, several mechanistic experiments were conducted. The haloarylation underwent smoothly in the presence of 2.0 equiv of BHT (see Supplementary Information), which indicated radical process may be not involved in the system. In order to illustrate the relative activity of TEMPO and [TEMPO][OTf], we further performed the bromoarylation reaction and aromatic halogenation reaction with various loadings of the catalyst. The bromoarylation of **S1** was promoted moderately by 10 mol% of TEMPO to afford 68% yield, and the yield could be significantly increased by only 2 mol% of [TEMPO][OTf] catalyst (Fig. 7, Eq. (1)). The same rule was exhibited in the aromatic chlorination of diclofenac **S60** (Fig. 7, Eq. (2)). These experiments indicate that TEMPO^+^ is the real catalyst to activate haleniums and we attributed the lower yields obtained by TEMPO catalysis to the different counterions formed in the system (Supplementary Tables 11 and 12). Besides, the reaction of TEMPO and DBDMH in MeCN was monitored (Fig. 7, Eq. (3)), and the formation of TEMPO^+^ was confirmed by HRMS-ESI analysis, which is identical with previous reports^94,95^. When NCS and [TEMPO][OTf] was mixed in MeCN, the signal of active intermediate [TEMPO·NCS]^+^ was detected by HRMS (Fig. 7, Eq. (4)).

**Fig. 8:**
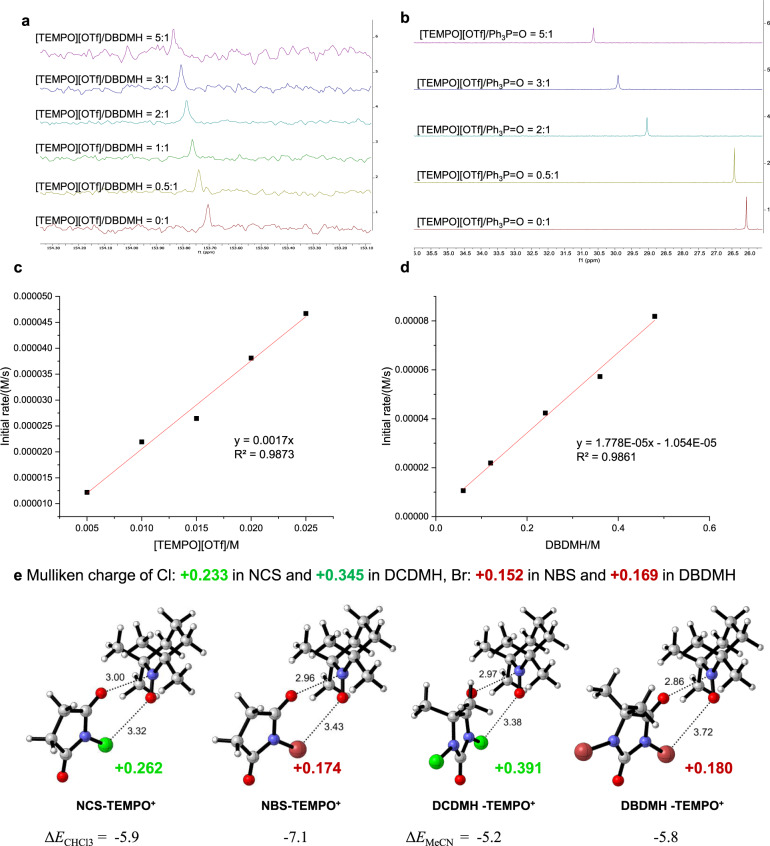
Mechanistic studies. **a** ^13^C-NMR titration experiment between DBDMH and [TEMPO][OTf]. **b** ^31^P-NMR titration experiment between Ph_3_P = O and [TEMPO][OTf]. **c** Kinetic study of [TEMPO][OTf]. **d** Kinetic study of DBDMH. **e** Plausible intermediate from halogenating reagents and TEMPO^+^. Δ*E* is energy change associated with halogenating reagent TEMPO^+^ complex formation (Δ*E* unit: kcal/mol).

To further illustrate the roles of TEMPO^+^, NMR titration studies were conducted. The ^13^C-NMR chemical shift of *C*-2 in DBDMH shifted to a lower field with the increasing amount of TEMPO^+^ (Fig. 8a), indicating that the electron density of *C*-2 was decreased in the presence of TEMPO^+^. This result was similar with that of typical Lewis acid In(OTf)_3_ (see Supplementary Fig. 2), suggesting that the positive nitrogen in TEMPO^+^ functioned as a potential Lewis acid. Furthermore, inspired by previous reports on phosphoxides as a common probe to measure Lewis acidity^96–98^, the result of ^31^P-NMR chemical shift of Ph_3_P = O shifted to a lower field with increasing amount of TEMPO^+^ confirmed the Lewis acidic property of TEMPO^+^ (Fig. 8b). Besides, the initial reaction rate was accelerated with the increment of [TEMPO][OTf] or DBDMH, and therefore first-order dependences of [TEMPO][OTf] and DBDMH were both established (Fig. 8c, d), which suggested that one [TEMPO][OTf] molecule was involved in the activation of DBDMH.

To explain the activation of the TEMPO^+^-catalyzed halogenation, DFT calculations^99^ were carried out. The halogenating reagents interacts with TEMPO^+^ toward two directions as the methyl groups stand in axial or equatorial bond, in which the equatorial bond side has more space for the interaction with lower stabilization energy than the axial bond one (Fig. 8e). The calculation result indicates that NXS or DXDMH forms a complex with TEMPO^+^ toward the equatorial bond direction and the stabilization energy is 7.6–9.5 kcal/mol. The N-atom of TEMPO^+^ interacts with O atom of the halogenating reagents (N–O distance: 2.75–2.79 Å), while O-atom of TEMPO^+^ interacts weekly with X atom of the halogenating reagents (O–X distance: 3.15–3.36 Å). The latter interaction also causes slight stabilization. The atomic charges were calculated using Mulliken population analysis. The calculated atomic charges show that the positive charge on the Cl atom of the halogenating reagent TEMPO^+^ complex is larger than that of isolated halogenating reagents, while the positive charge on the Br atom changes slightly. These results suggest that TEMPO^+^ could form a synergistic activation model of electrophilic halogenating reagents, where the carbonyl group and the halogen atom were both activated by TEMPO^+^ catalysis. TEMPO^+^ has short N–O distance with halogenating reagent, positive charge on the Cl atom, and low stabilization energy, which performs as a robust catalyst.

**Fig. 9:**
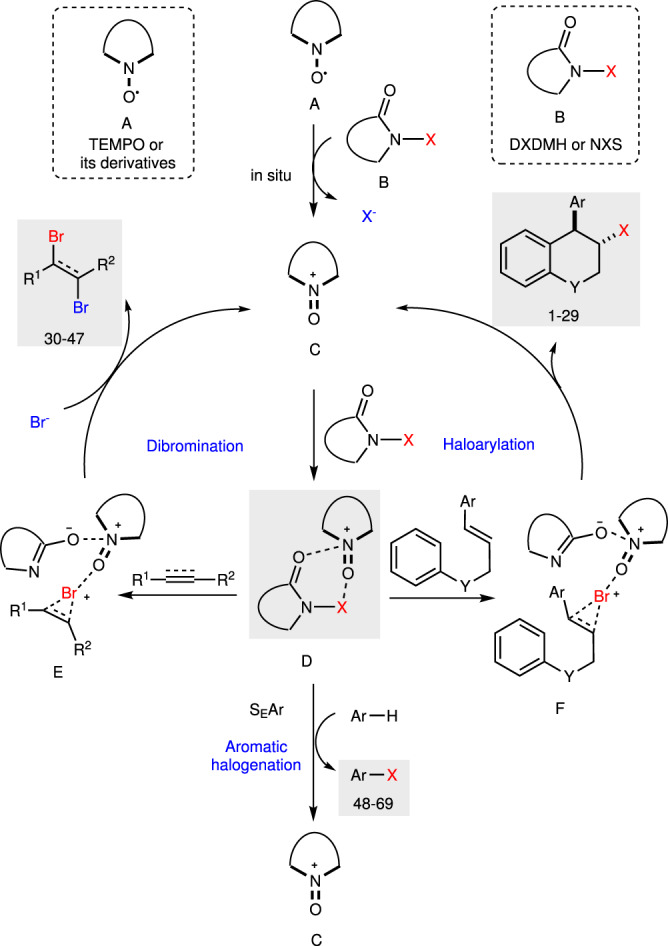
Proposed mechanism. The electropositive ammonium of N = O bond could interact with the carbonyl group of halenium reagents to decrease their electron density, and enhance their electrophilicity. Meantime, the oxygen atom on TEMPO^+^ could function like a Lewis base to form a polarized complex with halogenating reagents, which synergistically activates the haleniums.

Based on the above results and previous reports^76,86^, a plausible mechanism of present TEMPO catalysis is proposed (Fig. 9). The reaction is initiated with in situ oxidation of TEMPO **A** to TEMPO^+^ **C** by electrophilic halogenating reagents **B**, with the generation of X^–^. Then **C** interacts with halenium **B** to afford activated halogenating species **D**, which was confirmed by HRMS analysis. Two important features of TEMPO^+^ contribute to the enhanced reactivity of intermediate **D**. First, the electropositive ammonium of N = O bond could interact with the carbonyl group of halenium reagents to decrease their electron density, and enhance their electrophilicity. Meantime, we speculated the oxygen atom on TEMPO^+^ could function like a Lewis base to form a polarized complex with halogenating reagents, which synergistically activates the haleniums. Therefore, the highly reactive intermediate **D** could interact with alkenes or alkynes for the subsequent formation of halenium intermediate **E** or **F**, which is easily attacked by arene or bromine ion to deliver the corresponding products, with the regeneration of TEMPO^+^ **C**. The aromatic halogenation would deliver aryl halides via S_E_Ar process when intermediate **D** is treated with arenes.

In summary, readily available TEMPO and its derivatives have been developed as efficient catalysts to catalyze halogenation reactions. The high catalytic activity and good functional group tolerance of TEMPO^+^ made it compatible with the halogenation of arenes, olefins, and alkynes, including the haloarylation of olefins and dibromination of alkynes, which were rarely achieved by other methods. This chemistry would provide reliable catalysis in the synthesis of organohalides and promote the design and development of new catalysis in organic synthesis.

## Methods

### General procedure for TEMPO-catalyzed haloarylation

Substrate **S1–S12** (0.20 mmol), DXDMH (0.24 mmol), the catalyst (0.02 mmol), and MeCN (2.0 ml) were added to a reaction tube with a magnetic bar. The mixture was stirred at 25 °C for the specified reaction time. Upon completion of the reaction (monitored by TLC), the reaction mixture was quenched with saturated Na_2_SO_3_ aqueous solution (2 ml). The aqueous phase was diluted with water (3 mL) and extracted with EtOAc (5 ml × 3). The combined organic extracts were dried over anhydrous Na_2_SO_4_, filtered, and concentrated under reduced pressure. The residue was purified over silica gel chromatography to afford **1–29**.

### General procedure for TEMPO-catalyzed debromination of alkenes and alkynes

Substrate **S30–S47** (0.50 mmol), DBDMH (0.75 mmol), TEMPO (0.10 mmol), and DCE (2.0 ml) was added to a reaction tube with a magnetic bar. The mixture was stirred at the indicated temperature for the specified reaction time. Upon completion of the reaction (monitored by TLC), the reaction mixture was quenched with saturated Na_2_SO_3_ aqueous solution (2 ml). The aqueous phase was diluted with water (3 ml) and extracted with EtOAc (5 ml × 3). The combined organic extracts were dried over anhydrous Na_2_SO_4_, filtered, and concentrated under reduced pressure. The residue was purified over silica gel chromatography to afford **30–47**.

### General procedure for TEMPO-catalyzed halogenation of (hetero)arenes

Substrate **S48–S69** (0.50 mmol), NCS (80.2 mg, 0.60 mmol), [TEMPO][OTf] (30.5 mg, 0.10 mmol), and CHCl_3_ (2.0 ml) was added to a reaction tube with a magnetic bar. The mixture was stirred at 25 °C for 12 h. Then, the reaction mixture was quenched with saturated Na_2_SO_3_ aqueous solution (2 ml). The aqueous phase was diluted with water (3 ml) and extracted with EtOAc (5 ml × 3). The combined organic extracts were dried over anhydrous Na_2_SO_4_, filtered, and concentrated under reduced pressure. The residue was purified over silica gel chromatography to afford **48–69**.

## Supplementary information

Supplementary Information

## Data Availability

All data that support the findings of this study are available in the online version of this paper in the accompanying Supplementary Information (including experimental procedures, compound characterization data).
