# Supplementary material for: Oxoammonium salts are catalysing efficient and selective halogenation of olefins, alkynes and aromatics
Source: Nat Commun. 2021 Jun 23;12:3873. doi: 10.1038/s41467-021-24174-w (PMC8222362; doi:10.1038/s41467-021-24174-w)
Supplement: Supplementary file 1 — Supplementary Information [file 41467_2021_24174_MOESM1_ESM.pdf]

Oxoammonium salts are catalysing efficient and selective halogenation of olefins, alkynes and aromatics

Weijin Wang,[†] Xinyao Li,[†] Xiaoxue Yang,[†] Lingsheng Ai,[†] Zhiwen Gong,[†] Ning Jiao,[†,‡]
Song Song,*[,†]

[†] State Key Laboratory of Natural and Biomimetic Drugs, School of Pharmaceutical Sciences, Peking University, Xue Yuan Rd. 38, Beijing 100191, China

[‡] State Key Laboratory of Organometallic Chemistry, Shanghai Institute of Organic Chemistry, Chinese Academy of Sciences, Shanghai 200032, China

Supplementary Information

Table of contents:

(A) General remarks……………………………………………………….…….S2

(B) TEMPO-catalysed haloarylation

(a) Preparation of starting materials..S2

(b) Optimization of reaction conditions………………………….….……....S5

(c) Experimental procedures…………………………………..……..….…S7

(C) TEMPO-catalysed dibromination of alkenes and alkynes………………......S24

(a) Optimization of reaction conditions...S24

(b) Experimental procedures………………………………………………… S25

(D) TEMPO^{+}-catalysed aromatic halogenation……………..……………………..S33

(E) Mechanistic experiments…………………………………………………...S45

(E) References……………………………………………………..………...S71

(F) NMR spectra of compounds……………………….……………………….S72

(A) General remarks

All commercially available compounds were purchased from Sigma-Aldrich, Alfa-Aesar, Acros, Energy Chemicals, and Beijing Chemical Works, Ltd. Unless otherwise noted, materials obtained from commercial suppliers were used without further purification. Analysis of crude reaction mixture was done on an Agilent 7890 GC System with an Agilent 5975 Mass Selective Detector. Products were purified by flash chromatography on silica gel. ^{1}H-NMR spectra were recorded on Bruker AVANCE III-400 spectrometers. Chemical shifts (in ppm) were referenced TMS in $CDCl_3$ (0 ppm) and TMS in DMSO-d_6 (0 ppm). ^{13}C-NMR spectra were obtained by using the same NMR spectrometers and were calibrated with $CDCl_3$ (δ = 77.0 ppm) and DMSO-d_6 (39.5 ppm). Mass spectra were recorded using a PE SCLEX QSTAR spectrometer. High resolution mass spectra were obtained with a Bruker APEX IV Fourier transform ion cyclotron resonance mass spectrometer.

(B) TEMPO-catalysed intramolecular haloarylation

(a) Preparation of starting materials

Procedure A: The starting materials **S1~S8** were prepared according to previously reported literatures.[1]

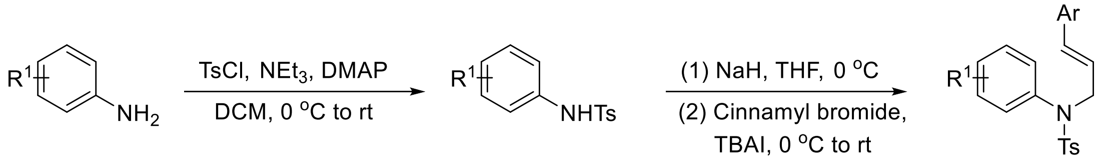

S1~S8

***N*-(4-(tert-butyl)phenyl)-*N*-cinnamyl-4-methylbenzenesulfonamide (S2)**

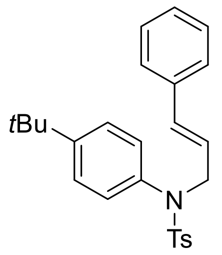

S2

White solid. ^{1}**H NMR** (400 MHz, Chloroform-*d*) δ 7.54 (d, *J* = 7.9 Hz, 2H), 7.31 – 7.17 (m, 9H), 7.00 (d, *J* = 8.4 Hz, 2H), 6.39 (d, *J* = 15.8 Hz, 1H), 6.10 (dt, *J* = 15.8, 6.5 Hz, 1H), 4.34 – 4.27 (m, 2H), 2.41 (s, 3H), 1.28 (s, 9H). ^{13}**C NMR** (101 MHz, Chloroform-*d*) δ 150.8, 143.2, 136.5, 136.4, 136.0, 133.4, 129.3, 128.4, 128.2, 127.7, 127.6, 126.4, 125.8, 124.4, 53.4, 34.5, 31.2, 21.5. **HRMS (ESI)** exact mass calc'd for $C_{26}H_{30}NO_2S$ ($[M+H]^+$): 420.1997; found *m/z*: 420.1994

***N*-cinnamyl-*N*-(4-fluorophenyl)-4-methylbenzenesulfonamide (S5)**

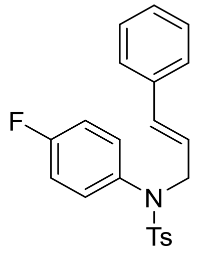

S5

White solid. ^{1}**H NMR** (400 MHz, Chloroform-*d*) δ 7.51 (d, *J* = 8.0 Hz, 2H), 7.29 – 7.16 (m, 7H), 7.03 (dd, *J* = 8.9, 4.9 Hz, 2H), 6.95 (t, *J* = 8.4 Hz, 2H), 6.35 (d, *J* = 15.8 Hz, 1H), 6.07 (dt, *J* = 15.8, 6.7 Hz, 1H), 4.30 (d, *J* = 6.7 Hz, 2H), 2.42 (s, 3H). ^{13}**C NMR** (101 MHz, Chloroform-*d*) δ 161.8 (d, *J* = 248.0 Hz), 143.6, 136.2, 135.2 (d, *J* = 38.5 Hz), 134.1, 130.8 (d, *J* = 8.8 Hz), 129.5, 128.5, 127.9, 127.7, 126.4, 123.8, 116.0, 115.8, 53.5, 21.5. ^{19}**F NMR** (376 MHz, Chloroform-*d*) δ -113.16 (ddd, *J* = 8.2, 5.4, 3.1 Hz). **HRMS (ESI)** exact mass calc'd for $C_{22}H_{21}FNO_2S$ ($[M+H]^+$): 382.1277; found *m/z*: 382.1280.

Procedure B: The starting materials **S9-S11** were prepared according to previously reported literatures.[1]

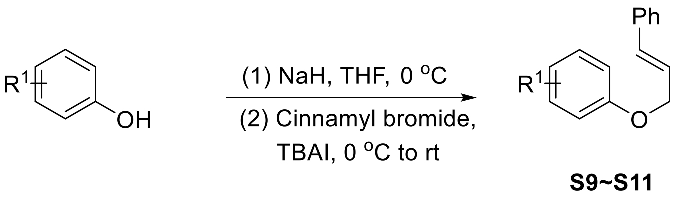

1-(tert-butyl)-4-(cinnamyloxy)benzene (S10)

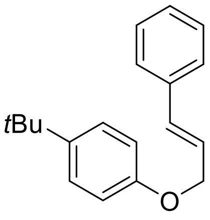

S10

White solid. **^{1}H NMR** (400 MHz, Chloroform-*d*) δ 7.43 – 7.36 (m, 2H), 7.36 – 7.27 (m, 4H), 7.26 – 7.19 (m, 1H), 6.94 – 6.86 (m, 2H), 6.72 (d, *J* = 16.0 Hz, 1H), 6.41 (dt, *J* = 16.0, 5.8 Hz, 1H), 4.67 (dd, *J* = 5.8, 1.6 Hz, 2H), 1.30 (s, 9H). **^{13}C NMR** (101 MHz, Chloroform-*d*) δ 156.4, 143.5, 136.5, 132.8, 128.5, 127.8, 126.5, 126.2, 124.8, 114.2, 68.6, 34.0, 31.5. **HRMS (EI)** exact mass calc'd for $C_{19}H_{22}O$ ([M]$^+$): 266.1665; found *m/z*: 266.1666.

1-(benzyloxy)-4-(cinnamyloxy)benzene (S11)

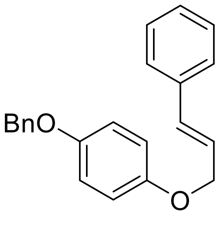

S11

White solid. **^{1}H NMR** (400 MHz, DMSO-d_6) δ 7.54 – 7.22 (m, 10H), 7.01 – 6.87 (m, 4H), 6.74 (d, *J* = 16.0 Hz, 1H), 6.49 (dt, *J* = 16.0, 5.8 Hz, 1H), 5.03 (s, 2H), 4.65 (dd, *J* = 5.8, 1.5 Hz, 2H). **^{13}C NMR** (101 MHz, DMSO-d_6) δ 152.5, 152.4, 137.3, 136.2, 132.1, 128.6, 128.4, 127.8, 127.7, 127.6, 126.4, 125.3, 115.64, 115.59, 69.6, 68.5. **HRMS (EI)** exact mass calc'd for $C_{22}H_{20}O_2$ ([M]$^+$): 316.1458; found *m/z*: 316.1460.

Procedure C: The starting material **S12** were prepared according to previously reported literatures.[2]

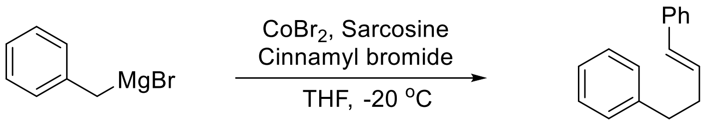

S12

(b) Optimization of reaction conditions

Substrate **S1** (0.10 mmol), DXDMH (0.12 mmol), catalyst (0.01mmol) and MeCN (2.0 mL) were added to a reaction tube with a magnetic bar. The mixture was stirred at 25 °C for 12 h. Then, the reaction mixture was quenched with saturated Na_2SO_3 aqueous solution (2 mL). The aqueous phase was diluted with water (3 mL) and extracted with EtOAc (5 mL × 3). The combined organic extracts were dried over anhydrous Na_2SO_4, filtered, and concentrated under reduced pressure. The residue was analyzed by NMR before purified over silica gel chromatography.

Supplementary Table 1. Optimization of chloroarylation[a]

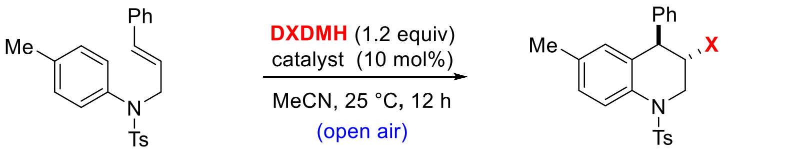

S1 **1**, X = Cl **2**, X = Br **3**, X = I

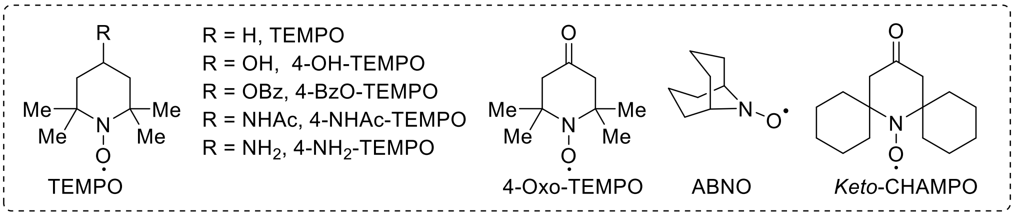

Entry	X	catalyst	Yield	Entry	X	catalyst	Yield
1	Cl	-	trace	16	Cl	Quinoline *N*-oxide	38%
2	Cl	2,4,6-$(Me)_3PhNH_2$	44%	17	Cl	TEMPO	64%
3	Cl	Ph_3P=S	38%	18	Cl	4-OH-TEMPO	61%
4	Cl	$(Me_2N)_2C$=S	33%	19	Cl	4-BzO-TEMPO	55%
5	Cl	Ph_2S	35%	20	Cl	4-Oxo-TEMPO	63%
6	Cl	Ph_2Se	29%	21	Cl	4-NHAc-TEMPO	70%
7	Cl	*n*Bu_3P	48%	**22**	**Cl**	**4-NH_2-TEMPO**	**75% (74%)**[b]
8	Cl	TMSOTf	trace	23	Cl	ABNO	57%
9	Cl	TfOH	trace	24	Cl	*Keto*-CHAMPO	40%
10	Cl	DMSO	35%	**25**	**Br**	**4-Oxo-TEMPO**	**92% (88%)**[b]
11	Cl	Ph_2S=O	47%	26	Br	4-NH_2-TEMPO	72%
12	Cl	Bn_2S=O	40%	27	Br	ABNO	78%
13	Cl	$MeNO_2$	10%	28	Br	*Keto*-CHAMPO	60%
14	Cl	Py *N*-oxide	42%	**29**	**I**	**4-Oxo-TEMPO**	**62% (60%)**[b]
15	Cl	4-NO_2Py *N*-oxide	37%	30	I	4-NH_2-TEMPO	43%

[a] Reactions were carried out with **S1** (0.10 mmol), catalyst (0.01 mmol) and DCDMH (0.12 mmol) in MeCN (1.0 mL) for 12 h at 25°C under air. Yields were determined by ^{1}H-NMR using 1,1,2,2-tetrachloroethane as internal standard. [b] Yield of isolated **1** for the reaction at 0.20 mmol scale. [c] Reaction was carried out under Ar. [d] 20 mol% of 4-NH_2-TEMPO was used.

DCDMH = 1,3-dichloro-5,5-dimethylhydantoin, TsOH = p-toluenesulfonic acid , TMSOTf = trimethylsilyl trifluoromethylsulfonate.

Supplementary Table 2. Optimization of bromoarylation of **S1**[a]

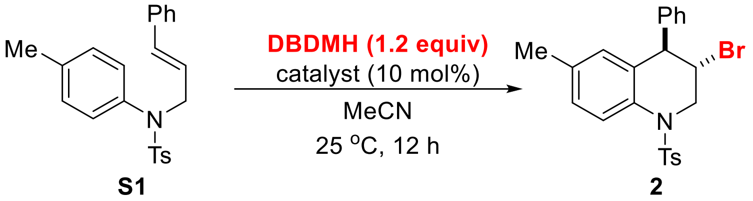

Entry	Catalyst	Yield
1	4-NH_2-TEMPO	72%
**2**	**4-Oxo-TEMPO**	**92% (88%)[b]**
3	4-OH-TEMPO	65%
4	4-NHAc-TEMPO	63%
5	4-BzO-TEMPO	52%
6	4-OMe-TEMPO	31%
7	TEMPO	62%
8	Ph_3P=S	54%
9	DMSO	52%
10	$PhNO_2$	62%
11	*L*-proline	52%
12	$MeNO_2$	60%
13	Ph_3P	69%

[a] Reactions were carried out with **S1** (0.10 mmol), catalyst (0.01 mmol) and DBDMH (0.12 mmol) in MeCN (1.0 mL) for 12 h at 25°C under air. Yields were determined by ^{1}H-NMR using 1,1,2,2-tetrachloroethane as internal standard. [b] Yield of isolated **2** for the reaction at 0.20 mmol scale.

DBDMH = 1,3-dibromo-5,5-dimethylhydantoin

Supplementary Table 3. Optimization of iodoarylation[a]

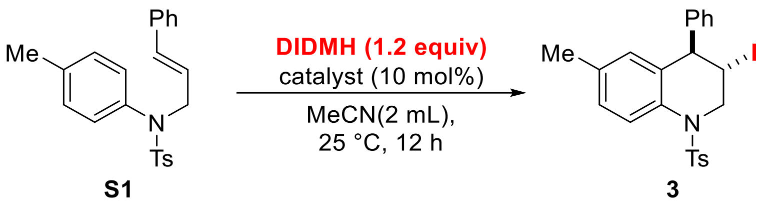

Entry	catalyst	Yield
**1**	**4-Oxo-TEMPO**	**62% (60%)[b]**
2	TEMPO	21%
3	4-OH-TEMPO	53%
4	4-NH_2-TEMPO	43%
5	4-NHAc-TEMPO	32%

[a] Reactions were carried out with **S1** (0.10 mmol), catalyst (0.01 mmol) and DIDMH (0.12 mmol) in MeCN (1.0 mL) for 12 h at 25°C under air. Yields were determined by ^{1}H-NMR using 1,1,2,2-tetrachloroethane as internal standard. [b] Yield of isolated **3** for the reaction at 0.20 mmol scale.

DIDMH = 1,3-diindo-5,5-dimethylhydantoin

(c) Experimental procedures

Substrate **S1-S12** (0.20 mmol), DXDMH (0.24 mmol), the catalyst (0.02 mmol) and the solvent were added to a reaction tube with a magnetic bar. The mixture was stirred at 25 °C for the specified reaction time. Upon completion of the reaction (monitored by TLC), the reaction mixture was quenched with saturated Na_2SO_3 aqueous solution (2 mL). The aqueous phase was diluted with water (3 mL) and extracted with EtOAc (5 mL × 3). The combined organic extracts were dried over anhydrous Na_2SO_4, filtered, and concentrated under reduced pressure. The residue was purified over silica gel chromatography to afford **1-29**.

3-chloro-6-methyl-4-phenyl-1-tosyl-1,2,3,4-tetrahydroquinoline (1)

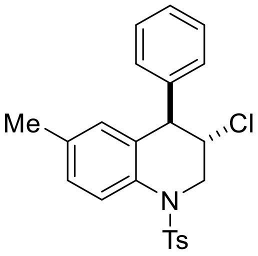

1

The reaction of **S1** (75.4 mg, 0.20 mmol), DCDMH (47.3 mg, 0.24 mmol) and 4-NH_2-TEMPO (3.4 mg, 0.02 mmol) at 25°C for 12 h afforded 60.1 mg (73%) of **1** as a

white solid (petroleum ether/ethyl acetate = 10:1). **^{1}H NMR** (400 MHz, Chloroform-*d*) δ 7.78 (d, *J* = 8.4 Hz, 1H), 7.64 – 7.55 (m, 2H), 7.28 (d, *J* = 8.1 Hz, 2H), 7.24 – 7.18 (m, 1H), 7.14 (dd, *J* = 8.1, 6.5 Hz, 2H), 7.03 (dd, *J* = 8.5, 2.1 Hz, 1H), 6.56 – 6.49 (m, 2H), 6.48 – 6.42 (m, 1H), 4.63 (dd, *J* = 14.0, 3.9 Hz, 1H), 3.93 (d, *J* = 9.5 Hz, 1H), 3.78 (ddd, *J* = 11.3, 9.5, 3.9 Hz, 1H), 3.61 (dd, *J* = 13.9, 11.2 Hz, 1H), 2.44 (s, 3H), 2.14 (s, 3H). **^{13}C NMR** (101 MHz, Chloroform-*d*) δ 144.2, 141.5, 136.5, 135.7, 133.1, 131.3, 130.6, 130.0, 128.8, 128.3, 128.2, 127.4, 127.2, 124.6, 57.3, 54.0, 52.0, 21.5, 20.7. **HRMS (ESI)** exact mass calc'd for $C_{23}H_{23}ClNO_2S$ ([M+H]$^+$): 412.1138; found *m/z*: 412.1138, 414.1111 (For ^{37}Cl).

3-bromo-6-methyl-4-phenyl-1-tosyl-1,2,3,4-tetrahydroquinoline (2)[1]

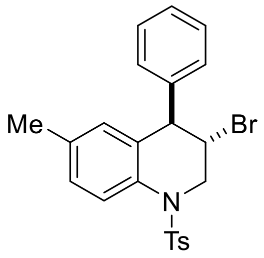

2

The reaction of **S1** (75.4 mg, 0.20 mmol), DBDMH (68.6 mg, 0.24 mmol) and 4-Oxo-TEMPO (3.4 mg, 0.02 mmol) at 25°C for 8 h afforded 80.3 mg (88%) of **2** as a white solid (petroleum ether/ethyl acetate = 10:1). **^{1}H NMR** (400 MHz, Chloroform-*d*) δ 7.76 (d, *J* = 8.5 Hz, 1H), 7.62 – 7.57 (m, 2H), 7.29 (d, *J* = 8.0 Hz, 2H), 7.24 – 7.18 (m, 1H), 7.14 (dd, *J* = 8.2, 6.6 Hz, 2H), 7.03 (dd, *J* = 8.4, 2.1 Hz, 1H), 6.54 – 6.47 (m, 2H), 6.47 – 6.41 (m, 1H), 4.73 (dd, *J* = 13.9, 3.8 Hz, 1H), 4.10 (d, *J* = 9.7 Hz, 1H), 3.86 (ddd, *J* = 11.7, 9.7, 3.8 Hz, 1H), 3.73 (dd, *J* = 13.9, 11.8 Hz, 1H), 2.45 (s, 3H), 2.14 (s, 3H). **^{13}C NMR** (101 MHz, Chloroform-*d*) δ 144.2, 141.8, 136.6, 135.8, 133.1, 131.6, 130.5, 130.0, 128.7, 128.3, 128.2, 127.4, 127.2, 124.8, 54.3, 52.7, 49.5, 21.5, 20.8. **MS (ESI)** m/z (%): 214.2 (80), 376.1 (100), 456.1 ([M+H]$^+$, 30), 458.1 ([M+H]$^+$, 30, for ^{81}Br).

3-iodo-6-methyl-4-phenyl-1-tosyl-1,2,3,4-tetrahydroquinoline (3)

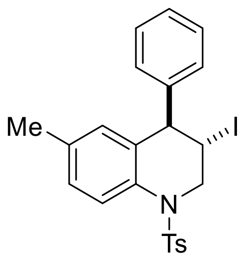

3

The reaction of **S1** (75.4 mg, 0.20 mmol), DIDMH (118.3 mg, 0.24 mmol) and 4-Oxo-TEMPO (3.4 mg, 0.02 mmol) at 25°C for 24 h afforded 62.4 mg (62%) of **3** as a white solid (petroleum ether/ethyl acetate = 10:1). **^{1}H NMR** (400 MHz, Chloroform-*d*) δ 7.74 (d, *J* = 8.5 Hz, 1H), 7.63 – 7.54 (m, 2H), 7.30 (d, *J* = 8.0 Hz, 2H), 7.24 – 7.17 (m, 1H), 7.13 (dd, *J* = 8.1, 6.8 Hz, 2H), 7.02 (dd, *J* = 8.4, 2.1 Hz, 1H), 6.52 – 6.45 (m, 2H), 6.40 (d, *J* = 2.1 Hz, 1H), 4.82 (dd, *J* = 13.8, 3.4 Hz, 1H), 4.20 (d, *J* = 10.0 Hz, 1H), 3.96 (ddd, *J* = 12.1, 10.1, 3.4 Hz, 1H), 3.85 (dd, *J* = 13.8, 12.3 Hz, 1H), 2.46 (s, 3H), 2.13 (s, 3H). **^{13}C NMR** (101 MHz, Chloroform-*d*) δ 144.2, 142.4, 136.8, 135.8, 133.2, 131.6, 130.6, 130.1, 128.6, 128.4, 128.2, 127.4, 127.3, 125.1, 55.5, 54.9, 29.1, 21.6, 20.8. **HRMS (ESI)** exact mass calc'd for $C_{23}H_{23}NO_2S$ ($[M+H]^+$): 504.0494; found *m/z*: 504.0492.

6-(tert-butyl)-3-chloro-4-phenyl-1-tosyl-1,2,3,4-tetrahydroquinoline (4)

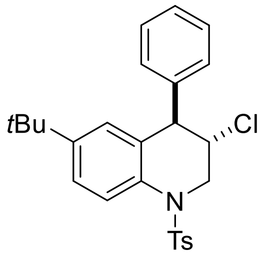

4

The reaction of **S2** (83.9 mg, 0.20 mmol), DCDMH (47.3 mg, 0.24 mmol) and 4-NH_2-TEMPO (3.4 mg, 0.02 mmol) at 25°C for 4 h afforded 63.6 mg (70%) of **4** as a white solid (petroleum ether/ethyl acetate = 10:1). **^{1}H NMR** (400 MHz, Chloroform-*d*) δ 7.76 (d, *J* = 8.7 Hz, 1H), 7.61 (d, *J* = 8.1 Hz, 2H), 7.28 (d, *J* = 8.0 Hz, 2H), 7.24 (dd, *J* = 8.9, 2.3 Hz, 1H), 7.22 – 7.18 (m, 1H), 7.15 (dd, *J* = 8.1, 6.3 Hz, 2H), 6.63 (d, *J* = 2.3 Hz, 1H), 6.61 – 6.52 (m, 2H), 4.69 (dd, *J* = 13.9, 3.8 Hz, 1H), 4.16 (d, *J* = 9.5 Hz,

1H), 3.89 (ddd, J = 11.6, 9.5, 3.7 Hz, 1H), 3.76 (dd, J = 13.9, 11.6 Hz, 1H), 2.44 (s, 3H), 1.11 (s, 9H). **^{13}C NMR** (101 MHz, Chloroform-*d*) δ 148.8, 144.2, 141.9, 136.7, 133.1, 131.0, 130.0, 128.7, 128.3, 127.3, 127.2, 127.1, 124.5, 124.1, 54.4, 52.6, 49.6, 34.2, 31.0, 21.5. **HRMS (ESI)** exact mass calc'd for $C_{26}H_{29}ClNO_2S$ ($[M+H]^+$): 454.1608; found *m/z*: 454.1602, 456.1575 (For ^{37}Cl).

3-bromo-6-(tert-butyl)-4-phenyl-1-tosyl-1,2,3,4-tetrahydroquinoline (5)

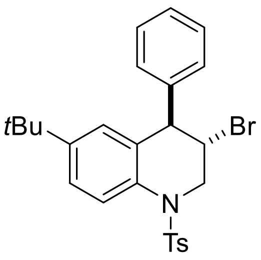

5

The reaction of **S2** (83.9 mg, 0.20 mmol), DBDMH (68.6 mg, 0.24 mmol) and 4-Oxo-TEMPO (3.4 mg, 0.02 mmol) at 25°C for 2 h afforded 89.7 mg (95%) of **5** as a white solid (petroleum ether/ethyl acetate = 10:1). **^{1}H NMR** (400 MHz, Chloroform-*d*) δ 7.78 (d, J = 8.7 Hz, 1H), 7.65 – 7.58 (m, 2H), 7.30 – 7.26 (m, 2H), 7.26 – 7.22 (m, 1H), 7.22 – 7.18 (m, 1H), 7.19 – 7.12 (m, 2H), 6.65 (dd, J = 2.3, 1.0 Hz, 1H), 6.63 – 6.56 (m, 2H), 4.59 (dd, J = 13.8, 3.9 Hz, 1H), 4.00 (d, J = 9.3 Hz, 1H), 3.81 (ddd, J = 11.1, 9.2, 3.8 Hz, 1H), 3.65 (dd, J = 13.9, 11.1 Hz, 1H), 2.44 (s, 3H), 1.12 (s, 9H). **^{13}C NMR** (101 MHz, Chloroform-*d*) δ 148.7, 144.2, 141.7, 136.6, 133.1, 130.7, 130.0, 128.8, 128.3, 127.4, 127.44, 127.39, 124.5, 123.9, 57.5, 54.1, 51.9, 34.2, 31.0, 21.5. **HRMS (ESI)** exact mass calc'd for $C_{26}H_{29}BrNO_2S$ ($[M+H]^+$): 498.1102; found *m/z*: 498.1080, 500.1059 (for ^{81}Br).

6-(tert-butyl)-3-iodo-4-phenyl-1-tosyl-1,2,3,4-tetrahydroquinoline (6)

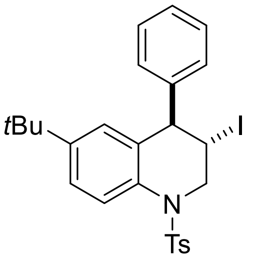

6

The reaction of **S2** (83.9 mg, 0.20 mmol), DIDMH (118.3 mg, 0.24 mmol) and 4-Oxo-TEMPO (3.4 mg, 0.02 mmol) at 25°C for 12 h afforded 75.3 mg (69%) of **6** as a white solid (petroleum ether/ethyl acetate = 10:1). **^{1}H NMR** (400 MHz, Chloroform-*d*) δ 7.74 (d, *J* = 8.6 Hz, 1H), 7.60 (d, *J* = 7.9 Hz, 2H), 7.29 (d, *J* = 7.9 Hz, 2H), 7.25 – 7.17 (m, 2H), 7.14 (t, *J* = 7.2 Hz, 2H), 6.59 (d, *J* = 2.3 Hz, 1H), 6.57 – 6.50 (m, 2H), 4.78 (dd, *J* = 13.8, 3.5 Hz, 1H), 4.26 (d, *J* = 10.0 Hz, 1H), 4.05 – 3.93 (m, 1H), 3.88 (dd, *J* = 14.0, 12.0 Hz, 1H), 2.45 (s, 3H), 1.10 (s, 9H). **^{13}C NMR** (101 MHz, Chloroform-*d*) δ 148.8, 144.2, 142.5, 136.9, 133.1, 131.1, 130.0, 128.6, 128.3, 127.3, 127.2, 127.1, 124.4, 124.4, 55.6, 54.8, 34.2, 31.0, 29.2, 21.6. **HRMS (ESI)** exact mass calc'd for $C_{26}H_{29}INO_2S$ ([M+H]$^+$): 546.0963; found *m/z*: 546.0964.

3-chloro-6-methoxy-4-phenyl-1-tosyl-1,2,3,4-tetrahydroquinoline (7)

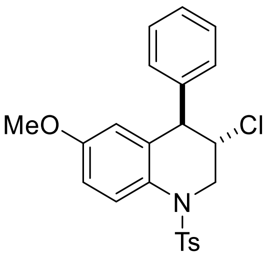

7

The reaction of **S3** (78.7 mg, 0.20 mmol), DCDMH (47.3 mg, 0.24 mmol) and 4-NH_2-TEMPO (3.4 mg, 0.02 mmol) at 25°C for 4 h afforded 71.9 mg (84%) of **7** as a white solid (petroleum ether/ethyl acetate = 8:1). **^{1}H NMR** (400 MHz, Chloroform-*d*) δ 7.83 (d, *J* = 9.1 Hz, 1H), 7.63 – 7.54 (m, 2H), 7.29 (d, *J* = 8.1 Hz, 2H), 7.23 – 7.17 (m, 1H), 7.13 (dd, *J* = 8.1, 6.5 Hz, 2H), 6.80 (dd, *J* = 9.1, 3.0 Hz, 1H), 6.54 – 6.44 (m, 2H), 6.18 – 6.07 (m, 1H), 4.66 (dd, *J* = 14.0, 4.0 Hz, 1H), 3.90 (d, *J* = 9.8 Hz, 1H), 3.75 (ddd, *J* = 11.5, 9.7, 4.0 Hz, 1H), 3.62 (s, 3H), 3.58 (dd, *J* = 10.9, 3.0 Hz, 1H), 2.45 (s, 3H). **^{13}C NMR** (101 MHz, Chloroform-*d*) δ 157.5, 144.2, 141.1, 136.4, 133.5, 130.0, 128.8, 128.7, 128.3, 127.5, 127.4, 126.5, 114.9, 113.1, 57.1, 55.3, 54.2, 52.3, 21.5. **HRMS (ESI)** exact mass calc'd for $C_{23}H_{23}ClNO_3S$ ([M+H]$^+$): 428.1087; found *m/z*: 428.1086, 430.1059 (For ^{37}Cl).

3-bromo-6-methoxy-4-phenyl-1-tosyl-1,2,3,4-tetrahydroquinoline (8)[1]

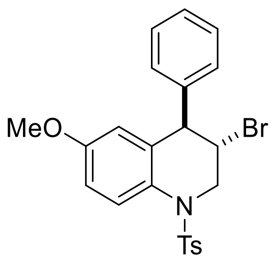

8

The reaction of **S3** (78.7 mg, 0.20 mmol), DBDMH (68.6 mg, 0.24 mmol) and 4-Oxo-TEMPO (3.4 mg, 0.02 mmol) at 25°C for 1 hour afforded 89.8 mg (95%) of **8** as a white solid (petroleum ether/ethyl acetate = 10:1). **^{1}H NMR** (400 MHz, Chloroform-*d*) δ 7.81 (d, *J* = 9.1 Hz, 1H), 7.62 – 7.53 (m, 2H), 7.29 (d, *J* = 8.0 Hz, 2H), 7.23 – 7.17 (m, 1H), 7.12 (t, *J* = 7.4 Hz, 2H), 6.79 (dd, *J* = 9.1, 3.0 Hz, 1H), 6.52 – 6.41 (m, 2H), 6.12 (d, *J* = 2.9 Hz, 1H), 4.75 (dd, *J* = 13.9, 3.8 Hz, 1H), 4.06 (d, *J* = 9.9 Hz, 1H), 3.84 (ddd, *J* = 12.0, 9.8, 3.8 Hz, 1H), 3.72 (dd, *J* = 13.9, 12.0 Hz, 1H), 3.60 (s, 3H), 2.45 (s, 3H). **^{13}C NMR** (101 MHz, Chloroform-*d*) δ 157.4, 144.2, 141.3, 136.4, 133.6, 130.0, 128.65, 128.57, 128.3, 127.4, 127.3, 126.5, 114.8, 113.1, 55.2, 54.4, 52.8, 49.1, 21.5. **MS (ESI)** m/z (%): 226.1 (100), 319.1 (20), 472.1 ([M+H]$^+$, 20), 474.1 ([M+H]$^+$, 20, for ^{81}Br).

3-iodo-6-methoxy-4-phenyl-1-tosyl-1,2,3,4-tetrahydroquinoline (9)

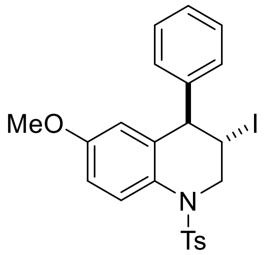

9

The reaction of **S3** (78.7 mg, 0.20 mmol), DIDMH (118.3 mg, 0.24 mmol) and 4-Oxo-TEMPO (3.4 mg, 0.02 mmol) at 25°C for 12 h afforded 74.8 mg (72%) of **9** as a white solid (petroleum ether/ethyl acetate = 10:1). **^{1}H NMR** (400 MHz, Chloroform-*d*) δ 7.79 (d, *J* = 9.1 Hz, 1H), 7.57 (d, *J* = 8.1 Hz, 2H), 7.31 (d, *J* = 8.0 Hz, 2H), 7.20 (t, *J* = 7.3 Hz, 1H), 7.12 (t, *J* = 7.5 Hz, 2H), 6.78 (dd, *J* = 9.1, 3.0 Hz, 1H), 6.44 (d, *J* = 7.5 Hz, 2H), 6.09 (d, *J* = 3.0 Hz, 1H), 4.84 (dd, *J* = 13.6, 3.2 Hz, 1H), 4.17 (d, *J* = 10.1 Hz,

1H), 3.94 (ddd, J = 13.2, 10.0, 3.2 Hz, 1H), 3.90 – 3.81 (m, 1H), 3.60 (s, 3H), 2.47 (s, 3H). **^{13}C NMR** (101 MHz, Chloroform-*d*) δ 157.4, 144.2, 142.0, 136.7, 133.5, 130.1, 128.8, 128.6, 128.4, 127.5, 127.4, 126.9, 115.0, 113.1, 55.6, 55.3, 55.1, 28.7, 21.6. **HRMS (ESI)** exact mass calc'd for $C_{23}H_{23}INO_3S$ ([M+Na]$^+$): 542.0258; found *m/z*: 542.0263.

3-chloro-4-phenyl-1-tosyl-1,2,3,4-tetrahydroquinoline (10)

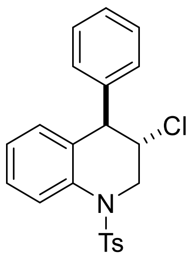

10

The reaction of **S4** (72.7 mg, 0.20 mmol), DCDMH (47.3 mg, 0.24 mmol) and 4-NH_2-TEMPO (3.4 mg, 0.02 mmol) at 25°C for 4 h afforded 49.3 mg (62%) of **10** as a white solid (petroleum ether/ethyl acetate = 10:1). **^{1}H NMR** (400 MHz, Chloroform-*d*) δ 7.91 (d, J = 8.3 Hz, 1H), 7.60 (d, J = 8.1 Hz, 2H), 7.29 (d, J = 8.1 Hz, 2H), 7.25 – 7.18 (m, 2H), 7.15 (t, J = 7.4 Hz, 2H), 7.03 (t, J = 7.5 Hz, 1H), 6.68 (d, J = 7.8 Hz, 1H), 6.56 – 6.52 (m, 2H), 4.66 (dd, J = 14.0, 3.9 Hz, 1H), 3.99 (d, J = 9.4 Hz, 1H), 3.82 (ddd, J = 13.3, 9.5, 3.9 Hz, 1H), 3.66 (dd, J = 14.0, 11.3 Hz, 1H), 2.45 (s, 3H). **^{13}C NMR** (101 MHz, Chloroform-*d*) δ 144.3, 141.5, 136.5, 135.7, 131.6, 130.4, 130.0, 128.9, 128.4, 127.40, 127.35, 125.9, 124.7, 57.1, 54.1, 52.0, 21.5. **HRMS (ESI)** exact mass calc'd for $C_{22}H_{21}ClNO_2S$ ([M+H]$^+$): 398.0982; found *m/z*: 398.0975, 400.0948 (For ^{37}Cl).

3-bromo-4-phenyl-1-tosyl-1,2,3,4-tetrahydroquinoline (11)[1]

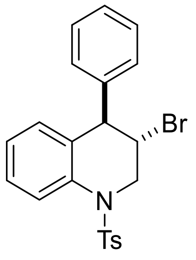

11

The reaction of **S4** (72.7 mg, 0.20 mmol), DBDMH (68.6 mg, 0.24 mmol) and 4-Oxo-TEMPO (3.4 mg, 0.02 mmol) at 25°C for 4 h afforded 72.6 mg (82%) of **11** as a white solid (petroleum ether/ethyl acetate = 10:1). **^{1}H NMR** (400 MHz, Chloroform-*d*) δ 7.89 (dd, *J* = 8.3, 1.3 Hz, 1H), 7.64 – 7.57 (m, 2H), 7.29 (d, *J* = 8.1 Hz, 2H), 7.25 – 7.18 (m, 2H), 7.14 (t, *J* = 7.3 Hz, 2H), 7.02 (td, *J* = 7.5, 1.3 Hz, 1H), 6.65 (dt, *J* = 7.9, 1.3 Hz, 1H), 6.56 – 6.47 (m, 2H), 4.76 (dd, *J* = 13.9, 3.8 Hz, 1H), 4.15 (d, *J* = 9.7 Hz, 1H), 3.90 (ddd, *J* = 11.8, 9.6, 3.8 Hz, 1H), 3.77 (dd, *J* = 13.9, 11.7 Hz, 1H), 2.45 (s, 3H). **^{13}C NMR** (101 MHz, Chloroform-*d*) δ 144.3, 141.7, 136.6, 135.7, 131.9, 130.4, 130.1, 128.8, 128.4, 127.38, 127.35, 127.3, 126.0, 124.8, 54.4, 52.7, 49.2, 21.6. **MS (ESI)** m/z (%): 142.1 (30), 362.1 (100), 442.1 ($[M+H]^+$, 40), 444.1 ($[M+H]^+$, 40, for ^{81}Br).

3-iodo-4-phenyl-1-tosyl-1,2,3,4-tetrahydroquinoline (12)

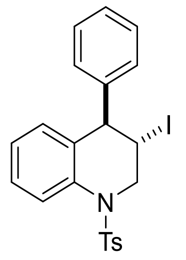

12

The reaction of **S4** (72.7 mg, 0.20 mmol), DIDMH (118.3 mg, 0.24 mmol) and 4-Oxo-TEMPO (3.4 mg, 0.02 mmol) at 25°C for 24 h afforded 50.9 mg (52%) of **12** as a white solid (petroleum ether/ethyl acetate = 10:1). **^{1}H NMR** (400 MHz, Chloroform-*d*) δ 7.86 (dd, *J* = 8.4, 1.3 Hz, 1H), 7.62 – 7.57 (m, 2H), 7.33 – 7.28 (m, 2H), 7.24 – 7.18 (m, 2H), 7.18 – 7.10 (m, 2H), 7.00 (td, *J* = 7.6, 1.3 Hz, 1H), 6.62 (dt, *J* = 7.9, 1.3 Hz, 1H), 6.53 – 6.46 (m, 2H), 4.85 (dd, *J* = 13.8, 3.4 Hz, 1H), 4.26 (d, *J* = 10.1 Hz, 1H), 4.00 (ddd, *J* = 12.2, 10.1, 3.5 Hz, 1H), 3.89 (dd, *J* = 13.8, 12.2 Hz, 1H), 2.46 (s, 3H). **^{13}C NMR** (101 MHz, Chloroform-*d*) δ 144.4, 142.3, 136.8, 135.8, 131.8, 130.4, 130.1, 128.6, 128.4, 127.4, 127.3, 126.0, 125.1, 55.5, 54.9, 28.7, 21.6. **HRMS (ESI)** exact mass calc'd for $C_{22}H_{21}INO_2S$ ($[M+H]^+$): 490.0337; found *m/z*: 490.0333.

3-chloro-6-fluoro-4-phenyl-1-tosyl-1,2,3,4-tetrahydroquinoline (13)

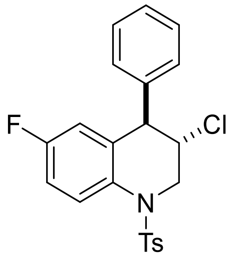

13

The reaction of **S5** (76.3 mg, 0.20 mmol), DCDMH (65.7 mg, 0.30 mmol) and 4-NH_2-TEMPO (6.8 mg, 0.04 mmol) in $MeNO_2$ (3 mL) at 60°C for 24 h afforded 29.9 mg (36%) of **13** as a pale yellow solid (petroleum ether/ethyl acetate = 10:1). **^{1}H NMR** (400 MHz, Chloroform-*d*) δ 7.89 (dd, *J* = 9.2, 5.3 Hz, 1H), 7.58 (d, *J* = 8.4 Hz, 2H), 7.31 (d, *J* = 8.1 Hz, 2H), 7.25 – 7.21 (m, 1H), 7.19 – 7.13 (m, 2H), 6.99 – 6.92 (m, 1H), 6.50 (d, *J* = 6.8 Hz, 2H), 6.36 (ddd, *J* = 9.2, 2.9, 1.1 Hz, 1H), 4.67 (dd, *J* = 14.1, 3.9 Hz, 1H), 3.92 (d, *J* = 9.3 Hz, 1H), 3.77 (ddd, *J* = 11.6, 9.7, 4.0 Hz, 1H), 3.62 (dd, *J* = 14.0, 11.4 Hz, 1H), 2.46 (s, 3H). **^{13}C NMR** (101 MHz, Chloroform-*d*) δ 160.4 (d, *J* = 246.1 Hz), 144.5, 140.6, 136.2, 134.2 (d, *J* = 8.0 Hz), 131.7 (d, *J* = 3.5 Hz), 130.2, 128.8, 128.6, 127.7, 127.5, 126.9 (d, *J* = 8.1 Hz), 116.5 (d, *J* = 23.1 Hz), 114.8 (d, *J* = 22.4 Hz), 56.5, 54.2, 52.2, 21.6. **^{19}F NMR** (376 MHz, Chloroform-*d*) δ -115.3 (td, *J* = 8.7, 4.8 Hz). **HRMS (EI)** exact mass calc'd for $C_{22}H_{19}ClFNO_2S$ ($[M]^+$): 416.0804; found *m/z*: 415.0805, 417.0778 (For ^{37}Cl).

3-bromo-6-fluoro-4-phenyl-1-tosyl-1,2,3,4-tetrahydroquinoline (14)

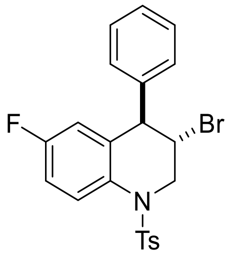

14

The reaction of **S5** (76.3 mg, 0.20 mmol), DBDMH (85.8 mg, 0.30 mmol) and 4-Oxo-TEMPO (6.8 mg, 0.04 mmol) at 60°C in $MeNO_2$ (3 mL) for 24 h afforded 54.3 mg (59%) of **14** as a white solid (petroleum ether/ethyl acetate = 10:1). **^{1}H NMR** (400 MHz, Chloroform-*d*) δ 7.87 (dd, *J* = 9.2, 5.2 Hz, 1H), 7.58 (d, *J* = 8.1 Hz, 2H), 7.32 (d,

J = 8.1 Hz, 2H), 7.23 (t, J = 7.3 Hz, 1H), 7.15 (t, J = 7.5 Hz, 2H), 6.94 (ddd, J = 9.9, 7.9, 3.0 Hz, 1H), 6.52 – 6.44 (m, 2H), 6.34 (dd, J = 9.3, 3.0 Hz, 1H), 4.76 (dd, J = 13.9, 3.7 Hz, 1H), 4.08 (d, J = 9.8 Hz, 1H), 3.84 (ddd, J = 11.6, 9.8, 3.7 Hz, 1H), 3.74 (dd, J = 13.9, 11.9 Hz, 1H), 2.47 (s, 3H). **^{13}C NMR** (101 MHz, Chloroform-*d*) δ 160.4 (d, J = 248.2 Hz), 144.6, 140.9, 136.4, 134.3 (d, J = 7.3 Hz), 131.7, 130.2, 128.7, 128.6, 127.7, 127.5, 127.1 (d, J = 8.4 Hz), 116.4 (d, J = 22.9 Hz), 114.8 (d, J = 22.6 Hz), 54.5, 52.8, 48.4, 21.6. **^{19}F NMR** (376 MHz, Chloroform-*d*) δ -115.2 (td, J = 8.7, 5.2 Hz). **HRMS (ESI)** exact mass calc'd for $C_{22}H_{20}BrFNO_2S$ ([M+H]$^+$): 460.0382; found *m/z*: 460.0383, 462.0363 (for ^{81}Br).

3,6-dichloro-4-phenyl-1-tosyl-1,2,3,4-tetrahydroquinoline (15)

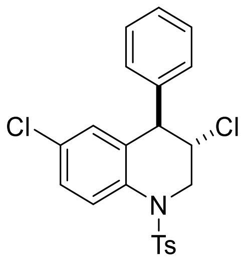

15

The reaction of **S6** (79.6 mg, 0.20 mmol), DCDMH (65.7 mg, 0.30 mmol) and 4-NH_2-TEMPO (6.8 mg, 0.04 mmol) in $MeNO_2$ (3.0 mL) at 60°C for 24 h afforded 41.5 mg (48%) of **15** as a pale yellow solid (petroleum ether/ethyl acetate = 10:1). **^{1}H NMR** (400 MHz, Chloroform-*d*) δ 7.89 (d, J = 8.9 Hz, 1H), 7.66 – 7.60 (m, 2H), 7.34 (d, J = 8.0 Hz, 2H), 7.27 – 7.15 (m, 4H), 6.68 (dd, J = 2.5, 1.0 Hz, 1H), 6.59 – 6.52 (m, 2H), 4.65 (dd, J = 14.0, 3.8 Hz, 1H), 3.96 (d, J = 9.3 Hz, 1H), 3.79 (ddd, J = 11.2, 9.3, 3.8 Hz, 1H), 3.66 (dd, J = 13.9, 11.1 Hz, 1H), 2.49 (s, 3H). **^{13}C NMR** (101 MHz, Chloroform-*d*) δ 144.6, 140.6, 136.2, 134.4, 133.2, 131.5, 130.2, 130.1, 128.8, 128.6, 127.7, 127.4, 126.0, 56.6, 53.9, 51.9, 21.6. **HRMS (ESI)** exact mass calc'd for $C_{22}H_{20}Cl_2NO_2S$ ([M+H]$^+$): 432.0592; found *m/z*: 432.0593, 434.0566 (For ^{37}Cl).

3-bromo-6-chloro-4-phenyl-1-tosyl-1,2,3,4-tetrahydroquinoline (16)[1]

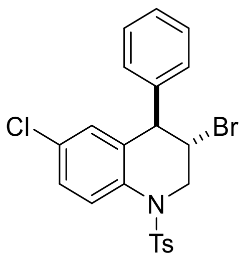

16

The reaction of **S6** (79.6 mg, 0.20 mmol), DBDMH (85.8 mg, 0.30 mmol) and 4-Oxo-TEMPO (6.8 mg, 0.04 mmol) at 60°C in $MeNO_2$ (3 mL) for 12 h afforded 47.7 mg (50%) of **16** as a white solid (petroleum ether/ethyl acetate = 10:1). **^{1}H NMR** (400 MHz, Chloroform-*d*) δ 7.85 (d, *J* = 8.9 Hz, 1H), 7.60 (d, *J* = 8.4 Hz, 2H), 7.35 – 7.30 (m, 2H), 7.26 – 7.12 (m, 4H), 6.63 (dd, *J* = 2.5, 1.0 Hz, 1H), 6.53 – 6.48 (m, 2H), 4.72 (dd, *J* = 13.7, 3.5 Hz, 1H), 4.09 (d, *J* = 9.4 Hz, 1H), 3.84 (ddd, *J* = 11.6, 9.5, 3.5 Hz, 1H), 3.74 (dd, *J* = 13.7, 11.6 Hz, 1H), 2.47 (s, 3H). **^{13}C NMR** (101 MHz, Chloroform-*d*) δ 144.6, 140.8, 136.3, 134.4, 133.5, 131.5, 130.2, 130.0, 128.7, 128.6, 127.7, 127.4, 126.2, 54.2, 52.5, 48.3, 21.6. **MS (ESI)** m/z (%): 294.1 (100), 396.1 (50), 398.1 (40), 476.0 ($[M+H]^+$, 40), 478.0 ($[M+H]^+$, 40, for ^{81}Br).

7-chloro-8-phenyl-5-tosyl-5,6,7,8-tetrahydro-[1,3]dioxolo[4,5-g]quinoline (17)

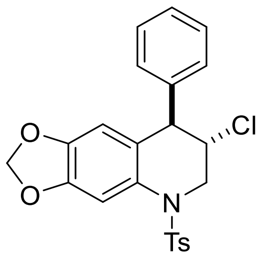

17

The reaction of **S7** (81.5 mg, 0.20 mmol), DCDMH (47.3 mg, 0.24 mmol) and 4-NH_2-TEMPO (1.7 mg, 0.01 mmol) at 25°C for 1 hour afforded 57.4 mg (65%) of **17** as a white solid (petroleum ether/ethyl acetate = 10:1). **^{1}H NMR** (400 MHz, Chloroform-*d*) δ 7.65 – 7.59 (m, 2H), 7.41 (d, *J* = 1.4 Hz, 1H), 7.31 (d, *J* = 7.9 Hz, 2H), 7.21 – 7.17 (m, 1H), 7.13 (t, *J* = 7.5 Hz, 2H), 6.49 (d, *J* = 7.4 Hz, 2H), 6.05 (s, 1H), 5.92 (d, *J* = 10.2 Hz, 2H), 4.65 (dd, *J* = 14.0, 3.9 Hz, 1H), 3.84 (d, *J* = 9.6 Hz, 1H), 3.78 – 3.67 (m, 1H), 3.57 (dd, *J* = 14.1, 11.4 Hz, 1H), 2.46 (s, 3H). **^{13}C NMR** (101 MHz, Chloroform-

d) δ 146.7, 146.1, 144.3, 141.3, 136.3, 130.1, 129.5, 128.7, 128.4, 127.6, 127.4, 125.6, 108.8, 106.0, 101.6, 57.1, 54.2, 52.5, 21.6. **HRMS (ESI)** exact mass calc'd for $C_{23}H_{21}ClNO_4S$ ($[M+H]^+$): 442.0880; found *m/z*: 442.0874, 444.0847 (For ^{37}Cl).

7-bromo-8-phenyl-5-tosyl-5,6,7,8-tetrahydro-[1,3]dioxolo[4,5-g]quinoline (18)[1]

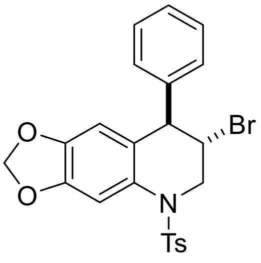

18

The reaction of **S7** (81.5 mg, 0.20 mmol), DBDMH (68.6 mg, 0.24 mmol) and 4-Oxo-TEMPO (3.4 mg, 0.02 mmol) at 25°C for 4 h afforded 85.6 mg (88%) of **18** as a white solid (petroleum ether/ethyl acetate = 10:1). **^{1}H NMR** (400 MHz, Chloroform-*d*) δ 7.66 – 7.60 (m, 2H), 7.40 (s, 1H), 7.33 (d, *J* = 8.1 Hz, 2H), 7.23 – 7.17 (m, 1H), 7.16 – 7.07 (m, 2H), 6.52 – 6.42 (m, 2H), 6.03 (d, *J* = 1.0 Hz, 1H), 5.92 (dd, *J* = 9.8, 1.4 Hz, 2H), 4.75 (dd, *J* = 13.8, 3.7 Hz, 1H), 4.01 (d, *J* = 9.8 Hz, 1H), 3.80 (ddd, *J* = 12.0, 9.8, 3.7 Hz, 1H), 3.70 (dd, *J* = 13.8, 12.1 Hz, 1H), 2.47 (s, 3H). **^{13}C NMR** (101 MHz, Chloroform-*d*) δ 146.7, 146.0, 144.3, 141.5, 136.3, 130.1, 129.4, 128.6, 128.3, 127.5, 127.4, 125.7, 108.7, 106.1, 101.6, 54.4, 53.0, 49.1, 21.5. **MS (ESI)** m/z (%): 250.1 (75), 406.1 (100), 486.1 ($[M+H]^+$, 15), 488.1 ($[M+H]^+$, 15, for ^{81}Br).

7-iodo-8-phenyl-5-tosyl-5,6,7,8-tetrahydro-[1,3]dioxolo[4,5-g]quinoline (19)

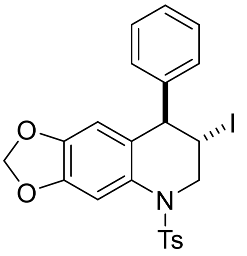

19

The reaction of **S7** (81.5 mg, 0.20 mmol), DIDMH (118.3 mg, 0.24 mmol) and 4-Oxo-TEMPO (3.4 mg, 0.02 mmol) at 25°C for 12 h afforded 96.0 mg (90%) of **19** as a white solid (petroleum ether/ethyl acetate = 8:1). **^{1}H NMR** (400 MHz, Chloroform-*d*)

δ 7.67 – 7.56 (m, 2H), 7.37 (s, 1H), 7.33 (d, *J* = 8.0 Hz, 2H), 7.23 – 7.17 (m, 1H), 7.12 (dd, *J* = 8.2, 6.8 Hz, 2H), 6.48 – 6.39 (m, 2H), 6.01 (d, *J* = 0.9 Hz, 1H), 5.91 (dd, *J* = 8.5, 1.4 Hz, 2H), 4.83 (dd, *J* = 13.4, 3.0 Hz, 1H), 4.12 (d, *J* = 9.9 Hz, 1H), 3.90 (ddd, *J* = 12.9, 9.9, 3.0 Hz, 1H), 3.86 – 3.78 (m, 1H), 2.48 (s, 3H). **^{13}C NMR** (101 MHz, Chloroform-*d*) δ 146.6, 146.0, 144.4, 142.1, 136.6, 130.2, 129.5, 128.5, 128.4, 127.5, 127.4, 125.6, 108.8, 106.3, 101.6, 55.6, 55.2, 28.7, 21.6. **HRMS (ESI)** exact mass calc'd for $C_{23}H_{21}INO_4S$ ([M+H]$^+$): 534.0236; found *m/z*: 534.0234.

3-chloro-4-(4-chlorophenyl)-1-tosyl-1,2,3,4-tetrahydroquinoline (20)

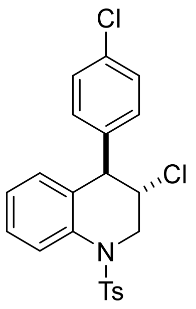

20

The reaction of **S8** (79.6 mg, 0.20 mmol), DCDMH (47.3 mg, 0.24 mmol) and 4-NH_2-TEMPO (3.4 mg, 0.02 mmol) at 25°C for 8 h afforded 45.0 mg (52%) of **20** as a white solid (petroleum ether/ethyl acetate = 10:1). **^{1}H NMR** (400 MHz, Chloroform-*d*) δ 7.89 (dd, *J* = 8.4, 1.2 Hz, 1H), 7.65 – 7.56 (m, 2H), 7.32 – 7.21 (m, 3H), 7.16 – 7.09 (m, 2H), 7.04 (td, *J* = 7.5, 1.3 Hz, 1H), 6.65 (dt, *J* = 7.8, 1.3 Hz, 1H), 6.53 – 6.44 (m, 2H), 4.66 (dd, *J* = 13.9, 3.8 Hz, 1H), 3.98 (d, *J* = 9.5 Hz, 1H), 3.76 (ddd, *J* = 11.4, 9.5, 3.8 Hz, 1H), 3.62 (dd, *J* = 13.9, 11.4 Hz, 1H), 2.45 (s, 3H). **^{13}C NMR** (101 MHz, Chloroform-*d*) δ 144.4, 140.0, 136.4, 135.7, 133.2, 131.1, 130.3, 130.2, 130.0, 128.6, 127.6, 127.4, 126.1, 124.8, 57.0, 53.5, 52.0, 21.6. **HRMS (ESI)** exact mass calc'd for $C_{22}H_{20}Cl_2NO_2S$ ([M+H]$^+$): 432.0592; found *m/z*: 432.0591, 434.0564 (For ^{37}Cl).

3-bromo-4-(4-chlorophenyl)-1-tosyl-1,2,3,4-tetrahydroquinoline (21)[1]

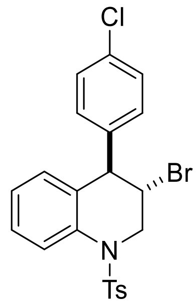

21

The reaction of **S8** (79.6 mg, 0.20 mmol), DBDMH (68.6 mg, 0.24 mmol) and 4-Oxo-TEMPO (1.7 mg, 0.01 mmol) at 25°C for 4 h afforded 66.8 mg (70%) of **21** as a white solid (petroleum ether/ethyl acetate = 10:1). **^{1}H NMR** (400 MHz, Chloroform-*d*) δ 7.87 (d, *J* = 8.2 Hz, 1H), 7.60 (d, *J* = 8.2 Hz, 2H), 7.29 (d, *J* = 8.0 Hz, 2H), 7.26 – 7.22 (m, 1H), 7.16 – 7.08 (m, 2H), 7.03 (t, *J* = 7.5 Hz, 1H), 6.62 (d, *J* = 7.9 Hz, 1H), 6.47 (d, *J* = 8.4 Hz, 2H), 4.76 (dd, *J* = 13.7, 3.6 Hz, 1H), 4.15 (d, *J* = 9.6 Hz, 1H), 3.84 (ddd, *J* = 13.0, 9.5, 3.5 Hz, 1H), 3.74 (dd, *J* = 13.8, 11.8 Hz, 1H), 2.46 (s, 3H). **^{13}C NMR** (101 MHz, Chloroform-*d*) δ 144.4, 140.3, 136.6, 135.7, 133.2, 131.4, 130.2, 130.1, 128.6, 127.6, 127.4, 126.1, 125.0, 53.8, 52.7, 48.9, 21.6. **MS (ESI)** m/z (%): 220.1 (100), 396.1 (80), 398.1 (80), 476.0 ([M+H]$^+$, 30), 478.0 ([M+H]$^+$, 30, for ^{81}Br).

3-bromo-6-methoxy-4-phenylchromane (22)[1]

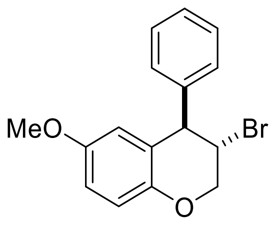

22

The reaction of **S9** (48.1 mg, 0.20 mmol), DBDMH (68.6 mg, 0.24 mmol) and 4-Oxo-TEMPO (3.4 mg, 0.02 mmol) at 25°C for 1 hour afforded 61.3 mg (96%) of **22** as a white solid (petroleum ether/ethyl acetate = 20:1). **^{1}H NMR** (400 MHz, Chloroform-*d*) δ 7.37 – 7.27 (m, 3H), 7.21 – 7.12 (m, 2H), 6.86 (d, *J* = 8.9 Hz, 1H), 6.76 (dd, *J* = 9.0, 3.0 Hz, 1H), 6.30 (d, *J* = 3.0 Hz, 1H), 4.45 – 4.34 (m, 3H), 4.17 (ddd, *J* = 13.0, 7.5, 2.5 Hz, 1H), 3.62 (s, 3H). **^{13}C NMR** (101 MHz, Chloroform-*d*) δ 154.1, 147.8, 142.5,

129.1, 128.8, 127.5, 123.0, 117.5, 114.9, 114.8, 67.9, 55.6, 51.8, 49.9. **MS (EI)** m/z (%): 91.0 (100), 115.0 (100), 318.0 (M^+, 90), 320.0 (90, for ^{81}Br).

3-iodo-6-methoxy-4-phenylchromane (23)

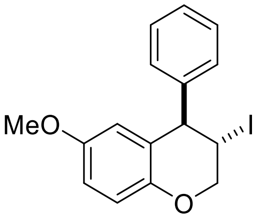

23

The reaction of **S9** (48.1 mg, 0.20 mmol), DIDMH (118.3 mg, 0.24 mmol) and 4-Oxo-TEMPO (3.4 mg, 0.02 mmol) at 25°C for 4 h afforded 58.6 mg (80%) of **23** as a white solid (petroleum ether/ethyl acetate = 20:1). **^{1}H NMR** (400 MHz, Chloroform-*d*) δ 7.38 – 7.27 (m, 3H), 7.19 – 7.11 (m, 2H), 6.84 (d, *J* = 8.9 Hz, 1H), 6.75 (dd, *J* = 8.9, 3.0 Hz, 1H), 6.25 (d, *J* = 2.9 Hz, 1H), 4.53 (td, *J* = 7.7, 3.0 Hz, 1H), 4.46 (d, *J* = 7.4 Hz, 1H), 4.36 (dd, *J* = 11.5, 3.0 Hz, 1H), 4.23 (dd, *J* = 11.5, 8.1 Hz, 1H), 3.61 (s, 3H). **^{13}C NMR** (101 MHz, Chloroform-*d*) δ 153.9, 147.7, 143.1, 128.9, 128.7, 127.5, 123.3, 117.5, 114.9, 114.7, 70.0, 55.6, 53.1, 29.1. **HRMS (EI)** exact mass calc'd for $C_{16}H_{15}O_2I$ ($[M]^+$):366.0111 ; found *m/z*: 366.0114.

3-bromo-6-(tert-butyl)-4-phenylchromane (24)

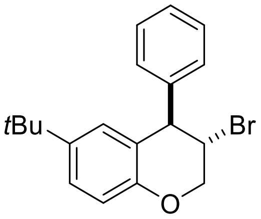

24

The reaction of **S10** (53.3 mg, 0.20 mmol), DBDMH (68.6 mg, 0.24 mmol) and 4-Oxo-TEMPO (3.4 mg, 0.02 mmol) at 25°C for 4 h afforded 54.6 mg (79%) of **26** as a white solid (petroleum ether/ethyl acetate = 40:1). **^{1}H NMR** (400 MHz, Chloroform-*d*) δ 7.35 – 7.28 (m, 3H), 7.21 (dd, *J* = 8.6, 2.5 Hz, 1H), 7.18 – 7.14 (m, 2H), 6.85 (d, *J* = 8.6 Hz, 1H), 6.76 (d, *J* = 2.4 Hz, 1H), 4.45 – 4.38 (m, 3H), 4.20 (ddd, *J* = 11.7, 6.2, 2.1 Hz, 1H), 1.16 (s, 9H). **^{13}C NMR** (101 MHz, Chloroform-*d*) δ 151.3, 144.1, 142.8, 129.1,

128.6, 127.37, 127.36, 125.5, 121.5, 116.0, 67.9, 51.6, 50.1, 34.1, 31.3. **HRMS (EI)** exact mass calc'd for $C_{19}H_{21}OBr$ ([M]$^+$): 344.0770; found *m/z*: 344.0774, 346.0754 (for ^{81}Br).

6-(tert-butyl)-3-iodo-4-phenylchromane (25)

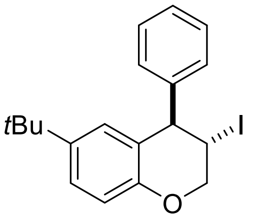

25

The reaction of **S10** (53.3 mg, 0.20 mmol), DIDMH (118.3 mg, 0.24 mmol) and 4-Oxo-TEMPO (3.4 mg, 0.02 mmol) at 25°C for 24 h afforded 57.3 mg (73%) of **25** as a white solid (petroleum ether/ethyl acetate = 20:1). **^{1}H NMR** (400 MHz, Chloroform-*d*) δ 7.36 – 7.27 (m, 3H), 7.20 (dd, *J* = 8.6, 2.5 Hz, 1H), 7.17 – 7.13 (m, 2H), 6.83 (d, *J* = 8.7 Hz, 1H), 6.70 (d, *J* = 2.4 Hz, 1H), 4.54 (td, *J* = 7.8, 3.0 Hz, 1H), 4.49 (d, *J* = 7.5 Hz, 1H), 4.38 (dd, *J* = 11.5, 3.0 Hz, 1H), 4.26 (dd, *J* = 11.5, 8.1 Hz, 1H), 1.14 (s, 9H). **^{13}C NMR** (101 MHz, Chloroform-*d*) δ 151.3, 144.0, 143.4, 128.9, 128.6, 127.34, 127.31, 125.4, 122.0, 116.1, 70.1, 53.0, 34.0, 31.3, 29.5. **HRMS (EI)** exact mass calc'd for $C_{19}H_{21}OI$ ([M]$^+$): 392.0632; found *m/z*: 392.0631.

6-(benzyloxy)-3-bromo-4-phenylchromane (26)

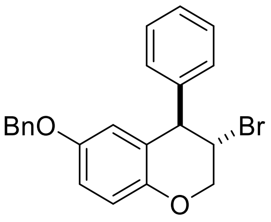

26

The reaction of **S11** (63.3 mg, 0.20 mmol), DBDMH (68.6 mg, 0.24 mmol) and 4-Oxo-TEMPO (3.4 mg, 0.02 mmol) at 25°C for 8 h afforded 60.1 mg (76%) of **26** as a white solid (petroleum ether/ethyl acetate = 20:1). **^{1}H NMR** (400 MHz, Chloroform-*d*) δ 7.36 – 7.26 (m, 8H), 7.17 – 7.12 (m, 2H), 6.89 – 6.78 (m, 2H), 6.38 (d, *J* = 2.7 Hz, 1H), 4.90 – 4.80 (m, 2H), 4.45 – 4.35 (m, 3H), 4.22 – 4.12 (m, 1H). **^{13}C NMR** (101

MHz, Chloroform-*d*) δ 153.2, 147.9, 142.3, 136.9, 129.0, 128.7, 128.5, 127.9, 127.53, 127.49, 123.1, 117.4, 116.1, 115.7, 70.5, 68.1, 51.8, 49.9. **HRMS (EI)** exact mass calc'd for $C_{22}H_{19}O_2Br$ ([M]$^+$): 394.0563; found *m/z*: 394.0566, 396.0546 (for ^{81}Br).

6-(benzyloxy)-3-iodo-4-phenylchromane (27)

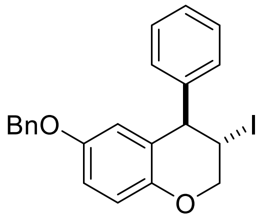

27

The reaction of **S11** (63.3 mg, 0.20 mmol), DIDMH (118.3 mg, 0.24 mmol) and 4-Oxo-TEMPO (3.4 mg, 0.02 mmol) at 25°C for 12 h afforded 69.0 mg (78%) of **27** as a white solid (petroleum ether/ethyl acetate = 20:1). **^{1}H NMR** (400 MHz, Chloroform-*d*) δ 7.38 – 7.24 (m, 8H), 7.13 (dd, *J* = 7.5, 2.0 Hz, 2H), 6.82 (d, *J* = 2.2 Hz, 2H), 6.33 (d, *J* = 2.2 Hz, 1H), 4.82 (d, *J* = 3.6 Hz, 2H), 4.51 (td, *J* = 8.0, 3.1 Hz, 1H), 4.44 (d, *J* = 7.7 Hz, 1H), 4.37 (dd, *J* = 11.4, 3.2 Hz, 1H), 4.23 (dd, *J* = 11.5, 8.3 Hz, 1H). **^{13}C NMR** (101 MHz, Chloroform-*d*) δ 153.1, 147.9, 143.0, 136.9, 128.9, 128.7, 128.5, 127.8, 127.52, 127.45, 123.5, 117.5, 116.1, 115.6, 70.5, 70.2, 53.1, 29.2. **HRMS (EI)** exact mass calc'd for $C_{22}H_{19}O_2I$ ([M]$^+$): 442.0424; found *m/z*: 442.0427.

2-bromo-1-phenyl-1,2,3,4-tetrahydronaphthalene (28)

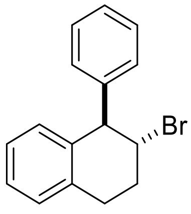

28

The reaction of **S12** (41.7 mg, 0.20 mmol), DBDMH (68.6 mg, 0.24 mmol) and 4-Oxo-TEMPO (3.4 mg, 0.02 mmol) at 25°C for 12 hour afforded 52.8 mg (92%) of **28** as a colorless oil (petroleum ether/ethyl acetate = 100:1). **^{1}H NMR** (400 MHz, Chloroform-*d*) δ 7.38 – 7.28 (m, 3H), 7.25 – 7.19 (m, 2H), 7.17 – 7.07 (m, 3H), 6.88 (dd, *J* = 7.7, 1.1 Hz, 1H), 4.62 (ddd, *J* = 7.6, 5.9, 2.8 Hz, 1H), 4.55 (d, *J* = 5.8 Hz, 1H),

3.18 (ddd, *J* = 17.1, 7.8, 5.7 Hz, 1H), 3.04 (dt, *J* = 17.1, 6.2 Hz, 1H), 2.46 – 2.36 (m, 1H), 2.26 – 2.17 (m, 1H). **^{13}C NMR** (101 MHz, Chloroform-*d*) δ 144.4, 136.1, 135.2, 130.6, 129.2, 128.6, 128.4, 126.9, 126.6, 126.3, 56.0, 54.7, 29.5, 27.5. **HRMS (EI)** exact mass calc'd for $C_{16}H_{15}Br$ ($[M]^+$): 286.0352; found *m/z*: 286.0352, 288.0322 (for ^{81}Br).

2-iodo-1-phenyl-1,2,3,4-tetrahydronaphthalene (29)

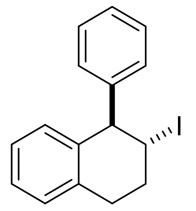

29

The reaction of **S12** (41.7 mg, 0.20 mmol), DIDMH (118.3 mg, 0.24 mmol) and 4-Oxo-TEMPO (3.4 mg, 0.02 mmol) at 25°C for 2 h afforded 42.1 mg (63%) of **29** as a colorless oil (petroleum ether/ethyl acetate = 100:1). **^{1}H NMR** (400 MHz, Chloroform-*d*) 7.35 – 7.27 (m, 3H), 7.22 – 7.17 (m, 2H), 7.12 – 7.04 (m, 3H), 6.84 (d, *J* = 7.8 Hz, 1H), 4.71 (ddd, *J* = 7.3, 5.8, 3.2 Hz, 1H), 4.62 (d, *J* = 5.9 Hz, 1H), 3.10 – 3.01 (m, 2H), 2.33 – 2.17 (m, 2H). **^{13}C NMR** (101 MHz, Chloroform-*d*) δ 145.3, 136.2, 135.1, 130.6, 129.1, 128.8, 128.5, 126.9, 126.6, 126.3, 56.1, 35.7, 31.2, 29.3. **HRMS (EI)** exact mass calc'd for $C_{16}H_{15}I$ ($[M]^+$): 334.0213; found *m/z*: 334.0217.

(C) TEMPO-catalysed dibrominaion of alkenes and alkynes

(a) Optimization of the Reaction Conditions

Supplementary Table 4. Optimization of dibromination reaction.

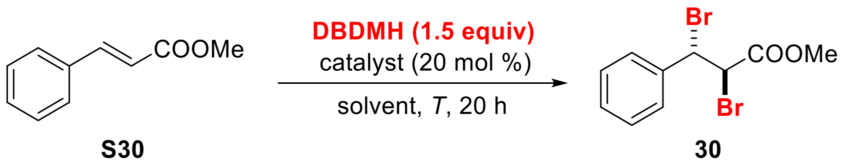

Entry	Catalyst	Solvent	*T* (°C)	Yield[b]
1	-	DCM	40 °C	Trace
2	DMSO	DCM	40 °C	Trace
3	TEMPO	DCM	40 °C	67 % + 15 % S.M.
4	TEMPO	DCE	40 °C	70 % + 30 % S.M.
5	TEMPO	CCl_4	40 °C	42 % + 44 % S.M.
6	TEMPO	DMSO	40 °C	Trace
7	TEMPO	EA	40 °C	53 % + 30 % S.M.
8	TEMPO	DMF	40 °C	52 % + 3 % S.M.
9	TEMPO	Acetone	40 °C	56 % + 25 % S.M.
10	TEMPO	DCE	60 °C	75 % + 23 % S.M.
11	4-MeO-TEMPO	DCE	60 °C	63 % + 24 % S.M.
12	4-Oxo-TEMPO	DCE	60 °C	52 % + 33 % S.M.
13	TEMPO (40 mol%)	DCE	60 °C	97 % + 3 % S.M.
**14[c]**	**TEMPO**	**DCE**	**60 °C**	**98 % (94 %[d])**

[a] Reactions were carried out with **S30** (0.50 mmol), catalyst (0.10 mmol) and DBDMH (0.75 mmol) for 20 h under air. [b] Yields were determined by H^1-NMR using 1,1,2,2-tetrachloroethane as internal standard. [c] Catalyst was added twice (0 h and 10 h, 10 mol% each time). [d] Isolated yield.

(b) Experimental Procedures

Typical procedure:

Substrate **S30-S47** (0.50 mmol), DBDMH (0.75 mmol), TEMPO (0.10 mmol) and DCE (2.0 mL) was added to a reaction tube with a magnetic bar. The mixture was stirred at at the indicated temperature for the specified reaction time. Upon completion of the reaction (monitored by TLC), the reaction mixture was quenched with saturated Na_2SO_3 aqueous solution (2 mL). The aqueous phase was diluted with water (3 mL) and extracted with EtOAc (5 mL × 3). The combined organic extracts were dried over anhydrous Na_2SO_4, filtered, and concentrated under reduced pressure. The residue was purified over silica gel chromatography to afford **30-47**.

2,3-dibromo-1,3-diphenylpropan-1-one (30)[3]

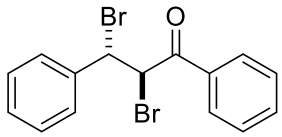

30

The reaction of **S30** (104.2 mg, 0.50 mmol), DBDMH (214 mg, 0.75 mmol) and TEMPO (15.6 mg, 0.10 mmol) in DCE (2.0 mL) at 60 °C for 0.5 h, affords 166.9 mg (91 %) of **30** as a white solid (petroleum ether/ethyl acetate = 10:1). **^{1}H NMR** (400 MHz, $CDCl_3$) δ 8.13 – 8.08 (m, 2H), 7.65 (t, *J* = 7.4 Hz, 1H), 7.58 – 7.50 (m, 4H), 7.46 – 7.35 (m, 3H), 5.84 (d, *J* = 10.6 Hz, 1H), 5.76 (d, *J* = 10.5 Hz, 1H). **^{13}C NMR** (101 MHz, $CDCl_3$) δ 191.1, 138.2, 134.4, 134.1, 129.3, 129.0, 128.9, 128.8, 128.3, 49.8, 46.8. **MS (EI)** m/z (%): 77.0 (100), 207.0 (70), 285.9 ($[M-Br]^+$, 10), 287.9 (10, for ^{81}Br).

2,3-dibromo-3-(4-nitrophenyl)-1-phenylpropan-1-one (31)[4]

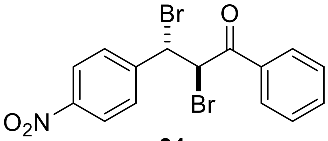

31

The reaction of **S31** (126.7 mg, 0.50 mmol), DBDMH (214 mg, 0.75 mmol) and TEMPO (15.6 mg, 0.10 mmol) in DCE (2.0 mL) at 60 °C for 0.5 h, affords 184.1 mg (89 %) of **31** as a white solid (petroleum ether/ethyl acetate = 5:1). **^{1}H NMR** (400 MHz, $CDCl_3$) δ 8.33 – 8.27 (m, 2H), 8.14 – 8.08 (m, 2H), 7.75 – 7.67 (m, 3H), 7.57 (t, *J* = 7.8 Hz, 2H), 5.79 (d, *J* = 11.2 Hz, 1H), 5.70 (d, *J* = 11.3 Hz, 1H). **^{13}C NMR** (101 MHz, $CDCl_3$) δ 190.4, 148.1, 145.1, 134.5, 134.0, 129.4, 129.1, 128.9, 124.1, 47.2, 45.8. **HRMS (ESI)** exact mass calc'd for $C_{15}H_{12}Br_2NO_3$ ($[M+H]^+$): 411.9184; found *m/z*: 411.9182, 413.9159 (for ^{81}Br).

3,4-dibromo-4-phenylbutan-2-one (32)[3]

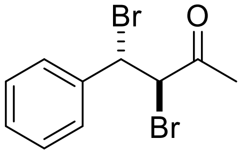

32

The reaction of **S32** (73.1 mg, 0.50 mool), DBDMH (214 mg, 0.75 mmol) and TEMPO (15.6 mg, 0.10 mool) in DCE (2 mL) at 60 °C for 20 h, affords 138.3 mg (94 %) of **32** as a white solid (petroleum ether/ethyl acetate = 10:1). **^{1}H NMR** (400 MHz, $CDCl_3$) δ 7.44 – 7.34 (m, 5H), 5.32 (d, *J* = 11.6 Hz, 1H), 4.94 (d, *J* = 11.7 Hz, 1H), 2.47 (s, 3H). **^{13}C NMR** (101 MHz, $CDCl_3$) δ 198.3, 137.7, 129.3, 128.8, 128.1, 52.8, 49.5, 26.9. **MS (EI)** m/z (%): 102.1 (100), 145.0 (90), 223.0 ($[M-Br]^+$, 20), 225.0 (20, for ^{81}Br).

methyl 2,3-dibromo-3-phenylpropanoate (33)[3]

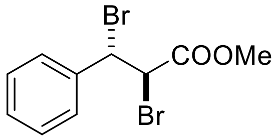

33

The reaction of **S33** (81.1 mg, 0.50 mmol), DBDMH (214 mg, 0.75 mmol) and TEMPO (15.6 mg, 0.10 mmol) in DCE (2.0 mL) at 60 °C for 20 h, affords 151.0 mg (94 %) of **33** as a white solid (petroleum ether/ethyl acetate = 10:1). **^{1}H NMR** (400 MHz, $CDCl_3$) δ 7.43 – 7.33 (m, 5H), 5.34 (d, *J* = 11.7 Hz, 1H), 4.85 (d, *J* = 11.8 Hz, 1H), 3.90 (s, 3H). **^{13}C NMR** (101 MHz, $CDCl_3$) δ 168.4, 137.5, 129.4, 128.9, 128.1, 53.5, 50.6, 46.7. **MS (EI)** m/z (%): 102.0 (90), 161.0 (100), 239.9 ($[M-Br]^+$, 20), 241.9 (20, for ^{81}Br).

methyl 2,3-dibromo-3-(4-chlorophenyl)propanoate (34)[5]

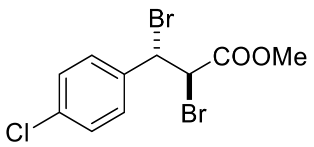

34

The reaction of **S34** (98.3 mg, 0.50 mool), DBDMH (214 mg, 0.75 mmol) and TEMPO (15.6 mg, 0.10 mool) in DCE (2 mL) at 60 °C for 20 h, affords 143.9 mg (81 %) of **34** as a white solid (petroleum ether/ethyl acetate = 10:1). **^{1}H NMR** (400 MHz, $CDCl_3$) δ 7.39 – 7.31 (m, 4H), 5.32 (d, *J* = 11.7 Hz, 1H), 4.79 (d, *J* = 11.7 Hz, 1H), 3.89 (s, 3H). **^{13}C NMR** (100 MHz, $CDCl_3$) δ 168.0, 136.1, 135.2, 129.3, 129.1, 53.5, 49.5,

46.4. **HRMS (ESI)** exact mass calc'd for $C_{10}H_{10}Br_2ClO_2$ ($[M+H]^+$): 354.8736; found *m/z*: 354.8736, 356.8714 (for ^{81}Br).

ethyl 2,3-dibromo-3-(4-bromophenyl)propanoate (35)

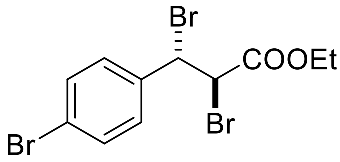

35

The reaction of **S35** (127.6 mg, 0.50 mmol), DBDMH (214 mg, 0.75 mmol) and TEMPO (15.6 mg, 0.10 mmol) in DCE (2.0 mL) at 60 °C for 0.5 h, affords 182.7 mg (88 %) of **35** as a white solid (petroleum ether/ethyl acetate = 10:1). **^{1}H NMR** (400 MHz, $CDCl_3$) δ 7.56 – 7.49 (m, 2H), 7.30 – 7.26 (m, 2H), 5.30 (d, *J* = 11.7 Hz, 1H), 4.76 (d, *J* = 11.7 Hz, 1H), 4.35 (q, *J* = 7.1 Hz, 2H), 1.37 (t, *J* = 7.1 Hz, 3H). **^{13}C NMR** (101 MHz, $CDCl_3$) δ 167.6, 136.8, 132.1, 129.7, 123.4, 62.8, 49.7, 46.8, 13.9. **HRMS (ESI)** exact mass calc'd for $C_{11}H_{12}Br_3O_2$ ($[M+H]^+$): 412.8387; found *m/z*: 412.8385, 414.3815 (for ^{81}Br).

ethyl 2,3-dibromo-3-(3-nitrophenyl)propanoate (36)

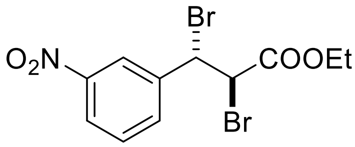

36

The reaction of **S36** (105.6 mg, 0.50 mmol), DBDMH (214 mg, 0.75 mmol) and TEMPO (15.6 mg, 0.10 mmol) in DCE (2.0 mL) at 60 °C for 20 h, affords 131.6 mg (69 %) of **36** as a white solid (petroleum ether/ethyl acetate = 10:1). **^{1}H NMR** (400 MHz, $CDCl_3$) δ 8.30 (t, *J* = 2.0 Hz, 1H), 8.26 – 8.22 (m, 1H), 7.78 – 7.73 (m, 1H), 7.61 (t, *J* = 8.0 Hz, 1H), 5.42 (d, *J* = 11.7 Hz, 1H), 4.82 (d, *J* = 11.8 Hz, 1H), 4.42 – 4.35 (m, 2H), 1.39 (t, *J* = 7.2 Hz, 3H). **^{13}C NMR** (101 MHz, $CDCl_3$) δ 167.2, 148.3, 139.8, 134.0, 130.0, 124.1, 123.1, 62.9, 48.3, 46.3, 13.8. **HRMS (ESI)** exact mass calc'd for $C_{11}H_{12}Br_2NO_4$ ($[M+H]^+$): 379.9133; found *m/z*: 379.9132, 381.9112 (for ^{81}Br).

2,3-dibromocyclohexan-1-one (37)[6]

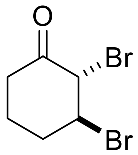

37

The reaction of **S37** (48.1 mg, 0.50 mmol), DBDMH (214 mg, 0.75 mmol) and TEMPO (15.6 mg, 0.10 mmol) in DCE (2.0 mL) at 60 °C for 20 h, affords 83.3 mg (65 %) of **37** as a white solid (petroleum ether/ethyl acetate = 5:1). **^{1}H NMR** (400 MHz, $CDCl_3$) δ 4.75 – 4.71 (m, 1H), 4.52 – 4.49 (m, 1H), 3.13 – 3.03 (m, 1H), 2.72 – 2.63 (m, 1H), 2.43 – 2.27 (m, 2H), 2.12 – 1.97 (m, 2H). **^{13}C NMR** (101 MHz, $CDCl_3$) δ 200.6, 53.1, 50.5, 35.1, 27.2, 21.5. **MS (EI)** m/z (%): 67.0 (100), 145.9 (70), 147.9 (70), 173.9 ($[M-Br]^+$, 60), 175.9 (60, for ^{81}Br).

methyl 1,2-dibromocyclopentane-1-carboxylate (38)

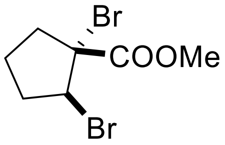

38

The reaction of **S38** (63.1 mg, 0.50 mmol), DBDMH (214 mg, 0.75 mmol) and TEMPO (15.6 mg, 0.10 mmol) in DCE (2.0 mL) at 60 °C for 20 h, affords 121.5 mg (85 %) of **38** as a colorless oil (petroleum ether/ethyl acetate = 10:1). **^{1}H NMR** (400 MHz, $CDCl_3$) δ 4.72 (d, *J* = 5.0 Hz, 1H), 3.83 (s, 3H), 3.01 – 2.83 (m, 2H), 2.44 – 2.28 (m, 2H), 2.18 – 2.00 (m, 2H). **^{13}C NMR** (101 MHz, $CDCl_3$) δ 168.6, 68.5, 57.8, 53.0, 35.2, 34.2, 19.7. **HRMS (ESI)** exact mass calc'd for $C_7H_{11}Br_2NO_2$ ($[M+H]^+$): 284.9126; found *m/z*: 284.9126, 286.9106 (for ^{81}Br).

methyl 1,2-dibromocyclohexane-1-carboxylate (39)

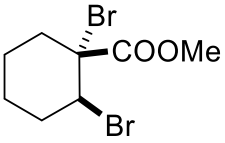

39

The reaction of **S39** (70.1 mg, 0.50 mmol), DBDMH (214 mg, 0.75 mmol) and TEMPO (15.6 mg, 0.10 mmol) in DCE (2.0 mL) at 60 °C for 20 h, affords 142.5 mg (95 %) of **39** as a colorless oil (petroleum ether/ethyl acetate = 100:1). **^{1}H NMR** (400 MHz, $CDCl_3$) δ 4.89 (dd, *J* = 2.7, 2.5 Hz, 1H), 3.83 (s, 3H), 2.55 – 2.43 (m, 1H), 2.43 – 2.30 (m, 1H), 2.16 (ddd, *J* = 15.3, 3.3, 1.7 Hz, 1H), 2.08 – 1.99 (m, 1H), 1.86 – 1.72 (m, 3H), 1.67 – 1.55 (m, 1H). **^{13}C NMR** (101 MHz, $CDCl_3$) δ 169.5, 63.5, 54.4, 53.0, 30.1, 29.5, 21.1, 19.4. **HRMS (ESI)** exact mass calc'd for $C_8H_{13}Br_2O_2$ ($[M+H]^+$): 298.9273; found *m/z*: 298.9271, 300.9251 (for ^{81}Br).

1,2-dibromopropylbenzene (40)[7]

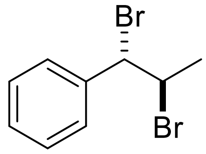

40

The reaction of **S40** (59.1 mg, 0.50 mmol), DBDMH (214 mg, 0.75 mmol) and TEMPO (15.6 mg, 0.10 mmol) in DCE (2.0 mL) at 25 °C for 1 h, affords 71.1 mg (51 %) of **40** as a white solid (petroleum ether/ethyl acetate = 100:1). **^{1}H NMR** (400 MHz, $CDCl_3$) δ 7.45 – 7.33 (m, 5H), 5.07 (d, *J* = 10.2 Hz, 1H), 4.68– 4.59(m, 1H), 2.08 (d, *J* = 6.5 Hz, 3H). **^{13}C NMR** (101 MHz, $CDCl_3$) δ 140.5, 128.8, 128.6, 127.7, 59.1, 51.1, 25.8. **MS (EI)** m/z (%): 117.0 (100), 91.0 (50), 196.9 ($[M-Br]^+$, 20), 198.9 (20, for ^{81}Br), 275.6 (M^+, 5).

1,2,3-(tribromopropyl)benzene (41)[8]

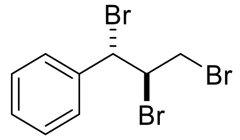

41

The reaction of **S41** (98.5 mg, 0.50 mmol), DBDMH (214 mg, 0.75 mmol) and TEMPO (15.6 mg, 0.10 mmol) in DCE (2.0 mL) at 25 °C for 20 h, affords 135.6 mg (76 %) of **41** as a white solid (petroleum ether/ethyl acetate = 100:1). **^{1}H NMR** (400 MHz, $CDCl_3$) δ 7.48 – 7.33 (m, 5H), 5.31 (dd, *J* = 9.5, 1.3 Hz, 1H), 4.74 (dtd, *J* = 9.6,

4.2, 1.3 Hz, 1H), 4.26 (ddd, J = 11.3, 4.2, 1.3 Hz, 1H), 3.94 (ddd, J = 11.5, 4.1, 1.3 Hz, 1H). **^{13}C NMR** (101 MHz, $CDCl_3$) δ 138.6, 129.2, 128.7, 128.2, 54.5, 54.1, 38.1. **MS (EI)** m/z (%): 117.0 (100), 274.8 ([M-Br]$^+$, 10), 276.8 (20, for ^{81}Br), 278.7 (10, for ^{81}Br), 355.5 (M$^+$, 5).

1,2-dibromodecane (42)[7]

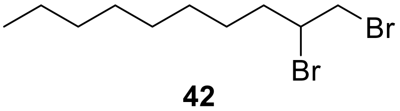

42

The reaction of **S42** (70.1 mg, 0.50 mmol), DBDMH (214 mg, 0.75 mmol) and TEMPO (15.6 mg, 0.10 mmol) in DCE (2.0 mL) at 25 °C for 1 h, affords 121.7 mg (81 %) of **42** as a white solid (petroleum ether/ethyl acetate = 100:1). **^{1}H NMR** (400 MHz, $CDCl_3$) δ 4.20 – 4.13 (m, 1H), 3.85 (dd, J = 10.2, 4.4 Hz, 1H), 3.63 (t, J = 10.0 Hz, 1H), 2.18 – 2.09 (m, 1H), 1.83 – 1.73 (m, 1H), 1.63 – 1.51 (m, 1H), 1.47 – 1.22 (m, 11H), 0.89 (t, J = 6.7 Hz, 3H). **^{13}C NMR** (101 MHz, $CDCl_3$) δ 53.1, 36.3, 36.0, 31.8, 29.3, 29.2, 28.8, 26.7, 22.6, 14.1. **MS (EI)** m/z (%): 55.1 (100), 83.1 (75), 219.0 ([M-Br]$^+$, 10), 221.0 (10, for ^{81}Br).

(*E*)-1,2-dibromooct-1-ene (43)[7]

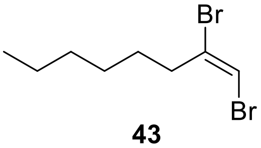

43

The reaction of **S43** (55.1 mg, 0.50 mmol), DBDMH (214 mg, 0.75 mmol) and TEMPO (15.6 mg, 0.10 mmol) in DCE (2.0 mL) at 60 °C for 0.5 h, affords 83.7 mg (62 %) of **43** as a white solid (etroleum ether/ethyl acetate = 100:1). **^{1}H NMR** (400 MHz, $CDCl_3$) δ 6.40 (s, 1H), 2.59 (t, J = 7.4 Hz, 2H), 1.62 – 1.53 (m, 2H), 1.38 – 1.28 (m, 6H), 0.95 – 0.88 (m, 3H). **^{13}C NMR** (101 MHz, $CDCl_3$) δ 127.0, 102.1, 36.9, 31.5, 28.0, 27.0, 22.5, 14.0. **MS (EI)** m/z (%): 109.1 (100), 67.0 (65), 267.9 (M$^+$, 10), 269.9 (15, for ^{81}Br), 271.9 (10, for ^{81}Br).

(*E*)-(1,2-dibromovinyl)benzene (44)[9]

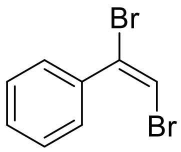

44

The reaction of **S44** (51.1 mg, 0.50 mmol), DBDMH (214 mg, 0.75 mmol) and TEMPO (15.6 mg, 0.10 mmol) in DCE (2.0 mL) at 60 °C for 3 h, affords 76.0 mg (58 %) of **44** as a white solid (petroleum ether/ethyl acetate = 100:1). **^{1}H NMR** (400 MHz, $CDCl_3$) δ 7.55 – 7.48 (m, 2H), 7.42 – 7.34 (m, 3H), 6.81 (d, *J* = 1.9 Hz, 1H). **^{13}C NMR** (101 MHz, $CDCl_3$) δ 137.0, 129.4, 129.1, 128.3, 121.3, 103.0. **MS (EI)** m/z (%): 102.0 (100), 180.9 (50), 259.9 (M^+, 20), 261.8 (35, for ^{81}Br), 263.8 (20, for ^{81}Br).

(*E*)-(1,2-dibromoprop-1-en-1-yl)benzene (45)[7]

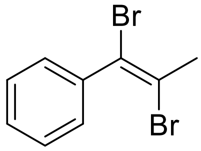

45

The reaction of **S45** (58.1 mg, 0.50 mmol), DBDMH (214 mg, 0.75 mmol) and TEMPO (15.6 mg, 0.10 mmol) in DCE (2.0 mL) at 60 °C for 3 h, affords 100.7 mg (73 %) of **45** as a white solid (petroleum ether/ethyl acetate = 100:1). **^{1}H NMR** (400 MHz, $CDCl_3$) δ 7.38 – 7.30 (m, 5H), 2.60 (s, 3H). **^{13}C NMR** (101 MHz, $CDCl_3$) δ 140.8, 129.1, 128.6, 128.2, 117.2, 116.8, 29.3. **MS (EI)** m/z (%): 115.0 (100), 194.9 (20), 273.9 (M^+, 10), 275.9 (25, for ^{81}Br), 277.9 (10, for ^{81}Br).

(*E*)-(1,2-dibromohex-1-en-1-yl)benzene (46)[7]

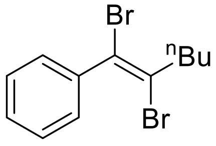

46

The reaction of **S46** (79.1 mg, 0.50 mmol), DBDMH (214 mg, 0.75 mmol) and TEMPO (15.6 mg, 0.10 mmol) in DCE (2.0 mL) at 60 °C for 3 h, affords 111.3 mg (70 %) of **46** as a white solid (petroleum ether/ethyl acetate = 100:1). **^{1}H NMR** (400

MHz, $CDCl_3$) δ 7.37 – 7.25 (m, 5H), 2.89 – 2.80 (m, 2H), 1.75 – 1.61 (m, 2H), 1.51 – 1.38 (m, 2H), 0.98 (t, *J* = 7.3 Hz, 3H). **^{13}C NMR** (101 MHz, $CDCl_3$) δ 140.9, 129.1, 128.4, 128.2, 123.6, 116.2, 40.8, 29.6, 21.7, 14.0. **MS (EI)** m/z (%): 115.0 (100), 194.9 (25), 315.9 (M^+, 10), 317.9 (25, for ^{81}Br), 319.9 (10, for ^{81}Br).

(*E*)-1,2-dibromo-1,2-diphenylethene (47)[10]

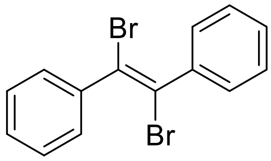

47

The reaction of **S47** (70.1 mg, 0.50 mmol), DBDMH (214 mg, 0.75 mmol) and TEMPO (15.6 mg, 0.10 mmol) in DCE (2.0 mL) at 60 °C for 3 h, affords 123.4 mg (73 %) of **47** as a white solid (petroleum ether/ethyl acetate = 100:1). **^{1}H NMR** (400 MHz, $CDCl_3$) δ 7.57 – 7.49 (m, 4H), 7.46 – 7.34 (m, 6H). **^{13}C NMR** (101 MHz, $CDCl_3$) δ 140.8, 129.1, 128.9, 128.4, 118.1. **MS (EI)** m/z (%): 178.1 (100), 210.0 (80), 257.9 ($[M\text{-}Br]^+$, 10), 259.9 (10, for ^{81}Br), 337.9 (M^+, 5).

(D) TEMPO$^+$-catalysed aromatic halogenation

Typical procedure:

Substrate **S48-S69** (0.50 mmol), NCS (80.2 mg, 0.60 mmol), [TEMPO][OTf] (30.5 mg, 0.10 mmol) or DMSO (7.8 mg ,0.10 mmol) and $CHCl_3$ (2.0 mL) was added to a reaction tube with a magnetic bar. The mixture was stirred at 25 °C for 12 h. Then, the reaction mixture was quenched with saturated Na_2SO_3 aqueous solution (2 mL). The aqueous phase was diluted with water (3 mL) and extracted with EtOAc (5 mL × 3). The combined organic extracts were dried over anhydrous Na_2SO_4, filtered, and concentrated under reduced pressure. The residue was purified over silica gel chromatography to afford **48-69**.

4-chloro-1-phenyl-1H-pyrazole (48)[11]

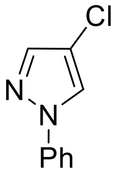

48

The reaction of **S48** (72.1 mg, 0.50 mmol), NCS (80.2 mg, 0.60 mmol) and [TEMPO][OTf] (30.5 mg, 0.10 mmol) in $CHCl_3$ (2.0 mL) at 25 °C for 12 h affords 85.7 mg (96 %) of **48** as a white solid (petroleum ether/ethyl acetate = 20:1). W/o catalyst: trace. DMSO catalysis: 96%. **^{1}H NMR** (400 MHz, $CDCl_3$) δ 7.86 (s, 1H), 7.64 – 7.57 (m, 3H), 7.45 – 7.38 (m, 2H), 7.31 – 7.24 (m, 1H). **^{13}C NMR** (101 MHz, $CDCl_3$) δ 139.6, 139.3, 129.4, 126.9, 124.7, 118.8, 112.3. **MS (EI)** m/z (%): 77.1 (75), 116.1 (80), 178.0 (M+, 100), 180.0 (30, for ^{37}Cl).

ethyl 3-chloro-1H-indole-2-carboxylate (49)[11]

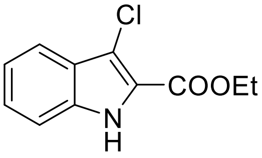

49

The reaction of **S49** (94.6 mg, 0.50 mmol), NCS (133.5 mg, 1.0 mmol) and [TEMPO][OTf] (30.5 mg, 0.10 mmol) in $CHCl_3$ (2.0 mL) at 25 °C for 12 h affords 83.9 mg (75 %) of **49** as a white solid (petroleum ether/ethyl acetate = 20:1). W/o catalyst: trace. DMSO catalysis: 97%. **^{1}H NMR** (400 MHz, $CDCl_3$) δ 9.22 (s, 1H), 7.73 – 7.68 (m, 1H), 7.43 – 7.31 (m, 2H), 7.21 (ddd, *J* = 8.0, 6.2, 1.8 Hz, 1H), 4.47 (q, *J* = 7.1 Hz, 2H), 1.45 (t, *J* = 7.1 Hz, 3H). **^{13}C NMR** (101 MHz, $CDCl_3$) δ 161.2, 134.8, 126.5, 126.2, 122.3, 121.2, 120.2, 112.4, 112.1, 61.4, 14.3. **MS (EI)** m/z (%): 149.1 (30),177.1 (100), 223.1 (M^+, 60), 225.1 (20, for ^{37}Cl).

3-chloro-2,6-dimethoxypyridine (50)[12]

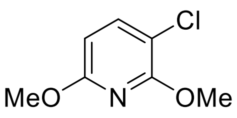

50

The reaction of **S50** (69.6 mg, 0.50 mmol), NCS (133.5 mg, 1.0 mmol) and [TEMPO][OTf] (30.5 mg, 0.10 mmol) in $CHCl_3$ (2.0 mL) at 25 °C for 12 h affords 52.1 mg (60 %) of **50** as a white solid (petroleum ether/ethyl acetate = 20:1). W/o catalyst: trace. DMSO catalysis: 83%. **^{1}H NMR** (400 MHz, $CDCl_3$) δ 7.49 (dd, J = 8.3, 0.9 Hz, 1H), 6.27 (dd, J = 8.3, 0.9 Hz, 1H), 4.00 (s, 3H), 3.90 (s, 3H). **^{13}C NMR** (101 MHz, $CDCl_3$) δ 161.2, 157.5, 140.5, 107.8, 101.9, 54.0, 53.7. **MS (EI)** m/z (%): 64.0 (100), 80.0 (80), 129.9 (60), 143.9 (85), 173.0 (M^+, 100), 175.0 (30, for ^{37}Cl).

4,5-dichloro-7H-pyrrolo[2,3-d]pyrimidine (51)[13]

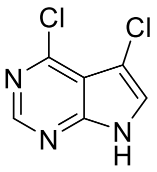

51

The reaction of **S51** (76.8 mg, 0.50 mmol), NCS (80.2 mg, 0.60 mmol) and [TEMPO][OTf] (30.5 mg, 0.10 mmol) in $CHCl_3$ (2.0 mL) at 25 °C for 12 h affords 74.3 mg (79 %) of **51** as a white solid (petroleum ether/ethyl acetate = 5:1). W/o catalyst: 15%. DMSO catalysis: 91%. **^{1}H NMR** (400 MHz, DMSO-d_6) δ 12.88 (s, 1H), 8.63 (s, 1H), 7.91 (s, 1H). **^{13}C NMR** (101 MHz, DMSO-d_6) δ 151.1, 150.5, 149.8, 126.1, 112.5, 101.4. **MS (EI)** m/z (%): 152.0 (50), 187.0 (M^+, 100), 189.0 (60, for ^{37}Cl).

2-chloro-5-phenylthiophene (52)[14]

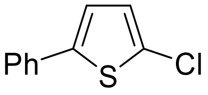

52

The reaction of **S52** (80.1 mg, 0.50 mmol), NCS (100.1 mg, 0.75 mmol) and [TEMPO][OTf] (30.5 mg, 0.10 mmol) in $CHCl_3$ (2.0 mL) at 25 °C for 12 h affords 82.7 mg (85 %) of **52** as a white solid (petroleum ether/ethyl acetate = 10:1). W/o catalyst: 12%. DMSO catalysis: 91%. **^{1}H NMR** (400 MHz, $CDCl_3$) δ 7.54 – 7.47 (m, 2H), 7.40

– 7.34 (m, 2H), 7.32 – 7.26 (m, 1H), 7.06 (d, *J* = 3.9 Hz, 1H), 6.88 (d, *J* = 3.9 Hz, 1H). **^{13}C NMR** (101 MHz, $CDCl_3$) δ 142.9, 133.7, 129.1, 129.0, 127.8, 127.1, 125.5, 122.2. **MS (EI)** m/z (%): 115.1 (50), 194.0 (M^+, 100), 196.0 (35, for ^{37}Cl).

5-chloro-2,3-dihydrobenzofuran (53)

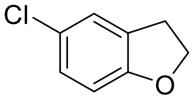

53

The reaction of **S53** (60.1 mg, 0.50 mmol), NCS (80.2 mg, 0.60 mmol) and [TEMPO][OTf] (30.5 mg, 0.10 mmol) in $CHCl_3$ (2.0 mL) at 25 °C for 12 h affords 70.3 mg (91 %) of **53** as a white solid (petroleum ether/ethyl acetate = 20:1). W/o catalyst: trace. DMSO catalysis: 94%. **^{1}H NMR** (400 MHz, $CDCl_3$) δ 7.14 (dt, *J* = 2.3, 1.2 Hz, 1H), 7.05 (dd, *J* = 8.5, 2.1 Hz, 1H), 6.69 (d, *J* = 8.5 Hz, 1H), 4.58 (t, *J* = 8.7 Hz, 2H), 3.27 – 3.14 (m, 2H). **^{13}C NMR** (101 MHz, $CDCl_3$) δ 158.7, 128.8, 127.7, 125.0, 124.9, 110.2, 71.6, 29.7. **HRMS (EI)** exact mass calc'd for C_7H_8ClO ($[M]^+$): 154.0179; found *m/z*: 154.0179, 156.0152 (for ^{37}Cl).

1-chloro-2-methoxynaphthalene (54)[15]

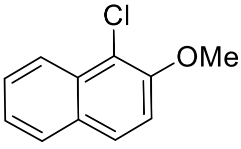

54

The reaction of **S54** (79.1 mg, 0.50 mmol), NCS (100.1 mg, 0.75 mmol) and [TEMPO][OTf] (30.5 mg, 0.10 mmol) in $CHCl_3$ (2.0 mL) at 25 °C for 12 h affords 77.06 mg (80 %) of **54** as a white solid (petroleum ether/ethyl acetate = 20:1). W/o catalyst: trace. DMSO catalysis: 95%. **^{1}H NMR** (400 MHz, $CDCl_3$) δ 8.21 (dt, *J* = 8.5, 0.9 Hz, 1H), 7.81 – 7.73 (m, 2H), 7.55 (ddd, *J* = 8.4, 6.8, 1.3 Hz, 1H), 7.39 (ddd, *J* = 8.1, 6.8, 1.2 Hz, 1H), 7.27 (d, *J* = 9.0 Hz, 1H), 4.01 (s, 3H). **^{13}C NMR** (101 MHz, $CDCl_3$) δ 152.5, 131.8, 129.5, 128.0, 127.9, 127.4, 124.3, 123.4, 116.8, 113.6, 56.9. **MS (EI)** m/z (%): 149.0 (100), 192.0 (M^+, 55), 194.0 (20, for ^{37}Cl).

2-(5-chloro-2-methoxyphenyl)acetic acid (55)

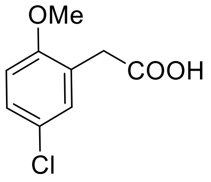

55

The reaction of **S55** (83.1 mg, 0.50 mmol), NCS (100.1 mg, 0.75 mmol) and [TEMPO][OTf] (30.5 mg, 0.10 mmol) in $CHCl_3$ (2.0 mL) at 25 °C for 12 h affords 65.2 mg (65%) of **55** as a white solid (petroleum ether/ethyl acetate = 2:1, with 1% of HOAc). W/o catalyst: trace. DMSO catalysis: 71%. **^{1}H NMR** (400 MHz, $CDCl_3$) δ 11.53 (s, 1H), 7.22 (dd, *J* = 8.7, 2.6 Hz, 1H), 7.16 (d, *J* = 2.6 Hz, 1H), 6.79 (d, *J* = 8.7 Hz, 1H), 3.80 (s, 3H), 3.62 (s, 2H). **^{13}C NMR** (101 MHz, $CDCl_3$) δ 177.5, 156.1, 130.8, 128.5, 125.3, 123.9, 111.6, 55.8, 35.4. **HRMS (ESI)** exact mass calc'd for $C_9H_8ClO_3$ ($[M-H]^-$): 199.0164; found *m/z*: 199.0160, 201.0133 (for ^{37}Cl).

ethyl 2-benzamido-3-(3-chloro-4-hydroxyphenyl)propanoate (56)

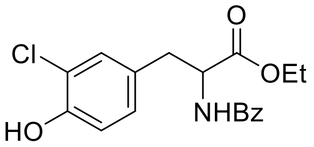

56

The reaction of **S56** (156.7 mg, 0.50 mmol), NCS (80.2 mg, 0.60 mmol) and [TEMPO][OTf] (30.5 mg, 0.10 mmol) in $CHCl_3$ (2.0 mL) at 25 °C for 12 h affords 140.9 mg (81 %) of **56** as a white solid (petroleum ether/ethyl acetate = 2:1). W/o catalyst: trace. DMSO catalysis: 86%. **^{1}H NMR** (400 MHz, $CDCl_3$) δ 7.76 (d, *J* = 7.6 Hz, 2H), 7.50 (t, *J* = 7.4 Hz, 1H), 7.41 (t, *J* = 7.6 Hz, 2H), 7.10 (d, *J* = 2.1 Hz, 1H), 6.95 – 6.89 (m, 1H), 6.89 – 6.82 (m, 2H), 6.73 (s, 1H), 5.03 (q, *J* = 6.0 Hz, 1H), 4.23 (q, *J* = 7.1 Hz, 2H), 3.15 (qd, *J* = 14.0, 5.5 Hz, 2H), 1.29 (t, *J* = 7.1 Hz, 3H). **^{13}C NMR** (101 MHz, $CDCl_3$) δ 171.6, 167.1, 150.9, 133.5, 131.9, 130.0, 129.0, 128.6, 128.5, 127.0, 119.9, 116.3, 61.9, 53.6, 36.7, 14.1. **HRMS (ESI)** exact mass calc'd for $C_{18}H_{19}ClNO_4$ ($[M+H]^+$): 348.0997; found *m/z*: 348.0993, 350.0966 (for ^{37}Cl).

3-chloro-4-hydroxy-5-methoxybenzaldehyde (57)[16]

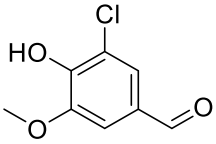

57

The reaction of **S57** (76.1 mg, 0.50 mmol), NCS (80.2 mg, 0.60 mmol) and [TEMPO][OTf] (30.5 mg, 0.10 mmol) in $CHCl_3$ (2.0 mL) at 25 °C for 12 h affords 77.4 mg (83 %) of **57** as a white solid (petroleum ether/ethyl acetate = 4:1). W/o catalyst: 26%. DMSO catalysis: 91%. **^{1}H NMR** (400 MHz, $CDCl_3$) δ 9.80 (s, 1H), 7.51 (d, *J* = 2.0 Hz, 1H), 7.35 (d, *J* = 2.0 Hz, 1H), 6.53 (s, 1H), 3.99 (s, 3H). **^{13}C NMR** (101 MHz, $CDCl_3$) δ 189.9, 147.9, 147.8, 129.2, 127.1, 119.8, 107.4, 56.6. **MS (EI)** m/z (%): 143.0 (20), 185.0 (M^+, 100), 187.0 (35, for ^{37}Cl).

2-(5-chloro-6-methoxynaphthalen-2-yl)propanoic acid (58)[12]

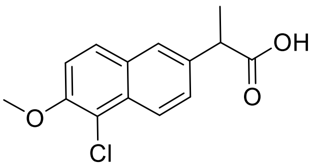

58

The reaction of **S58** (115.1 mg, 0.50 mmol), NCS (100.1 mg, 0.75 mmol) and [TEMPO][OTf] (30.5 mg, 0.10 mmol) in $CHCl_3$ (2.0 mL) at 25 °C for 12 h affords 111.2 mg (84 %) of **58** as a white solid (petroleum ether/ethyl acetate = 3:1, with 1% of HOAc). W/o catalyst: trace. DMSO catalysis: 96%. **^{1}H NMR** (400 MHz, $CDCl_3$) δ 8.18 (d, *J* = 8.8 Hz, 1H), 7.76 – 7.69 (m, 2H), 7.53 (dd, *J* = 8.8, 1.8 Hz, 1H), 7.29 (d, *J* = 9.0 Hz, 1H), 4.02 (s, 3H), 3.90 (q, *J* = 7.2 Hz, 1H), 1.60 (d, *J* = 7.2 Hz, 3H). **^{13}C NMR** (101 MHz, $CDCl_3$) δ 179.9, 152.6, 135.7, 131.2, 129.4, 127.8, 127.4, 126.4, 124.1, 116.8, 114.0, 57.0, 45.0, 18.0. **MS (ESI)** 204.0 (35), 219.0 (100), 263.0 [$(M-H)^-$, 20], 265.0 (7, for ^{37}Cl).

5-((4-chloro-3,5-dimethylphenoxy)methyl)oxazolidin-2-one (59)

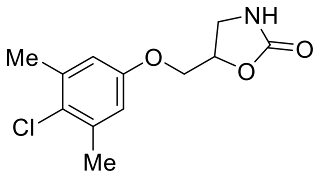

59

The reaction of **S59** (0.50 mmol), NCS (80.2 mg, 0.60 mmol) and [TEMPO][OTf] (30.5 mg, 0.10 mmol) in $CHCl_3$ (2.0 mL) at 25 °C for 12 h affords 116.3 mg (91%) of **59** as a white solid (petroleum ether/ethyl acetate = 1:1). W/o catalyst: trace. DMSO catalysis: 76%. **^{1}H NMR** (400 MHz, $CDCl_3$) δ 6.65 (s, 2H), 5.62 (s, 1H), 5.01 – 4.91 (m, 1H), 4.11 (d, *J* = 4.8 Hz, 2H), 3.77 (t, *J* = 8.8 Hz, 1H), 3.60 (dd, *J* = 8.7, 6.1 Hz, 1H), 2.34 (s, 6H). **^{13}C NMR** (101 MHz, $CDCl_3$) δ 155.9, 137.4, 127.2, 124.8, 114.6, 74.2, 68.2, 42.7, 20.9. **HRMS (ESI)** exact mass calc'd for $C_{12}H_{15}ClNO_3$ ($[M+H]^+$): 256.0741; found *m/z*: 256.0740, 258.0173 (for ^{37}Cl).

2-(5-chloro-2-((2,6-dichlorophenyl)amino)phenyl)acetic acid (60)[12]

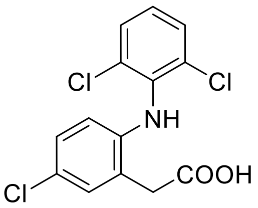

60

The reaction of **S60** (148.1 mg, 0.50 mmol), NCS (100.1 mg, 0.75 mmol) and [TEMPO][OTf] (30.5 mg, 0.10 mmol) in $CHCl_3$ (2.0 mL) at 25 °C for 12 h affords 133.9 mg (81 %) of **60** as a white solid (petroleum ether/ethyl acetate = 1:1, with 1% of HOAc). W/o catalyst: 81%. DMSO catalysis: 87%. **^{1}H NMR** (400 MHz, DMSO-d_6) δ 12.72 (s, 1H), 7.54 (d, *J* = 8.1 Hz, 2H), 7.32 – 7.26 (m, 2H), 7.22 (t, *J* = 8.1 Hz, 1H), 7.10 (dd, *J* = 8.6, 2.6 Hz, 1H), 6.23 (d, *J* = 8.6 Hz, 1H), 3.73 (s, 2H). **^{13}C NMR** (101 MHz, DMSO-d_6) δ 172.6, 141.9, 136.7, 130.8, 130.5, 129.2, 127.2, 126.2, 125.5, 123.7, 116.9, 37.0. **MS (ESI)** 283.9 (65), 299.9 (45), 327.9 [$(M-H)^-$, 20], 329.9 (100), 331.9 (30, for ^{37}Cl).

1-((4-chloronaphthalen-1-yl)oxy)-3-(isopropylamino)propan-2-ol (61)

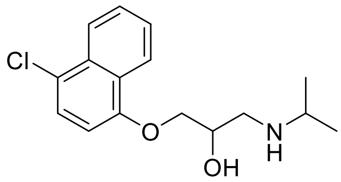

61

The reaction of **S61** (179.7 mg, 0.50 mmol), NCS (80.2 mg, 0.60 mmol) and [TEMPO][OTf] (30.5 mg, 0.10 mmol) in $CHCl_3$ (2.0 mL) at 25 °C for 12 h affords 114.6 mg (72 %) of **61** as a white solid (petroleum ether/ethyl acetate = 1:1, with 1% of NEt_3). W/o catalyst: 20%. DMSO catalysis: 75%. **^{1}H NMR** (400 MHz, DMSO-d_6) δ 9.35 (s, 1H), 8.92 (s, 1H), 8.39 (dd, *J* = 8.3, 1.2 Hz, 1H), 8.12 (d, *J* = 8.4 Hz, 1H), 7.73 (ddd, *J* = 8.3, 6.8, 1.3 Hz, 1H), 7.65 (ddd, *J* = 8.2, 6.9, 1.3 Hz, 1H), 7.61 (d, *J* = 8.2 Hz, 1H), 7.01 (d, *J* = 8.3 Hz, 1H), 6.08 (d, *J* = 5.0 Hz, 1H), 4.46 (dq, *J* = 8.1, 4.0 Hz, 1H), 4.21 (dt, *J* = 7.2, 3.4 Hz, 2H), 3.40 (d, *J* = 6.4 Hz, 1H), 3.26 (dd, *J* = 12.7, 3.0 Hz, 1H), 3.11 (dd, *J* = 12.6, 9.1 Hz, 1H), 1.32 (t, *J* = 6.4 Hz, 6H). **^{13}C NMR** (101 MHz, DMSO-d_6) δ 153.7, 130.9, 128.5, 126.8, 126.7, 126.4, 124.0, 123.1, 122.3, 106.2, 70.8, 65.7, 50.3, 47.4, 19.1, 18.7. **HRMS (ESI)** exact mass calc'd for $C_{16}H_{21}ClNO_2$ ($[M+H]^+$): 294.1259; found *m/z*: 294.1261, 296.1234 (for ^{37}Cl).

Methyl-2-(2-chloro-6,7-dihydrothieno[3,2-c]pyridin-5(4H)-yl)-2-(2-chlorophenyl)acetate (62)

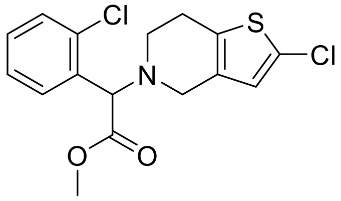

62

The reaction of **S62** (160.9 mg, 0.50 mmol), NCS (100.1 mg, 0.75 mmol) and [TEMPO][OTf] (30.5 mg, 0.10 mmol) in $CHCl_3$ (2.0 mL) at 25 °C for 12 h affords 89.1 mg (50%) of **62** as a white solid (petroleum ether/ethyl acetate = 20:1, with 1% of NEt_3). W/o catalyst: trace. DMSO catalysis: 45%. **^{1}H NMR** (400 MHz, $CDCl_3$) δ 7.67 – 7.63 (m, 1H), 7.43 – 7.39 (m, 1H), 7.28 (td, *J* = 4.4, 4.0, 1.8 Hz, 2H), 6.49 (s, 1H), 4.90 (s, 1H), 3.72 (s, 3H), 3.64 (dt, *J* = 14.5, 1.9 Hz, 1H), 3.53 (dt, *J* = 14.3, 1.8 Hz, 1H), 2.88

(t, J = 6.0 Hz, 2H), 2.78 – 2.73 (m, 2H). **^{13}C NMR** (101 MHz, $CDCl_3$) δ 171.0, 134.6, 133.4, 132.6, 131.9, 129.7, 129.3, 127.1, 127.0, 124.1, 67.5, 52.0, 50.0, 47.8, 25.2. **HRMS (ESI)** exact mass calc'd for $C_{16}H_{16}Cl_2NO_2S$ ($[M+H]^+$): 356.0273; found *m/z*: 356.0279, 358.0252 (for ^{37}Cl).

4-chloro-1-((2-chlorophenyl)diphenylmethyl)-1H-imidazole (63)[11]

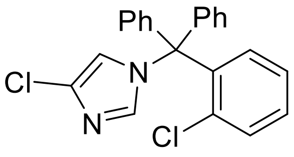

63

The reaction of **S63** (172.4 mg, 0.50 mmol), NCS (80.2 mg, 0.60 mmol) and [TEMPO][OTf] (30.5 mg, 0.10 mmol) in $CHCl_3$ (2.0 mL) at 25 °C for 12 h affords 85.3 mg (45 %) of **63** as a white solid (petroleum ether/ethyl acetate = 10:1). W/o catalyst: trace. DMSO catalysis: 49%. **^{1}H NMR** (400 MHz, $CDCl_3$) δ 7.43 (dd, J = 7.9, 1.5 Hz, 1H), 7.40 – 7.33 (m, 7H), 7.31 – 7.27 (m, 2H), 7.19 – 7.15 (m, 4H), 6.94 (dd, J = 7.9, 1.6 Hz, 1H), 6.66 (d, J = 1.6 Hz, 1H). **^{13}C NMR** (101 MHz, $CDCl_3$) δ 140.1, 139.6, 137.5, 135.5, 132.3, 130.3, 130.08, 130.06, 129.2, 128.4, 128.1, 127.1, 117.3, 75.8. **MS (ESI)** 379.1 [$(M + H)^+$, 100], 381.1 (65), 383.1 (15, for ^{37}Cl).

(2R,6aS,12aS)-7-chloro-8,9-dimethoxy-2-(prop-1-en-2-yl)-1,2,12,12a-tetrahydrochromeno[3,4-b]furo[2,3-h]chromen-6(6aH)-one (64)[11]

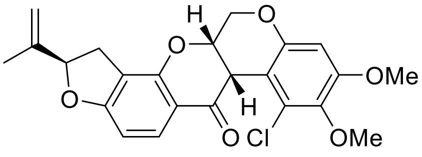

64

The reaction of **S64** (214.4 mg, 0.50 mmol), NCS (80.2 mg, 0.60 mmol) and [TEMPO][OTf] (30.5 mg, 0.10 mmol) in $CHCl_3$ (2.0 mL) at 25 °C for 12 h affords 132.9 mg (62 %) of **64** as a white solid (petroleum ether/ethyl acetate = 1:1). W/o catalyst: trace. DMSO catalysis: trace. **^{1}H NMR** (400 MHz, $CDCl_3$) δ 7.76 (d, J = 8.5 Hz, 1H), 6.53 (d, J = 8.5 Hz, 1H), 6.35 (s, 1H), 5.33 (dd, J = 9.9, 7.5 Hz, 1H), 5.15 – 5.06 (m, 2H), 4.95 (s, 1H), 4.53 (dd, J = 6.2, 2.2 Hz, 1H), 4.27 (ddd, J = 10.5, 4.5, 2.2

Hz, 1H), 4.10 (t, *J* = 11.0 Hz, 1H), 3.85 (s, 3H), 3.82 (s, 3H), 3.32 (dd, *J* = 15.8, 10.0 Hz, 1H), 2.99 (dd, *J* = 15.8, 7.5 Hz, 1H), 1.78 (s, 3H). **^{13}C NMR** (101 MHz, $CDCl_3$) δ 186.4, 167.3, 155.9, 153.8, 149.5, 143.0, 140.3, 130.2, 129.8, 112.6, 112.5 (2C), 107.2, 104.7, 99.4, 87.8, 72.1, 63.0, 60.7, 55.9, 44.0, 31.1, 16.9. **HRMS (ESI)** exact mass calc'd for $C_{23}H_{22}ClO_6$ ($[M+H]^+$): 429.1105; found m/z: 429.1106, 431.1079 (for ^{37}Cl).

8-chloro-7-hydroxy-3-(4-methoxyphenyl)-4H-chromen-4-one (65)

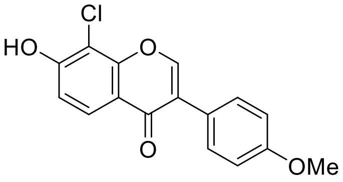

65

The reaction of **S66** (134.1 mg, 0.50 mmol), NCS (80.2 mg, 0.60 mmol) and [TEMPO][OTf] (30.5 mg, 0.10 mmol) in $CHCl_3$ (2.0 mL) at 25 °C for 12 h affords 139.2 mg (92 %) of **65** as a white solid (petroleum ether/ethyl acetate = 1:1). W/o catalyst: 25%. DMSO catalysis: 29%. **^{1}H NMR** (400 MHz, DMSO-*d*$_6$) δ 11.63 (s, 1H), 8.50 (s, 1H), 7.94 (d, *J* = 8.8 Hz, 1H), 7.53 (d, *J* = 8.8 Hz, 2H), 7.15 (d, *J* = 8.9 Hz, 1H), 7.00 (d, *J* = 8.8 Hz, 2H), 3.79 (s, 3H). **^{13}C NMR** (101 MHz, DMSO-*d*$_6$) δ 174.4, 159.1, 158.5, 153.4, 153.1, 130.2, 124.9, 123.8, 123.3, 117.4, 114.7, 113.7, 106.6, 55.2. **HRMS (ESI)** exact mass calc'd for $C_{16}H_{12}ClO_4$ ($[M+H]^+$): 303.0424; found *m/z*: 303.0421, 305.0394 (for ^{37}Cl).

(2R,3R,4R,5R)-2-(acetoxymethyl)-5-(2-amino-6,8-dichloro-9H-purin-9-yl)tetrahydrofuran-3,4-diyl diacetate (66)

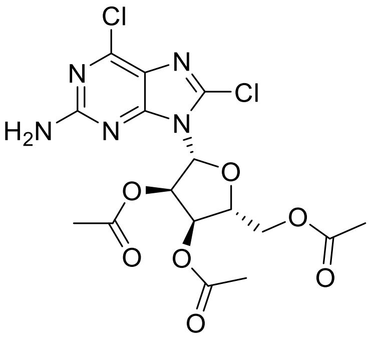

66

The reaction of **S66** (213.9 mg, 0.50 mmol), NCS (80.2 mg, 0.60 mmol) and [TEMPO][OTf] (30.5 mg, 0.10 mmol) in $CHCl_3$ (2.0 mL) at 25 °C for 12 h affords 219.6 mg (95 %) of **66** as a white solid (petroleum ether/ethyl acetate = 1:1). W/o catalyst: 25%. DMSO catalysis: 29%. **^{1}H NMR** (400 MHz, $CDCl_3$) δ 6.24 (dd, *J* = 5.6, 4.0 Hz, 1H), 6.04 (d, *J* = 4.1 Hz, 1H), 6.01 (t, *J* = 5.7 Hz, 1H), 5.37 (s, 2H), 4.48 (dd, *J* = 11.6, 3.8 Hz, 1H), 4.41 (td, *J* = 5.8, 3.7 Hz, 1H), 4.34 (dd, *J* = 11.6, 5.7 Hz, 1H), 2.15 (s, 3H), 2.13 (s, 3H), 2.01 (s, 3H). **^{13}C NMR** (101 MHz, $CDCl_3$) δ 170.6, 169.6, 169.4, 158.7, 153.3, 150.6, 139.7, 123.9, 87.3, 79.6, 72.0, 70.2, 62.6, 20.54, 20.49, 20.4. **HRMS (ESI)** exact mass calc'd for $C_{16}H_{18}Cl_2N_5O_7$ ([M+H]$^+$): 462.0584; found *m/z*: 462.0583, 464.0556 (for ^{37}Cl).

2-(5-bromo-2-((2,6-dichlorophenyl)amino)phenyl)acetic acid (67)

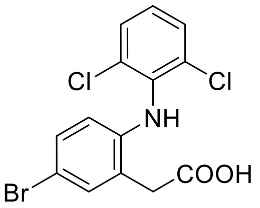

67

The reaction of **S60** (148.1 mg, 0.50 mmol), NBS (106.8 mg, 0.60 mmol) and [TEMPO][OTf] (30.5 mg, 0.10 mmol) in $CHCl_3$ (2.0 mL) at 25 °C for 12 h affords 131.2 mg (70%) of **67** as a white solid (petroleum ether/ethyl acetate = 1:1, with 1% of HOAc). W/o catalyst: 30%. DMSO catalysis: 35%. **^{1}H NMR** (400 MHz, DMSO-*d*$_6$) δ 12.73 (s, 1H), 7.53 (d, *J* = 8.0 Hz, 2H), 7.39 (d, *J* = 2.5 Hz, 1H), 7.31 (s, 1H), 7.25 – 7.18 (m, 2H), 6.17 (d, *J* = 8.5 Hz, 1H), 3.72 (s, 2H). **^{13}C NMR** (101 MHz, DMSO-*d*$_6$) δ 172.7, 142.4, 136.6, 133.3, 130.9, 130.1, 129.2, 126.3, 125.9, 117.2, 111.4, 36.9. **HRMS (ESI)** exact mass calc'd for $C_{14}H_9NO_2Cl_2Br$ ([M-H]$^-$): 371.9194; found *m/z*: 371.9197, 373.9173, 375.9148 (for ^{81}Br and ^{37}Cl).

***N*-(2-(1-(3-bromo-5-ethoxy-4-methoxyphenyl)-2-(methylsulfonyl)ethyl)-1,3-dioxoisoindolin-4-yl)acetamide (68)**

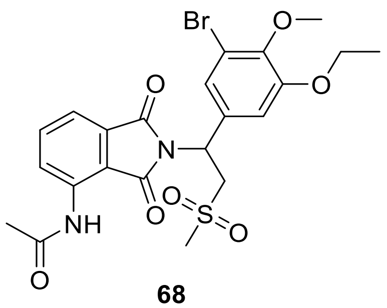

68

The reaction of **S68** (230.2 mg, 0.50 mmol), NBS (106.8 mg, 0.60 mmol) and [TEMPO][OTf] (30.5 mg, 0.10 mmol) in $CHCl_3$ (2.0 mL) at 25 °C for 12 h affords 258.9 mg (96%) of **68** as a white solid (petroleum ether/ethyl acetate = 2:1). W/o catalyst: trace. DMSO catalysis: trace. **^{1}H NMR** (400 MHz, $CDCl_3$) δ 9.47 (s, 1H), 8.78 (d, *J* = 8.5 Hz, 1H), 7.68 (dd, *J* = 8.5, 7.3 Hz, 1H), 7.52 (d, *J* = 7.3 Hz, 1H), 7.35 (s, 1H), 7.03 (s, 1H), 6.33 (dd, *J* = 11.9, 2.9 Hz, 1H), 4.59 – 4.48 (m, 1H), 4.08 (p, *J* = 6.9 Hz, 2H), 3.86 (s, 3H), 3.48 (dd, *J* = 14.6, 3.0 Hz, 1H), 3.03 (s, 3H), 2.27 (s, 3H), 1.46 (t, *J* = 7.0 Hz, 3H). **^{13}C NMR** (101 MHz, $CDCl_3$) δ 169.7, 169.1, 168.0, 150.1, 148.2, 137.6, 136.2, 131.0, 127.9, 125.0, 118.3, 115.6, 115.0, 113.4, 113.0, 64.7, 56.2, 53.6, 48.7, 40.9, 24.9, 14.5. **HRMS (ESI)** exact mass calc'd for $C_{22}H_{24}BrN_2O_7S$ ($[M+H]^+$): 539.0491; found *m/z*: 539.0488, 541.0468 (for ^{81}Br).

(S)-2-(5-iodo-6-methoxynaphthalen-2-yl)propanoic acid (69)

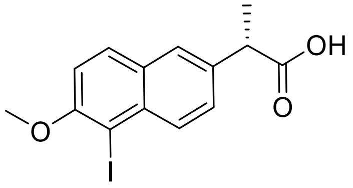

69

The reaction of **S69** (115.1 mg, 0.50 mmol), NIS (168.4 mg, 0.75 mmol) and [TEMPO][OTf] (30.5 mg, 0.10 mmol) in $CHCl_3$ (2.0 mL) at 25 °C for 12 h affords 113.6 mg (75%) of **69** as a white solid (petroleum ether/ethyl acetate = 4:1, with 1% of HOAc). W/o catalyst: trace. DMSO catalysis: 45%. **^{1}H NMR** (400 MHz, $CDCl_3$) δ 8.10 (d, *J* = 8.8 Hz, 1H), 7.77 (d, *J* = 8.9 Hz, 1H), 7.66 (d, *J* = 1.9 Hz, 1H), 7.50 (dd, *J* = 8.8, 1.9 Hz, 1H), 7.19 (d, *J* = 8.9 Hz, 1H), 4.01 (s, 3H), 3.90 (q, *J* = 7.1 Hz, 1H), 1.60 (d, *J* = 7.1 Hz, 3H). **^{13}C NMR** (101 MHz, $CDCl_3$) δ 180.1, 156.7, 135.7, 135.0, 131.8, 130.2,

129.8, 128.0, 126.5, 113.2, 87.4, 57.2, 44.9, 18.1. **HRMS (ESI)** exact mass calc'd for $C_{14}H_{12}IO_3$ ([M-H]$^-$): 354.9825; found *m/z*: 354.9831.

Typical procedure for Lewis-base-catalysed chlorination of diclofenac： diclofenac (0.5 mmol) and NCS (100.1 mg, 0.75 mmol) were dissolved in $CHCl_3$ (2 mL). Then the catalyst (0.1 mmol) was added to the solution under air at 25 °C and the mixture was stirred for 12 h. After concentrating in vacuum, the yield of product was determined by ^1H NMR analysis of the crude reaction mixture using 1,1,2,2-tetrachloroethane as the internal standard.

Supplementary Table 5. Chlorination of diclofenac with different catalysts.

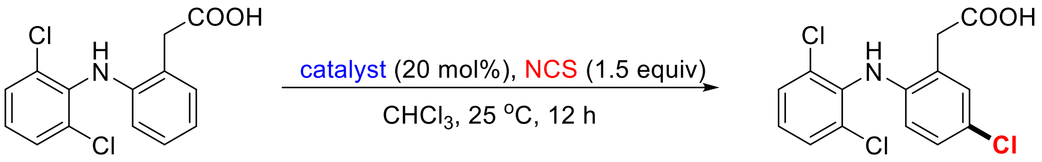

S60, diclofenac **60**

Entry	catalyst	Yield
1	-	2%
2	[TEMPO][OTf]	82%
3	DMSO	87%
4	$Ph_3P=S$	5%
5	Ph_2S	31%
6	Ph_2Se	37%
7	$^nBu_3P$	42%
8	2,4,6-trimethylaniline	20%

(E) Mechanistic experiments

(a) Radical inhibition experiment

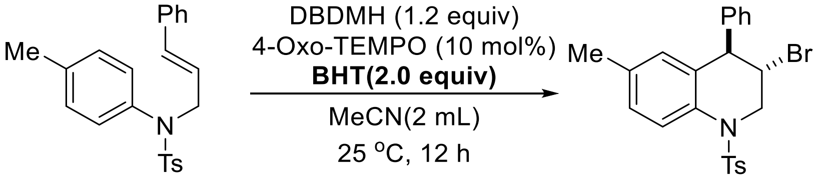

S1 **2**

70% yield

Substrate **S1** (0.20 mmol), DBDMH (68.6 mg, 0.24 mmol), 4-Oxo-TEMPO (0.02mmol), BHT (0.40 mmol) and MeCN (2.0 mL) were added to a reaction tube with a magnetic bar. The mixture was stirred at 25 °C for 12 h, the reaction mixture was quenched with saturated Na_2SO_3 aqueous solution (2 mL). The aqueous phase was diluted with water (3 mL) and extracted with EtOAc (5 mL × 3). The combined organic extracts were dried over anhydrous Na_2SO_4, filtered, and concentrated under reduced pressure. The residue was then analyzed by NMR spectrum. Yield was determined by ^{1}H-NMR using 1,1,2,2-tetrachloroethane as internal standard.

(b) Control experiments

[TEMPO][OTf] was prepared following a reported procedure[17], TfOH (6.0 mmol, 1.2 equiv) was added dropwise to a solution of TEMPO (5.0 mmol, 1.0 equiv) in Et_2O (20 mL) over 0.5 h at 0 °C. After the solution had become amber in color, NaOCl (2.5 mmol) was added over 1 h at 0 °C and the mixture was stirred for an additional 1 h at 0 °C. The formed yellow precipitate was filtered and washed with ice-cold 5% $NaHCO_3$ (5 mL), H_2O (20 mL) and Et_2O (40 mL). The solid was dried in vacuo over 24 h to yield [TEMPO][OTf] (77%) as a bright yellow solid.

Supplementary Table 6. Catalyst loading experiment of bromoarylation of **S1**.

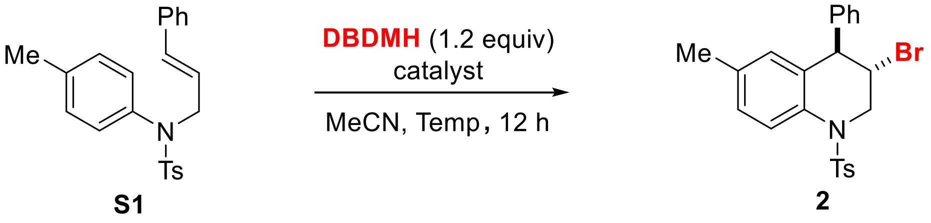

Entry	Catalyst	Temp	Yield
1	-	25 °C	trace
2	10 mol% TEMPO	25 °C	68%
3	10 mol% [TEMPO][OTf]	25 °C	98%
4	10 mol% 4-Oxo-TEMPO	25 °C	92%
5	2 mol% 4-Oxo-TEMPO	25 °C	61%
6	2 mol% 4-Oxo-TEMPO	60 °C	95%
7	2 mol% [TEMPO][OTf]	60 °C	88%

Substrate **S1** (75.5 mg, 0.20 mmol), DBDMH (68.6 mg, 0.24 mmol), catalyst and MeCN (2.0 mL) were added to a reaction tube with a magnetic bar. The mixture was

stirred at specified temperature for 12 h, the reaction mixture was quenched with saturated Na_2SO_3 aqueous solution (2.0 mL). The aqueous phase was diluted with water (3 mL) and extracted with EtOAc (5 mL × 3). The combined organic extracts were dried over anhydrous Na_2SO_4, filtered, and concentrated under reduced pressure. The residue was then analyzed by NMR spectrum. Yield was determined by ^{1}H-NMR using 1,1,2,2-tetrachloroethane as internal standard.

Supplementary Table 7. Catalyst loading experiment of chlorination of **S60**.

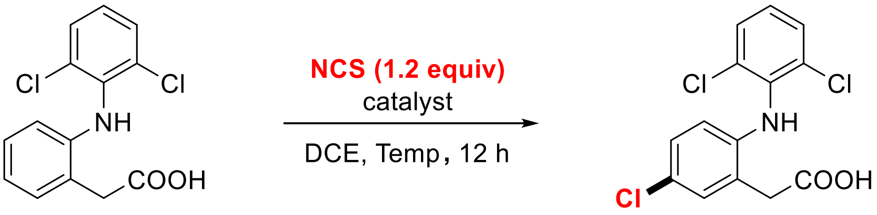

S60 **60**

Entry	Catalyst	Temp	Yield
1	-	25 °C	trace
2	-	60 °C	30%
3	20 mol% TEMPO	25 °C	55%
4	5 mol% TEMPO	60 °C	32%
5	10 mol% [TEMPO][OTf]	25 °C	86%
6	5 mol% [TEMPO][OTf]	60 °C	78%
7	2 mol% [TEMPO][OTf]	60 °C	72%

Diclofenac **S60** (148.1 mg, 0.50 mmol), NCS (80.2 mg, 0.60 mmol), catalyst and DCE (2.0 mL) were added to a reaction tube with a magnetic bar. The mixture was stirred at specific temperature for 12 h, the reaction mixture was quenched with saturated Na_2SO_3 aqueous solution (2.0 mL). The aqueous phase was diluted with water (3 mL) and extracted with EtOAc (5 mL × 3). The combined organic extracts were dried over anhydrous Na_2SO_4, filtered, and concentrated under reduced pressure. The residue was then analyzed by NMR spectrum. Yield was determined by ^{1}H-NMR using 1,1,2,2-tetrachloroethane as internal standard.

Supplementary Table 8. Catalyst loading experiment of bromination of **S58**.

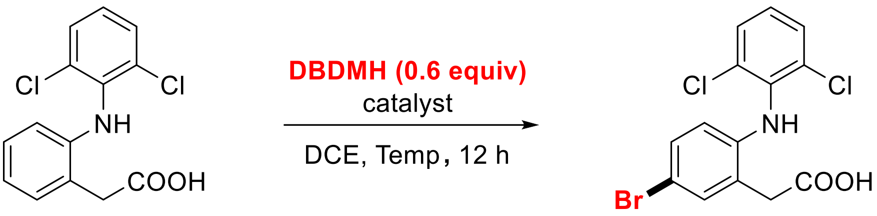

S60 **67**

Entry	Catalyst	Temp	Yield
1	-	25 °C	21%
2	-	60 °C	24%
3	20 mol% TEMPO	25 °C	37%
4	5 mol% TEMPO	60 °C	36%
5	10 mol% [TEMPO][OTf]	25 °C	64%
6	5 mol% [TEMPO][OTf]	60 °C	82%
7	2 mol% [TEMPO][OTf]	60 °C	70%

Diclofenac **S60** (148.1 mg, 0.50 mmol), DBDMH (85.7 mg, 0.30 mmol), catalyst and DCE (2.0 mL) were added to a reaction tube with a magnetic bar. The mixture was stirred at specific temperature for 12 h, the reaction mixture was quenched with saturated Na_2SO_3 aqueous solution (2.0 mL). The aqueous phase was diluted with water (3 mL) and extracted with EtOAc (5 mL × 3). The combined organic extracts were dried over anhydrous Na_2SO_4, filtered, and concentrated under reduced pressure. The residue was then analyzed by NMR spectrum. Yield was determined by ^{1}H-NMR using 1,1,2,2-tetrachloroethane as internal standard.

(c) NMR analysis of [TEMPO][OTf]

DBDMH (0.1 mmol) was dissolved in CD_3CN (1.0 mL), then different amount of [TEMPO][OTf] was added according to the given ratio. The mixture was analyzed by ^{13}C-NMR.

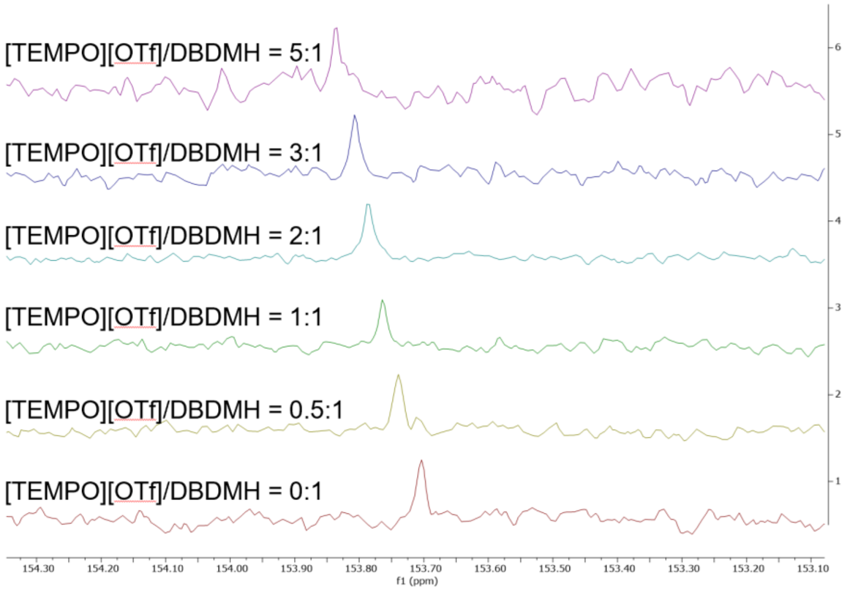

Supplementary Figure 1. C-2 chemical shift of DBDMH with different amount of [TEMPO][OTf]

DBDMH (0.1 mmol) was dissolved in CD_3CN (1.0 mL), then different amount of $In(OTf)_3$ was added according to the given ratio. The mixture was analyzed by ^{13}C-NMR.

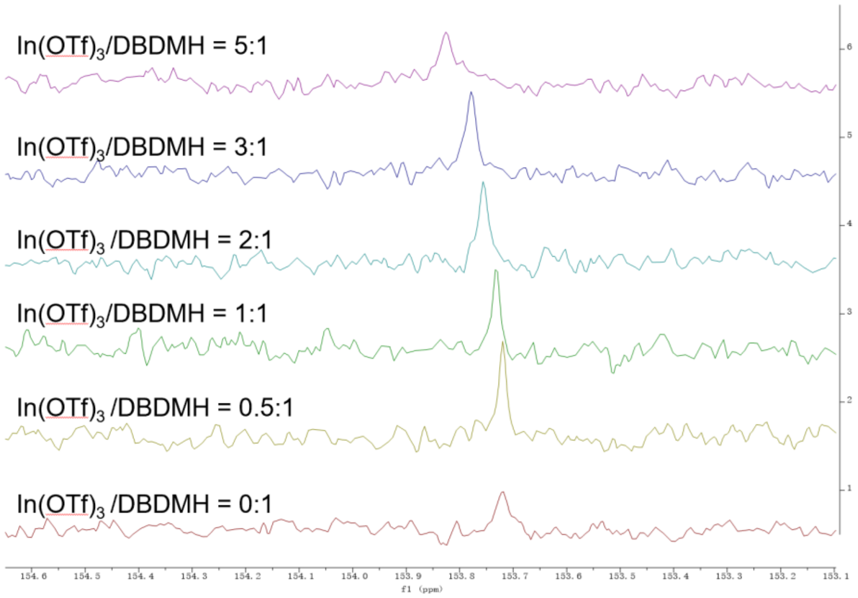

Supplementary Figure 2. C-2 chemical shift of DBDMH with different amount of $In(OTf)_3$

Ph$_3$P=O (0.1 mmol) was dissolved in CD$_3$CN (1.0 mL), then different amount of [TEMPO][OTf] was added according to the given ratio. The mixture was analyzed by ^{31}P-NMR.

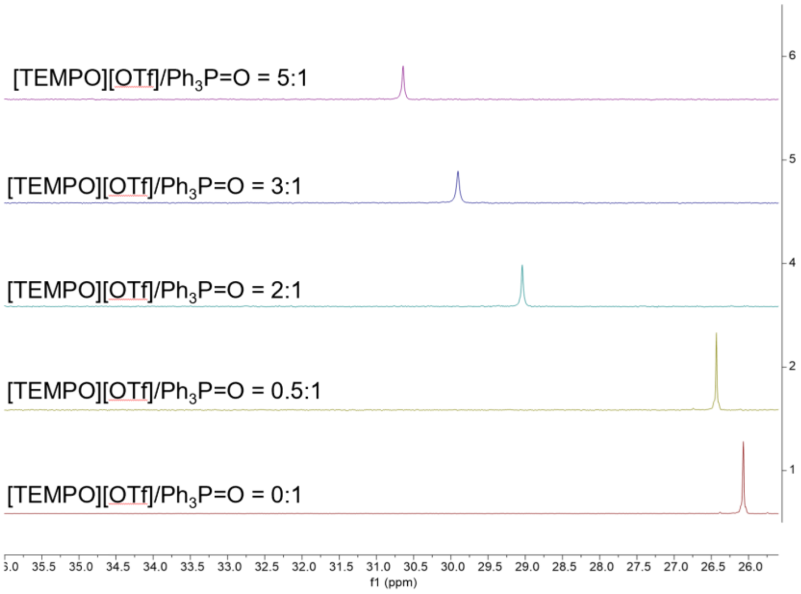

Supplementary Figure 3. ^{31}P-NMR chemical shift of Ph$_3$P=O with different amount of [TEMPO][OTf]

(d) Kinetic Studies

Dependence of the reaction rate on concentration of [TEMPO][OTf]

S1 (0.1 mmol), 1,1,2,2-tetrachloroethane (0.05 mmol) and DBDMH (0.12 mmol) were dissolved in CD$_3$CN (1.0 mL). [TEMPO][OTf] was added to the solution under air at room temperature. And the mixture was monitored by ^{1}H-NMR to determine the product yield. Kinetic profiles of different initial concentration of [TEMPO][OTf] (from 0.005 M to 0.025 M), the plot of k_{obs} vs [TEMPO][OTf] displayed a liner relationship in [TEMPO][OTf], which indicates a first-order kinetic dependence in [TEMPO][OTf].

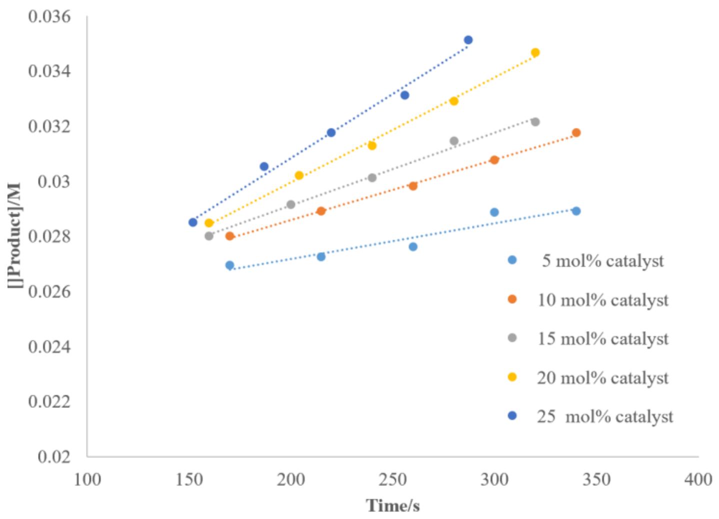

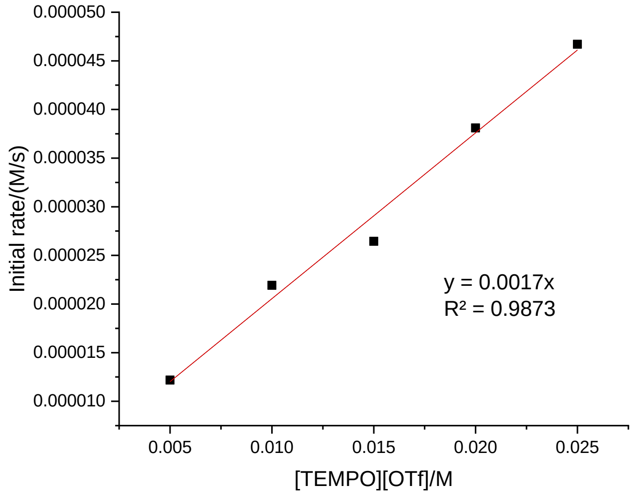

Supplementary Figure 4. Dependence of the reaction rate on concentration of [TEMPO][OTf]

**[TEMPO][OTf]/M**	**Reaction rate/(M/s)**
0.005	0.00001218
0.010	0.00002192
0.015	0.00002645
0.020	0.00003810
0.025	0.00004670

Dependence of the reaction rate on concentration of DBDMH

S1 (0.1 mmol), 1,1,2,2-tetrachloroethane (0.05 mmol) and [TEMPO][OTf] (0.12 mmol) were dissolved in CD_3CN (1.0 mL). DBDMH was added to the solution under

air at room temperature. And the mixture was monitored by ^{1}H-NMR to determine the product yield. Kinetic profiles of different initial concentration of DBDMH (from 0.06 M to 0.48 M), the plot of k_{obs} vs DBDMH displayed a liner relationship in DBDMH, which indicates a first-order kinetic dependence in DBDMH.

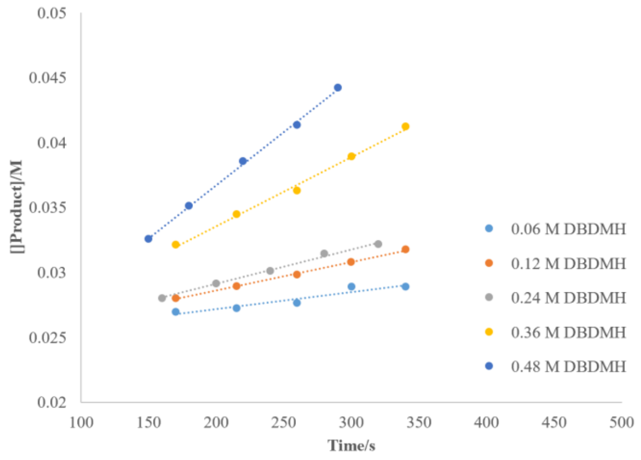

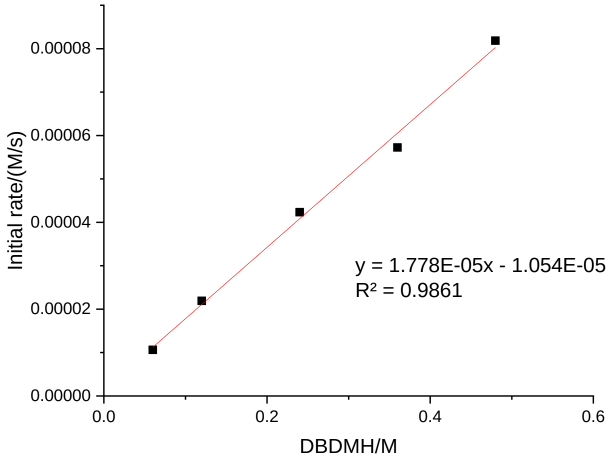

Supplementary Figure 5. Dependence of the reaction rate on concentration of DBDMH

DBDMH/M	Reaction rate/(M/s)
0.06	0.00001062
0.12	0.00002192
0.24	0.00004235
0.36	0.00005724
0.48	0.00008186

Dependence of the reaction rate on concentration of substrate 1a

DBDMH (0.12 mmol), 1,1,2,2-tetrachloroethane (0.05 mmol) and [TEMPO][OTf] (0.12 mmol) were dissolved in CD_3CN (1.0 mL). **S1** was added to the solution under air at room temperature. And the mixture was monitored by ^{1}H-NMR to determine the product yield. Kinetic profiles of different initial concentration of DBDMH (from 0.06 M to 0.48 M), the plot of k_{obs} vs **S1** displayed a liner relationship in **S1**, which indicates a first-order kinetic dependence in **S1**.

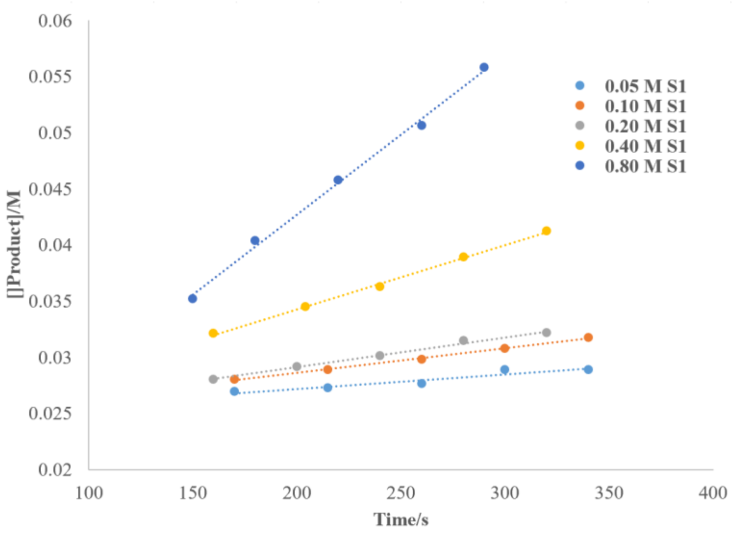

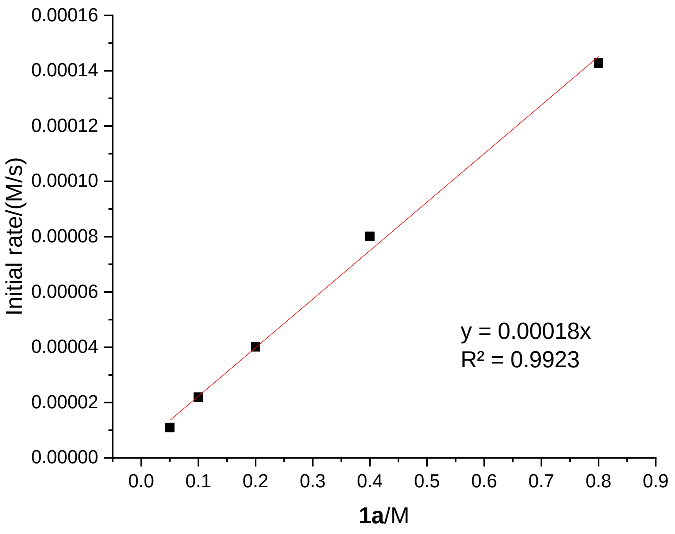

Supplementary Figure 6. Dependence of the reaction rate on concentration of DBDMH

**S1/M**	**Reaction rate/(M/s)**

0.05	0.00001062
0.10	0.00002192
0.20	0.00004235
0.40	0.00005724
0.80	0.00008186

(e) HRMS-ESI analysis of the reaction between DBDMH and TEMPO

TEMPO (0.1 mmol) and NCS (0.1 mmol) were dissolved in MeCN (2 mL). The solution was determined by the HRMS-ESI. The peak of $[TEMPO]^+$ was detected [HRMS (ESI) Calcd for $(C_9H_{18}NO)^+$: 156.1383, Found: 156.1387] with very high intensity. The peak of $[DMH]^-$ was detected [HRMS (ESI) Calcd for $(C_5H_7N_2O_2)^-$: 127.0513, Found: 127.0512] with very high intensity.

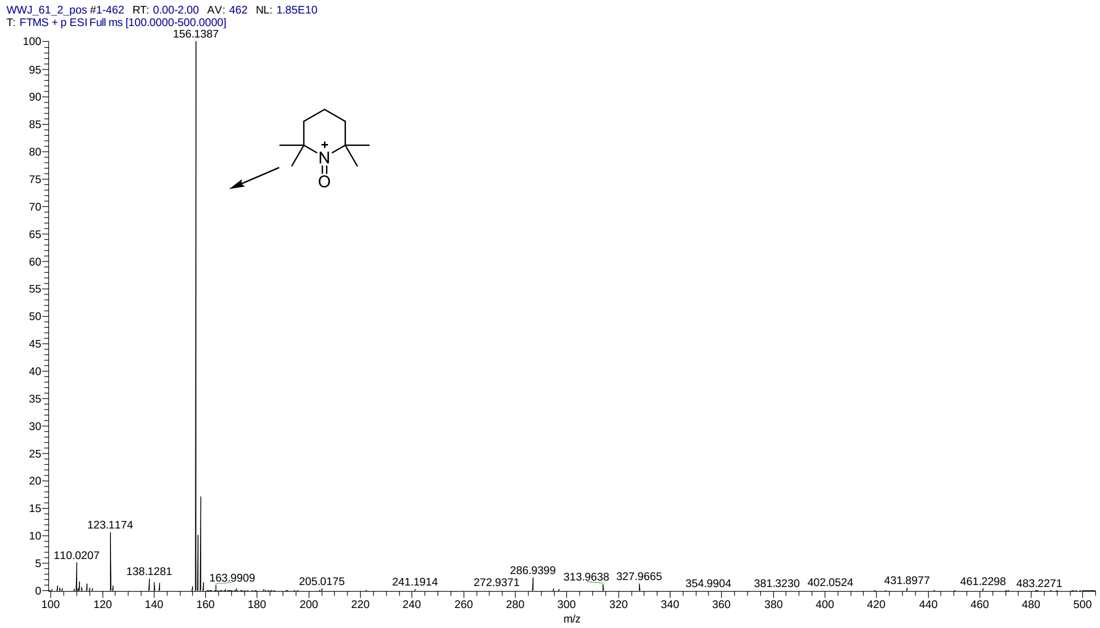

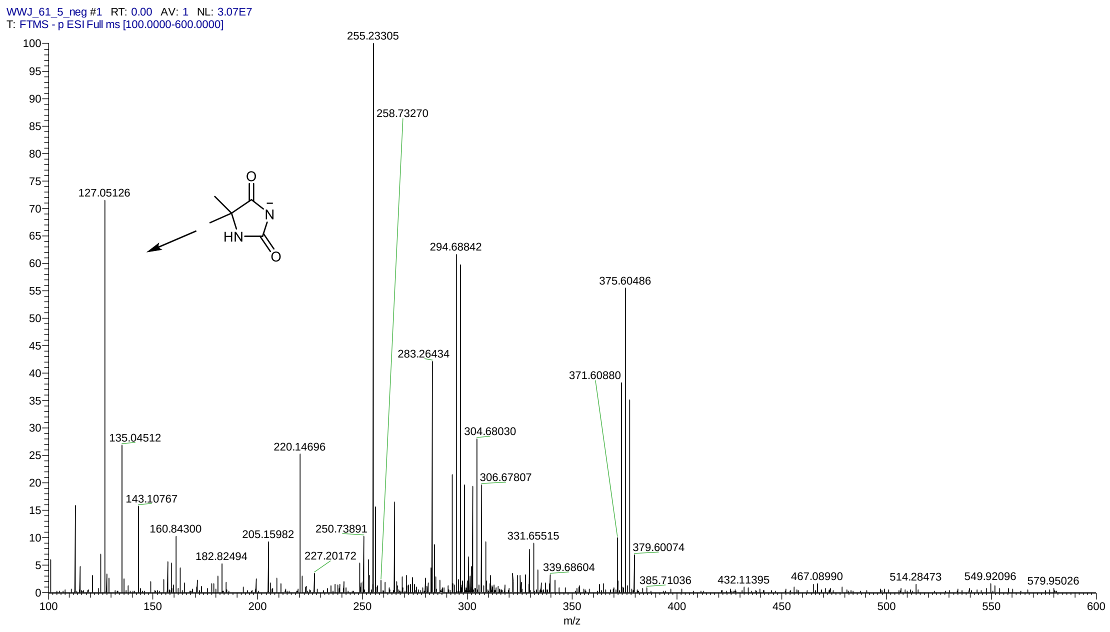

Supplementary Figure 7. HRMS-ESI analysis the reaction between DBDMH and TEMPO

(f) HRMS-ESI analysis of active intermediate

[TEMPO][OTf] (0.1 mmol) and NCS (0.1 mmol) were dissolved in MeCN (2 mL). The solution was determined by the HRMS-ESI. The peak of $[TEMPO][NCS]^+$ was detected [HRMS (ESI) Calcd for $(C_{13}H_{22}ClN_2O_3)^+$: 289.1313, Found: 289.1317, 291.1275 (for ^{37}Cl)] with very low intensity due to the easily decomposed property of $[TEMPO][NCS]^+$ during the ionization process.

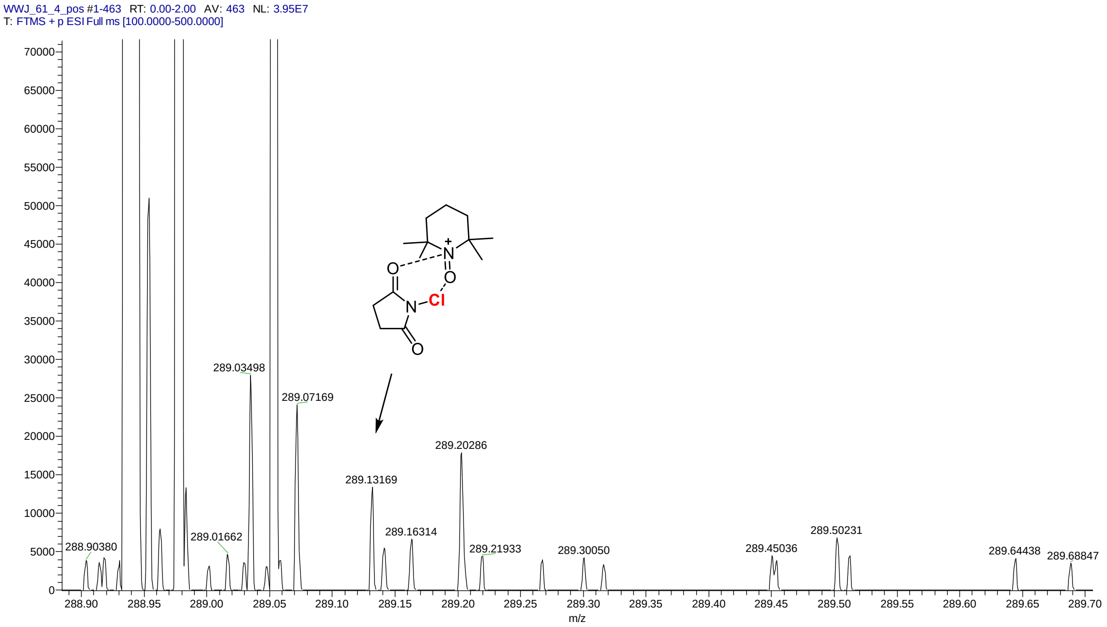

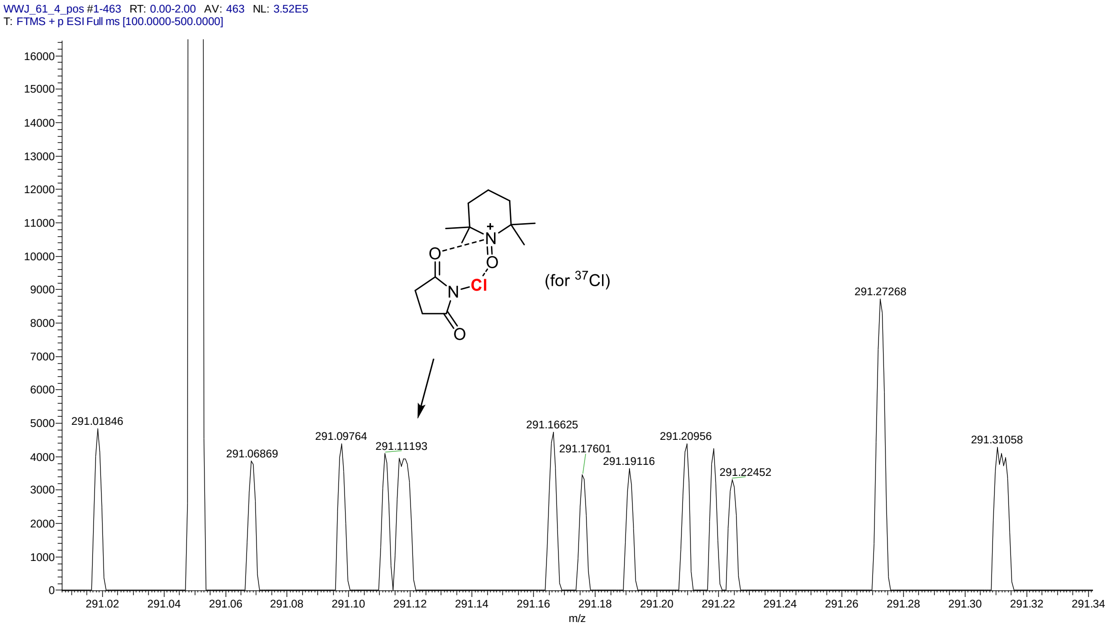

Supplementary Figure 8. HRMS-ESI capture of [TEMPO][NCS]$^+$

(g) Effect of various counterions on reaction outcome

Substrate **S1** (75.5 mg, 0.20 mmol), DCDMH or DBDMH (0.24 mmol), catalyst and MeCN (2.0 mL) were added to a reaction tube with a magnetic bar. The mixture was stirred at 25 ^{o}C for 12 h, the reaction mixture was quenched with saturated Na_2SO_3 aqueous solution (2.0 mL). The aqueous phase was diluted with water (3 mL) and extracted with EtOAc (5 mL × 3). The combined organic extracts were dried over

anhydrous Na_2SO_4, filtered, and concentrated under reduced pressure. The residue was then analyzed by NMR spectrum. Yield was determined by ^{1}H-NMR using 1,1,2,2-tetrachloroethane as internal standard.

Supplementary Table 9. Effect of various counterions on chloroarylation of **S1**

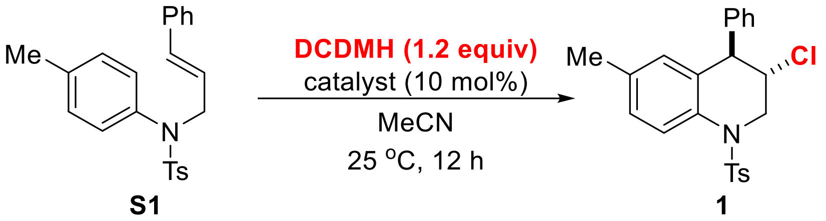

Entry	Catalyst	Yield
1	TEMPO	64%
2	[TEMPO][OTf]	75%
3	[TEMPO][PF_6]	62%
4	[TEMPO][ClO_4]	55%
5	[TEMPO][BF_4]	72%

Supplementary Table 10. Effect of various counterions on bromoarylation of **S1**

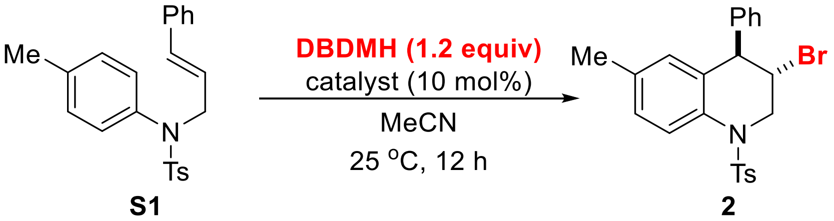

Entry	Catalyst	Yield
1	TEMPO	62%
2	[TEMPO][OTf]	98%
3	[TEMPO][PF_6]	70%
4	[TEMPO][ClO_4]	50%
5	[TEMPO][BF_4]	85%

(h) Effect of nitroxyl radicals on reaction outcome

Substrate **S1** (75.5 mg, 0.20 mmol), DCDMH (47.3 mg, 0.24 mmol), catalyst (0.02 mmol) and MeCN (2.0 mL) were added to a reaction tube with a magnetic bar. The mixture was stirred at 25 °C for 12 h, the reaction mixture was quenched with saturated Na_2SO_3 aqueous solution (2.0 mL). The aqueous phase was diluted with water (3 mL)

and extracted with EtOAc (5 mL × 3). The combined organic extracts were dried over anhydrous Na_2SO_4, filtered, and concentrated under reduced pressure. The residue was then analyzed by NMR spectrum. Yield was determined by ^{1}H-NMR using 1,1,2,2-tetrachloroethane as internal standard.

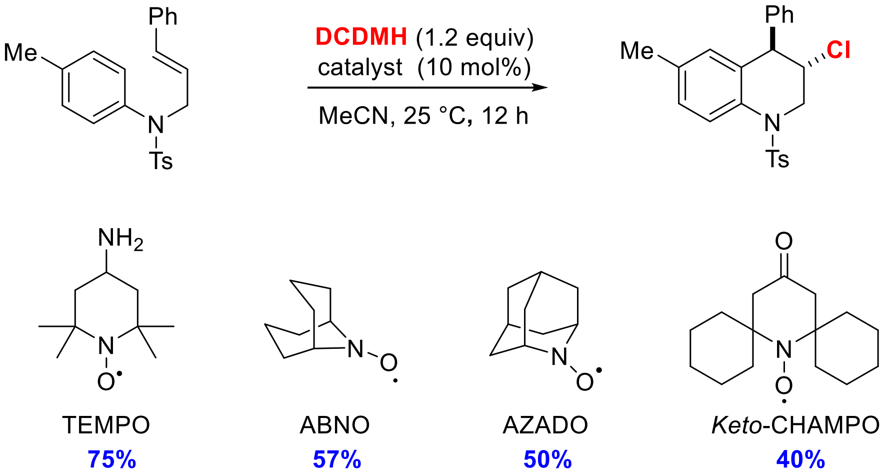

Supplementary Figure 9. Effect of nitroxyl radicals on reaction outcome

(i) DFT calculation

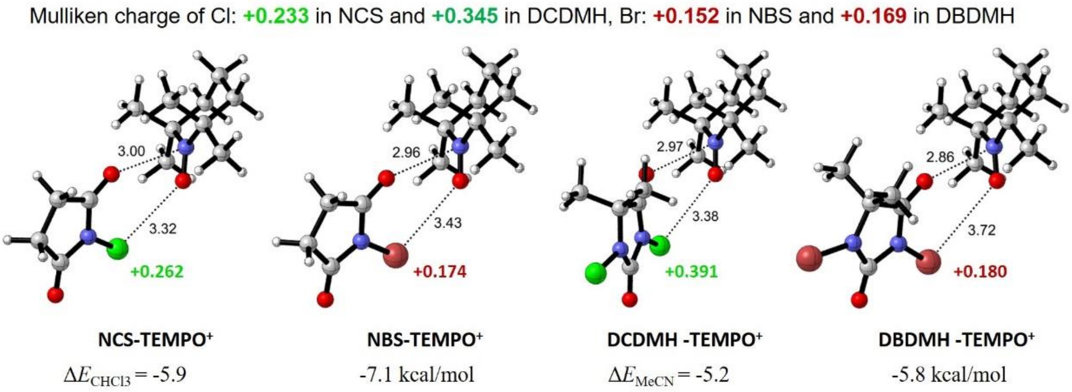

Supplementary Figure 10. Plausible intermediate from halogenating reagents and $TEMPO^+$. *ΔE* is energy change associated with halogenating-reagent-$TEMPO^+$ complex formation.

DFT calculations were performed with the Gaussian 09 program[18]. All geometry optimizations were carried out at the M06-2X[19] level of density functional theory with Grimme's D3 empirical dispersion correction[20], and using the basis set of 6-31+G(d,p)[21,22] for all the atoms (keyword 5D) and the PCM model[24,25] in chloroform or

acetonitrile as a solvent. The reported energies are Gibbs free energies in chloroform solution (ΔE).

Coordinates and Energies.

TEMPO$^+$ (in $CHCl_3$)

Sum of electronic and zero-point Energies= -483.053700

Center Number	Atomic Number	Atomic Type	Coordinates (Angstroms) X	Y	Z
1	7	0	-0.000020	-0.776063	-0.127258
2	8	0	-0.000035	-1.937161	-0.356426
3	6	0	1.350241	-0.074642	0.021755
4	6	0	-1.350237	-0.074636	0.021788
5	6	0	1.246517	1.306955	-0.645296
6	1	0	2.158756	1.845639	-0.370283
7	1	0	1.268194	1.169601	-1.732752
8	6	0	-1.246537	1.306982	-0.645215
9	1	0	-2.158759	1.845650	-0.370124
10	1	0	-1.268293	1.169654	-1.732673
11	6	0	0.000006	2.091270	-0.255670
12	1	0	0.000001	3.045143	-0.788847
13	1	0	0.000024	2.336516	0.811194
14	6	0	1.637025	0.018631	1.532670
15	1	0	1.505582	-0.948384	2.024004
16	1	0	2.688021	0.303560	1.621061
17	1	0	1.040299	0.774565	2.039504
18	6	0	2.395368	-0.950906	-0.656598
19	1	0	3.335095	-0.394638	-0.644499
20	1	0	2.544458	-1.894709	-0.129974
21	1	0	2.129815	-1.155803	-1.696213
22	6	0	-2.395449	-0.950797	-0.656551
23	1	0	-2.544465	-1.894698	-0.130095
24	1	0	-3.335182	-0.394545	-0.644235
25	1	0	-2.130029	-1.155487	-1.696242
26	6	0	-1.636890	0.018526	1.532726
27	1	0	-2.687548	0.304680	1.621202
28	1	0	-1.506543	-0.948842	2.023667
29	1	0	-1.039259	0.773530	2.039868

NCS (in $CHCl_3$)

Sum of electronic and zero-point Energies= -819.976941

Center Number	Atomic Number	Atomic Type	Coordinates (Angstroms) X	Y	Z
1	6	0	1.883312	-0.766993	-0.015855
2	6	0	1.883312	0.766993	0.015856
3	1	0	2.340628	-1.177026	-0.919079
4	1	0	2.379339	-1.219471	0.844995
5	1	0	2.379341	1.219471	-0.844993
6	1	0	2.340625	1.177025	0.919081
7	6	0	0.425762	-1.181802	-0.000066
8	8	0	-0.045442	-2.290247	0.006810
9	6	0	0.425762	1.181802	0.000063
10	8	0	-0.045442	2.290247	-0.006809
11	7	0	-0.324752	0.000000	-0.000001
12	17	0	-2.008734	0.000000	0.000000

NCS-TEMPO$^+$ (in $CHCl_3$)

Sum of electronic and zero-point Energies= -1303.040100

Center Number	Atomic Number	Atomic Type	Coordinates (Angstroms) X	Y	Z
1	6	0	-4.512304	-1.307837	0.650747
2	6	0	-3.104910	-1.766228	1.057167
3	1	0	-5.011868	-2.004567	-0.025595
4	1	0	-5.173566	-1.136031	1.502444
5	1	0	-2.821685	-2.728272	0.624701
6	1	0	-2.970285	-1.833607	2.138978
7	6	0	-4.324840	0.005834	-0.078388
8	8	0	-5.146507	0.733113	-0.567670
9	6	0	-2.159561	-0.709761	0.528137
10	8	0	-0.951800	-0.667495	0.607490
11	7	0	-2.938722	0.256477	-0.095085
12	7	0	1.733181	0.163503	-0.430071
13	8	0	0.821568	0.462378	-1.120958
14	6	0	2.398622	-1.190689	-0.650727
15	6	0	2.211202	1.173009	0.608288
16	6	0	3.914187	-0.920393	-0.735519
17	1	0	4.395609	-1.903027	-0.763531
18	1	0	4.128592	-0.428579	-1.692234
19	6	0	3.742217	1.275557	0.457396
20	1	0	4.090150	1.879063	1.301605

21	1	0	3.965308	1.839232	-0.456246
22	6	0	4.450975	-0.073684	0.413367
23	1	0	5.520789	0.092485	0.263418
24	1	0	4.353097	-0.599551	1.367880
25	6	0	2.026469	-2.110738	0.526268
26	1	0	0.956887	-2.060585	0.741409
27	1	0	2.271410	-3.125964	0.204587
28	1	0	2.594369	-1.900292	1.430771
29	6	0	1.850753	-1.756263	-1.952770
30	1	0	2.403418	-2.673959	-2.164373
31	1	0	0.789186	-1.998823	-1.867952
32	1	0	1.993499	-1.063286	-2.785312
33	6	0	1.526232	2.497258	0.302661
34	1	0	0.450140	2.441230	0.480882
35	1	0	1.954419	3.242876	0.976160
36	1	0	1.703297	2.816202	-0.727277
37	6	0	1.780087	0.662124	1.996051
38	1	0	1.808079	1.527669	2.662210
39	1	0	0.758454	0.275991	1.974138
40	1	0	2.451830	-0.093798	2.398356
41	17	0	-2.279599	1.623684	-0.823289

NBS (in $CHCl_3$)

Sum of electronic and zero-point Energies= -2931.300167

Center Number	Atomic Number	Atomic Type	Coordinates (Angstroms) X	Y	Z
1	6	0	-2.366820	-0.766554	0.017236
2	6	0	-2.366825	0.766550	-0.017228
3	1	0	-2.821457	-1.175924	0.922173
4	1	0	-2.863133	-1.221994	-0.841952
5	1	0	-2.863126	1.221985	0.841970
6	1	0	-2.821476	1.175918	-0.922159
7	6	0	-0.907751	-1.179184	0.000145
8	8	0	-0.442975	-2.290721	-0.007419
9	6	0	-0.907755	1.179183	-0.000158
10	8	0	-0.442982	2.290721	0.007407
11	7	0	-0.154968	0.000000	0.000008
12	35	0	1.681044	0.000001	0.000001

NBS-TEMPO⁺ (in $CHCl_3$)

Sum of electronic and zero-point Energies= -3414.365108

Center Number	Atomic Number	Atomic Type	Coordinates (Angstroms) X	Y	Z
1	6	0	-4.122323	1.902533	-0.656150
2	6	0	-2.658684	2.318880	-0.850539
3	1	0	-4.652650	2.516578	0.074696
4	1	0	-4.700676	1.910577	-1.582213
5	1	0	-2.364353	3.168194	-0.230168
6	1	0	-2.412217	2.560400	-1.886791
7	6	0	-4.079844	0.479922	-0.136591
8	8	0	-4.990465	-0.248211	0.154647
9	6	0	-1.833676	1.119434	-0.434779
10	8	0	-0.627508	1.005445	-0.428012
11	7	0	-2.720790	0.125998	-0.044585
12	35	0	-2.168835	-1.520333	0.549179
13	7	0	1.976385	-0.158932	0.378455
14	8	0	1.071975	-0.514711	1.051541
15	6	0	2.755585	1.080872	0.805684
16	6	0	2.345268	-0.999758	-0.839532
17	6	0	4.244183	0.675247	0.797885
18	1	0	4.810083	1.594791	0.976763
19	1	0	4.426782	0.011225	1.651662
20	6	0	3.861123	-1.264172	-0.738272
21	1	0	4.147136	-1.752875	-1.674898
22	1	0	4.033380	-1.985255	0.069766
23	6	0	4.690012	-0.009152	-0.490065
24	1	0	5.741937	-0.291114	-0.395911
25	1	0	4.632168	0.676884	-1.340908
26	6	0	2.442319	2.218370	-0.182437
27	1	0	1.370967	2.277744	-0.386055
28	1	0	2.756641	3.142079	0.309249
29	1	0	2.991252	2.135636	-1.118259
30	6	0	2.280275	1.454948	2.202015
31	1	0	2.900958	2.286435	2.542817
32	1	0	1.236980	1.778115	2.195435
33	1	0	2.393636	0.626236	2.904442
34	6	0	1.540105	-2.288108	-0.759068
35	1	0	0.471303	-2.094446	-0.875552
36	1	0	1.872796	-2.929691	-1.577711
37	1	0	1.708259	-2.812803	0.184558
38	6	0	1.959189	-0.214902	-2.106364
39	1	0	1.937079	-0.943746	-2.920000

40	1	0	0.964644	0.225789	-2.007191
41	1	0	2.680736	0.556550	-2.368201

TEMPO$^+$ (in MeCN)

Sum of electronic and zero-point Energies= -483.067016

Center Number	Atomic Number	Atomic Type	Coordinates (Angstroms) X	Y	Z
1	7	0	0.000034	-0.776195	-0.127275
2	8	0	0.000130	-1.937183	-0.356154
3	6	0	1.349209	-0.074592	0.021166
4	6	0	-1.349208	-0.074667	0.021164
5	6	0	1.246019	1.307402	-0.644635
6	1	0	2.158189	1.844665	-0.367715
7	1	0	1.266910	1.171313	-1.732028
8	6	0	-1.246097	1.307324	-0.644647
9	1	0	-2.158309	1.844531	-0.367762
10	1	0	-1.266958	1.171207	-1.732037
11	6	0	-0.000066	2.091081	-0.253196
12	1	0	-0.000088	3.045781	-0.784993
13	1	0	-0.000091	2.333640	0.814339
14	6	0	1.635112	0.016863	1.532308
15	1	0	1.498953	-0.950330	2.021718
16	1	0	2.686621	0.299765	1.621763
17	1	0	1.038545	0.773511	2.038253
18	6	0	2.394948	-0.949426	-0.657997
19	1	0	3.332260	-0.389170	-0.647103
20	1	0	2.546866	-1.891116	-0.128408
21	1	0	2.127395	-1.156528	-1.696778
22	6	0	-2.395003	-0.949484	-0.657940
23	1	0	-2.546886	-1.891185	-0.128374
24	1	0	-3.332312	-0.389226	-0.646963
25	1	0	-2.127527	-1.156546	-1.696747
26	6	0	-1.635066	0.016798	1.532311
27	1	0	-2.686523	0.299885	1.621811
28	1	0	-1.499071	-0.950447	2.021667
29	1	0	-1.038347	0.773291	2.038301

DCDMH (in MeCN)

Sum of electronic and zero-point Energies= -1374.104609

Center Number	Atomic Number	Atomic Type	Coordinates (Angstroms) X	Y	Z
1	6	0	0.800969	0.993707	-0.012851
2	6	0	-0.246851	-1.154429	-0.061851
3	8	0	-0.443539	-2.343461	-0.027451
4	6	0	-0.725720	1.137864	-0.001216
5	8	0	-1.357424	2.164914	0.016412
6	6	0	1.403244	1.748126	-1.192416
7	1	0	1.202123	2.815556	-1.079973
8	1	0	2.485582	1.594962	-1.207137
9	1	0	0.979222	1.397438	-2.135961
10	6	0	1.356548	1.463855	1.332153
11	1	0	2.443534	1.353609	1.340497
12	1	0	1.112237	2.519646	1.467415
13	1	0	0.931401	0.887956	2.158332
14	7	0	-1.237118	-0.144009	0.002282
15	7	0	0.926580	-0.464181	-0.189116
16	17	0	2.402469	-1.261717	0.014640
17	17	0	-2.879043	-0.504060	0.013011

DCDMH-TEMPO$^+$ (in MeCN)

Sum of electronic and zero-point Energies= -1857.179877

Center Number	Atomic Number	Atomic Type	Coordinates (Angstroms) X	Y	Z
1	6	0	-2.473524	-1.017410	0.576162
2	6	0	-3.329085	0.945626	-0.483206
3	8	0	-4.063064	1.769858	-0.965369
4	6	0	-1.390096	0.060452	0.479720
5	8	0	-0.247441	-0.026076	0.867563
6	7	0	-1.971161	1.150931	-0.119991
7	7	0	2.428425	0.167169	-0.417788
8	8	0	1.558094	0.712833	-1.003647
9	6	0	2.744229	-1.281410	-0.773769
10	6	0	3.211612	0.964009	0.618424
11	6	0	4.267777	-1.351167	-1.001768
12	1	0	4.507148	-2.413050	-1.114128
13	1	0	4.497475	-0.862157	-1.955682
14	6	0	4.703274	0.751465	0.289824
15	1	0	5.262501	1.214291	1.108671
16	1	0	4.940576	1.309407	-0.623747

17	6	0	5.089005	-0.713197	0.113795
18	1	0	6.147989	-0.771040	-0.150984
19	1	0	4.977577	-1.264872	1.052540
20	6	0	2.273207	-2.178647	0.386046
21	1	0	1.293795	-1.864953	0.755086
22	1	0	2.183449	-3.186133	-0.026732
23	1	0	2.981902	-2.215113	1.210926
24	6	0	1.968256	-1.615484	-2.039268
25	1	0	2.271362	-2.618685	-2.345765
26	1	0	0.890899	-1.616913	-1.858077
27	1	0	2.195151	-0.918482	-2.848839
28	6	0	2.799027	2.421361	0.475382
29	1	0	1.753635	2.566594	0.758100
30	1	0	3.429192	3.004010	1.150486
31	1	0	2.950116	2.785784	-0.543265
32	6	0	2.832029	0.443135	2.016355
33	1	0	3.194212	1.191598	2.725116
34	1	0	1.748715	0.357677	2.124022
35	1	0	3.303610	-0.505632	2.262657
36	17	0	-1.178755	2.602961	-0.418298
37	7	0	-3.558564	-0.366411	-0.178526
38	17	0	-5.137773	-0.967169	-0.163175
39	6	0	-2.836220	-1.225709	2.047285
40	1	0	-3.618696	-1.983649	2.129248
41	1	0	-1.952325	-1.575910	2.584738
42	1	0	-3.188672	-0.295788	2.500800
43	6	0	-2.015873	-2.302399	-0.104399
44	1	0	-1.145791	-2.702241	0.421652
45	1	0	-2.819396	-3.042100	-0.059827
46	1	0	-1.756767	-2.119589	-1.149675

DBDMH (in MeCN)

Sum of electronic and zero-point Energies= -5596.752631

Center Number	Atomic Number	Atomic Type	Coordinates (Angstroms) X	Y	Z
1	6	0	0.749358	1.327714	-0.017150
2	6	0	-0.096056	-0.903900	-0.063021
3	8	0	-0.186426	-2.107469	-0.048253
4	6	0	-0.784963	1.329062	0.003075
5	8	0	-1.501875	2.299289	0.032318
6	6	0	1.259562	2.098811	-1.229672

7	1	0	0.958186	3.145258	-1.146386
8	1	0	2.351462	2.049611	-1.258238
9	1	0	0.858193	1.679677	-2.155163
10	6	0	1.270361	1.898321	1.301335
11	1	0	2.362999	1.918756	1.286369
12	1	0	0.903495	2.920506	1.416946
13	1	0	0.934794	1.296996	2.150203
14	7	0	-1.179528	0.009106	-0.010401
15	7	0	1.012502	-0.113968	-0.145587
16	35	0	-2.925437	-0.545603	0.011762
17	35	0	2.694491	-0.834722	0.015617

DBDMH-TEMPO$^+$ (in MeCN)

Sum of electronic and zero-point Energies= -6079.828867

Center Number	Atomic Number	Atomic Type	Coordinates (Angstroms) X	Y	Z
1	6	0	-1.939576	-1.210765	0.213852
2	6	0	-2.935970	0.926231	-0.163920
3	8	0	-3.723286	1.827468	-0.317370
4	6	0	-0.866978	-0.126437	0.078774
5	8	0	0.329652	-0.288989	0.156385
6	7	0	-1.519258	1.057512	-0.144438
7	7	0	3.124630	0.054344	-0.360327
8	8	0	2.458793	0.771379	-1.023697
9	6	0	3.528951	-1.298470	-0.936071
10	6	0	3.614187	0.565417	0.990217
11	6	0	5.068754	-1.351971	-0.830770
12	1	0	5.356347	-2.366768	-1.122560
13	1	0	5.489216	-0.665881	-1.575576
14	6	0	5.148745	0.394416	0.968523
15	1	0	5.495500	0.642909	1.976324
16	1	0	5.570700	1.140283	0.284765
17	6	0	5.603283	-1.001262	0.554477
18	1	0	6.695810	-1.024268	0.527096
19	1	0	5.299627	-1.748907	1.293824
20	6	0	2.839615	-2.416633	-0.134168
21	1	0	1.788887	-2.182111	0.050412
22	1	0	2.891505	-3.311504	-0.758678
23	1	0	3.338040	-2.640095	0.806502
24	6	0	3.060446	-1.331631	-2.383154
25	1	0	3.431210	-2.261102	-2.819867

26	1	0	1.969442	−1.325987	−2.447048
27	1	0	3.458335	−0.492704	−2.958028
28	6	0	3.205571	2.026582	1.096079
29	1	0	2.118017	2.128092	1.134918
30	1	0	3.627532	2.413984	2.025656
31	1	0	3.591534	2.618784	0.263528
32	6	0	2.945312	−0.249331	2.111050
33	1	0	3.091206	0.325040	3.028852
34	1	0	1.873164	−0.355522	1.935294
35	1	0	3.394412	−1.229056	2.257700
36	7	0	−3.152579	−0.404011	0.021125
37	6	0	−1.869273	−1.827649	1.608710
38	1	0	−2.634868	−2.601440	1.706198
39	1	0	−0.887839	−2.287856	1.745952
40	1	0	−2.023930	−1.070223	2.381030
41	6	0	−1.768262	−2.245842	−0.895625
42	1	0	−0.801469	−2.741648	−0.781574
43	1	0	−2.558225	−2.997421	−0.816148
44	1	0	−1.819403	−1.774461	−1.880021
45	35	0	−0.674143	2.663921	−0.387163
46	35	0	−4.847411	−1.085117	0.185349

(j) Proposed mechanism for the formation of bromide anions

Procedure for eq 1: substrate **S30** (81.1 mg, 0.50 mmol), DBDMH (214 mg, 0.75 mmol), TEMPO (15.6 mg, 0.10 mmol) and DCE (2.0 mL) were added to a reaction tube with a magnetic bar. The mixture was stirred at 60 °C for 20 h, the reaction mixture was quenched with saturated Na_2SO_3 aqueous solution (2.0 mL). The aqueous phase was diluted with water (3 mL) and extracted with EtOAc (5 mL × 3). The combined organic extracts were dried over anhydrous Na_2SO_4, filtered, and concentrated under reduced pressure. The residue was then analyzed by NMR spectrum. Yield and diastereoselectivity was determined by ^{1}H-NMR using 1,1,2,2-tetrachloroethane as internal standard.

Procedure for eq 2: substrate **S30** (81.1 mg, 0.50 mmol), Br_2 (119.8 mg, 0.75 mmol) and DCE (2.0 mL) were added to a reaction tube with a magnetic bar. The mixture was stirred at 60 °C for 1 h, and the post-processing is the same as above.

Procedure for eq 3: DBDMH (214 mg, 0.75 mmol), TEMPO (15.6 mg, 0.10 mmol) and DCE (2.0 mL) were added to a reaction tube with a magnetic bar. The mixture was stirred at 60 °C for 20 h, then the substrate **S30** (81.1 mg, 0.50 mmol) was added and stirred for another 1 h. The post-processing is the same as above.

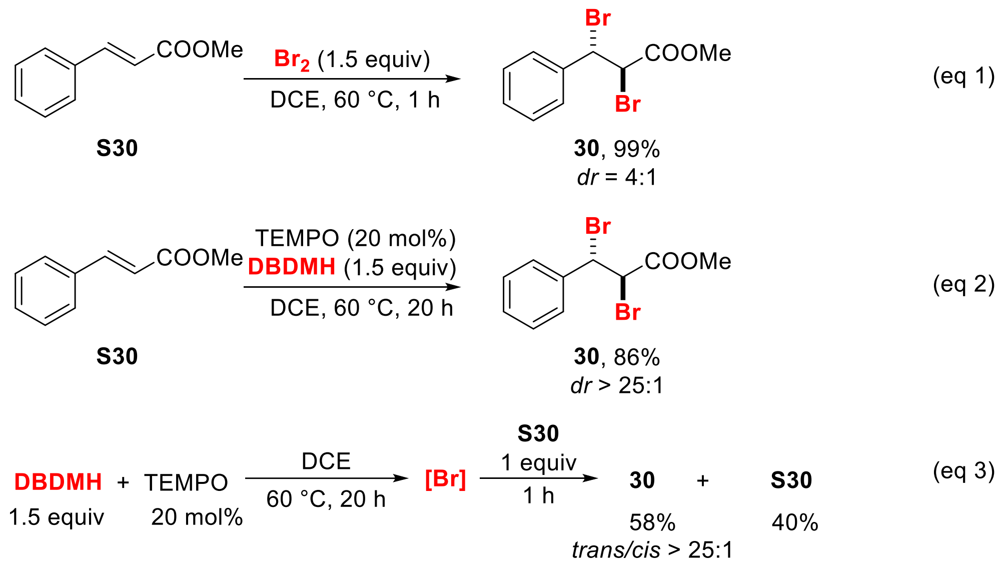

Supplementary Figure 11. Mechanistic studies of dibromination reaction.

Procedure for NMR analysis: TEMPO (0.1 mmol) and DBDMH (0.1 mmol) were dissolved in CD_3CN (1 mL). The solution was then monitored by ^{1}H-NMR.

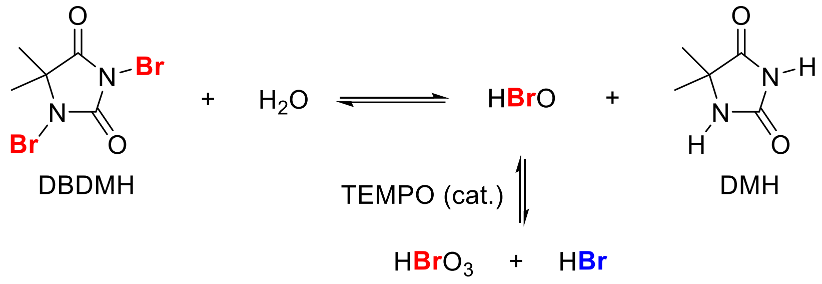

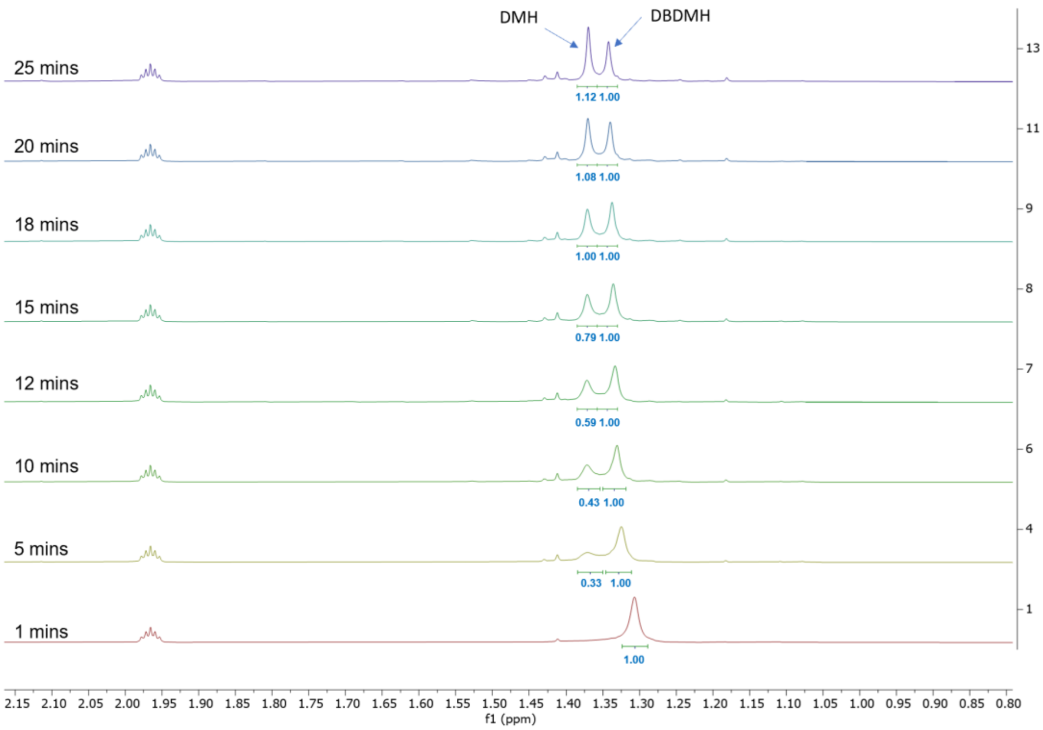

Supplementary Figure 12. NMR analysis of reaction between DBDMH and TEMPO.

The formation of bromide anions is attributed to trace amount of water in the reaction system. The hydrolysis of DBDMH was often assumed to be the *in situ* formation of hypobromites and the dimethylhydantoin. Hypobromic acid could undergo further disproportionation reaction to form HBr and $HBrO_3$[26]. TEMPO might catalyze the disproportionation reaction to facilitate the formation of HBr. Therefore, in this case, TEMPO not only functions as an activator of haleniums, but a redox catalyst to promote the formation of bromide anions. To testify our hypothesis, we conducted some mechanistic experiments. Initially, we compared the dibromination of **S30** with Br_2 and our optimized condition. Although Br_2 is highly reactive and completes the reaction in 1 hour, it is not selective (*dr* = 4:1, eq 1). In contrast, the reaction completed in 20 hours with excellent diastereoselectivity under our condition (*dr* > 25:1, eq 2). These results suggest that Br_2 is not the brominating reagent in the reaction system. Besides, if DBDMH and TEMPO were stirred without the addition of **S30** for 20 hours, the bromide species could also achieve the dibromination of **S30** with lower yield and excellent diastereoselectivity (*dr* > 25:1), perhaps due to the decomposition of DBDMH as shown above. Furthermore, we monitored the reaction

mixture of DBDMH and TEMPO in CD_3CN (reaction ratio = 1:0.2), the results clearly implied that TEMPO could facilitate the decomposition of DBDMH.

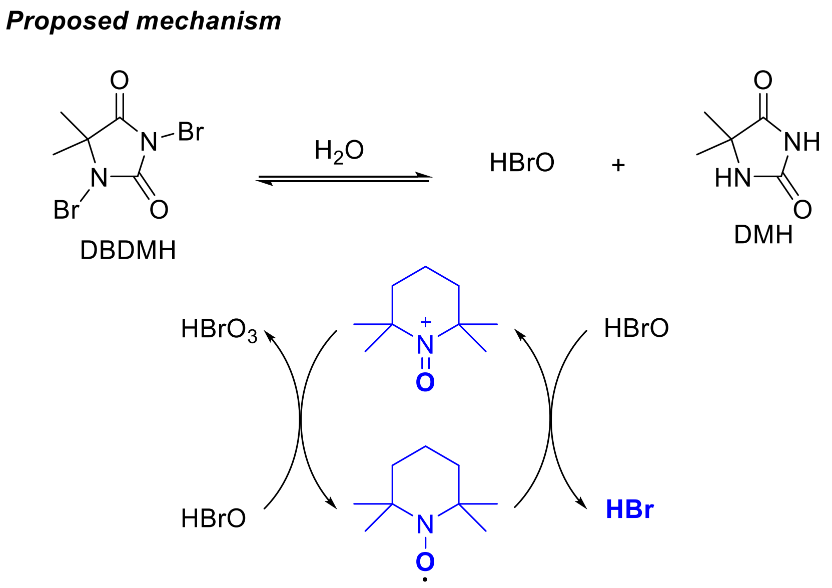

Supplementary Figure 13. Proposed mechanism for the formation of bromide anions.

(E) Supplementary References

1. Chan, Y. C. & Yeung, Y. Y. Halogen Bond Catalysed Bromocarbocyclization. *Angew. Chem. Int. Ed.* **57**, 3483-3487 (2018).
2. Frlan, R., Sova, M., Gobec, S., Stavber, G. & Casar, Z. Cobalt-Catalysed Cross-Coupling of Grignards with Allylic and Vinylic Bromides: Use of Sarcosine as a Natural Ligand. *J. Org. Chem.* **80**, 7803-7809 (2015).
3. Hernandez-Torres, G., Tan, B. & Barbas, C. F. Organocatalysis as a safe practical method for the stereospecific dibromination of unsaturated compounds. *Org. Lett.* **14**, 1858-1861 (2012).
4. Wang, G.-W. & Gao, J. Solvent-free bromination reactions with sodium bromide and oxone promoted by mechanical milling. *Green. Chem.* **14**, 1125-1131 (2012).
5. Yu, T. Y., Wang, Y., Hu, X. Q. & Xu, P. F. Triphenylphosphine oxide-catalysed stereoselective poly- and dibromination of unsaturated compounds. *Chem. Commun.* **50**, 7817-7820 (2014).
6. Malanga, C., Mannucci, S. & Lardicci, L. Carbon-halogen bond activation by nickel catalyst: Synthesis of alkenes, from 1,2-dihalides. *Tetrahedron* **54**, 1021-1028 (1998).
7. Song, S., Li, X. W., Sun, X., Yuan, Y. Z. & Jiao, N. Efficient bromination of olefins, alkynes, and ketones with dimethyl sulfoxide and hydrobromic acid. *Green. Chem.* **17**, 3285-3289 (2015).
8. LaLonde, R. T. & Debboli, A.
9. D. Electrophilic addition of bromine to arylcyclopropanes. Kinetics and mechanistic implications. *J. Org. Chem.* **38**, 4228-4232 (1973).
10. Xiang, J. *et al.* Method for transforming alkynes into (E)-dibromoalkenes. *J. Org. Chem.* **79**, 11378-11382 (2014).
11. Rej, S., Pramanik, S., Tsurugi, H. & Mashima, K. Dehalogenation of vicinal dihalo compounds by 1,1'-bis(trimethylsilyl)-1H,1'H-4,4'-bipyridinylidene for giving alkenes and alkynes in a salt-free manner. *Chem. Commun.* **53**, 13157-13160 (2017).
12. Rodriguez, R. A. *et al.* Palau'chlor: a practical and reactive chlorinating reagent. *J. Am. Chem. Soc.* **136**, 6908-6911 (2014).
13. Song, S. *et al.* DMSO-catalysed late-stage chlorination of (hetero)arenes. *Nature Catal.* **3**, 107-115 (2020).
14. Liu, Y. *et al.* Structural optimization elaborates novel potent Akt inhibitors with promising anticancer activity. *Eur. J. Med. Chem.* **138**, 543-551 (2017).
15. Samanta, R. C. & Yamamoto, H. Selective Halogenation Using an Aniline Catalyst. *Chem. Eur. J.* **21**, 11976-11979 (2015).
16. Nahide, P. D. *et al.* In Situ Formed IIII-Based Reagent for the Electrophilic ortho-Chlorination of Phenols and Phenol Ethers: The Use of PIFA-$AlCl_3$ System. *Eur. J. Org. Chem.* **2018**, 485-493 (2018).
17. Chittimalla, S. K. & Bandi, C. Unanticipated participation of HCl in nucleophilic chlorination reaction: expedient route to meta chlorophenols. *Tetrahedron Lett.* **57**, 15-19 (2016).
18. Huo, H.-r., Tang, X.-Y. & Gong, Y.-f. Metal-Free Synthesis of Pyrrolo[1,2-a]quinoxalines Mediated by TEMPO Oxoammonium Salts. *Synthesis* **50**, 2727-2740 (2018).

19. M. J. Frisch, G. W. *et al. Gaussian 09*, Revision A.01, Gaussian, Inc., Wallingford CT, 2009.
20. Zhao, Y. & Truhlar, D. G. The M06 suite of density functionals for main group thermochemistry, thermochemical kinetics, noncovalent interactions, excited states, and transition elements: two new functionals and systematic testing of four M06-class functionals and 12 other functionals. *Theor. Chem. Account* **120**, 215-241 (2008).
21. Grimme, S., Antony, J., Ehrlich, S. & Krieg, H. A consistent and accurate *ab initio* parametrization of density functional dispersion correction (DFT-D) for the 94 elements H-Pu. *J. Chem. Phys.* **132**, 154104 (2010).
22. Hay, J. P. & Wadt, W. R. *Ab initio* effective core potentials for molecular calculations. Potentials for K to Au including the outermost core orbitals *J. Chem. Phys.* **82**, 299 (1985).
23. Friesner, R. A. *et al.* Correlated *ab Initio* Electronic Structure Calculations for Large Molecules *J. Phys. Chem. A* **103**, 1913-1928 (1999).
24. Becke, A. D. Density ‐ functional thermochemistry. III. The role of exact exchange. *J. Chem. Phys.* **98**, 5648 (1993).
25. Lee, C., Yang, W. & Parr, R. G. Development of the Colle-Salvetti Correlation-Energy Formula into a Functional of the Electron Density. *Phys. Rev. B*, **37**, 785-789 (1988).
26. Song, S., Liu, P. & Song, Q. J. Quantification of Dibromodimethylhydantoin Disinfectants in Water by Chemiluminescent Method. *Anal. Sci.* **23**, 327-330 (2007).

(F) NMR Spectra of Compounds

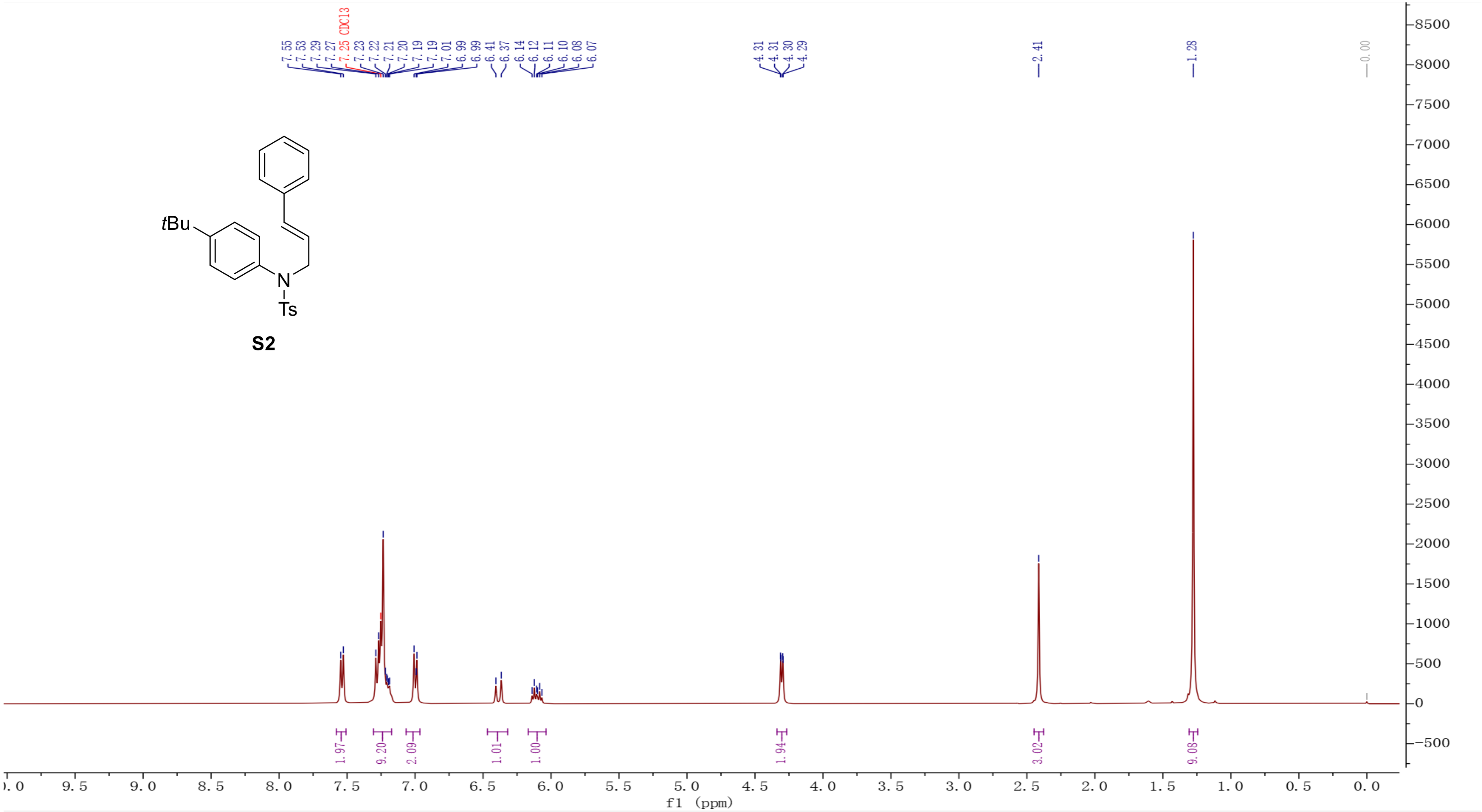

Supplementary Figure 14. ^{1}H NMR spectra of compound **S2**.

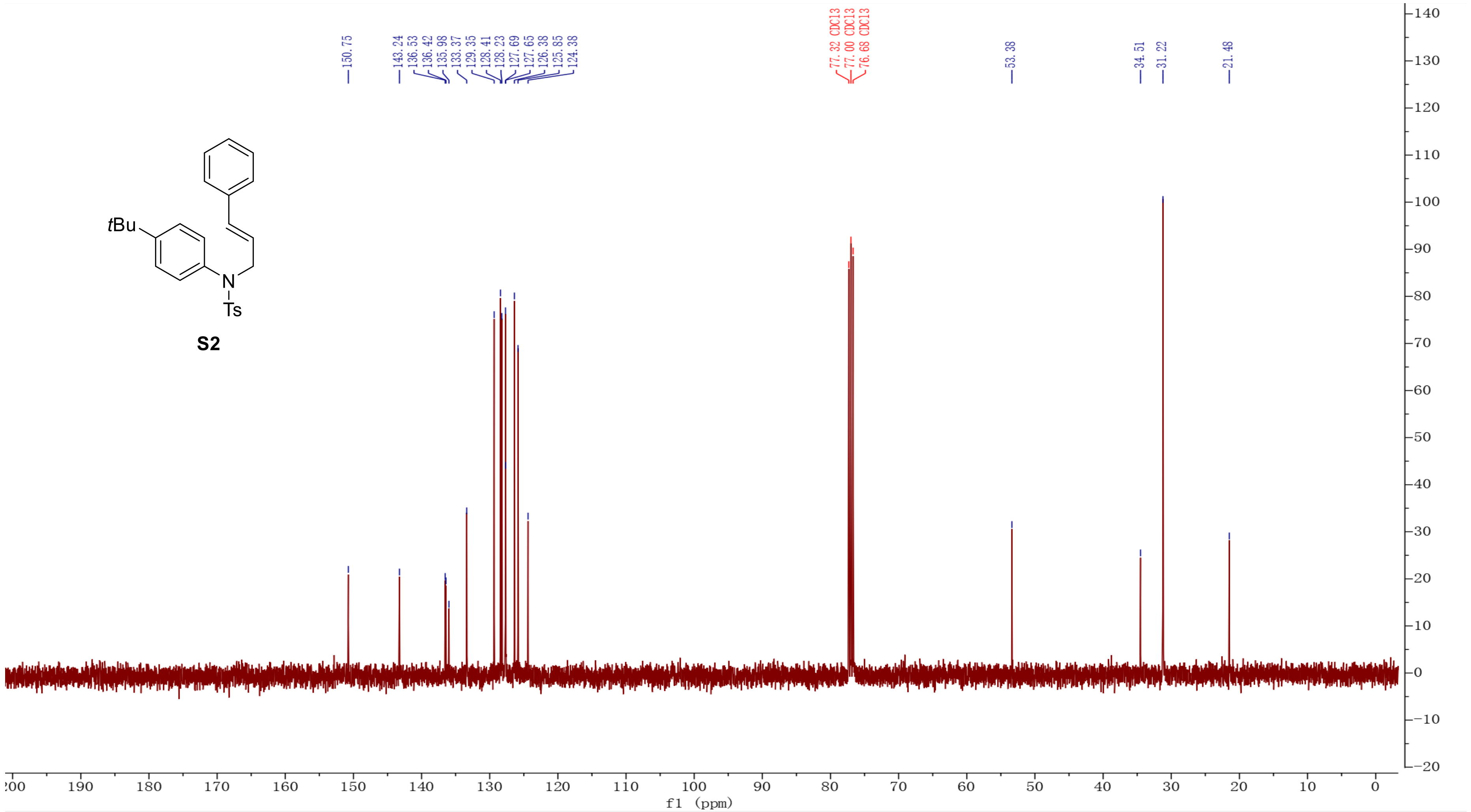

Supplementary Figure 15. ^{13}C NMR spectra of compound **S2**.

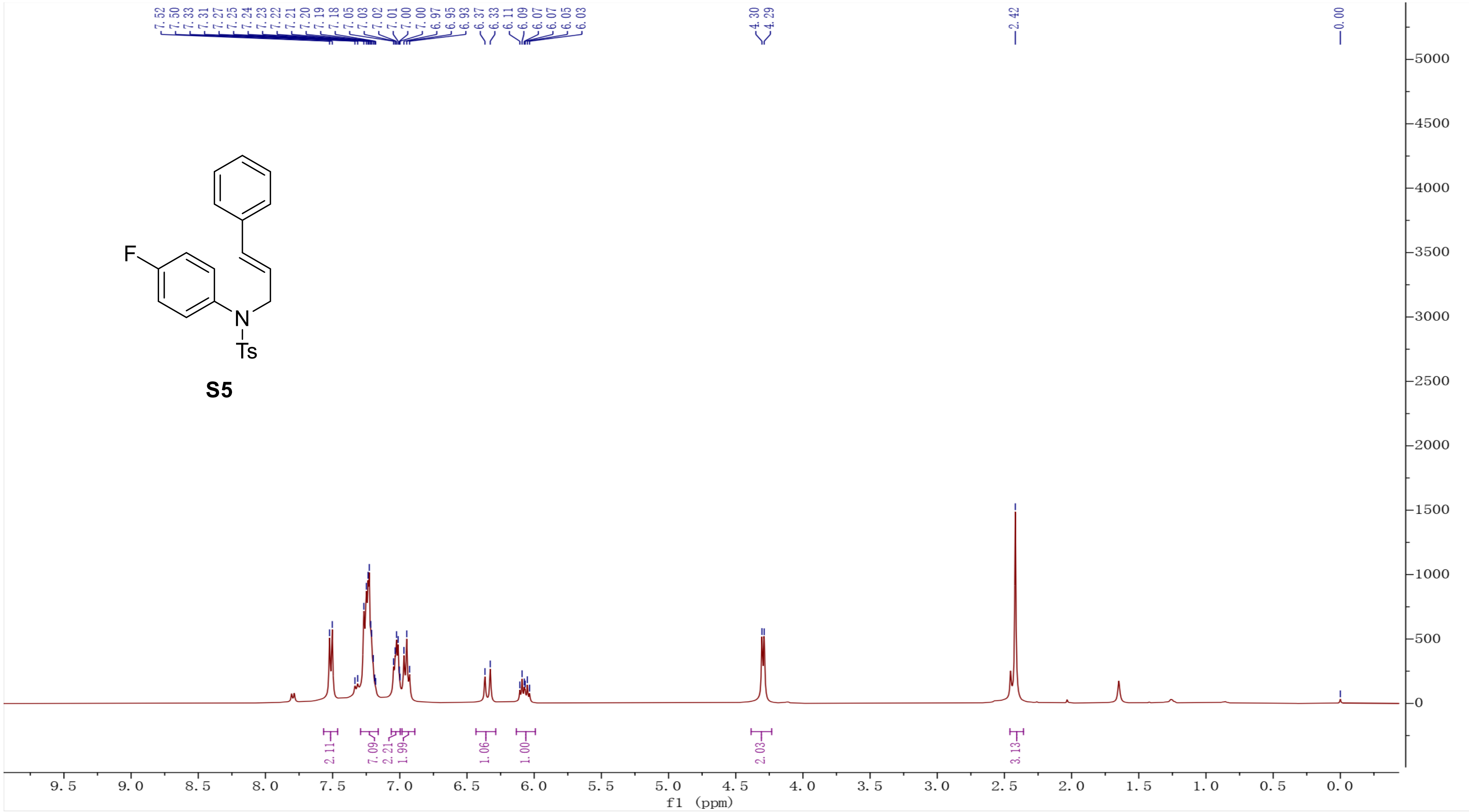

Supplementary Figure 16. ^{1}H NMR spectra of compound **S5**.

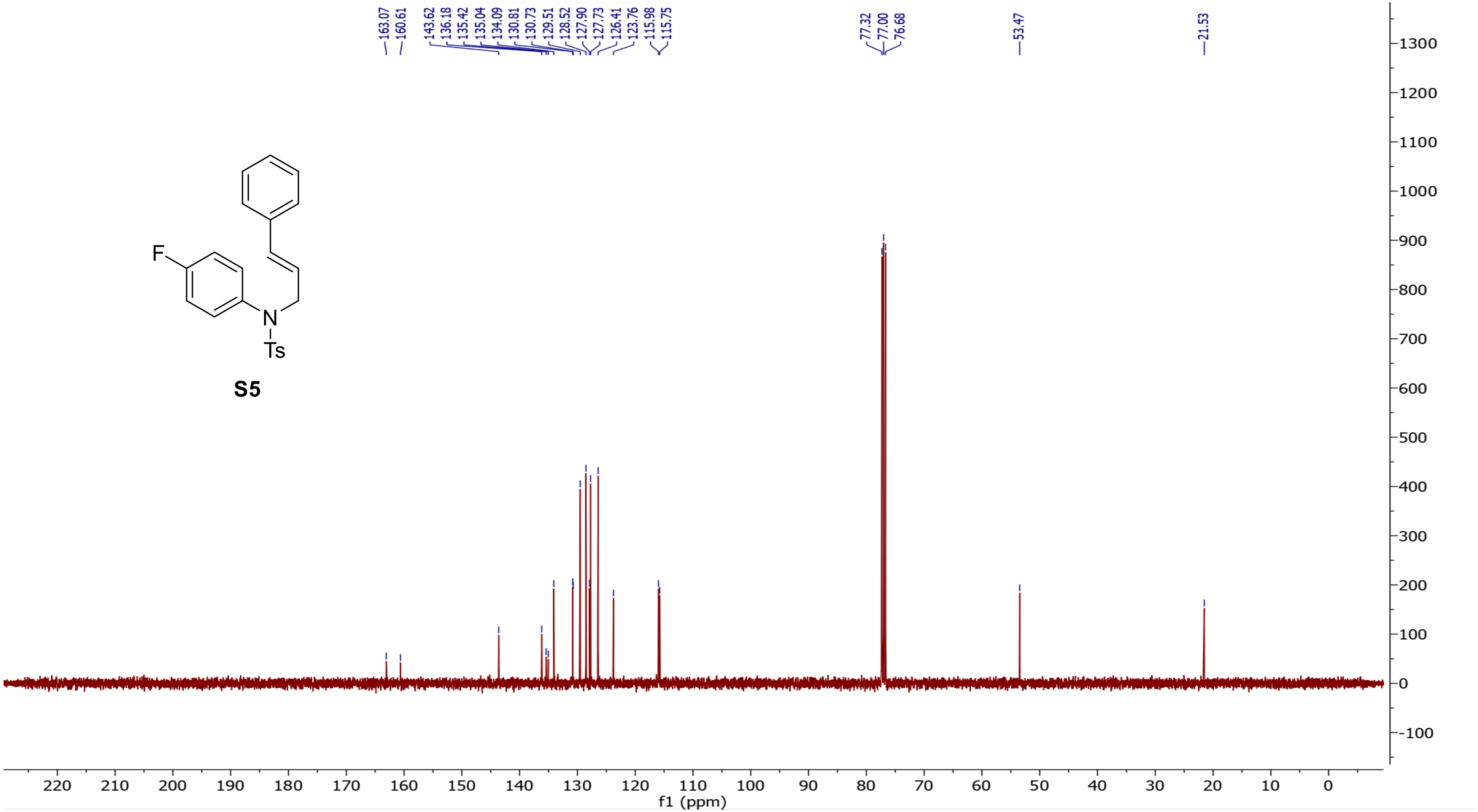

Supplementary Figure 17. ^{13}C NMR spectra of compound **S5**.

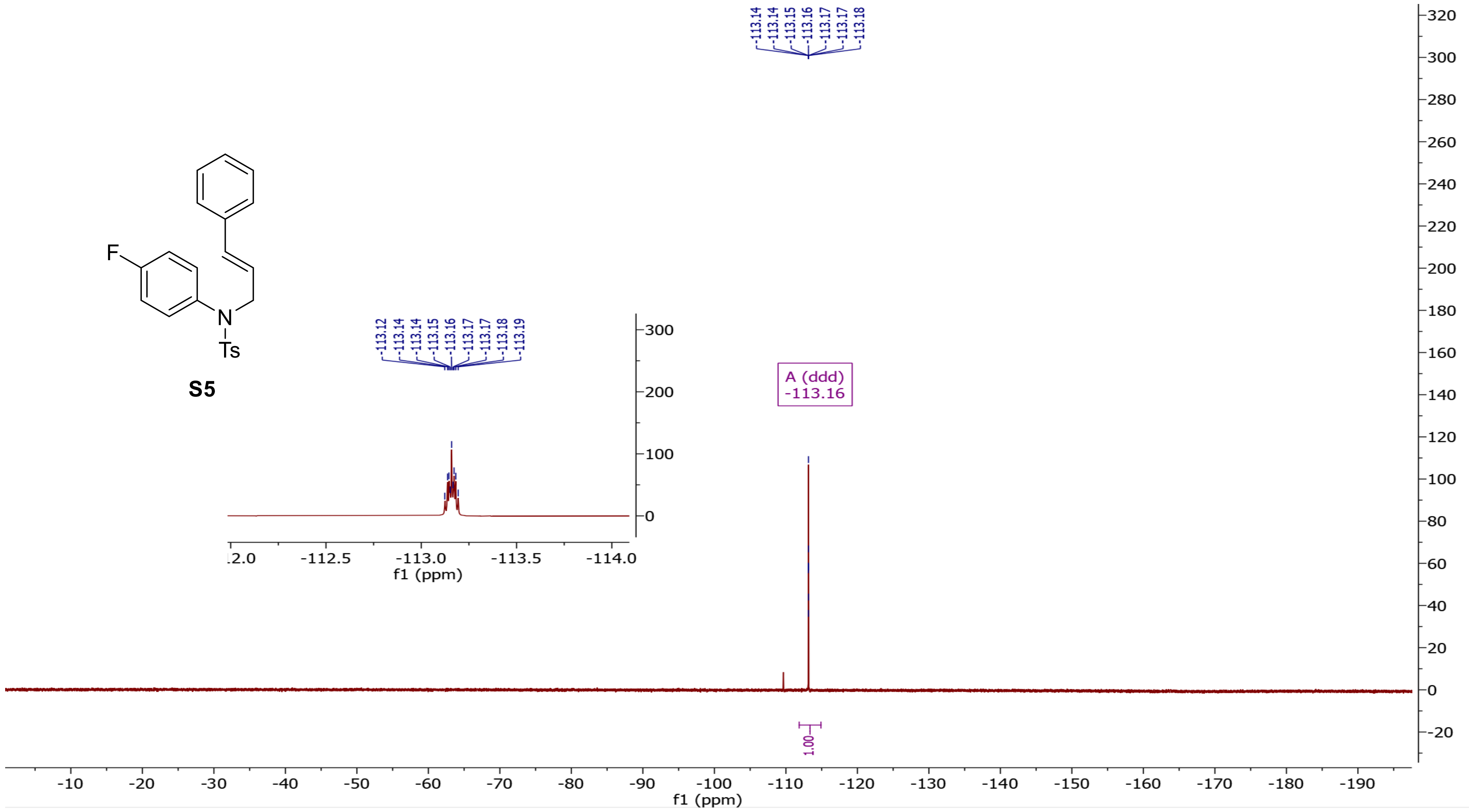

Supplementary Figure 18. ^{19}F NMR spectra of compound **S5**.

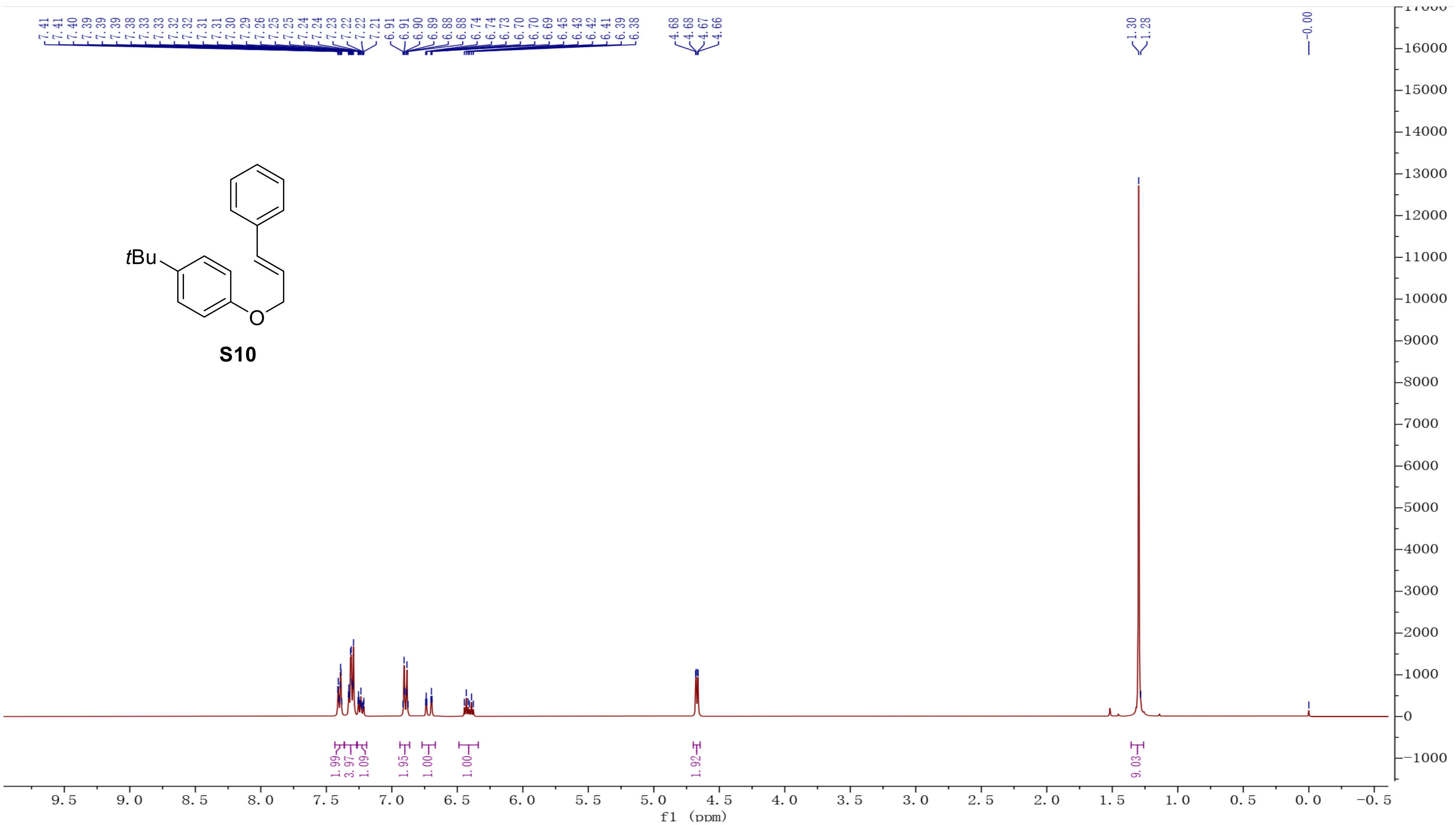

Supplementary Figure 19. ^{1}H NMR spectra of compound **S10**.

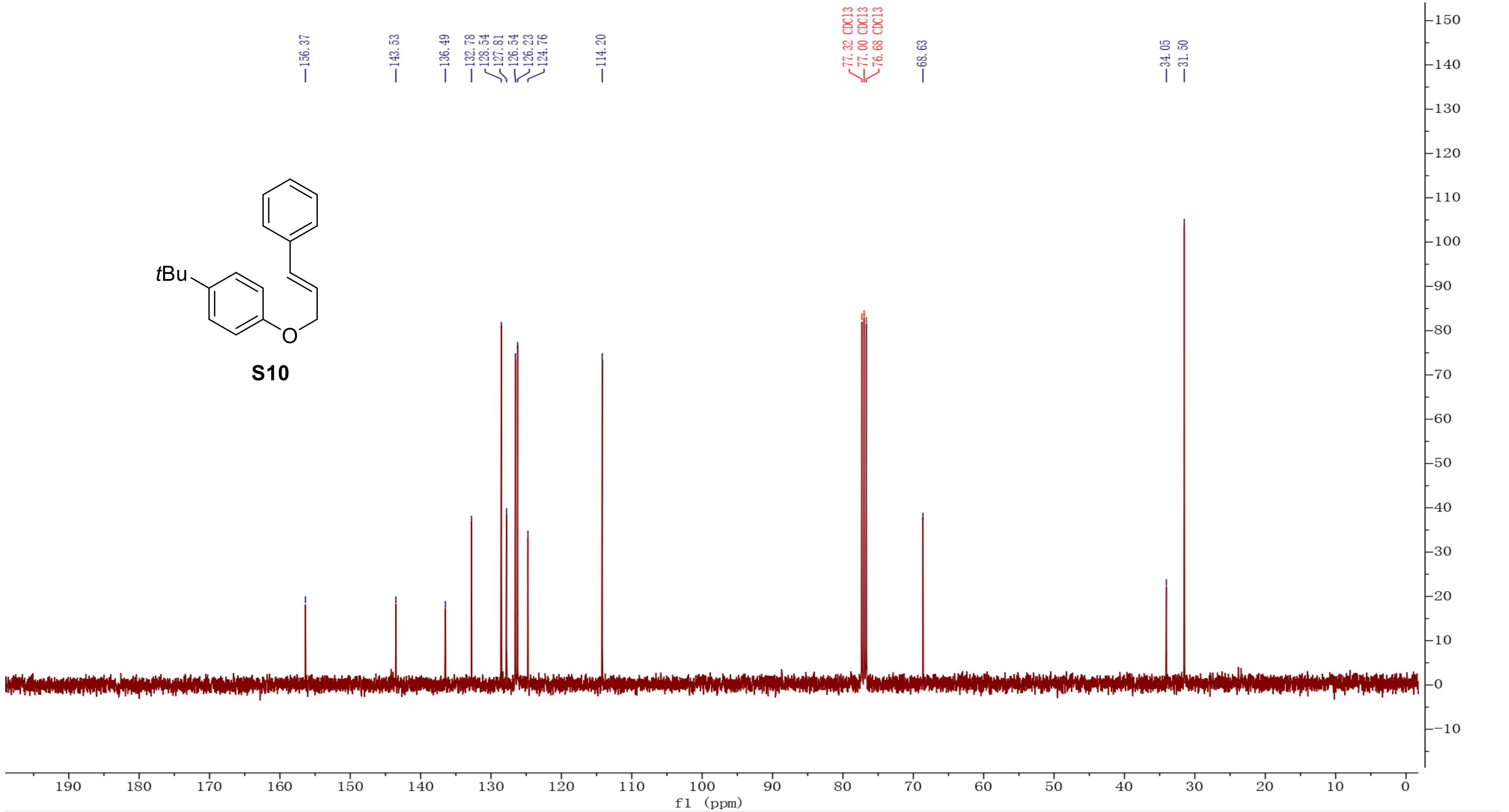

Supplementary Figure 20. ^{13}C NMR spectra of compound **S10**.

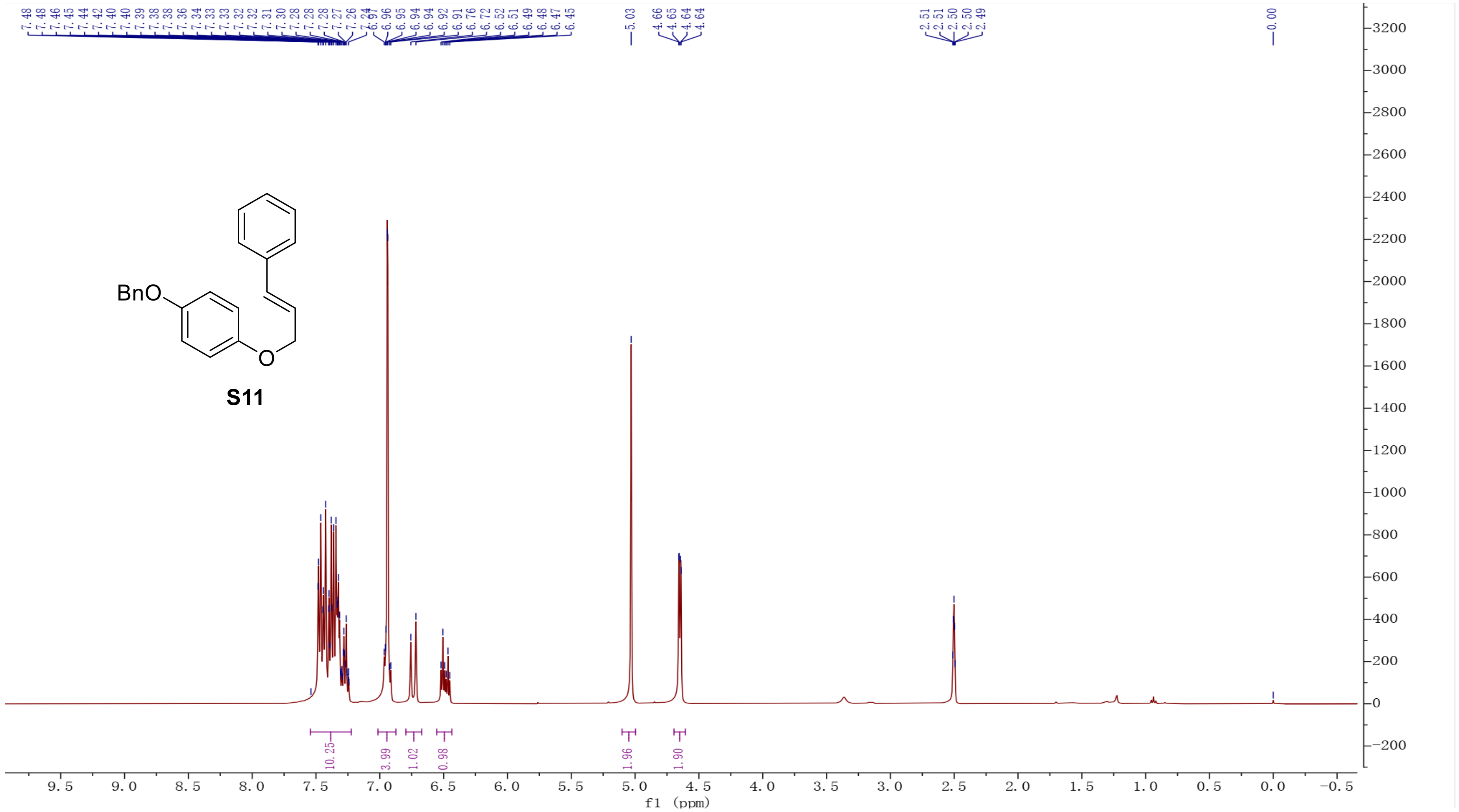

Supplementary Figure 21. ^{1}H NMR spectra of compound **S11**.

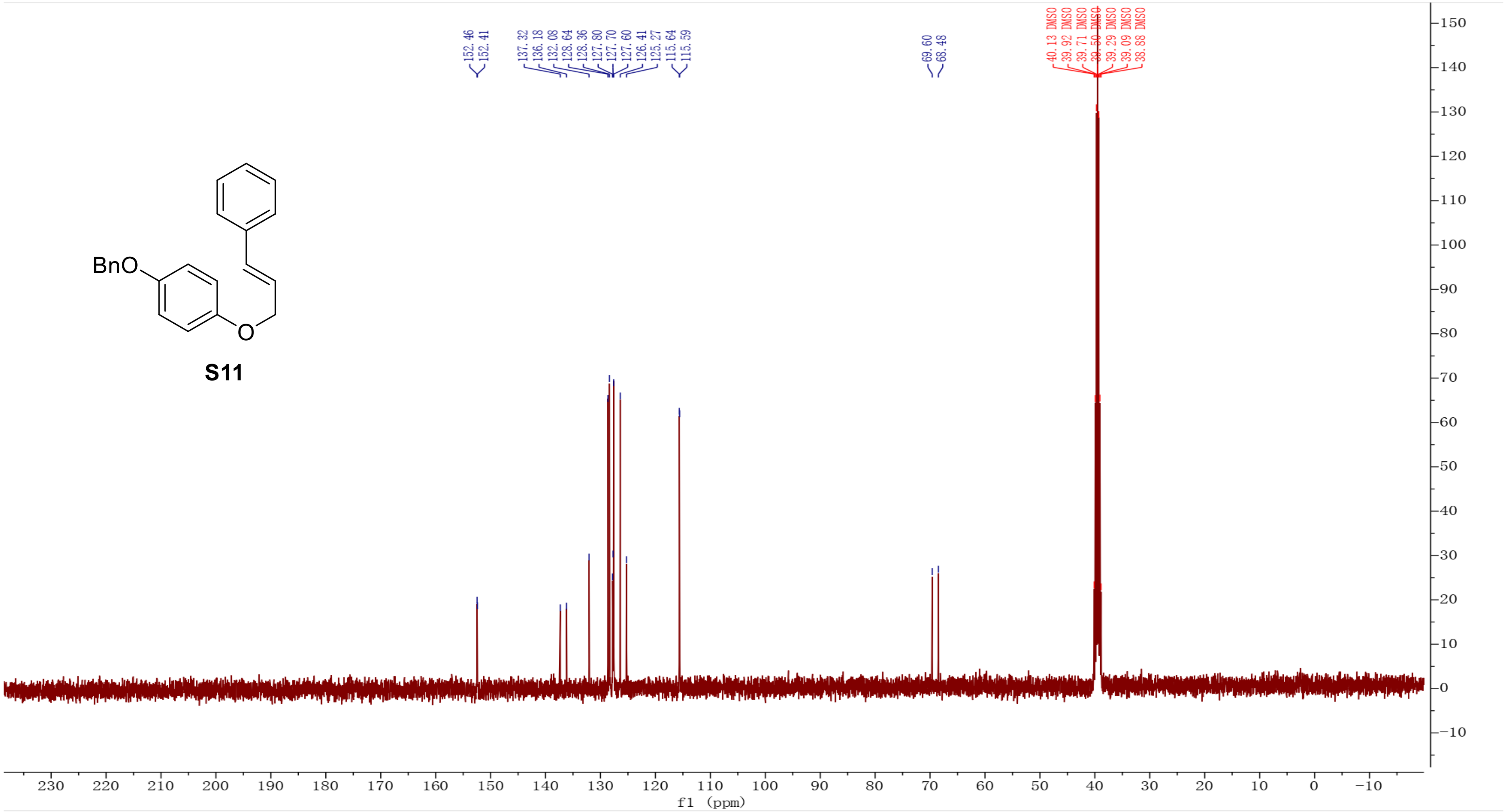

Supplementary Figure 22. ^{13}C NMR spectra of compound **S11**.

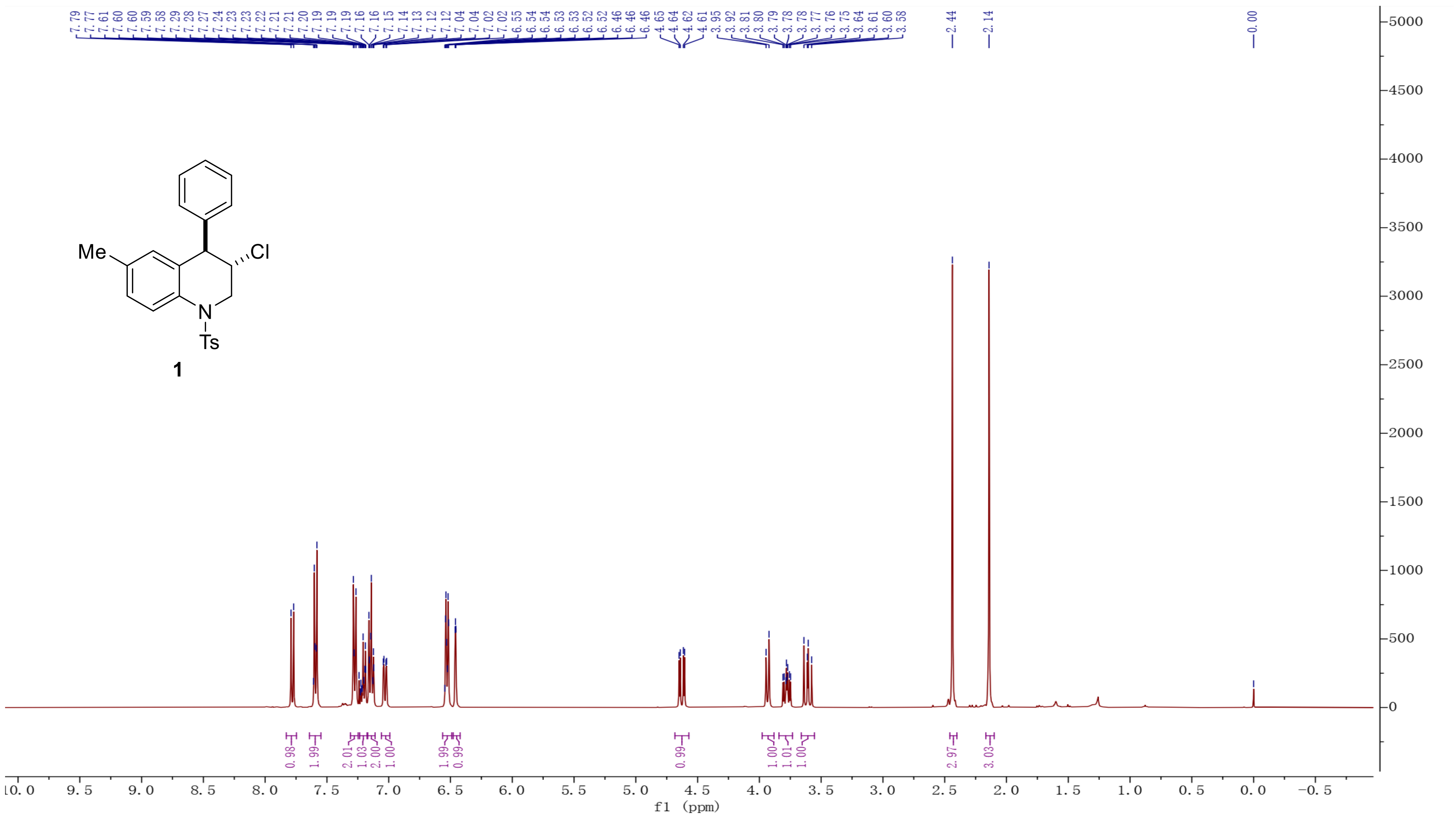

Supplementary Figure 23. ^{1}H NMR spectra of compound **1**.

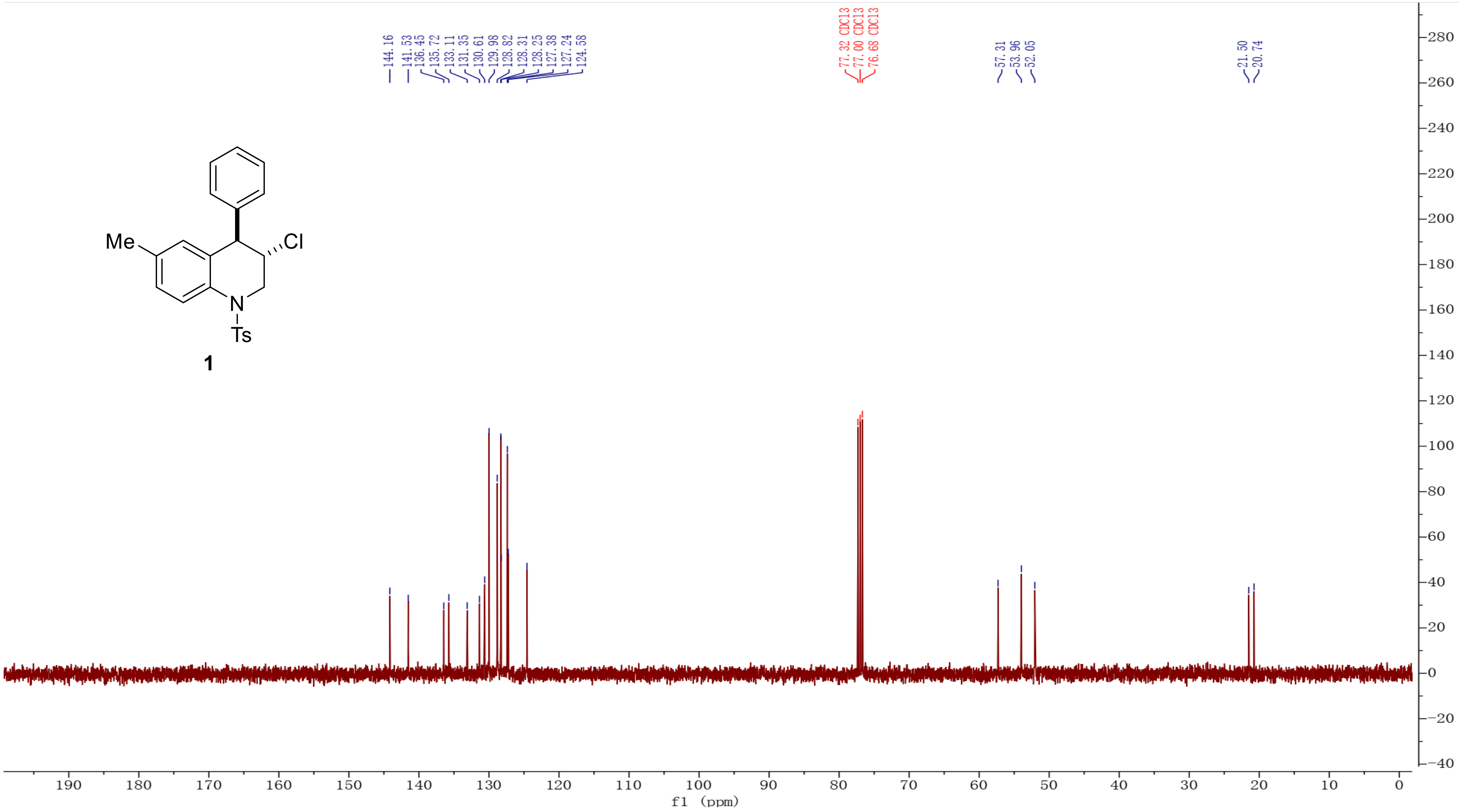

Supplementary Figure 24. ^{13}C NMR spectra of compound **1**.

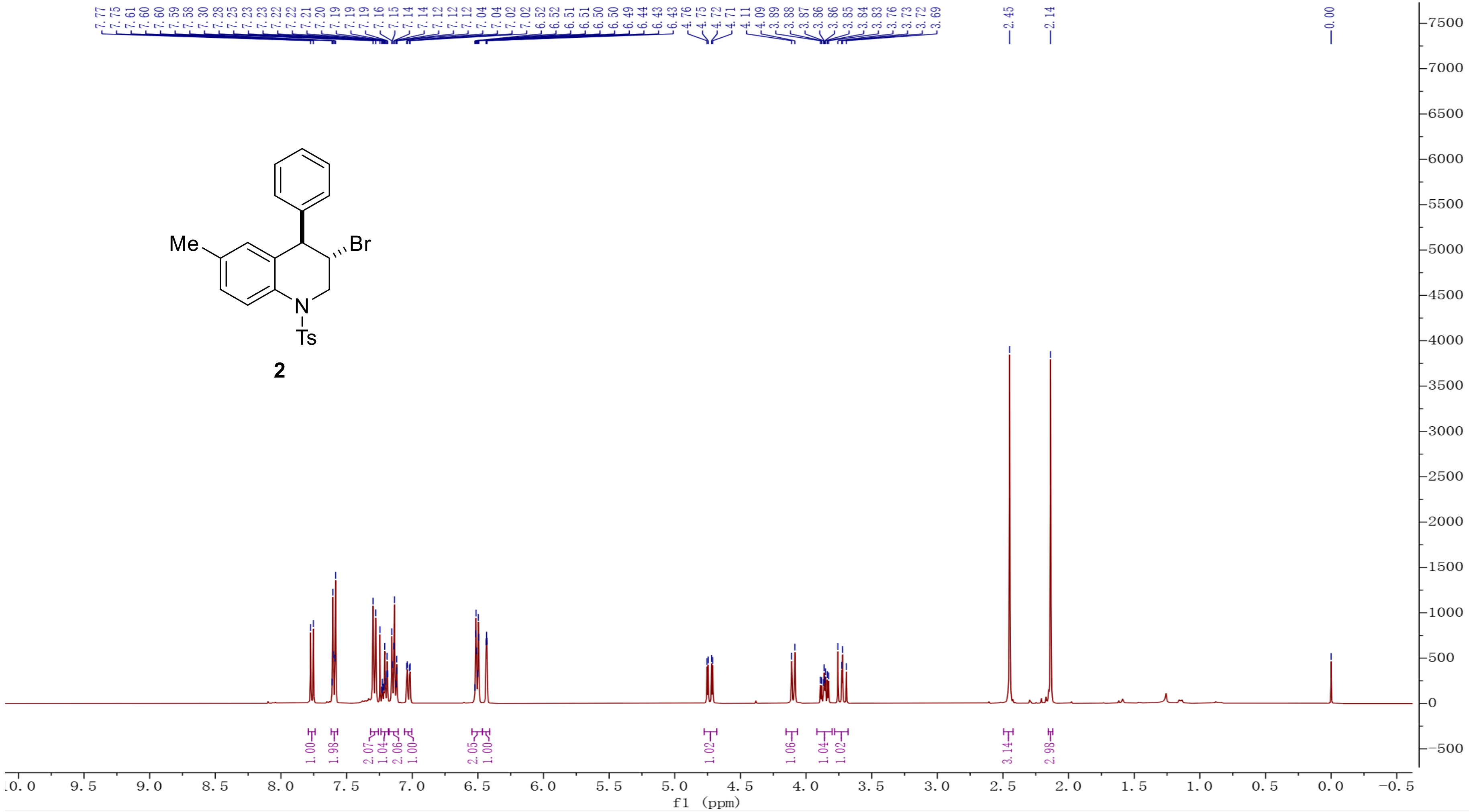

Supplementary Figure 25. ^{1}H NMR spectra of compound **2**.

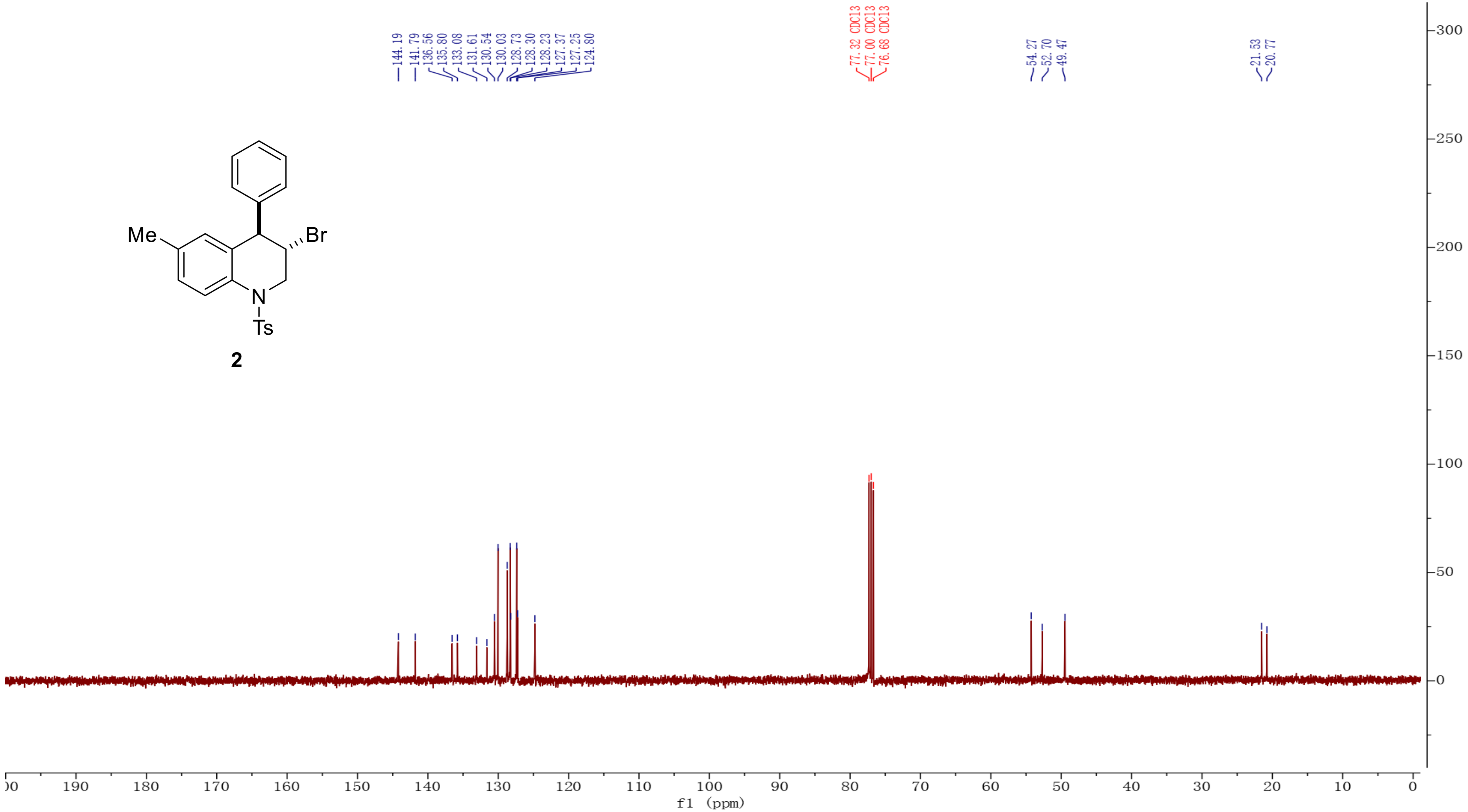

Supplementary Figure 26. ^{13}C NMR spectra of compound **2**.

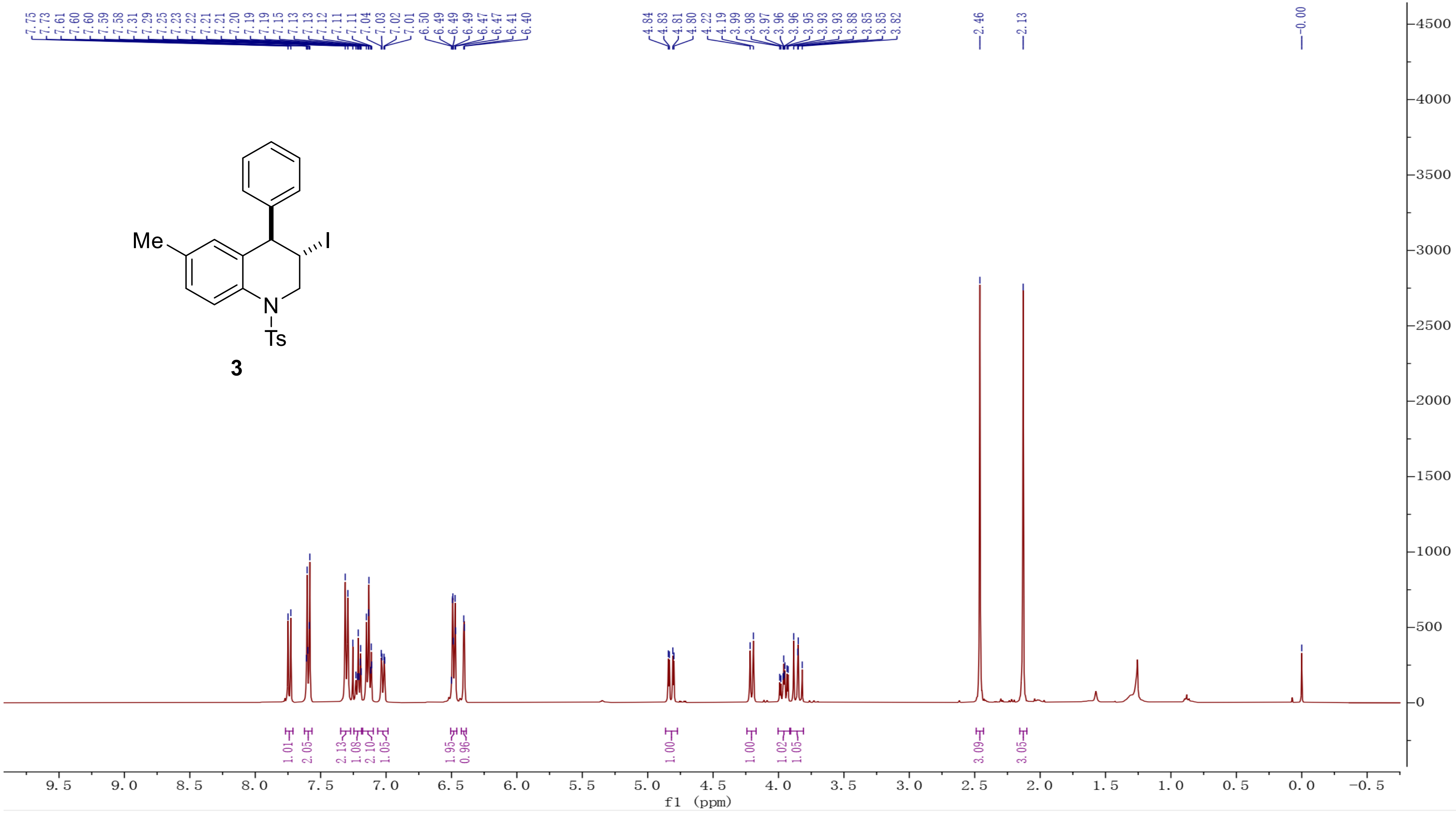

Supplementary Figure 27. ^{1}H NMR spectra of compound **3**.

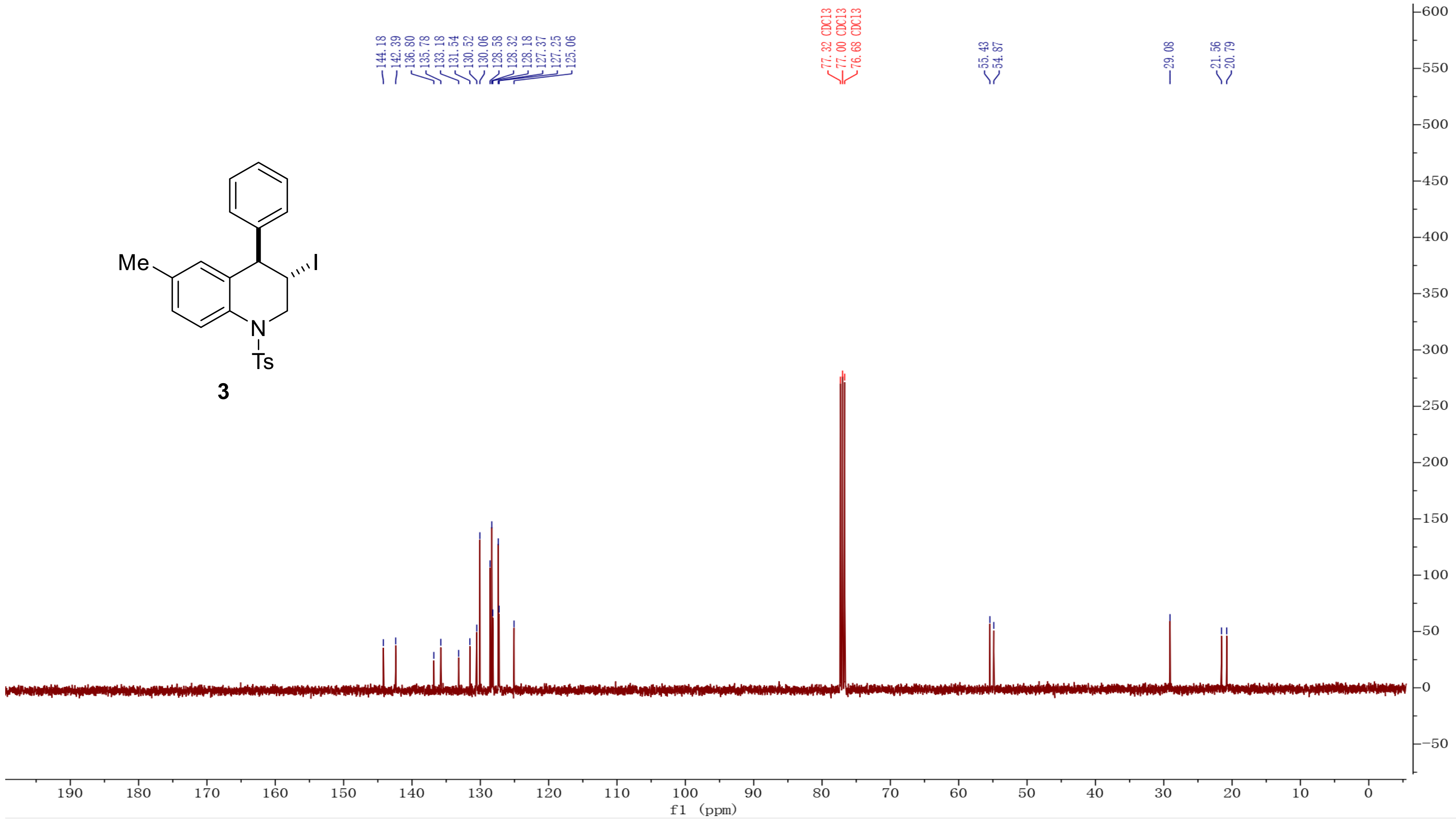

Supplementary Figure 28. ^{13}C NMR spectra of compound **3**.

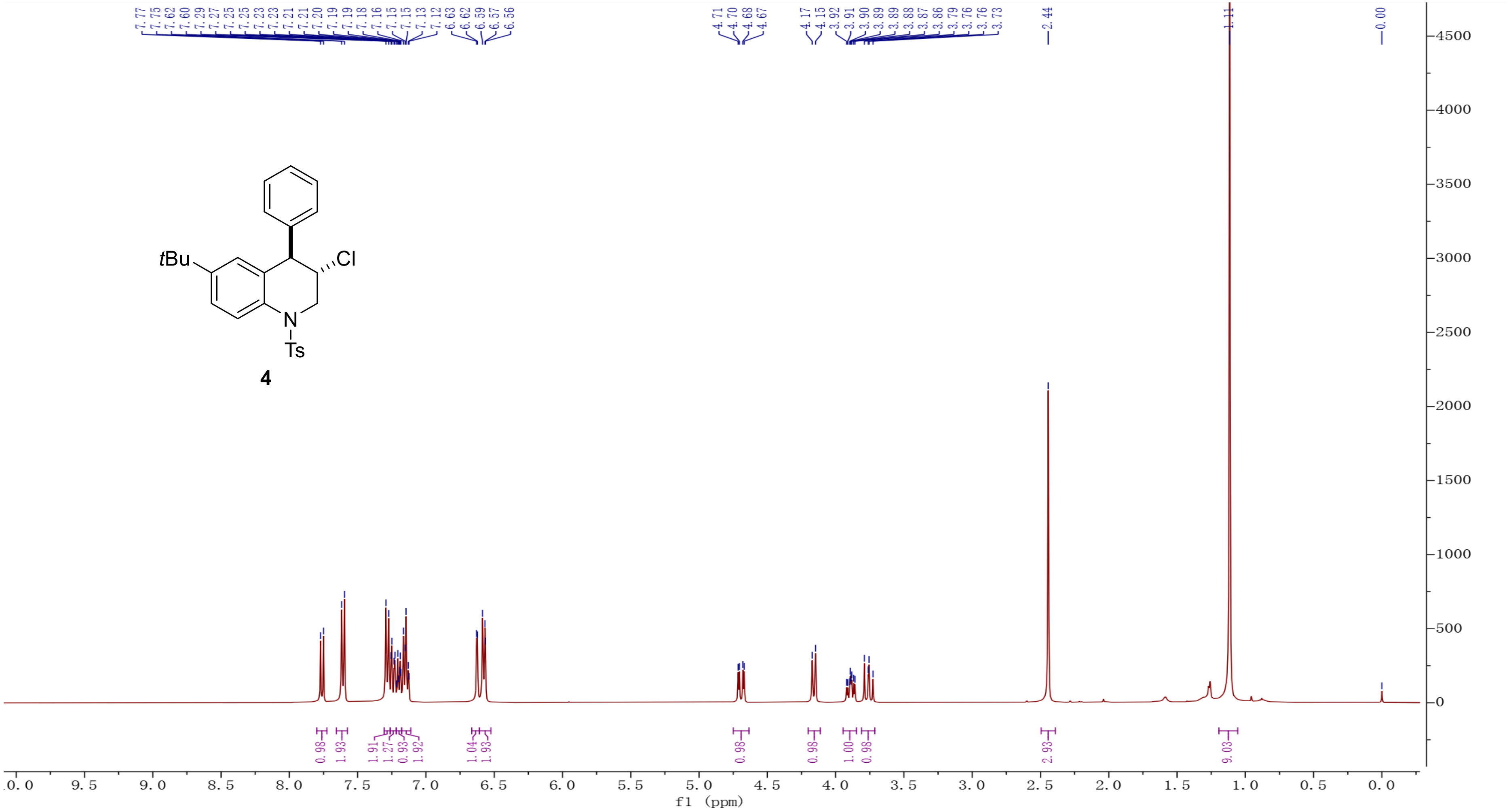

Supplementary Figure 29. ^{1}H NMR spectra of compound **4**.

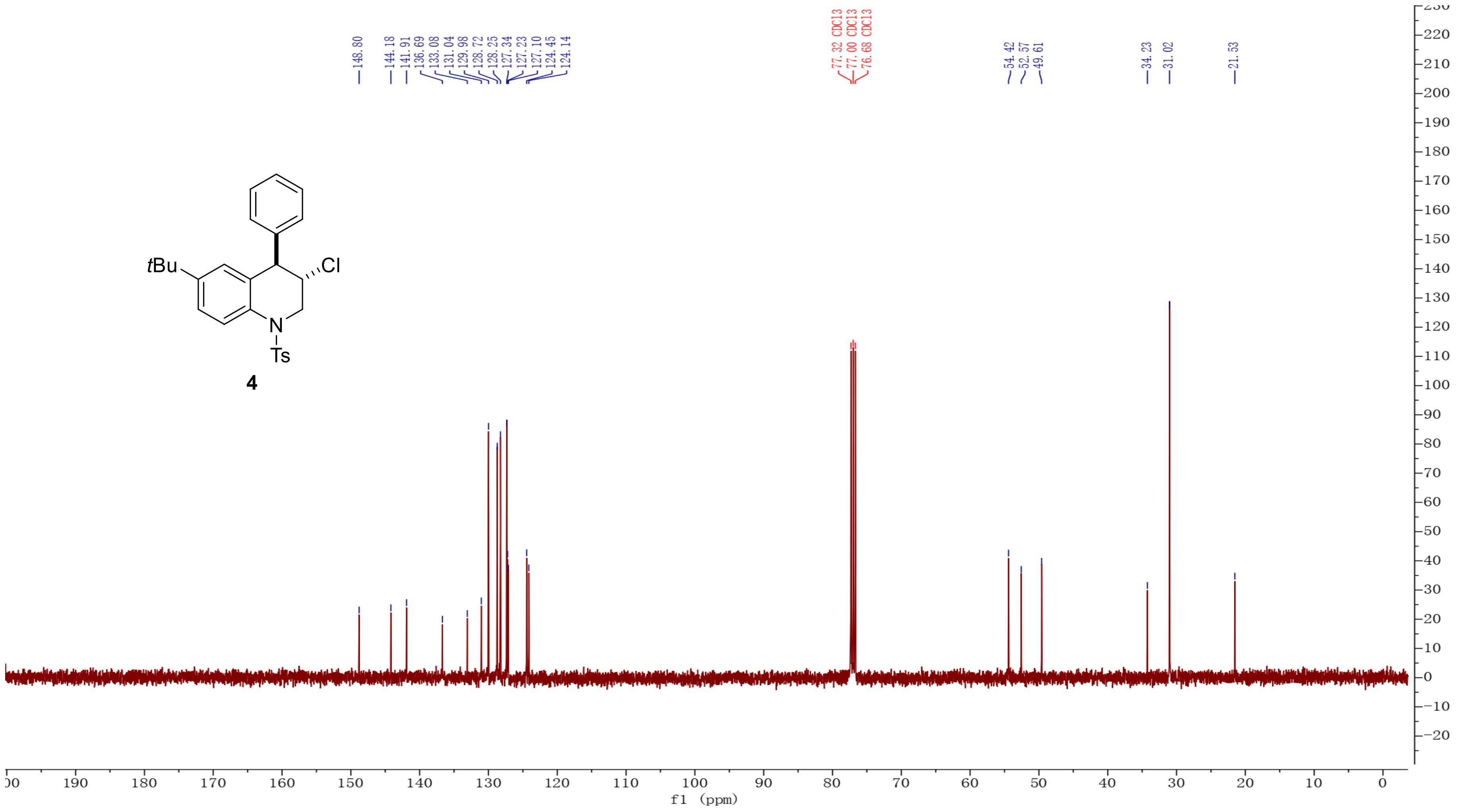

Supplementary Figure 30. ^{13}C NMR spectra of compound **4**.

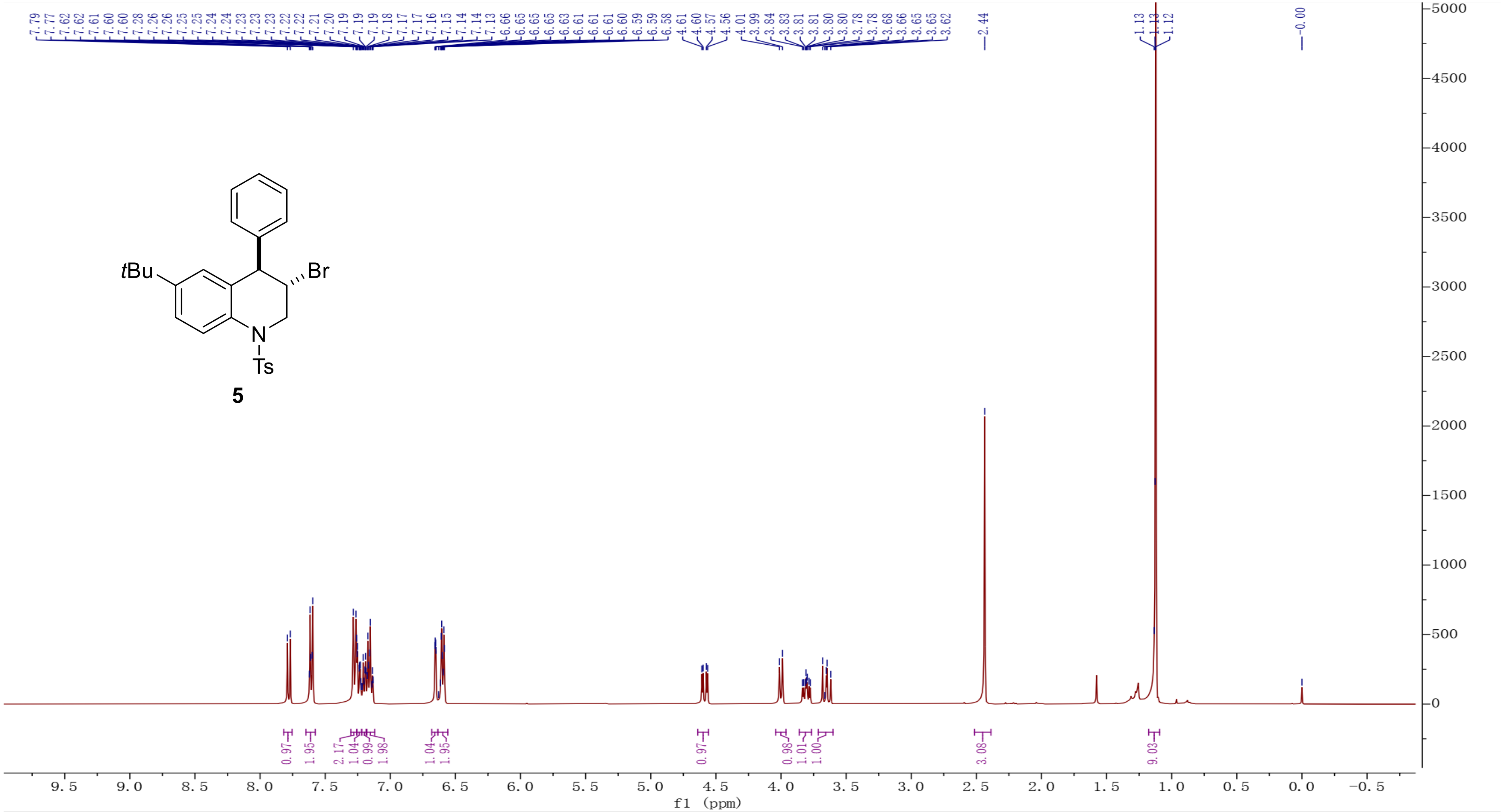

Supplementary Figure 31. ^{1}H NMR spectra of compound **5**.

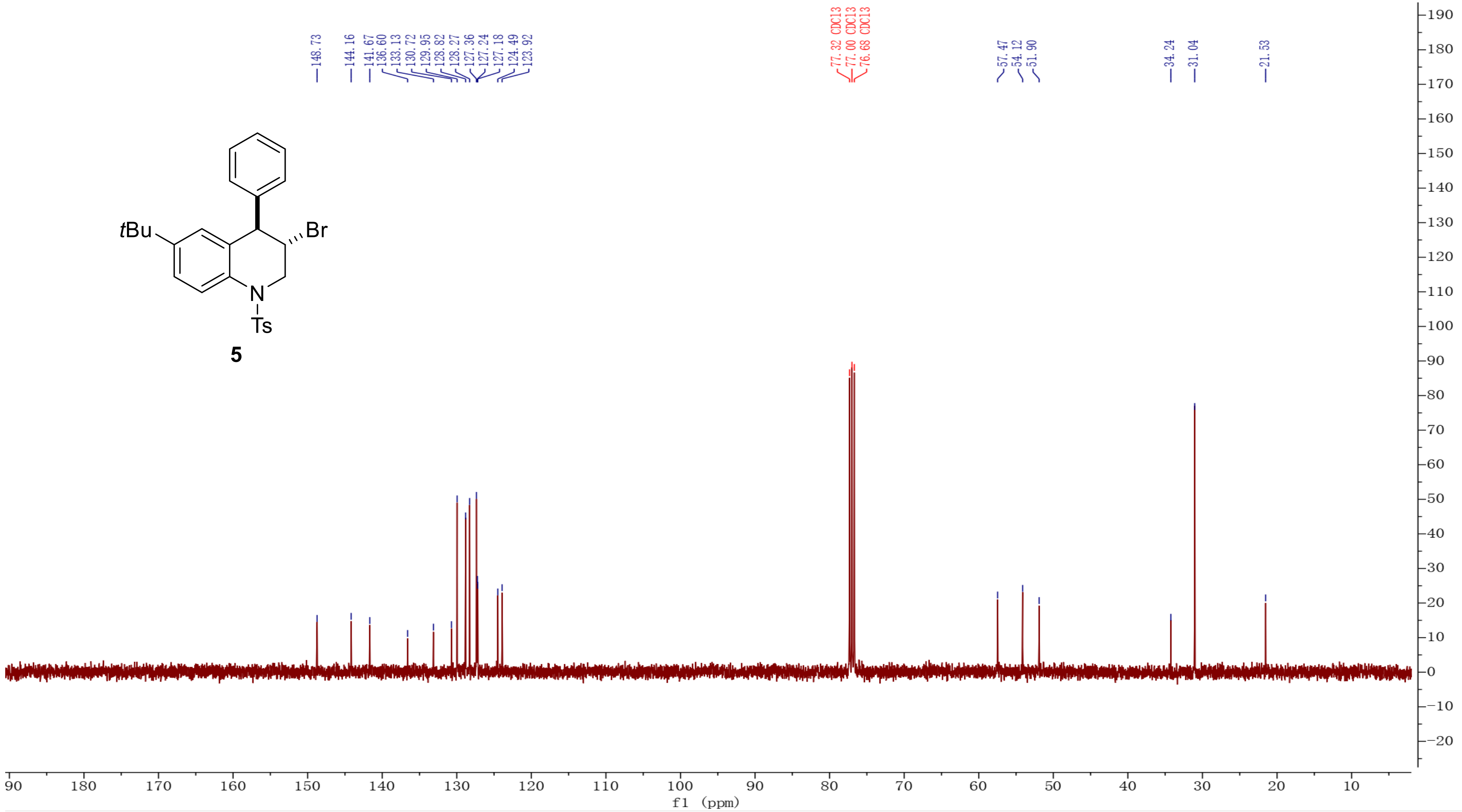

Supplementary Figure 32. ^{13}C NMR spectra of compound **5**.

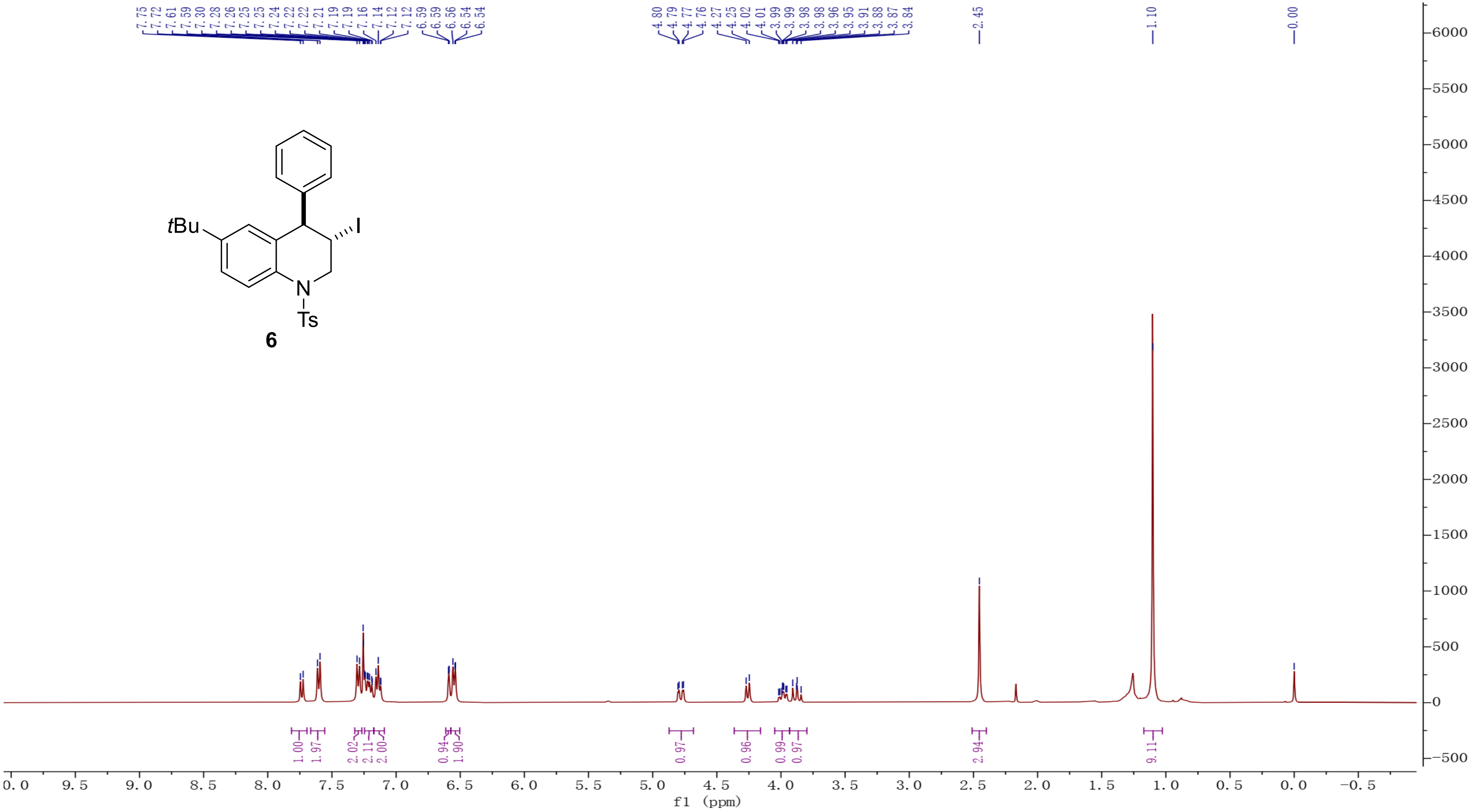

Supplementary Figure 33. ^{1}H NMR spectra of compound **6**.

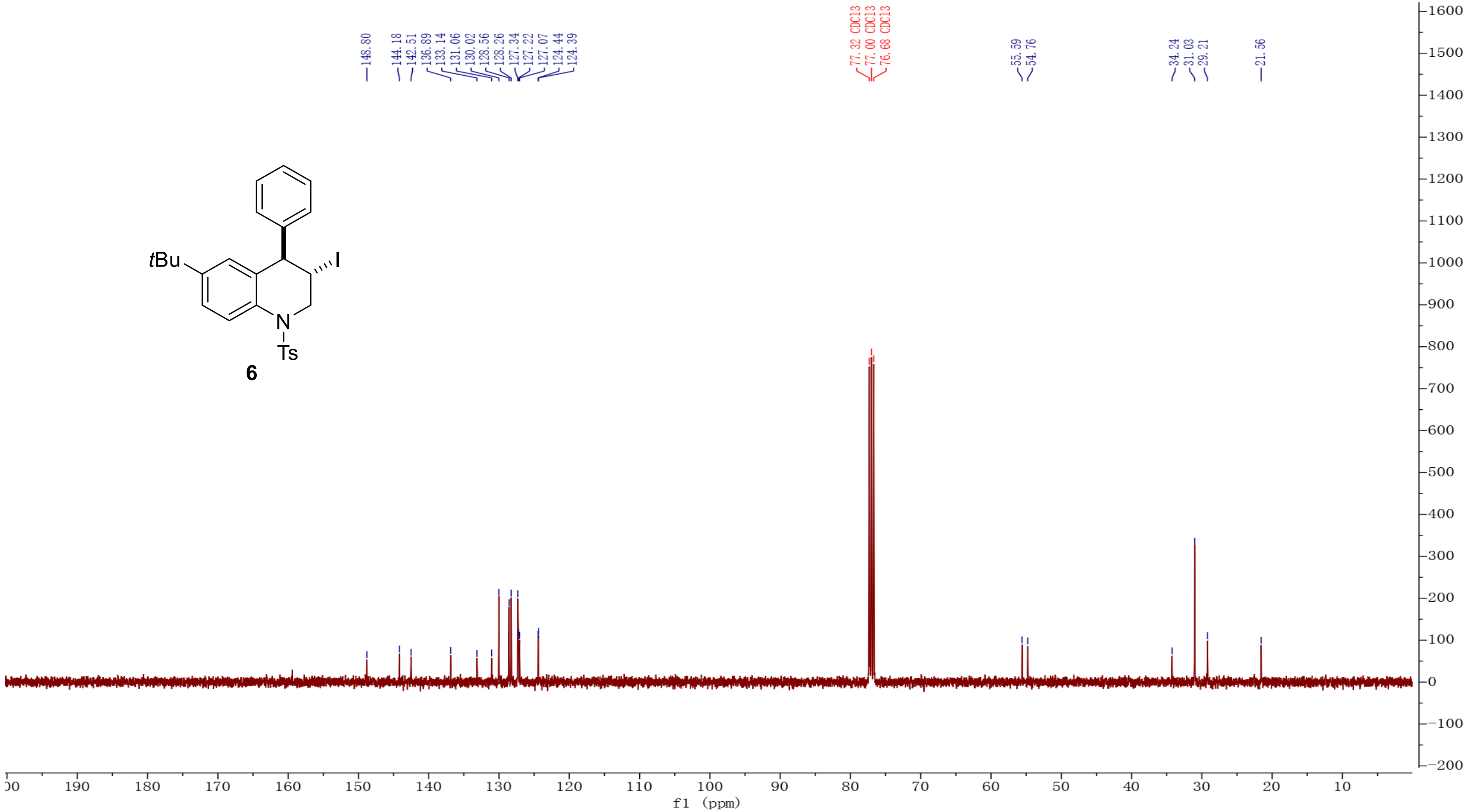

Supplementary Figure 34. ^{13}C NMR spectra of compound **6**.

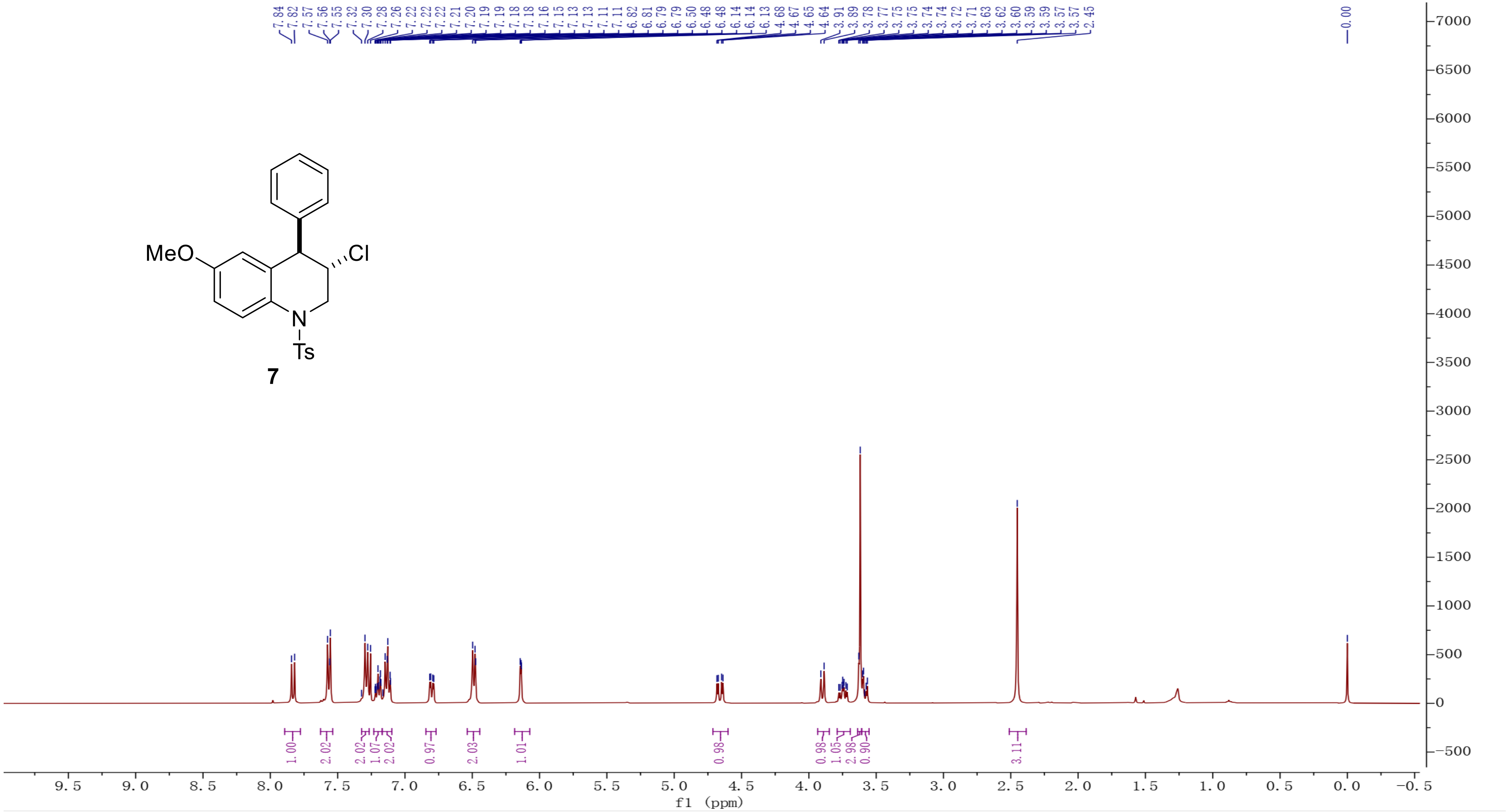

Supplementary Figure 35. ^{1}H NMR spectra of compound **7**.

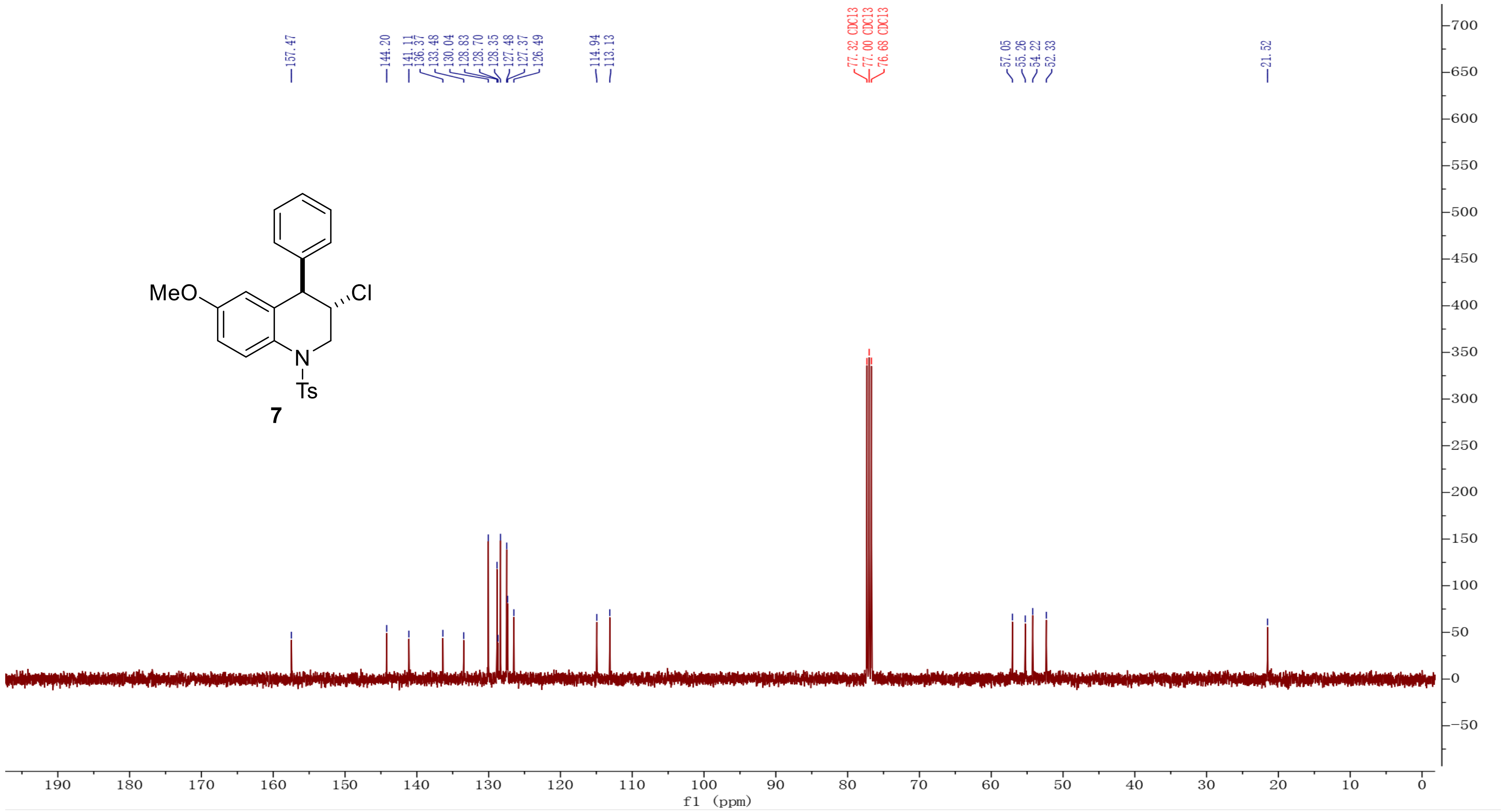

Supplementary Figure 36. ^{13}C NMR spectra of compound **7**.

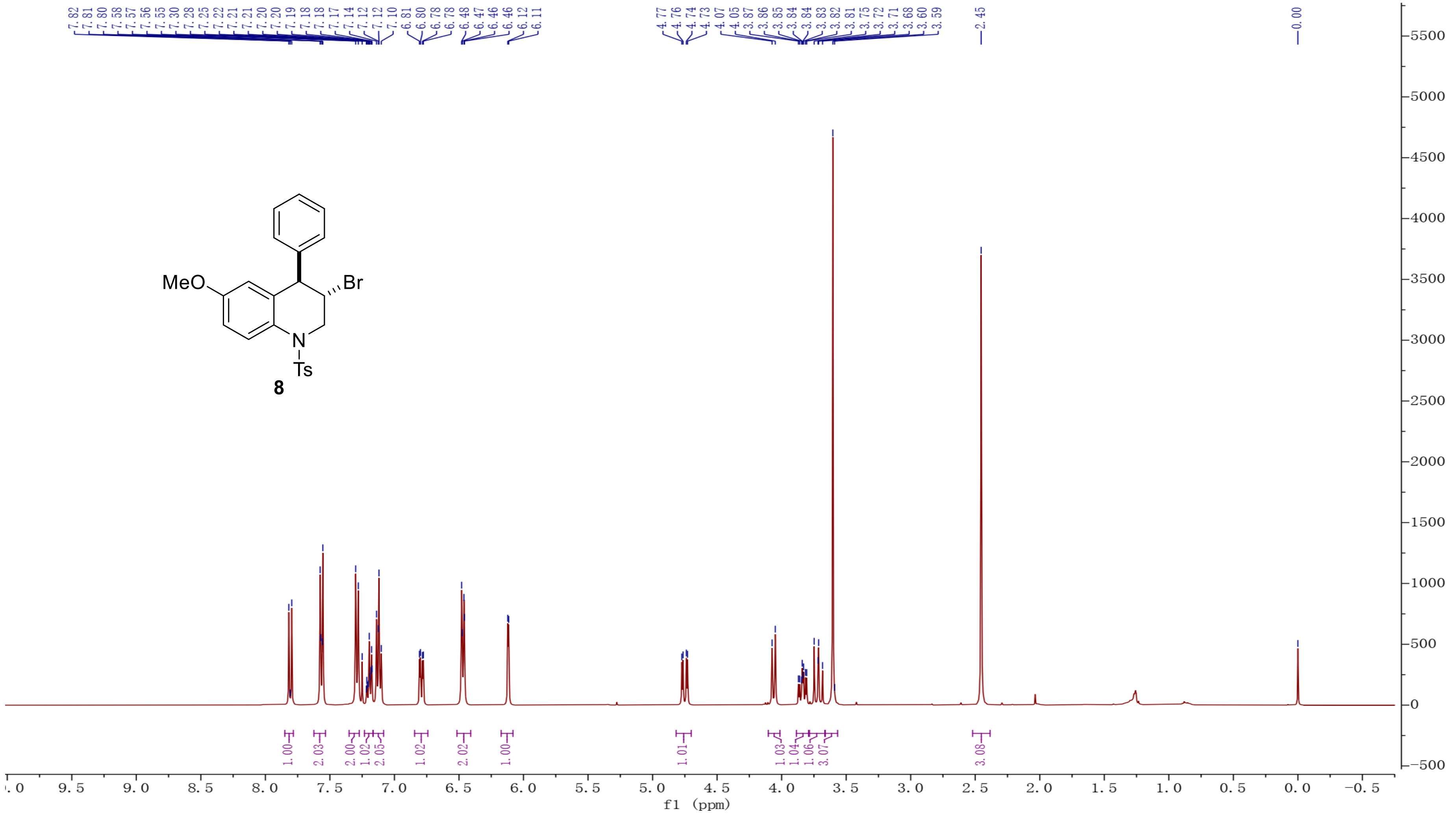

Supplementary Figure 37. ^{1}H NMR spectra of compound **8**.

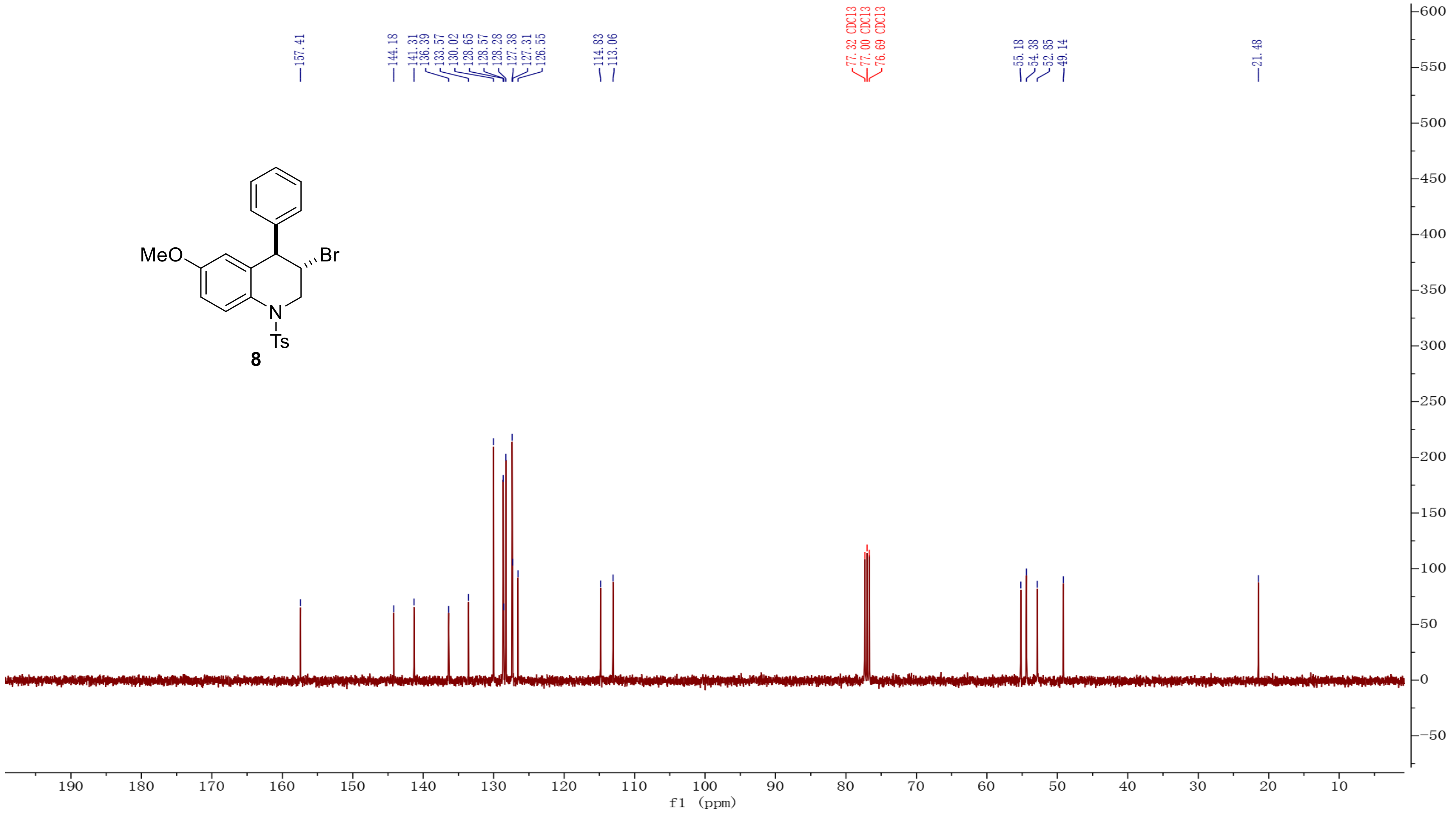

Supplementary Figure 38. ^{13}C NMR spectra of compound **8**.

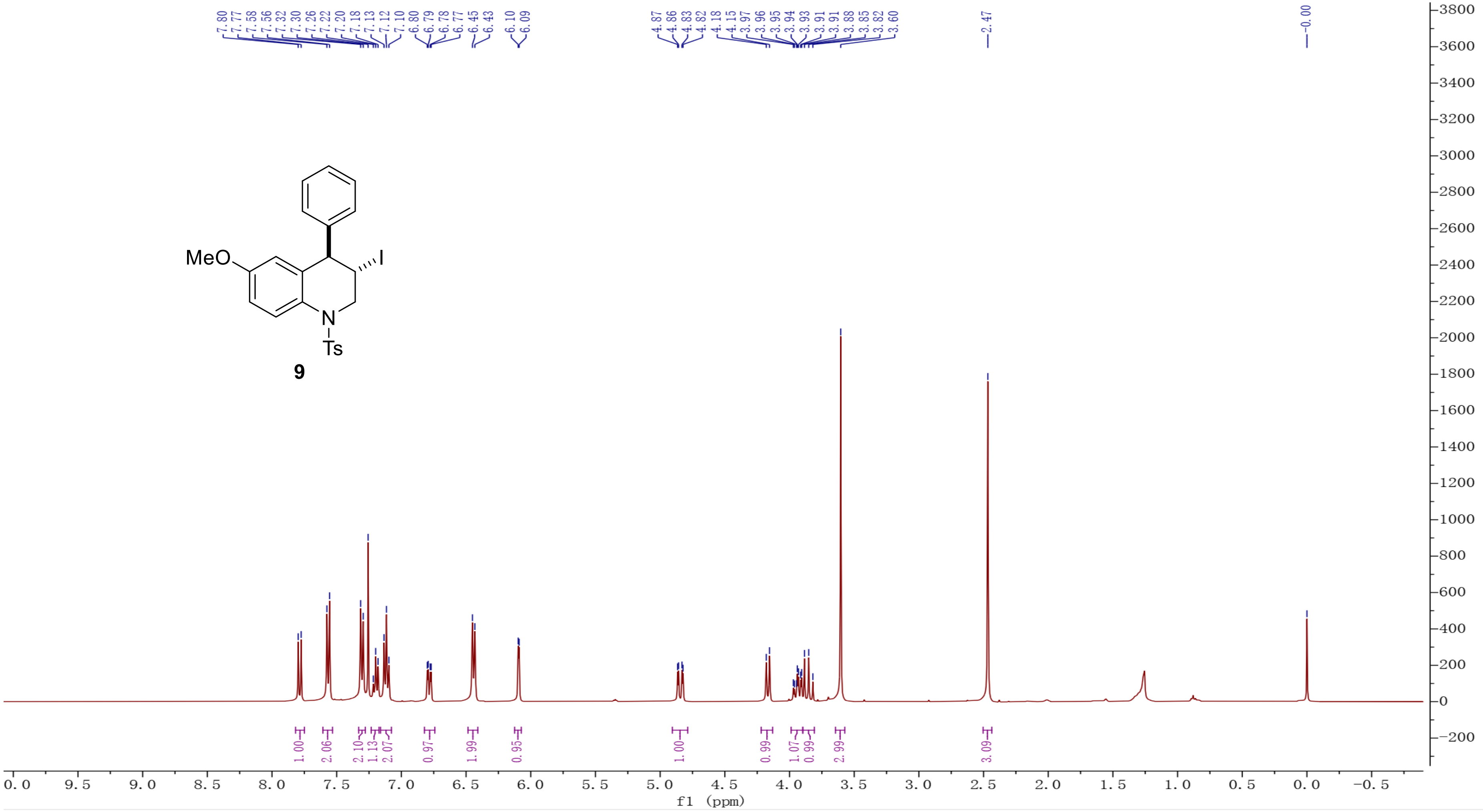

Supplementary Figure 39. ^{1}H NMR spectra of compound **9**.

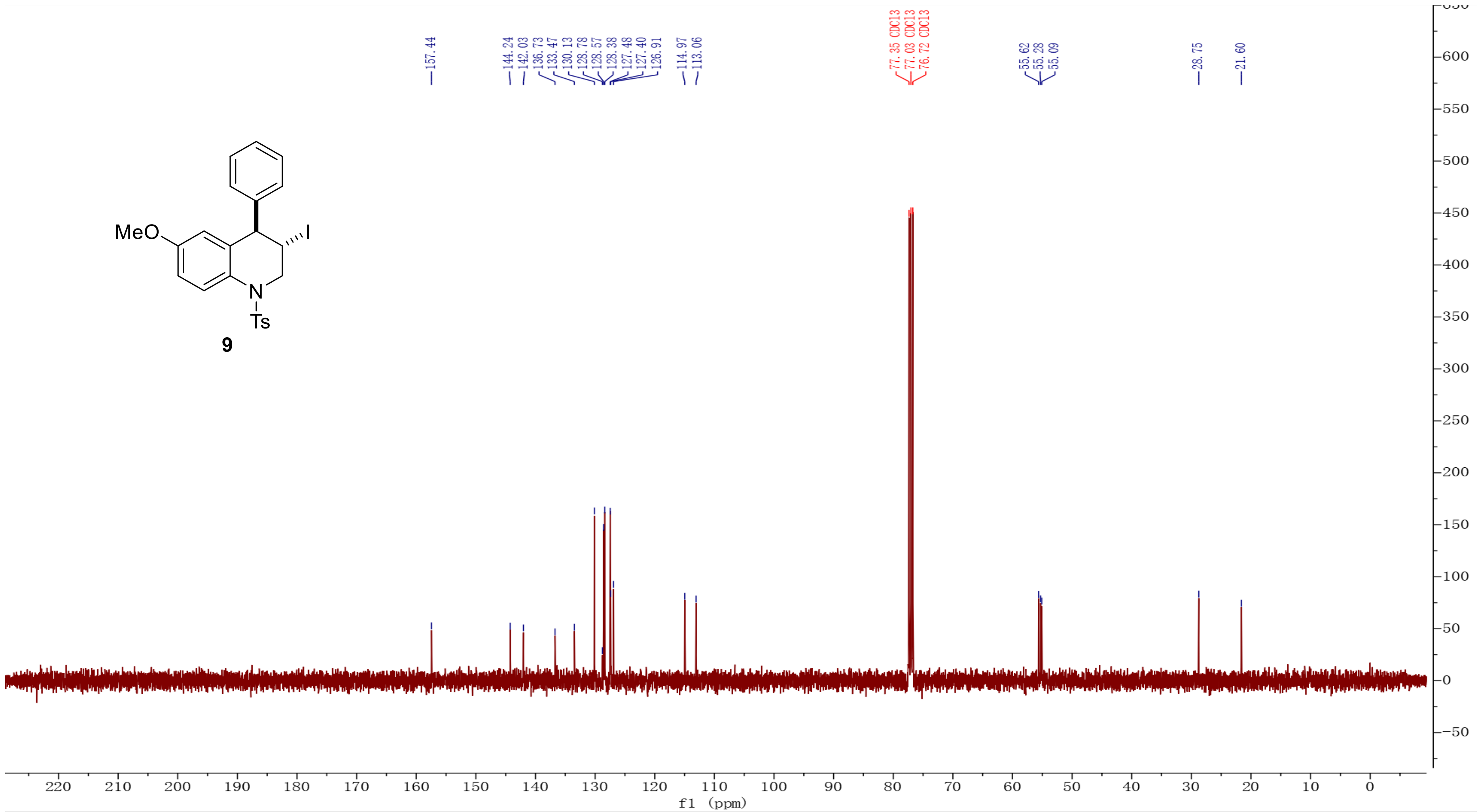

Supplementary Figure 40. ^{13}C NMR spectra of compound **9**.

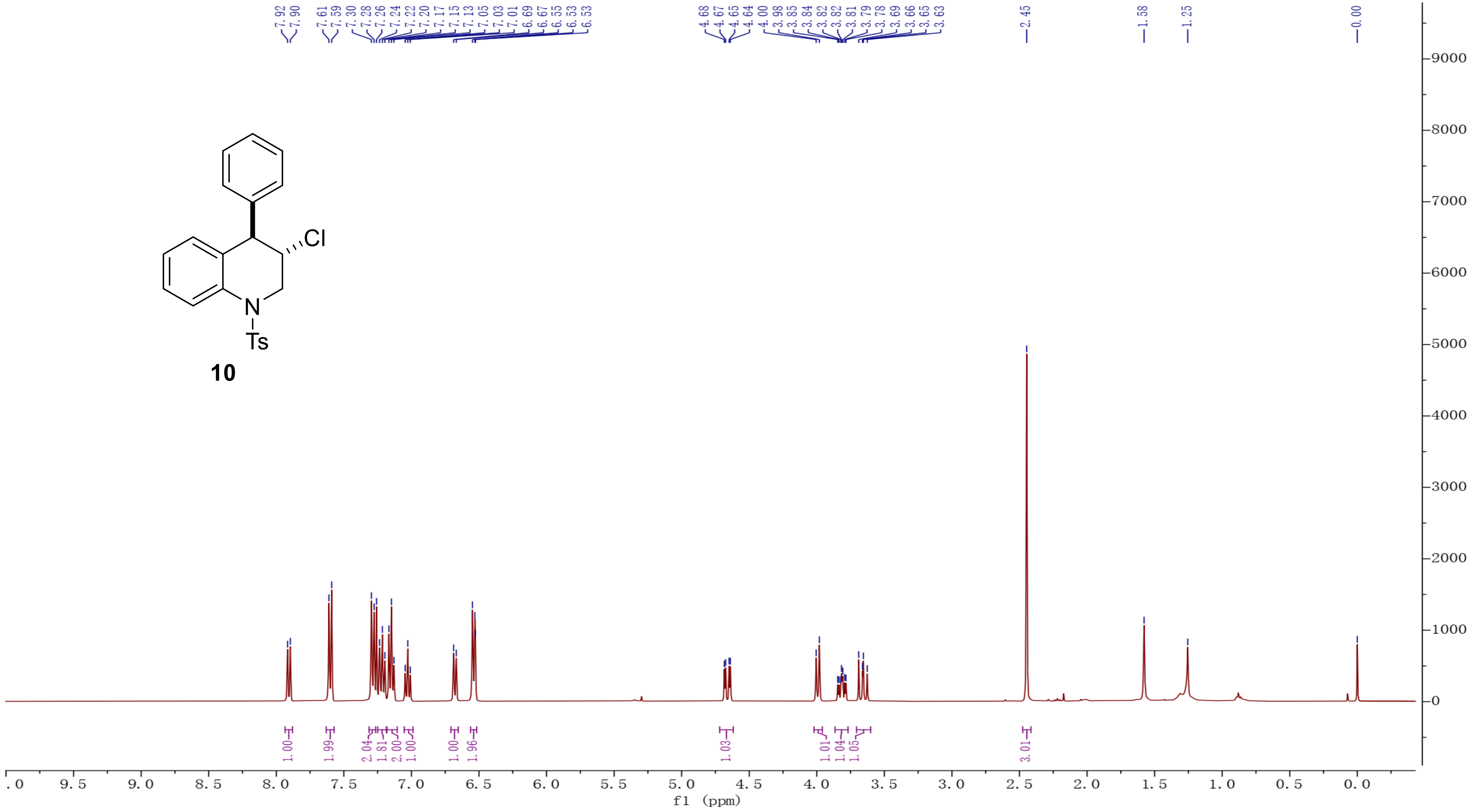

Supplementary Figure 41. ^{1}H NMR spectra of compound **10**.

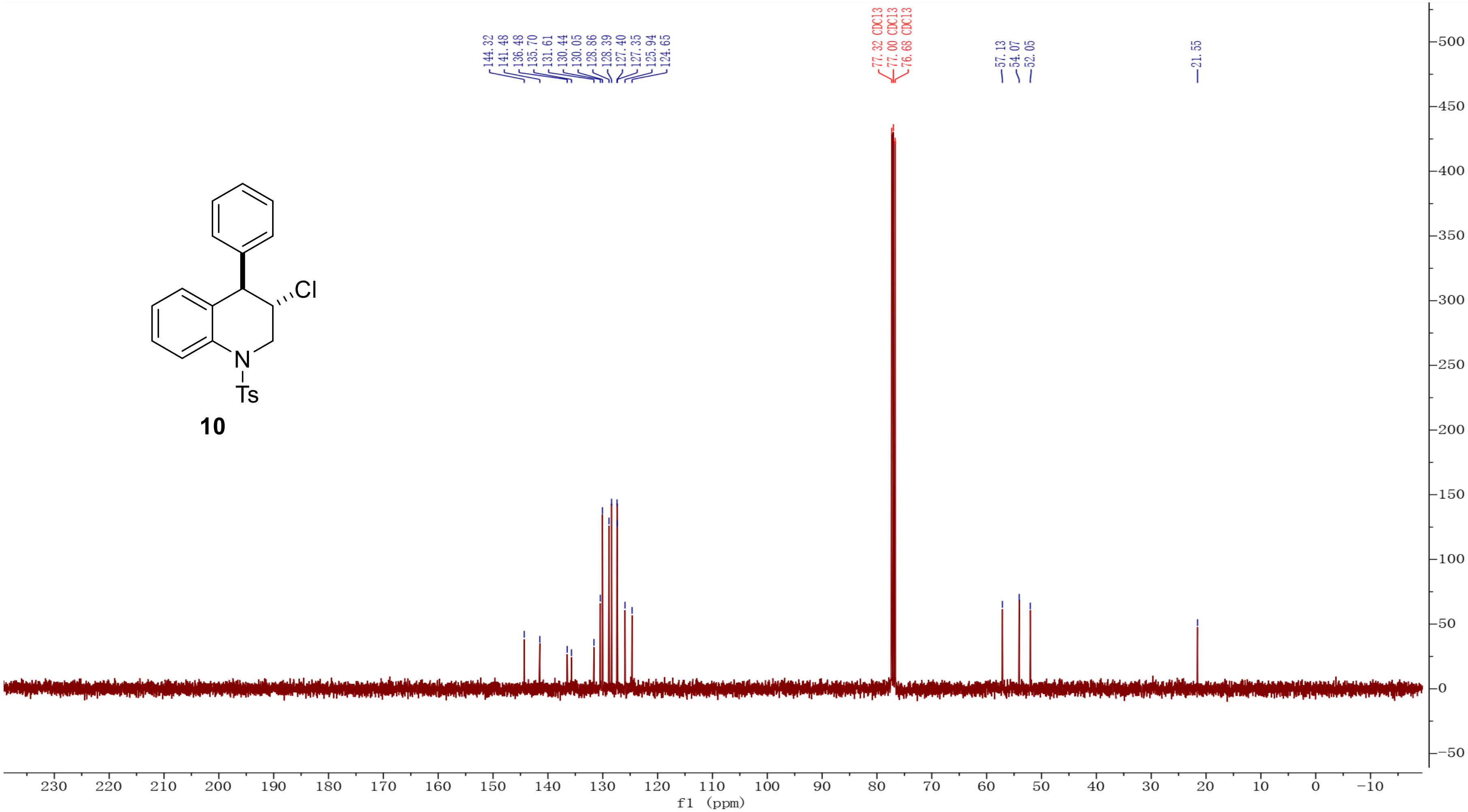

Supplementary Figure 42. ^{13}C NMR spectra of compound **10**.

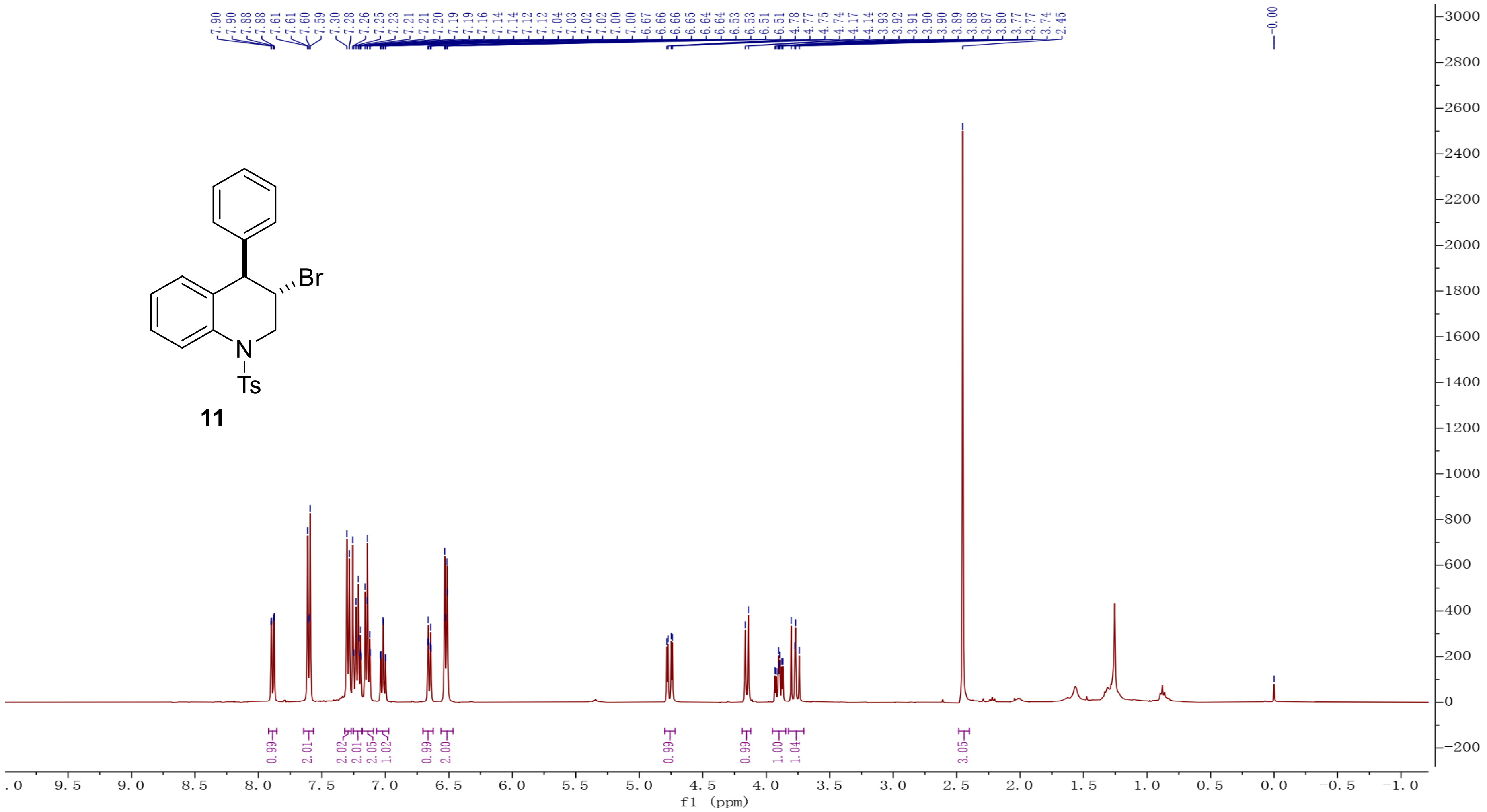

Supplementary Figure 43. ^{1}H NMR spectra of compound **11**.

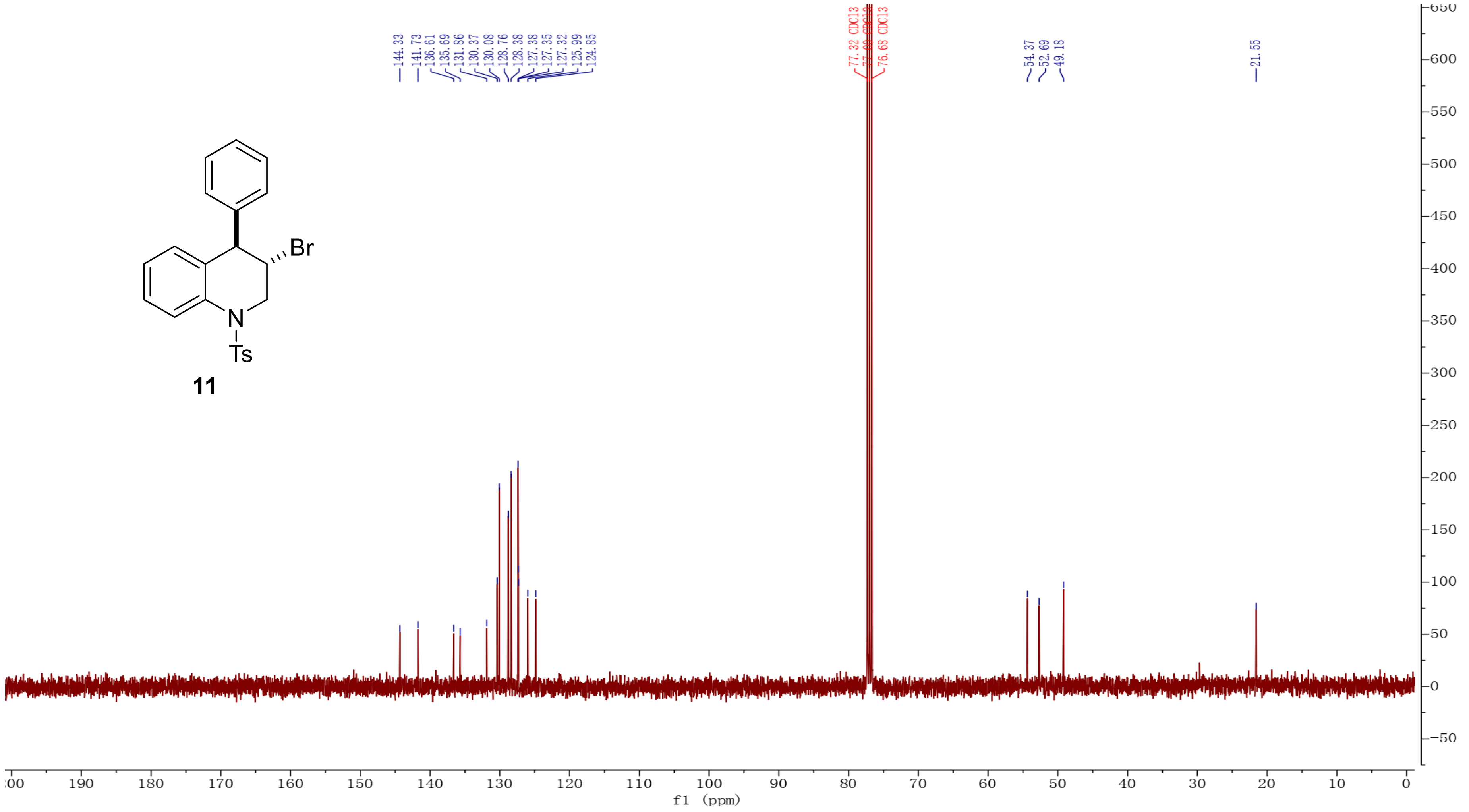

Supplementary Figure 44. ^{13}C NMR spectra of compound **11**.

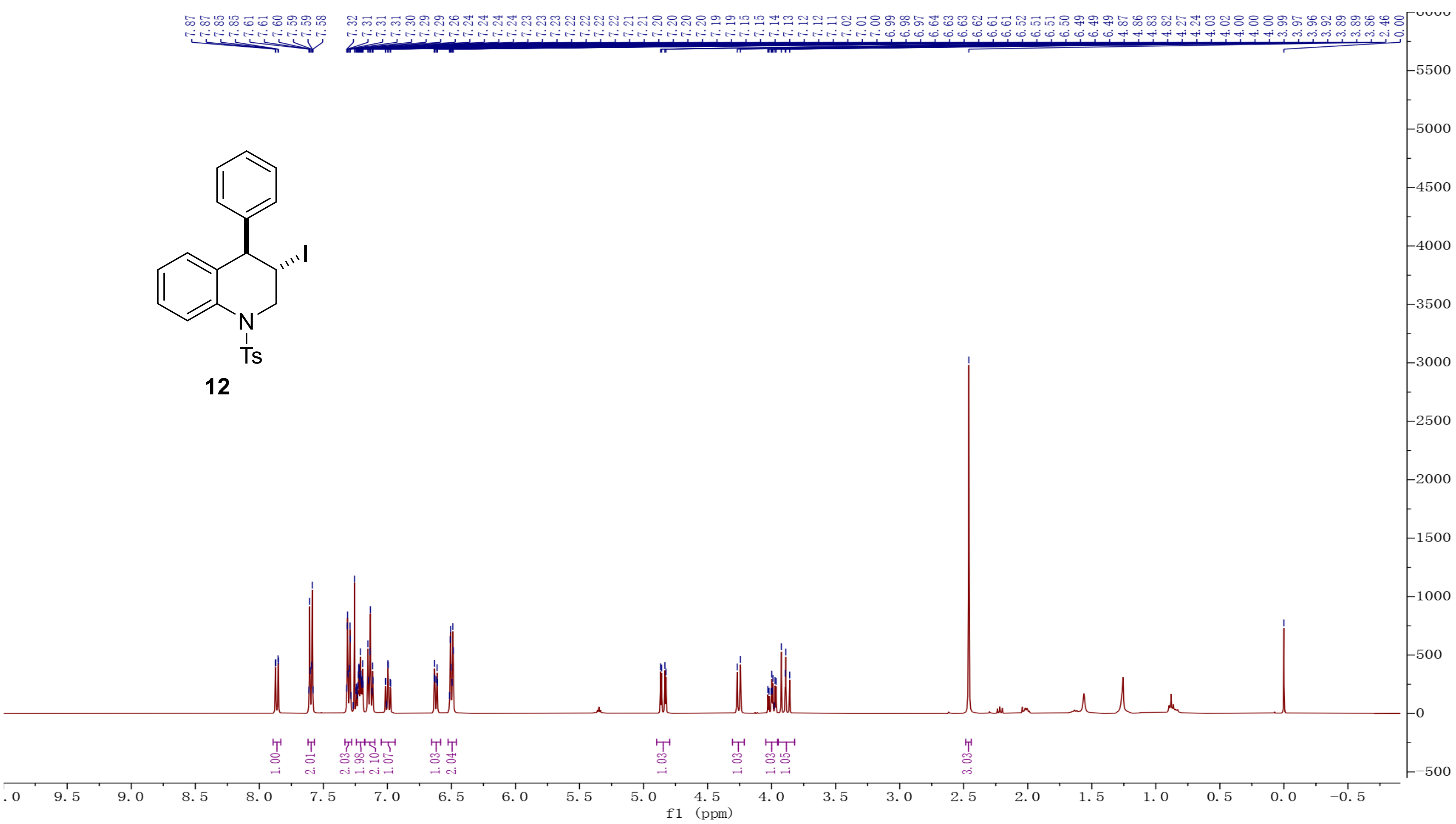

Supplementary Figure 45. ^{1}H NMR spectra of compound **12**.

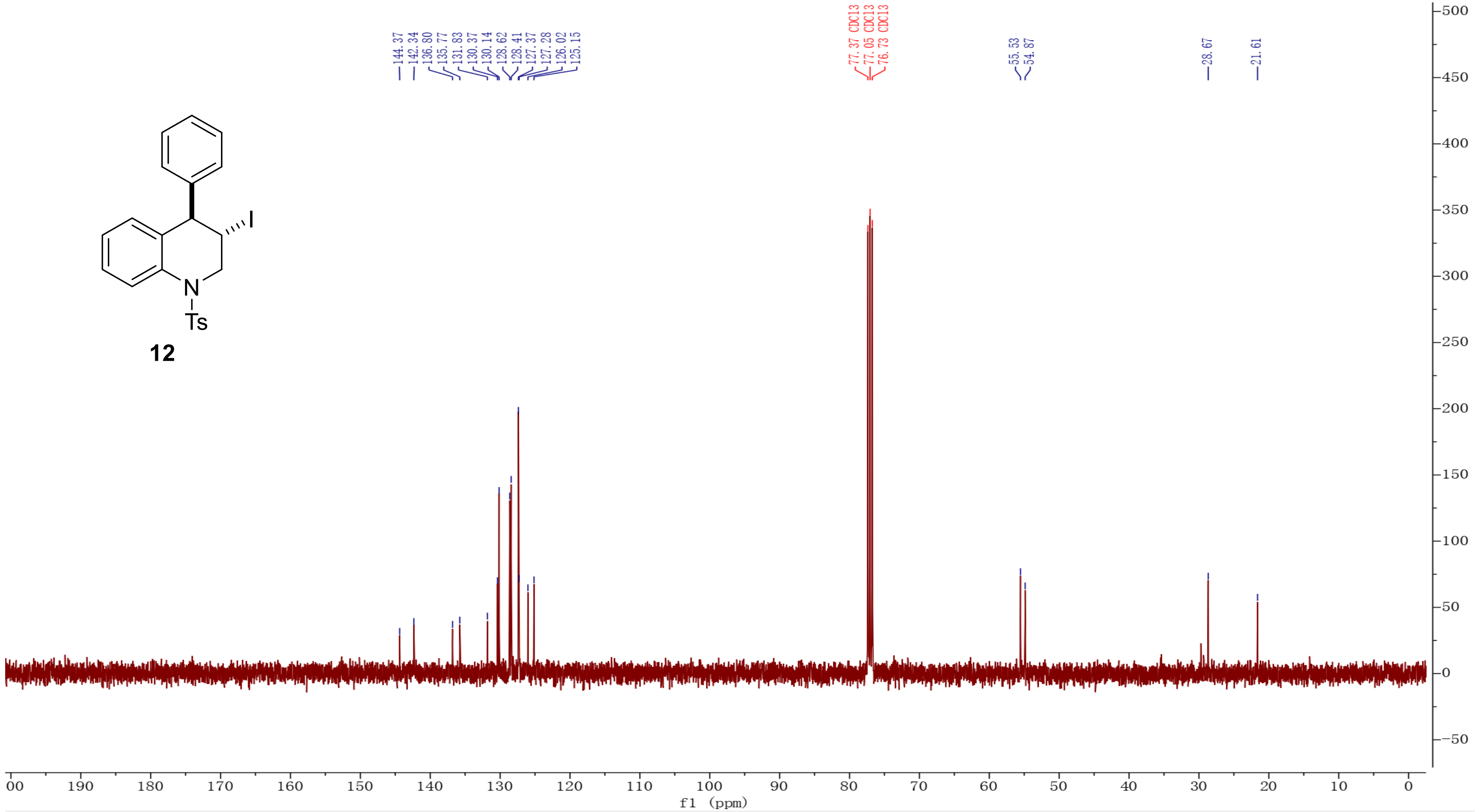

Supplementary Figure 46. ^{13}C NMR spectra of compound **12**.

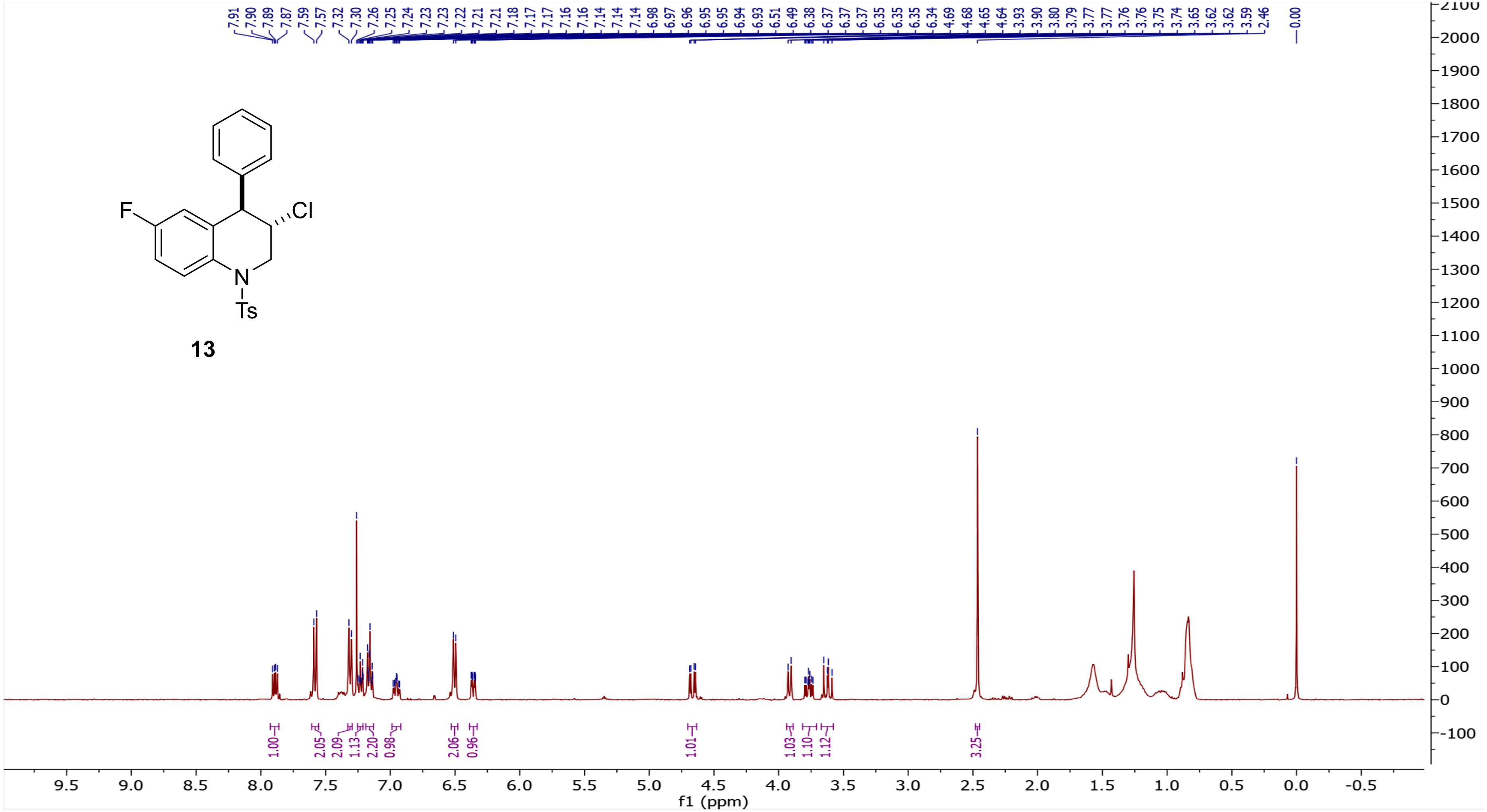

Supplementary Figure 47. ^{1}H NMR spectra of compound **13**.

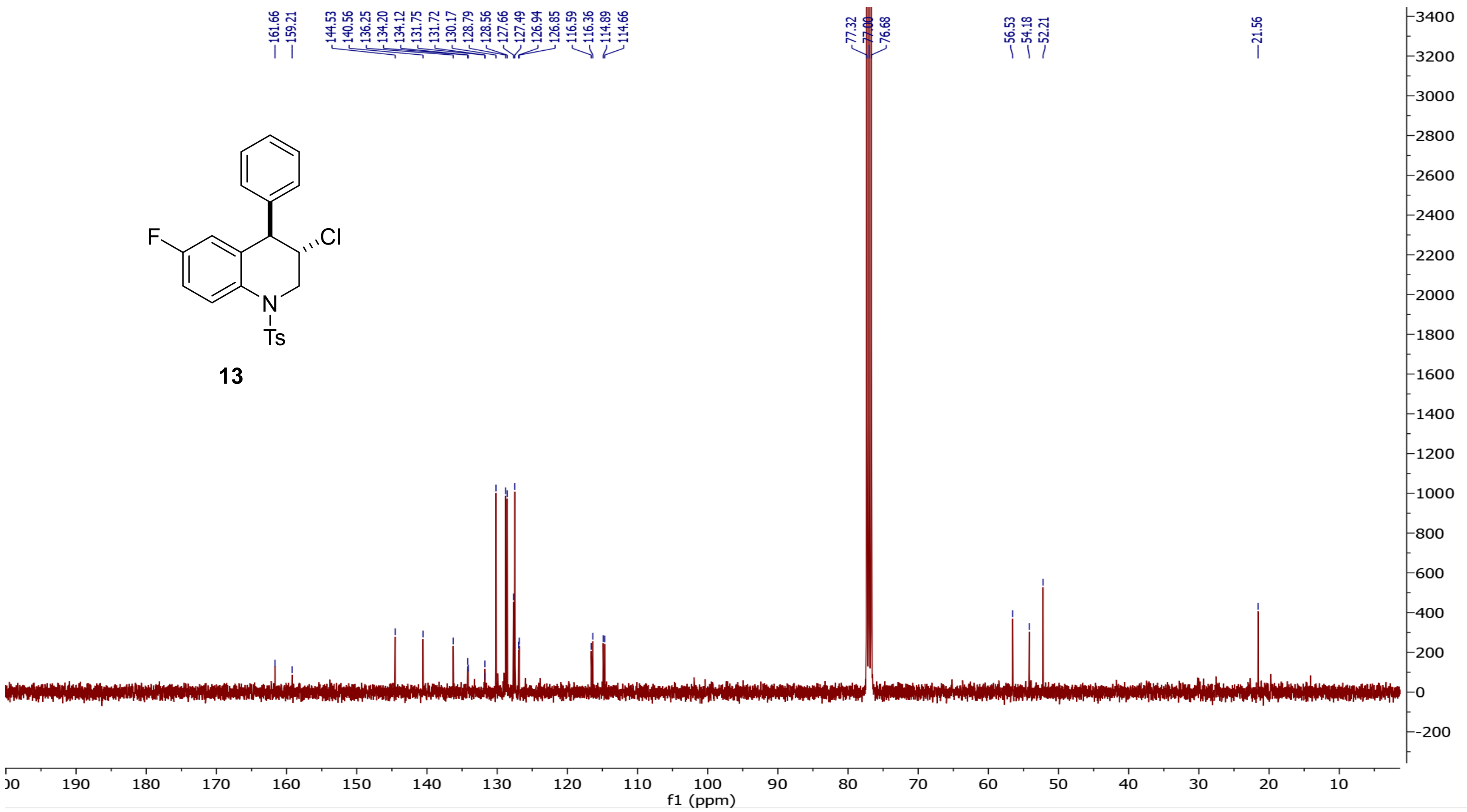

Supplementary Figure 48. ^{13}C NMR spectra of compound **13**.

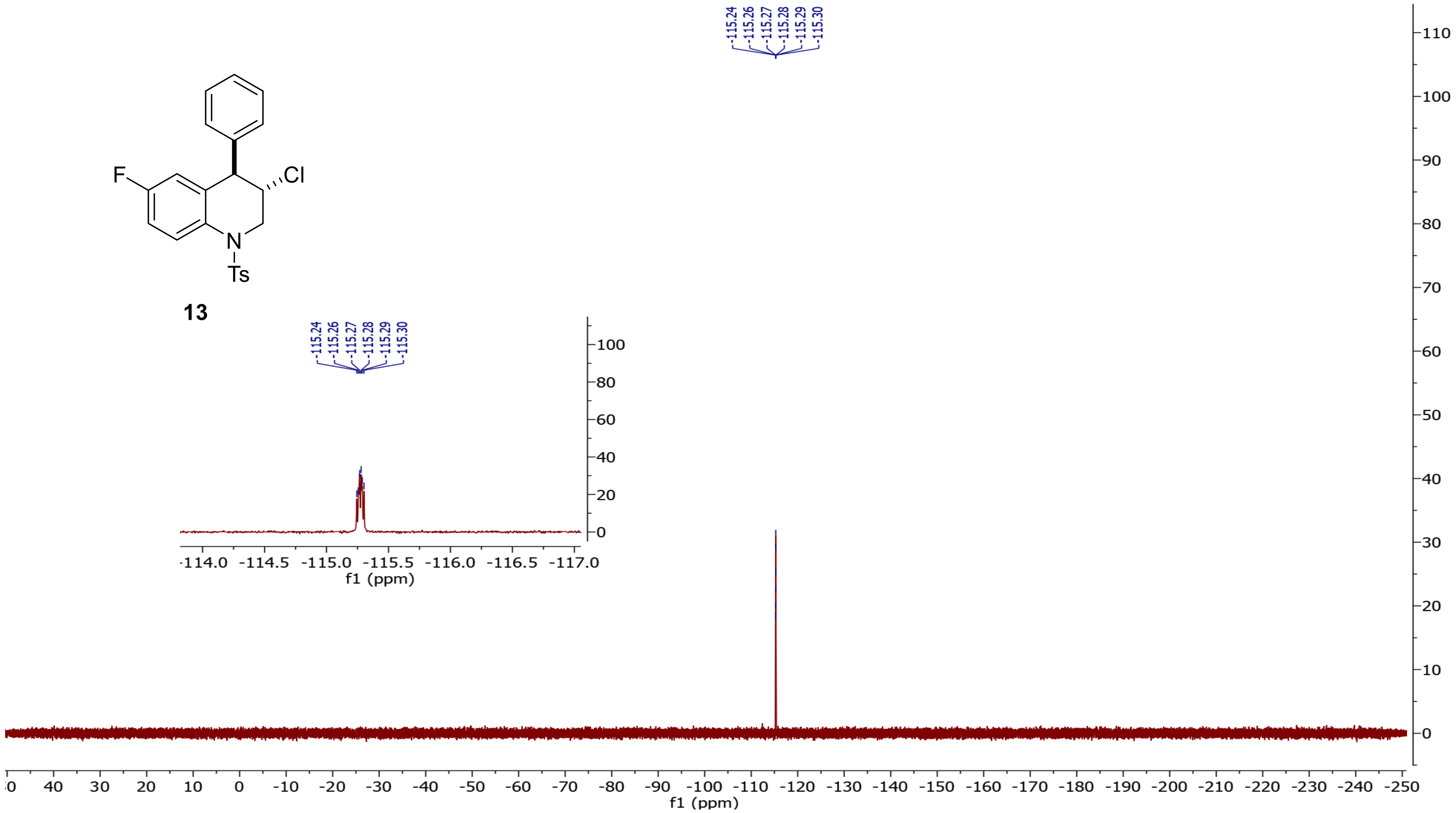

Supplementary Figure 49. ^{19}F NMR spectra of compound **13**.

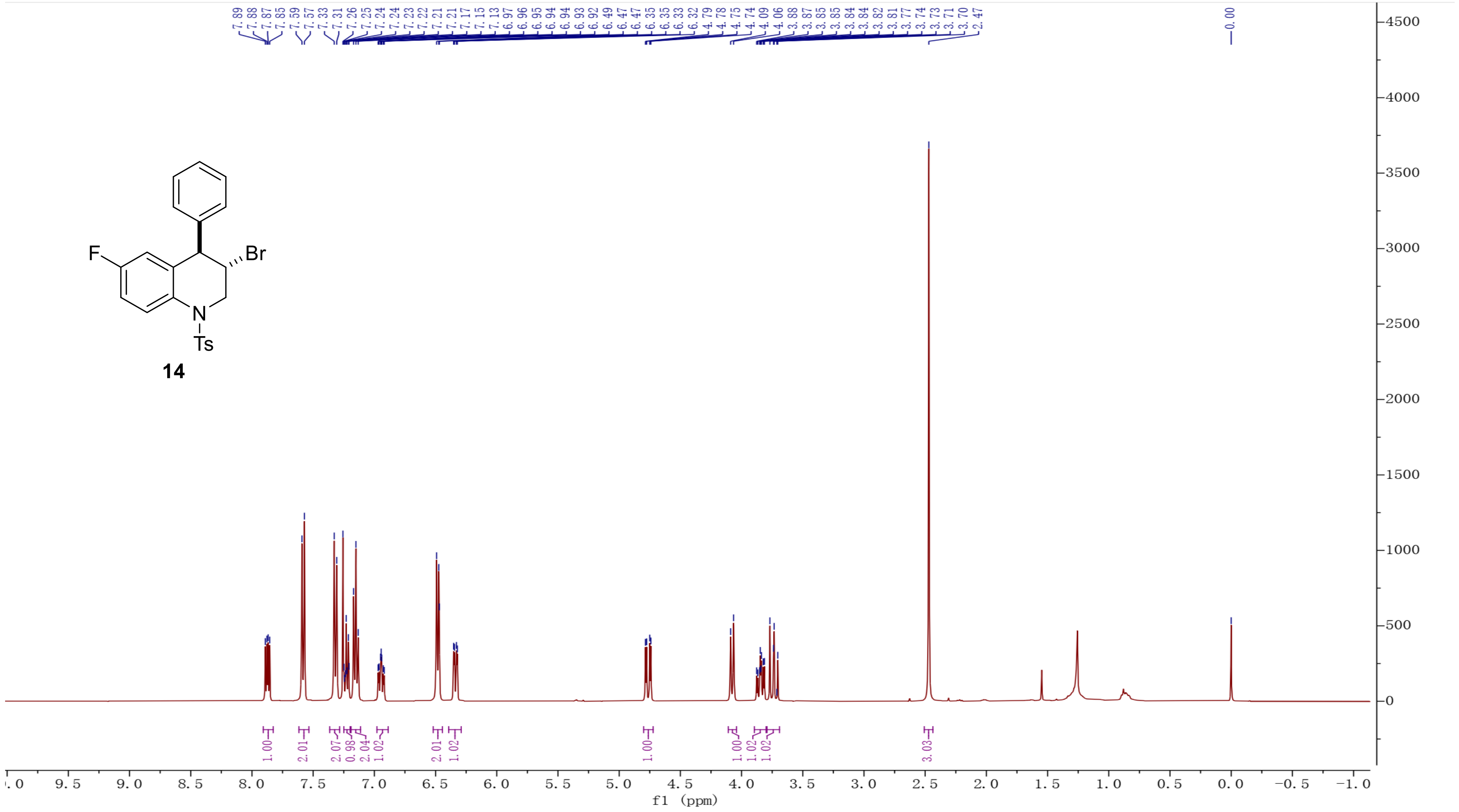

Supplementary Figure 50. ^{1}H NMR spectra of compound **14**.

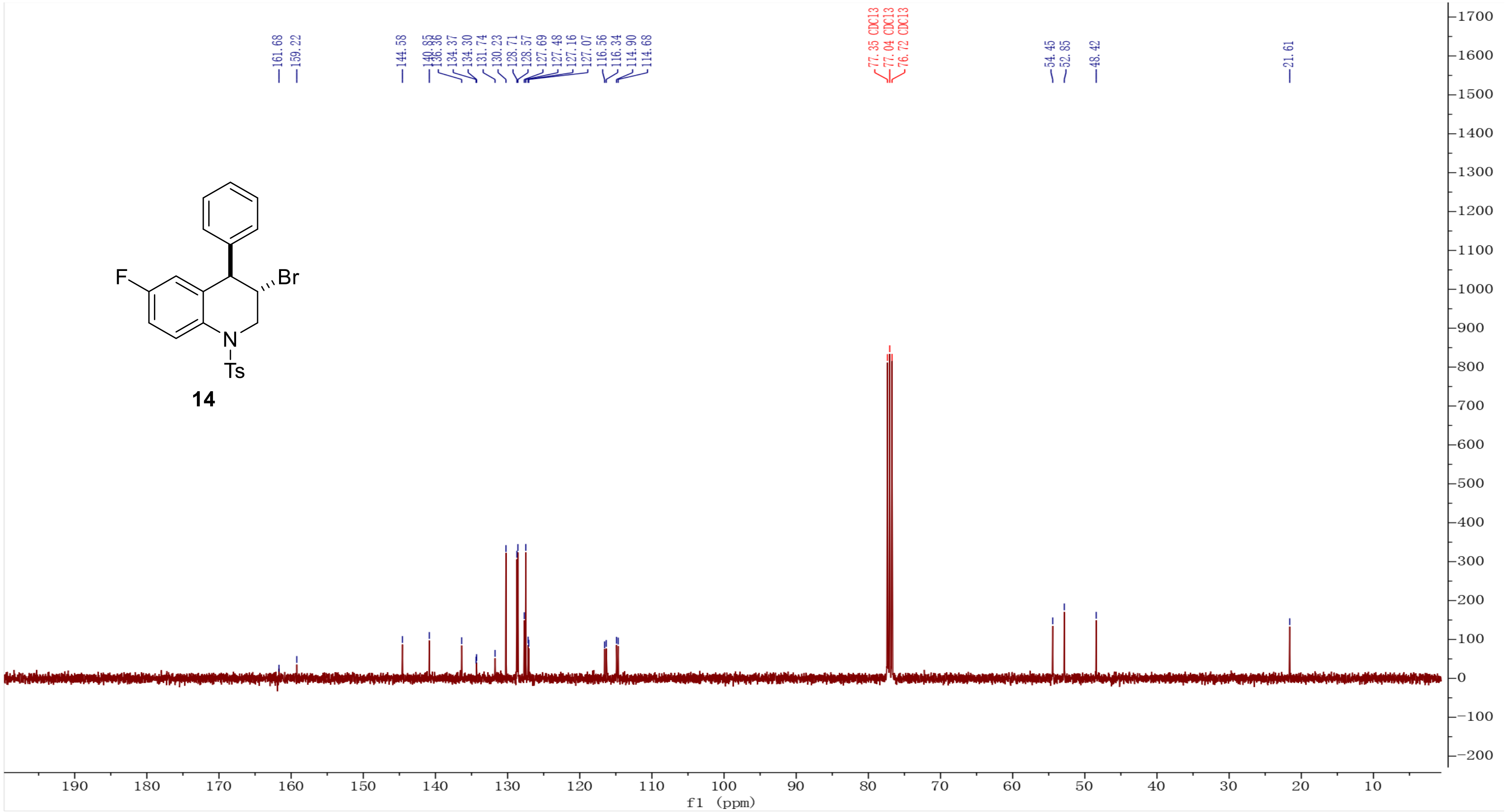

Supplementary Figure 51. ^{13}C NMR spectra of compound **14**.

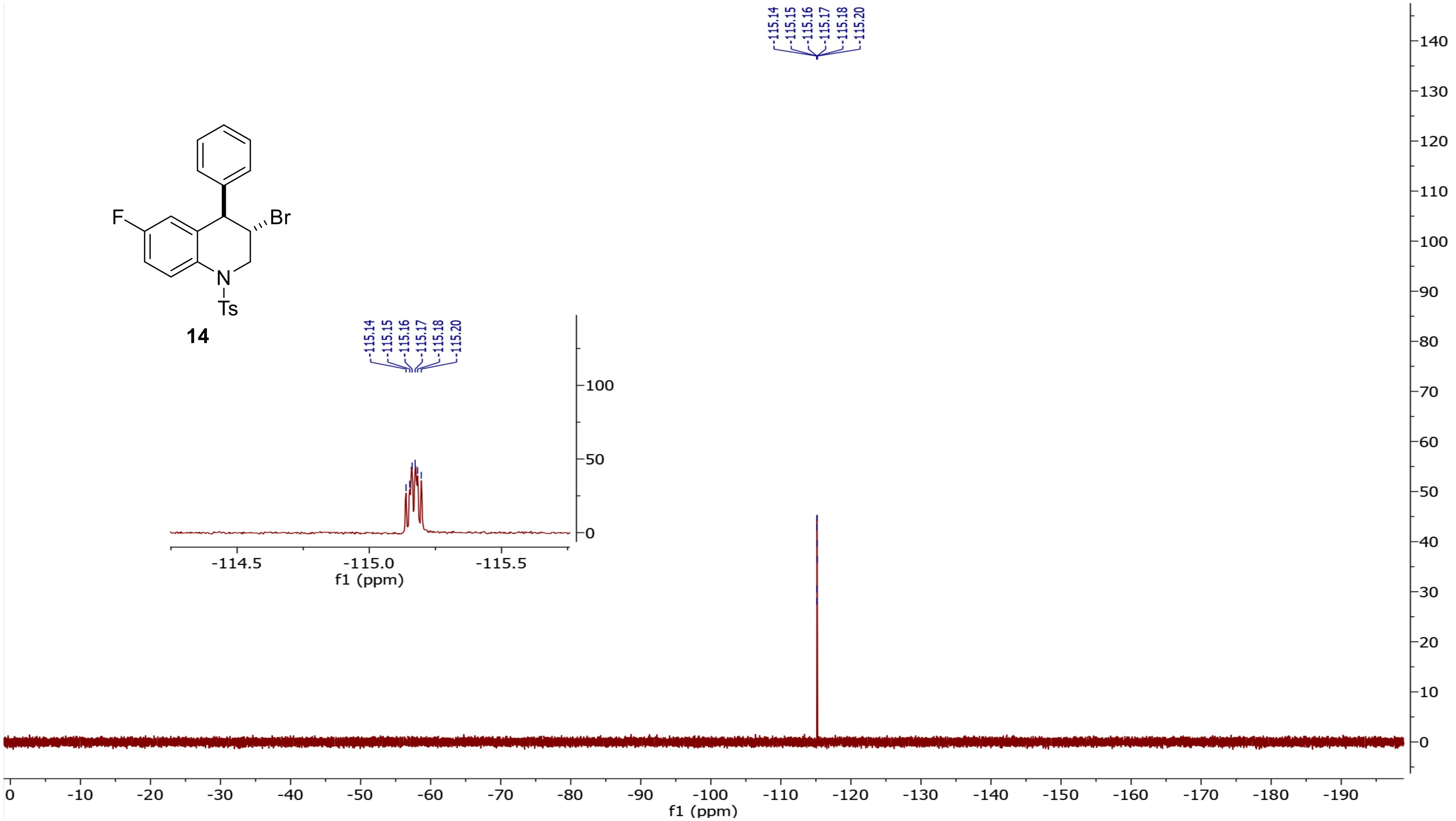

Supplementary Figure 52. ^{19}F NMR spectra of compound **14**.

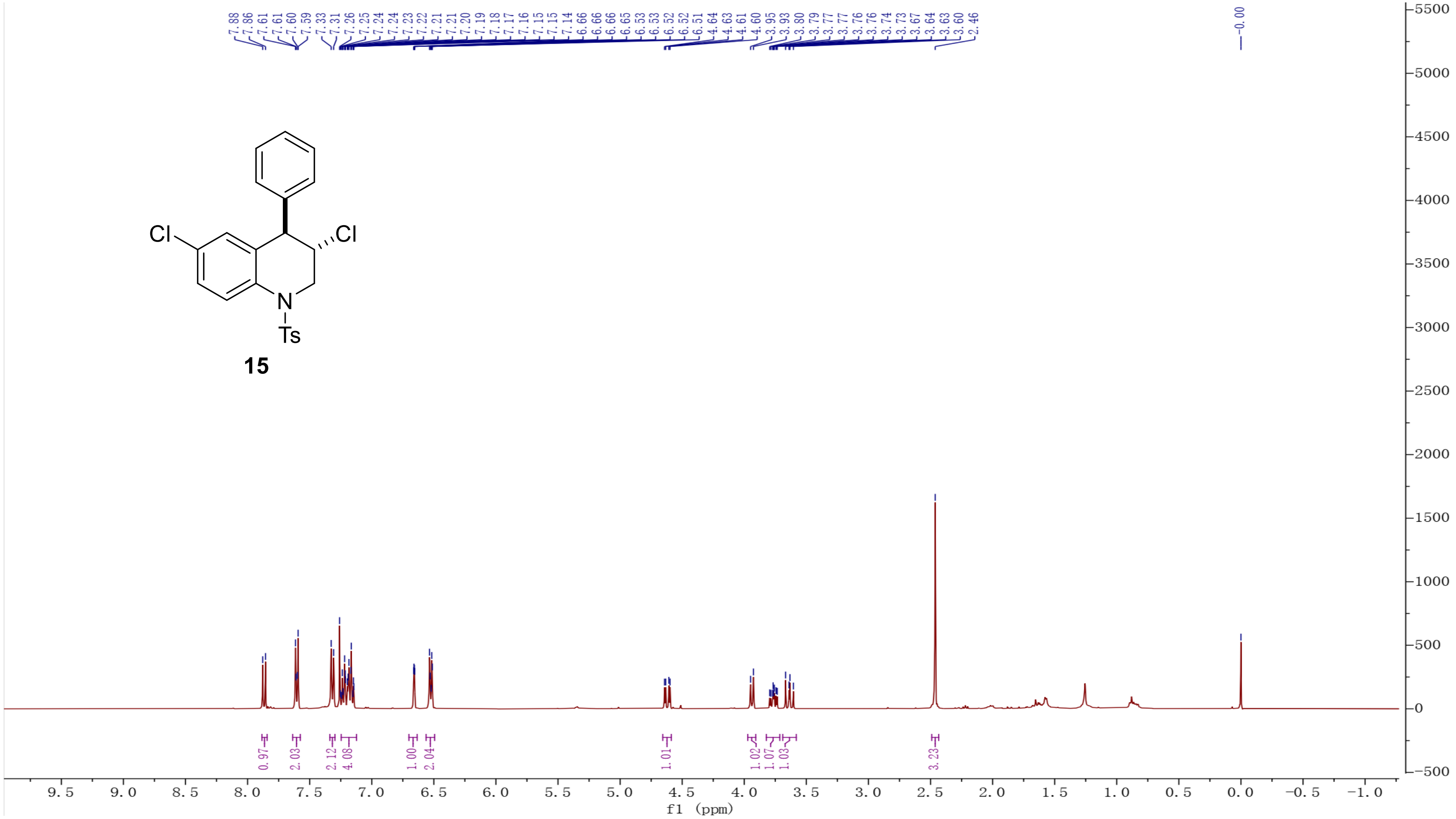

Supplementary Figure 53. ^{1}H NMR spectra of compound **15**.

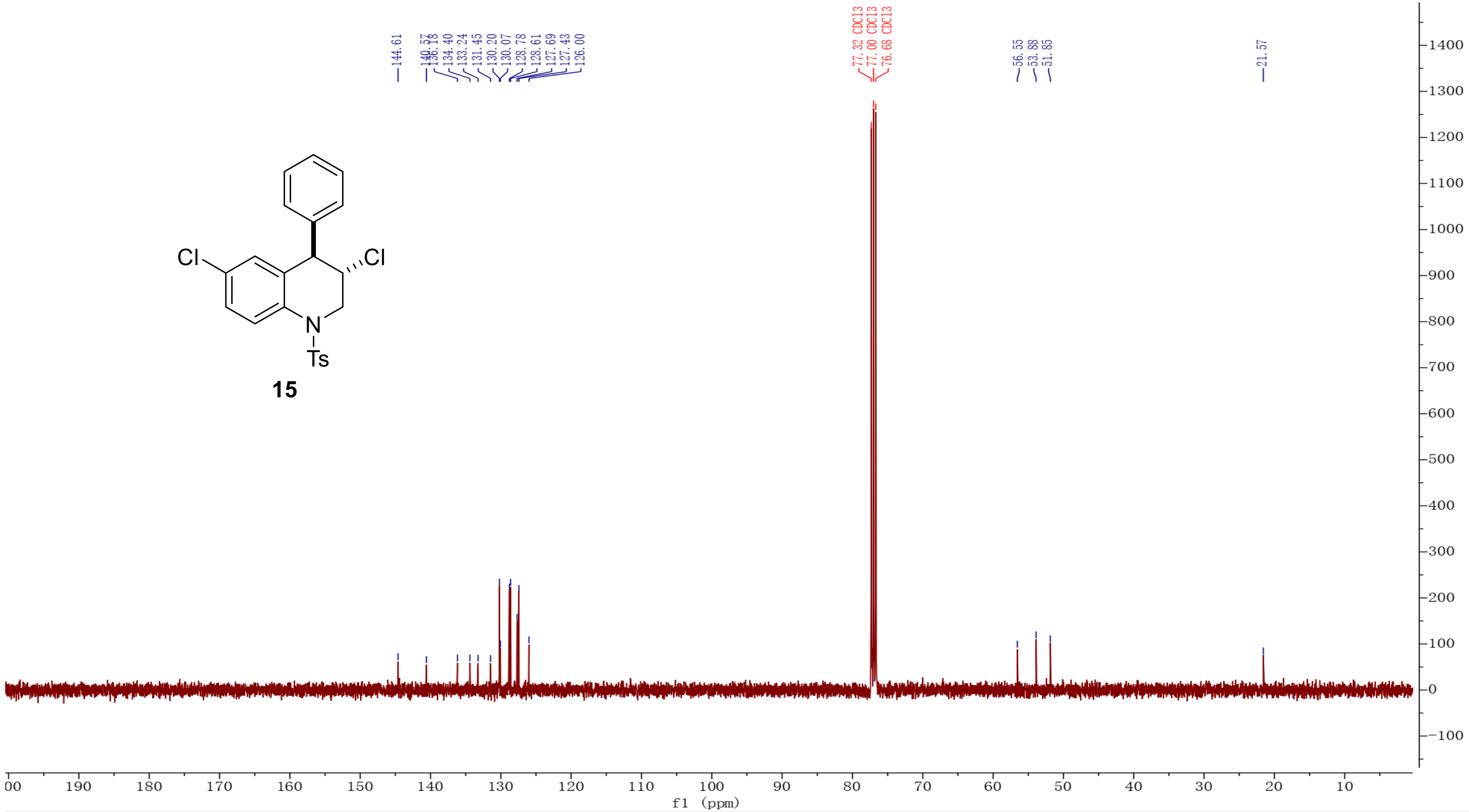

Supplementary Figure 54. ^{13}C NMR spectra of compound **15**.

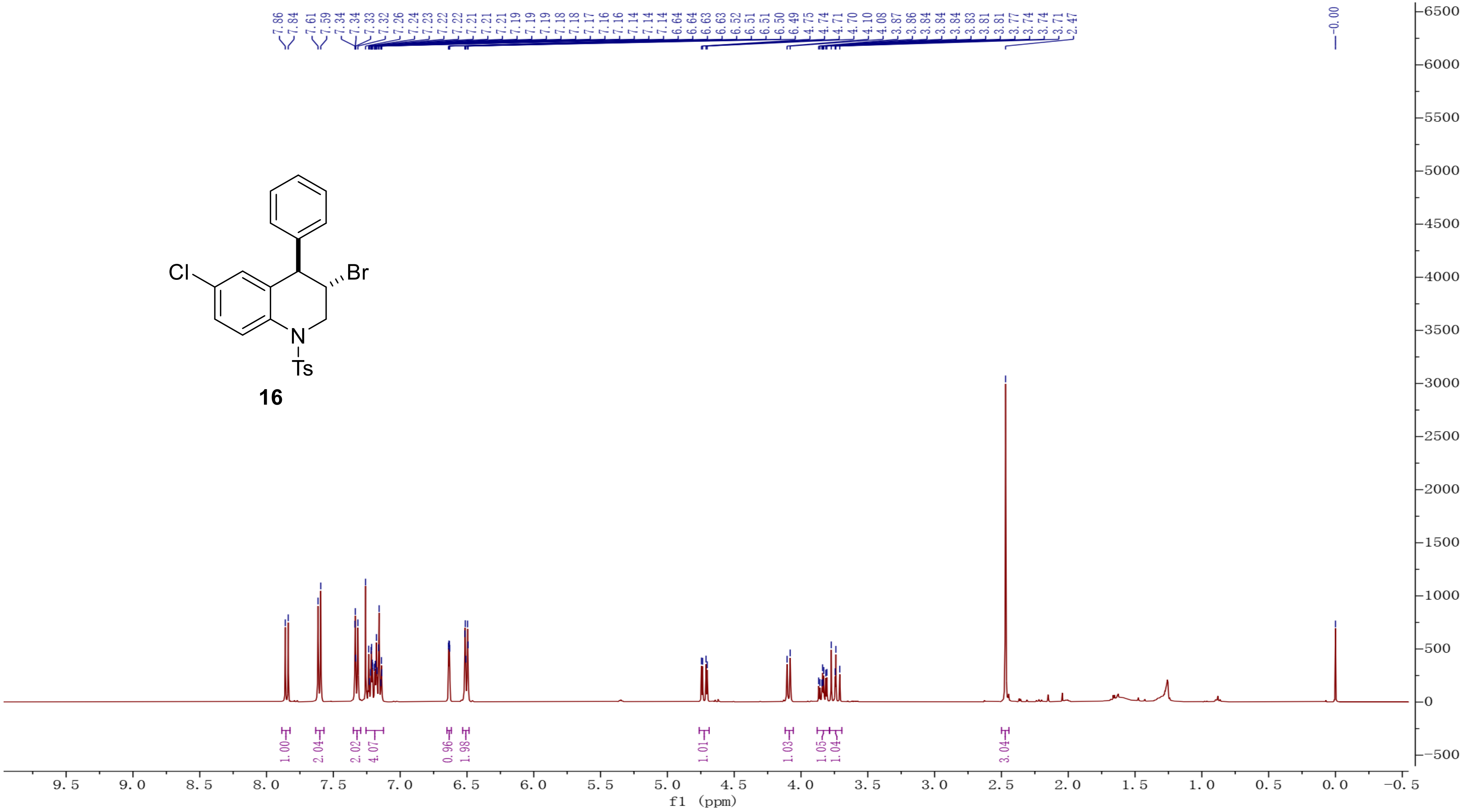

Supplementary Figure 55. ^{1}H NMR spectra of compound **16**.

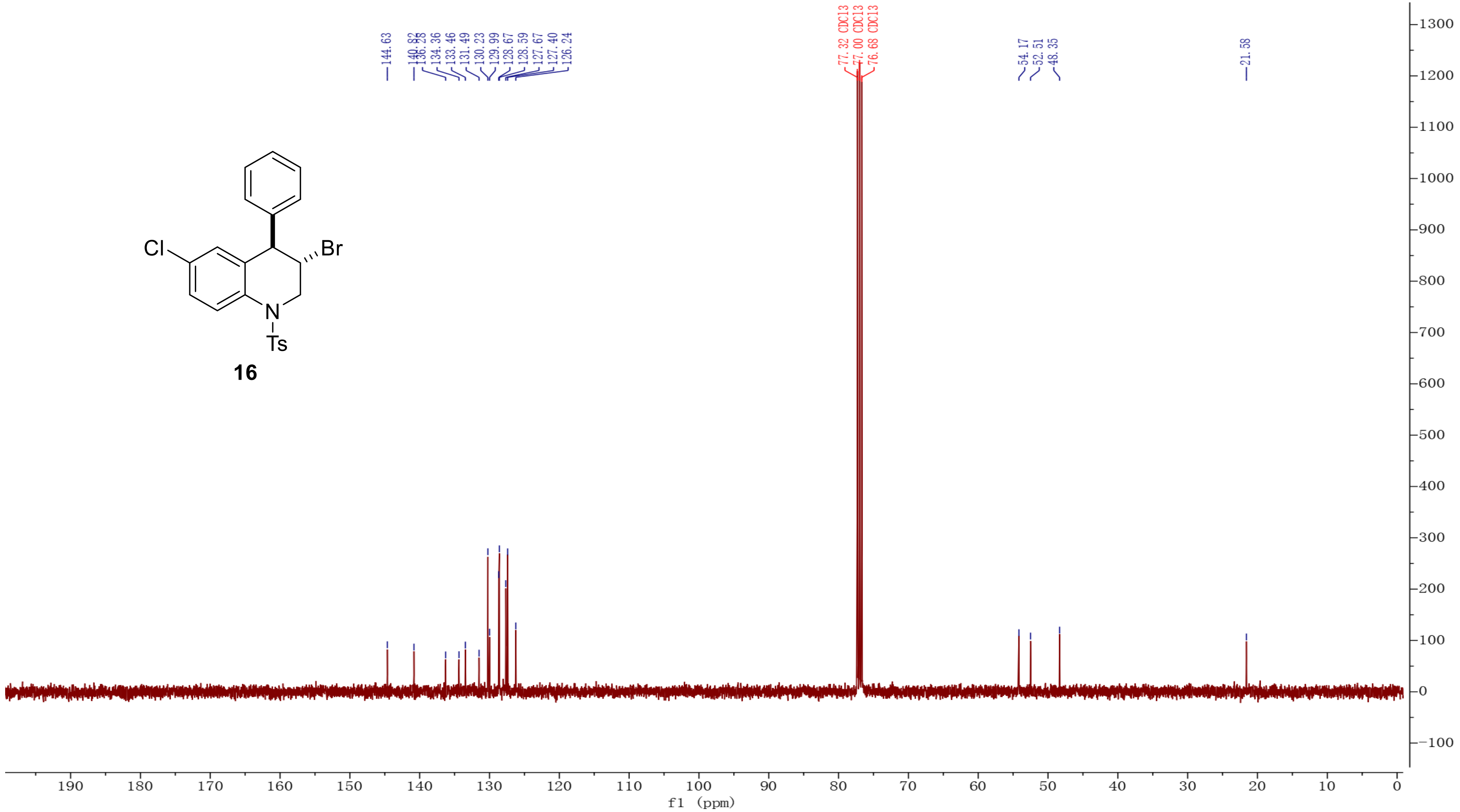

Supplementary Figure 56. ^{13}C NMR spectra of compound **16**.

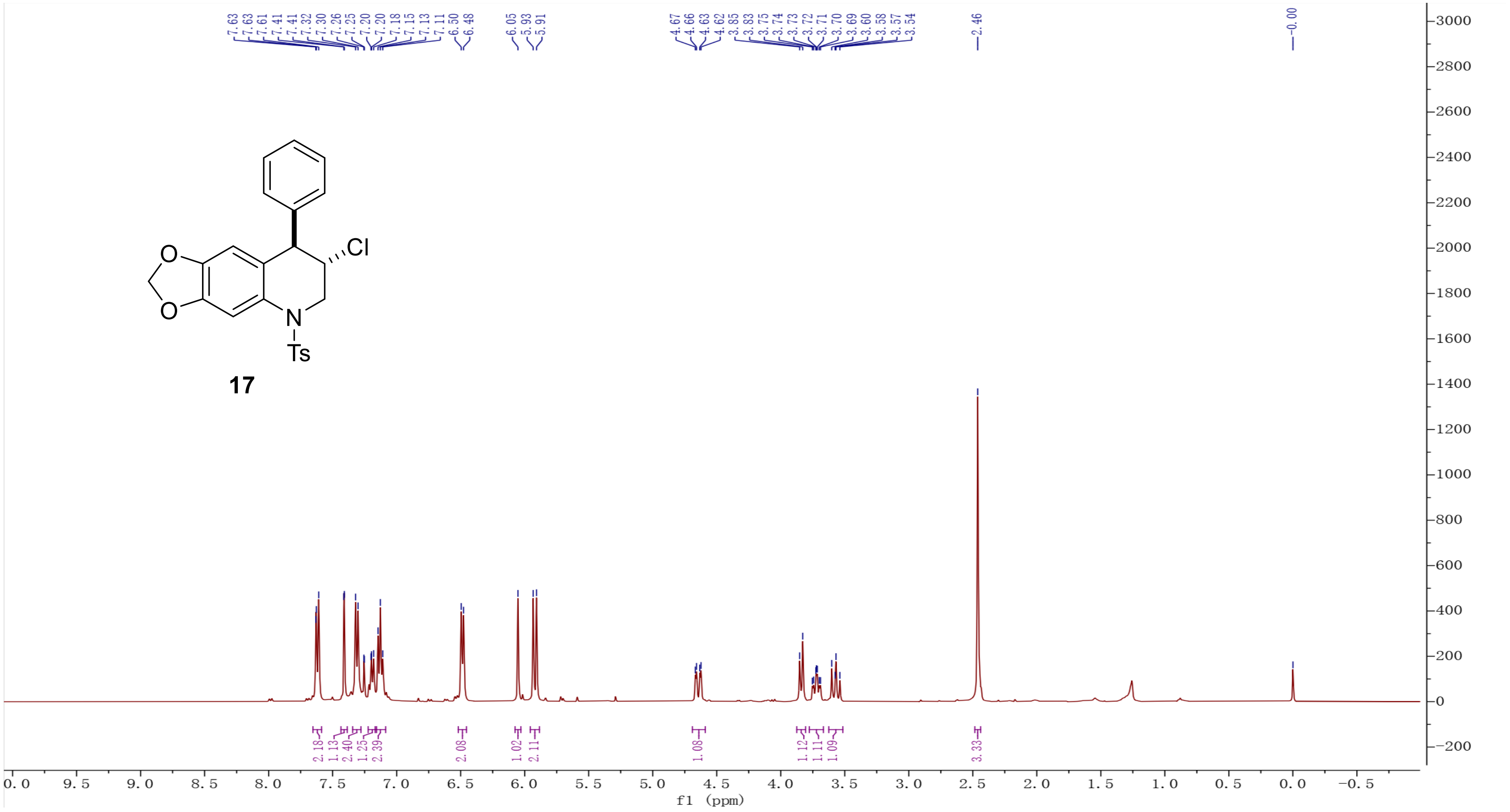

Supplementary Figure 57. ^{1}H NMR spectra of compound **17**.

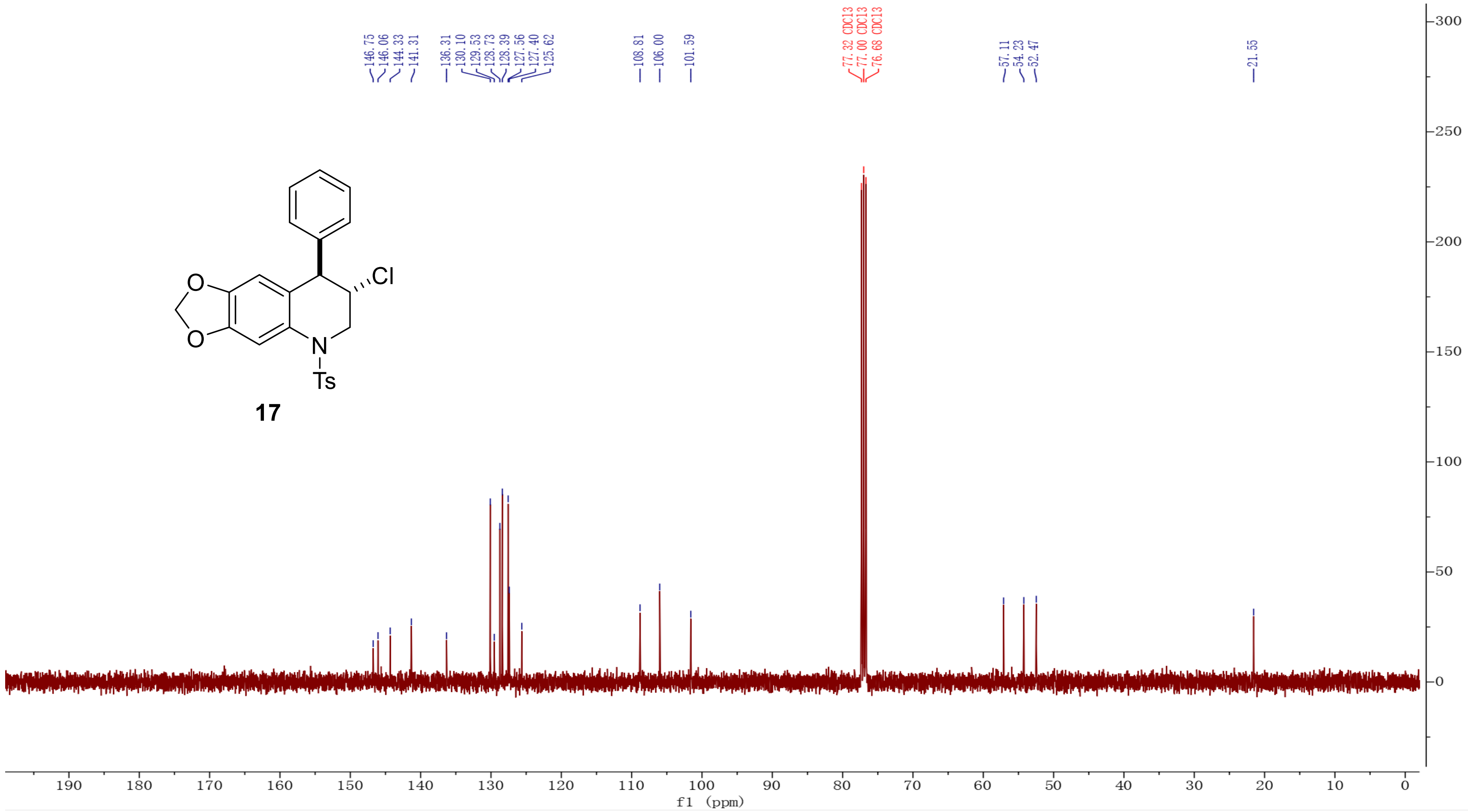

Supplementary Figure 58. ^{13}C NMR spectra of compound **17**.

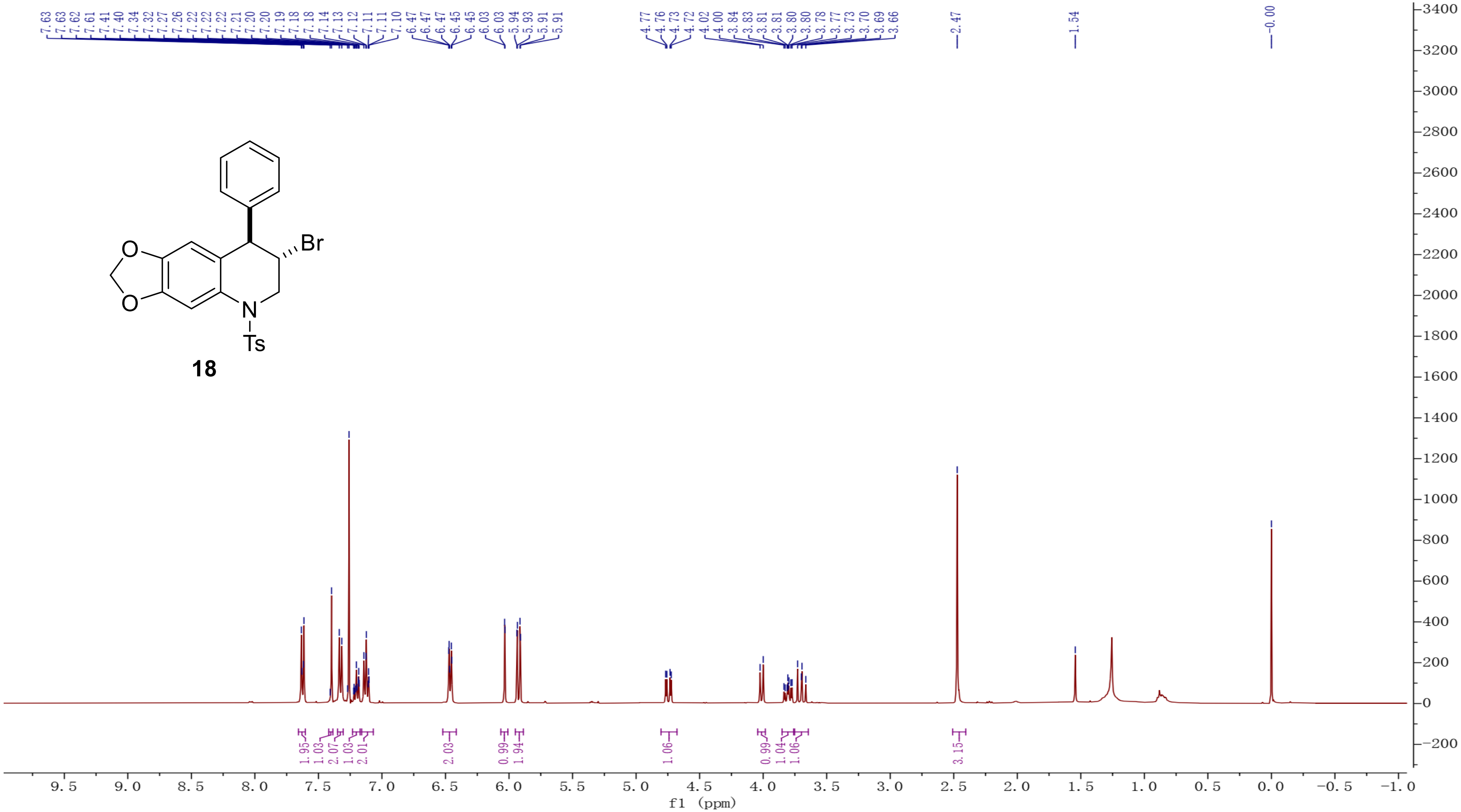

Supplementary Figure 59. ^{1}H NMR spectra of compound **18**.

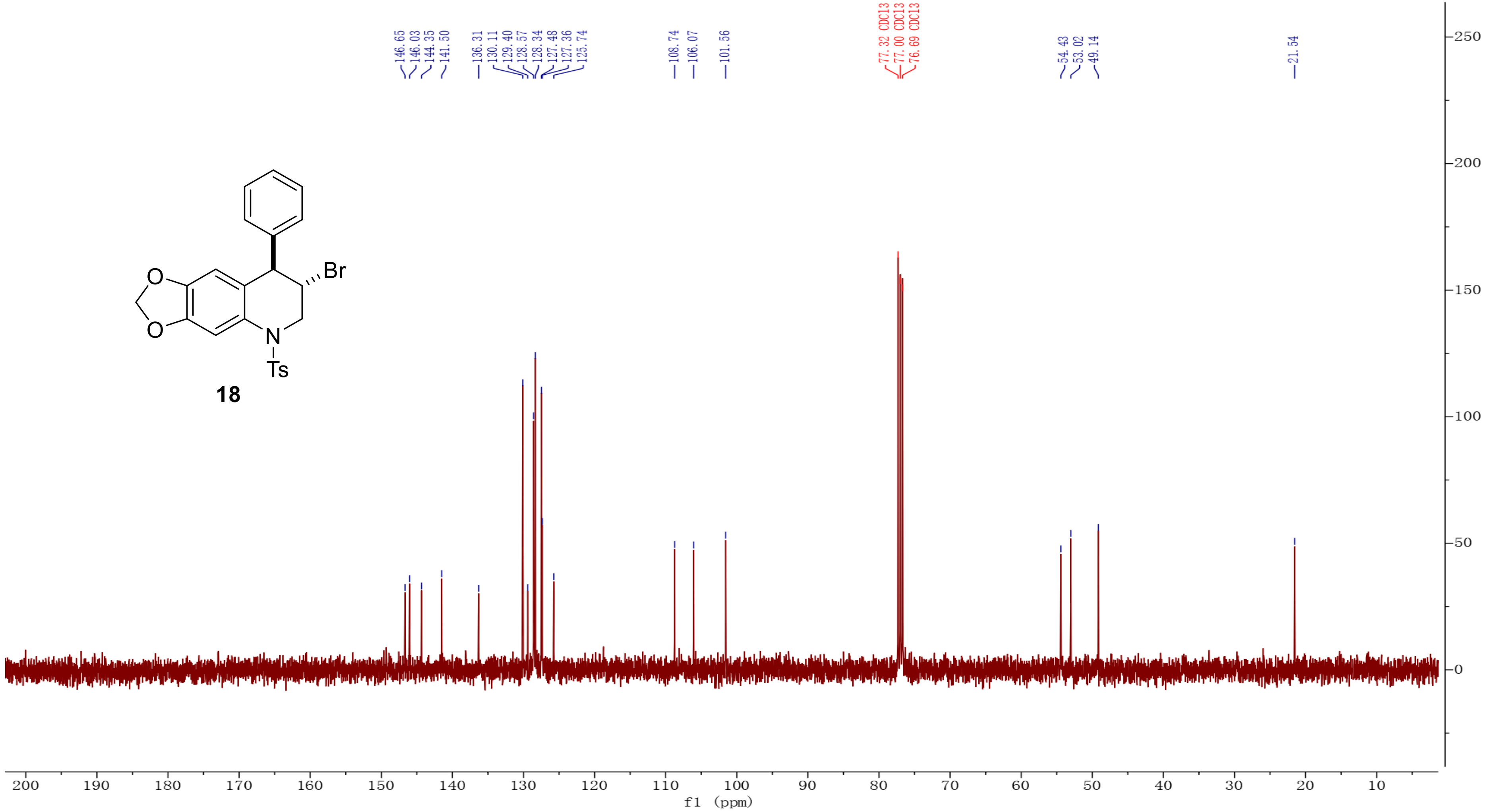

Supplementary Figure 60. ^{13}C NMR spectra of compound **18**.

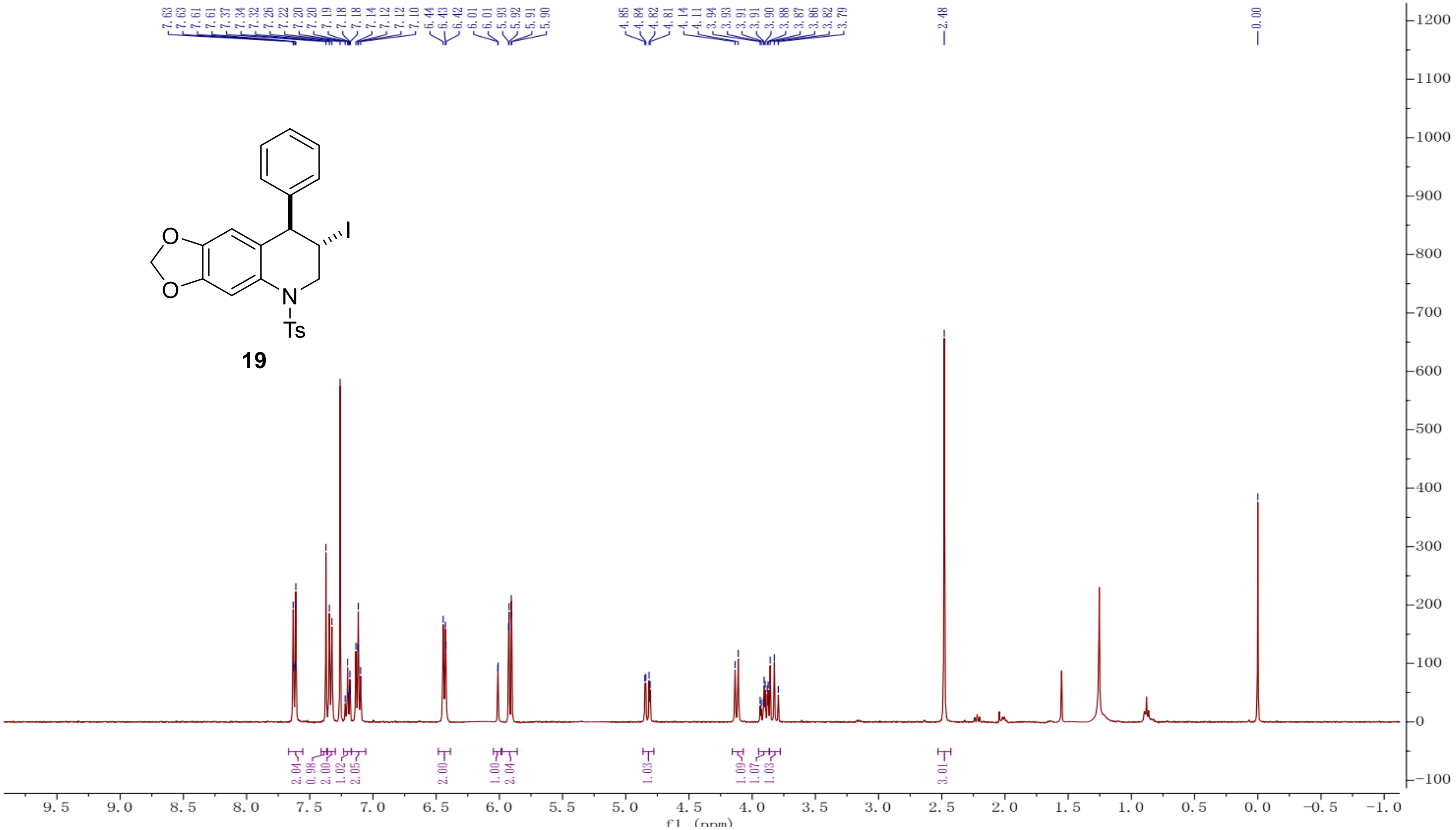

Supplementary Figure 61. ^{1}H NMR spectra of compound **19**.

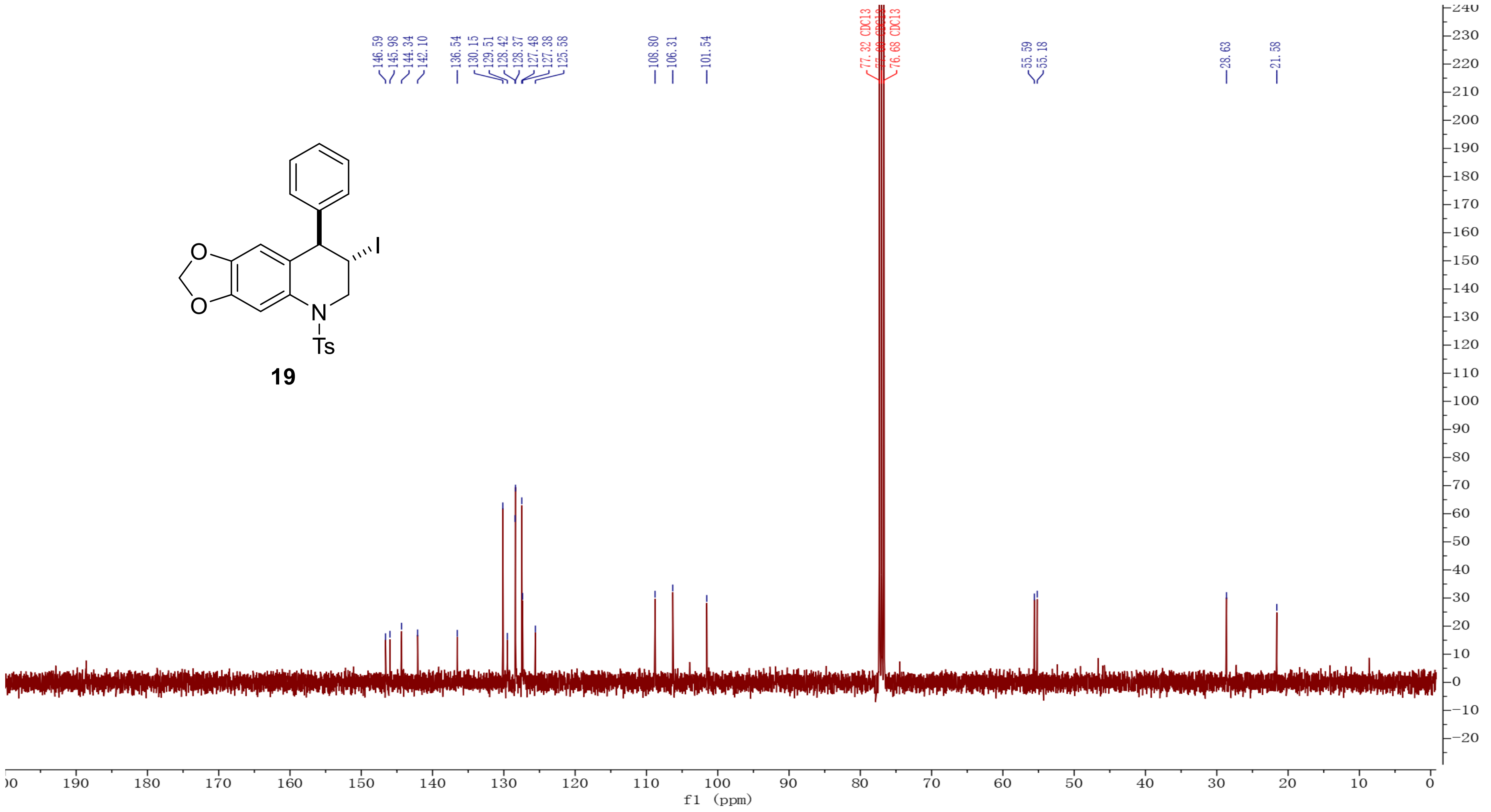

Supplementary Figure 62. ^{13}C NMR spectra of compound **19**.

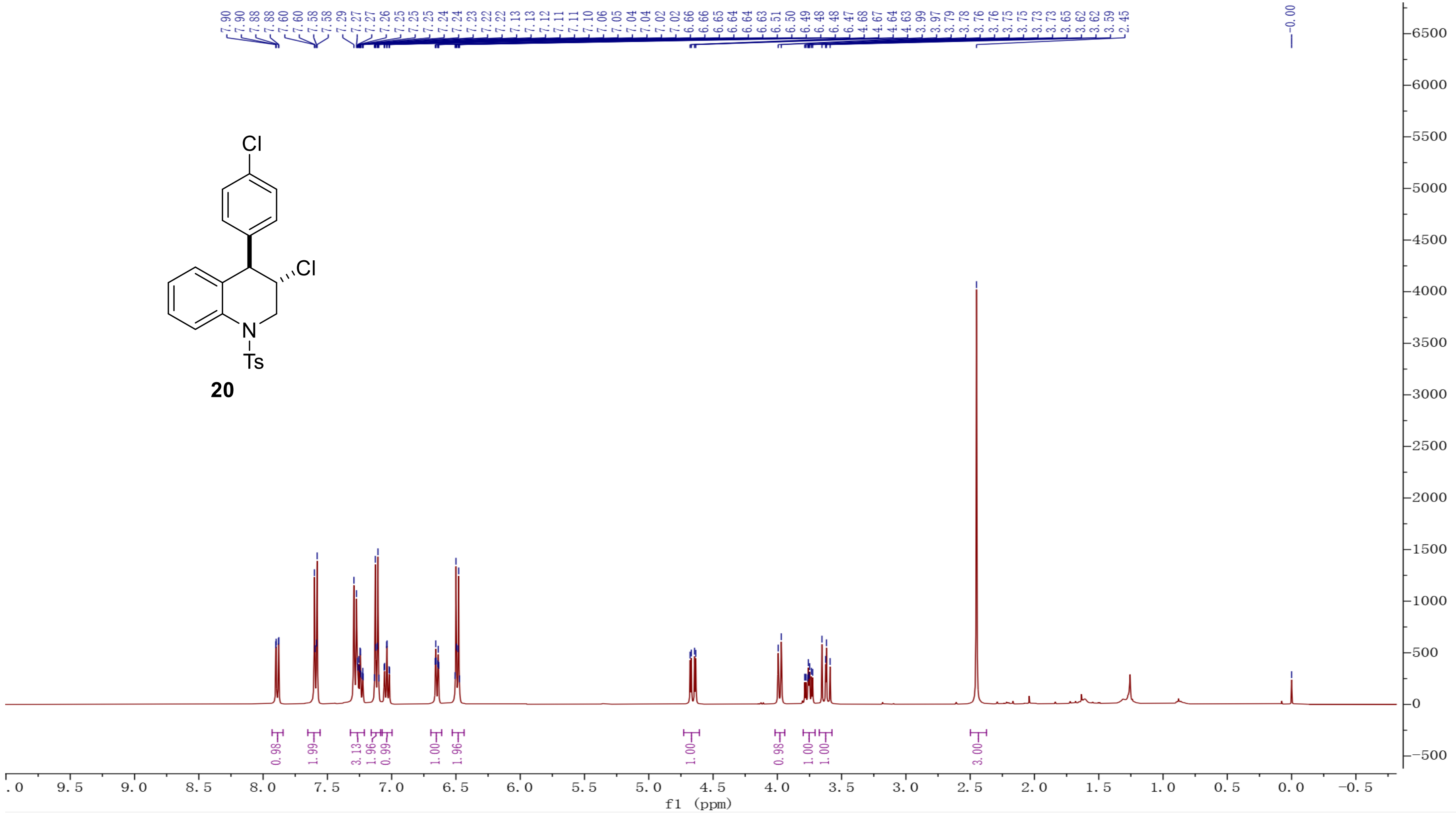

Supplementary Figure 63. ^1H NMR spectra of compound **20**.

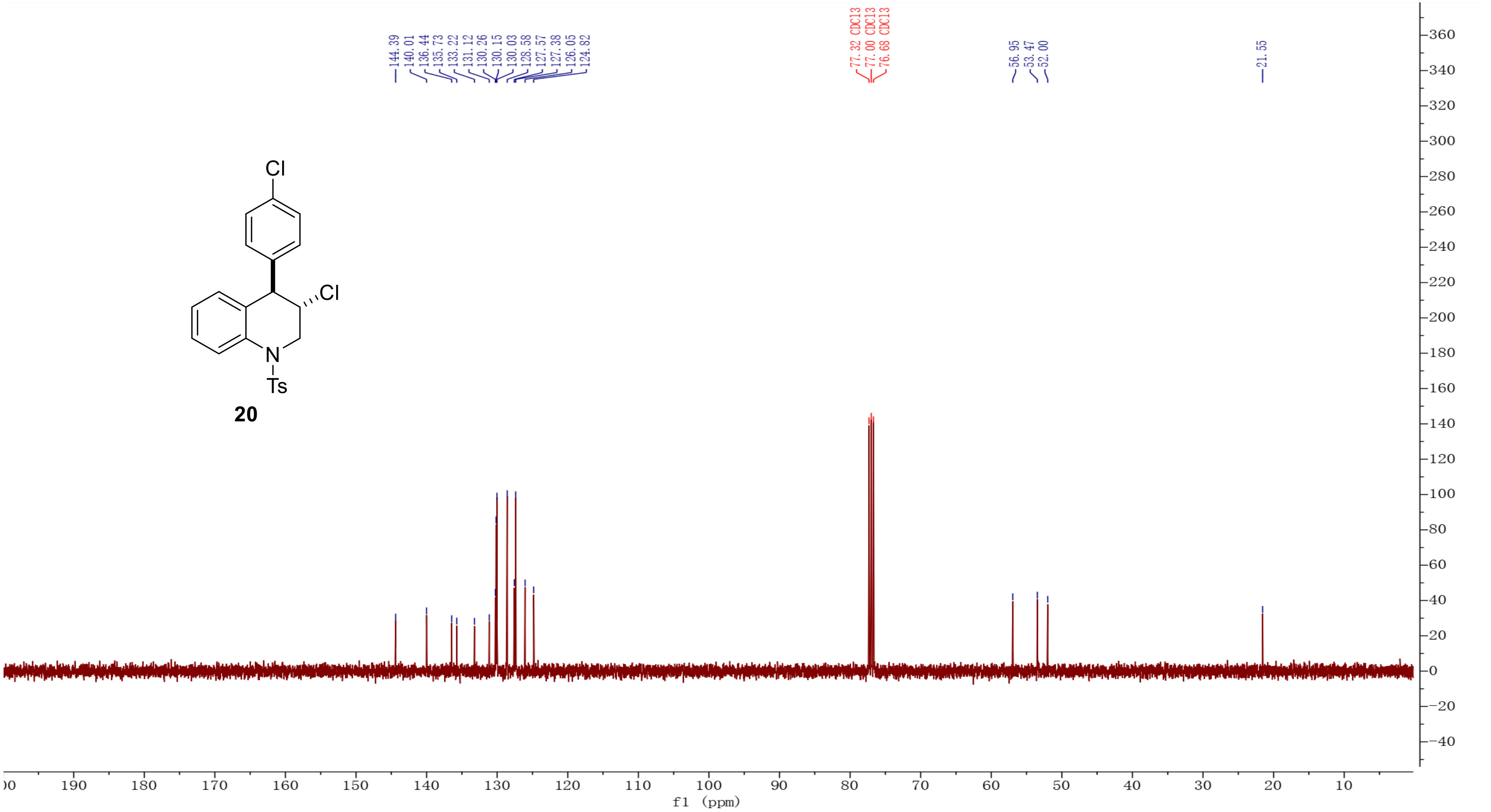

Supplementary Figure 64. ^{13}C NMR spectra of compound **20**.

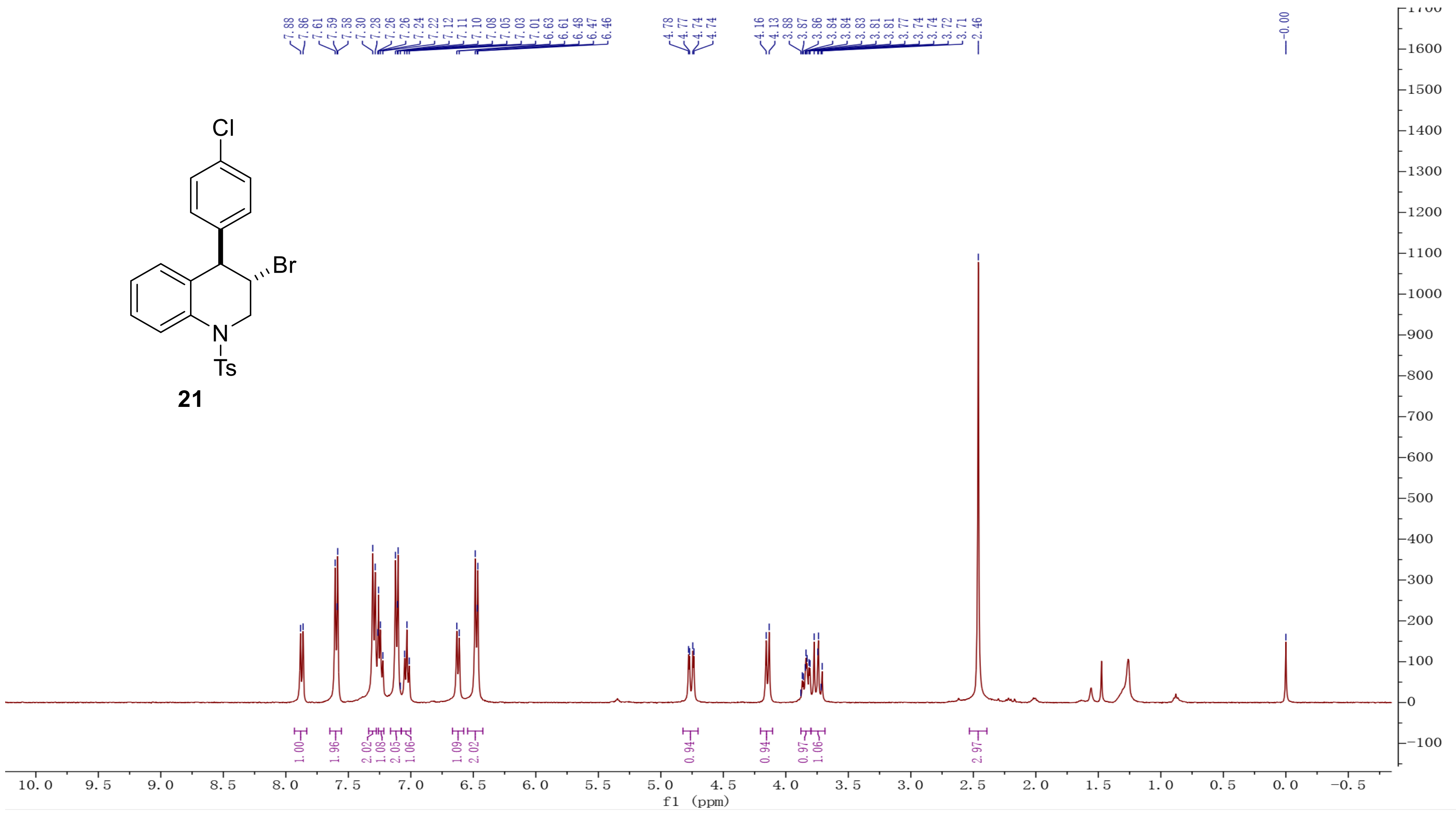

Supplementary Figure 65. ^{1}H NMR spectra of compound **21**.

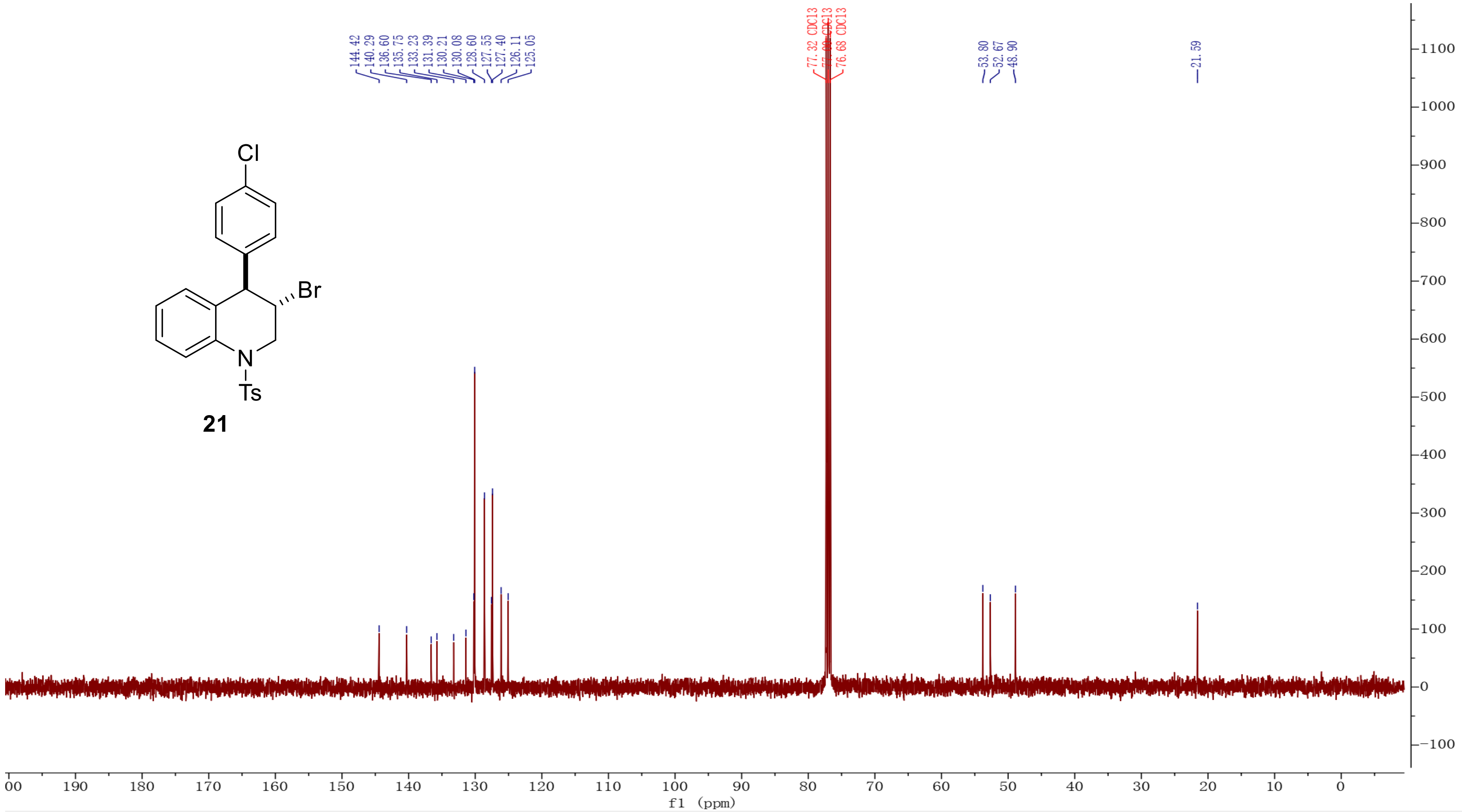

Supplementary Figure 66. ^{13}C NMR spectra of compound **21**.

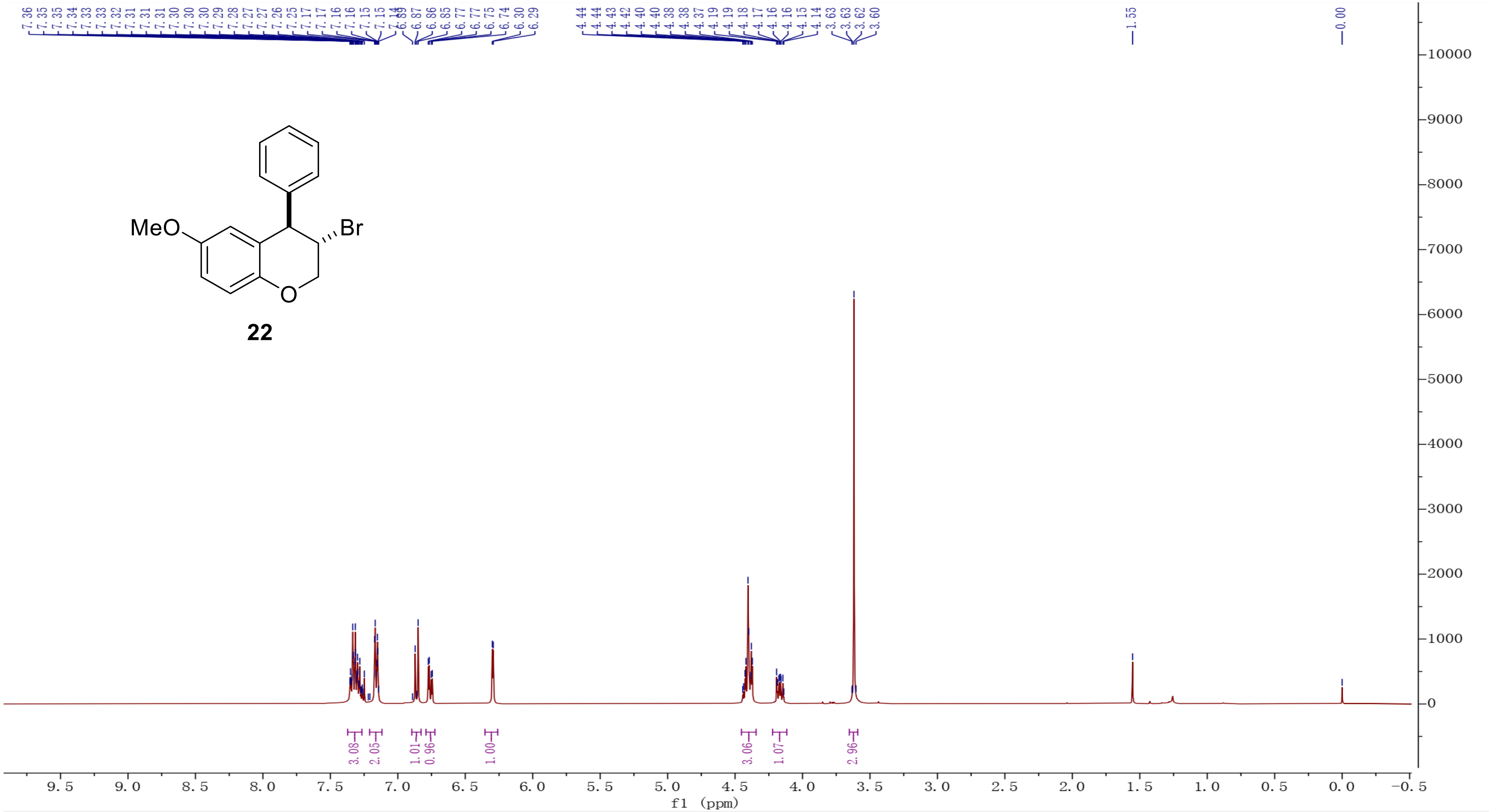

Supplementary Figure 67. ^{1}H NMR spectra of compound **22**.

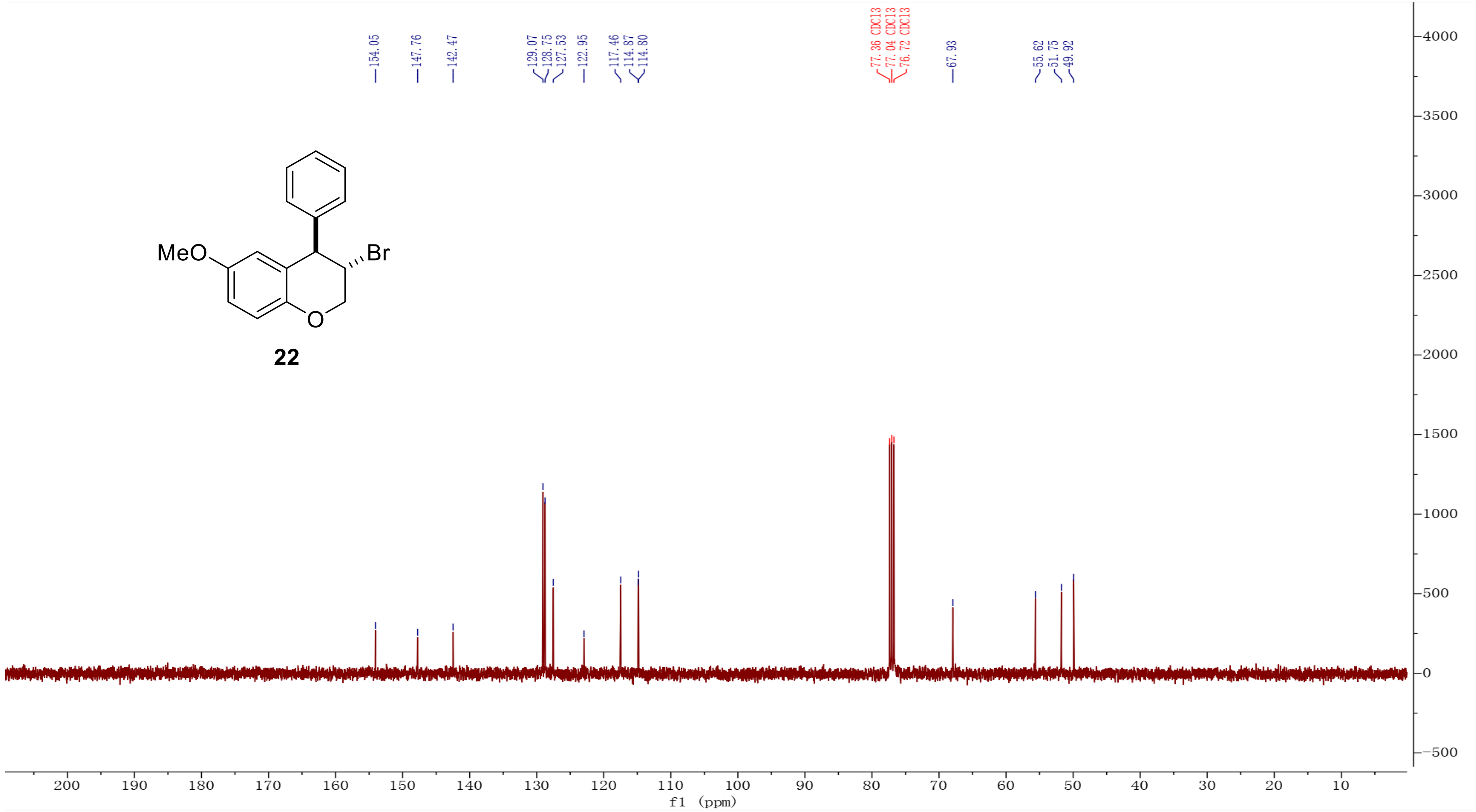

Supplementary Figure 68. ^{13}C NMR spectra of compound **22**.

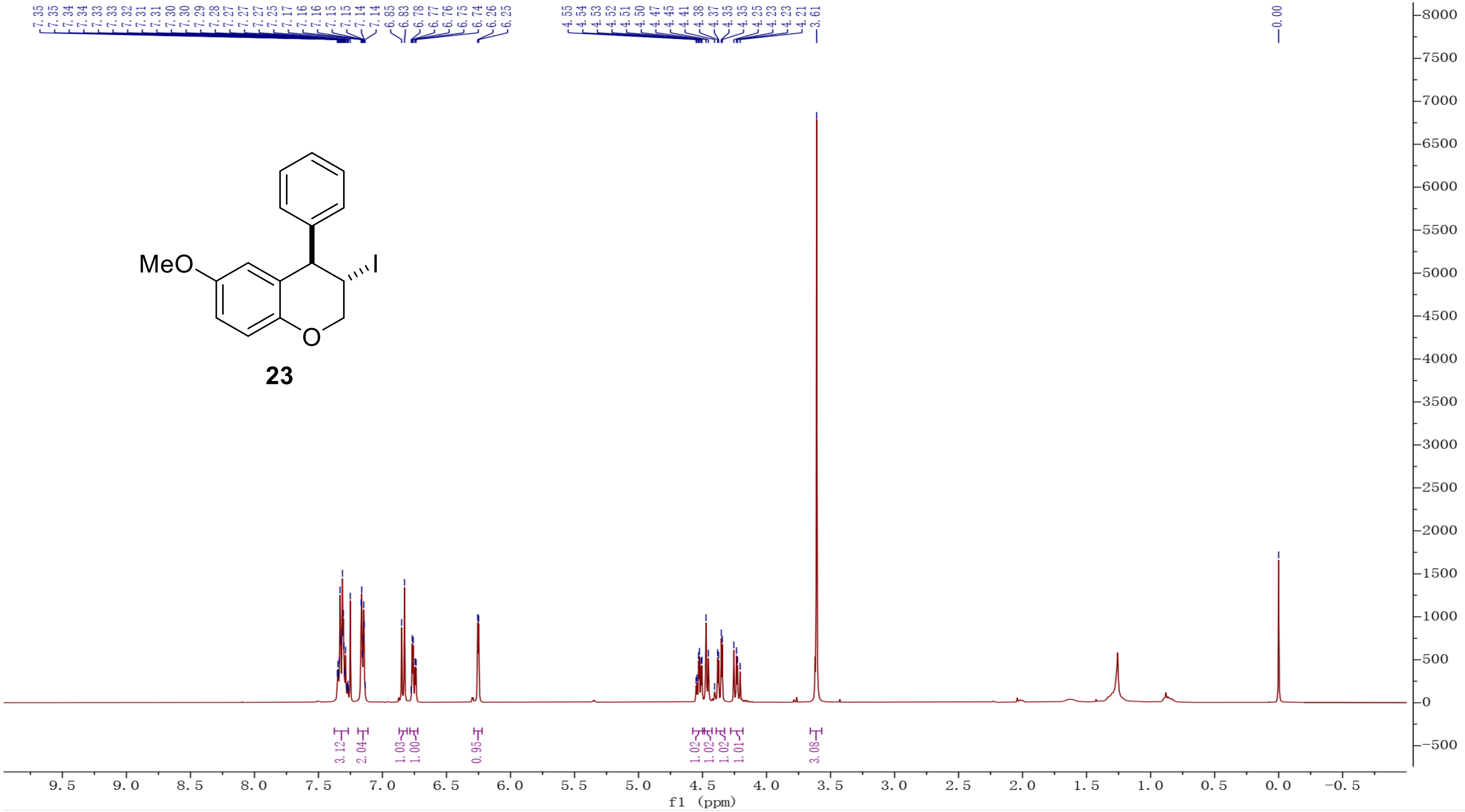

Supplementary Figure 69. ^{1}H NMR spectra of compound **23**.

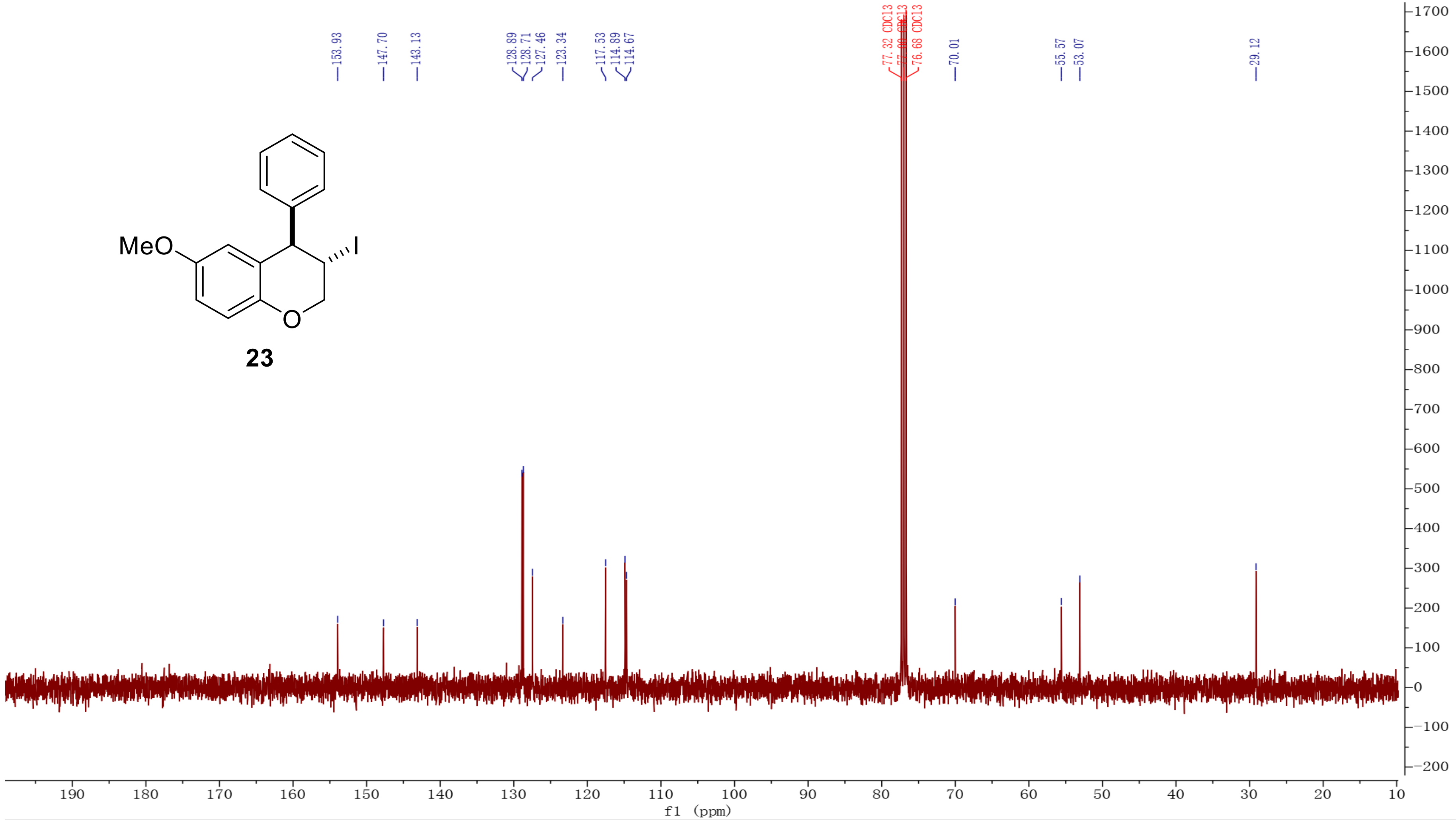

Supplementary Figure 70. ^{13}C NMR spectra of compound **23**.

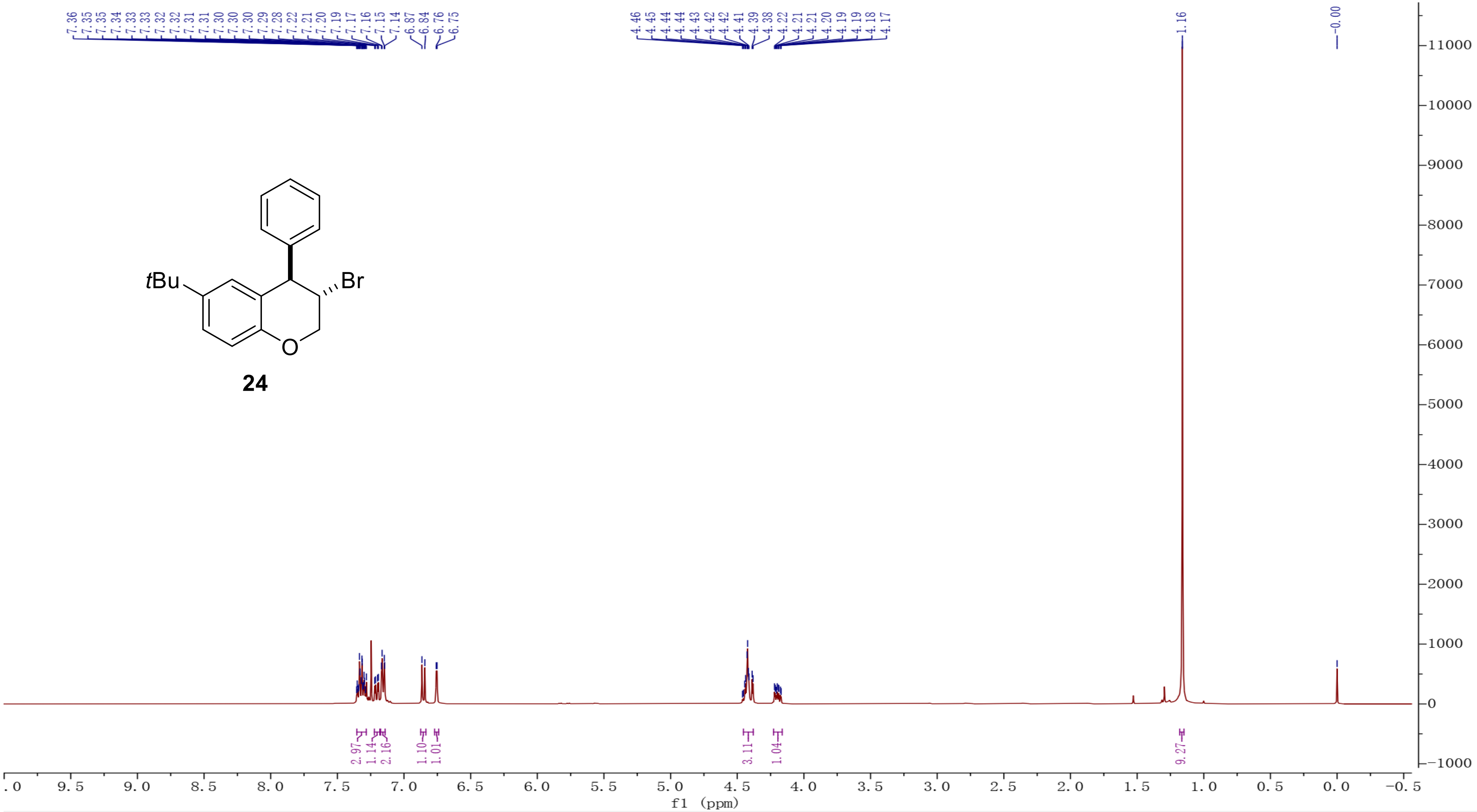

Supplementary Figure 71. ^{1}H NMR spectra of compound **24**.

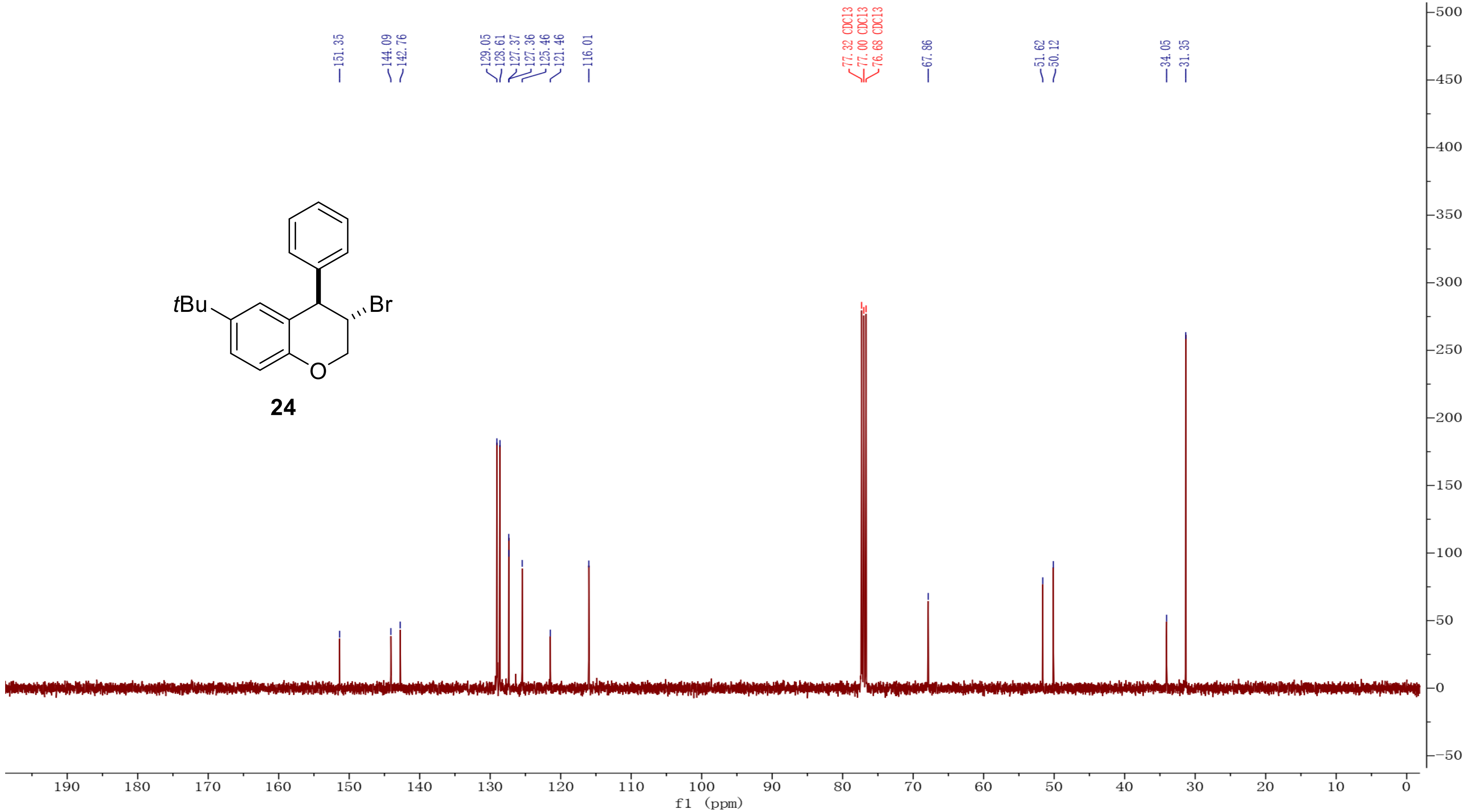

Supplementary Figure 72. ^{13}C NMR spectra of compound **24**.

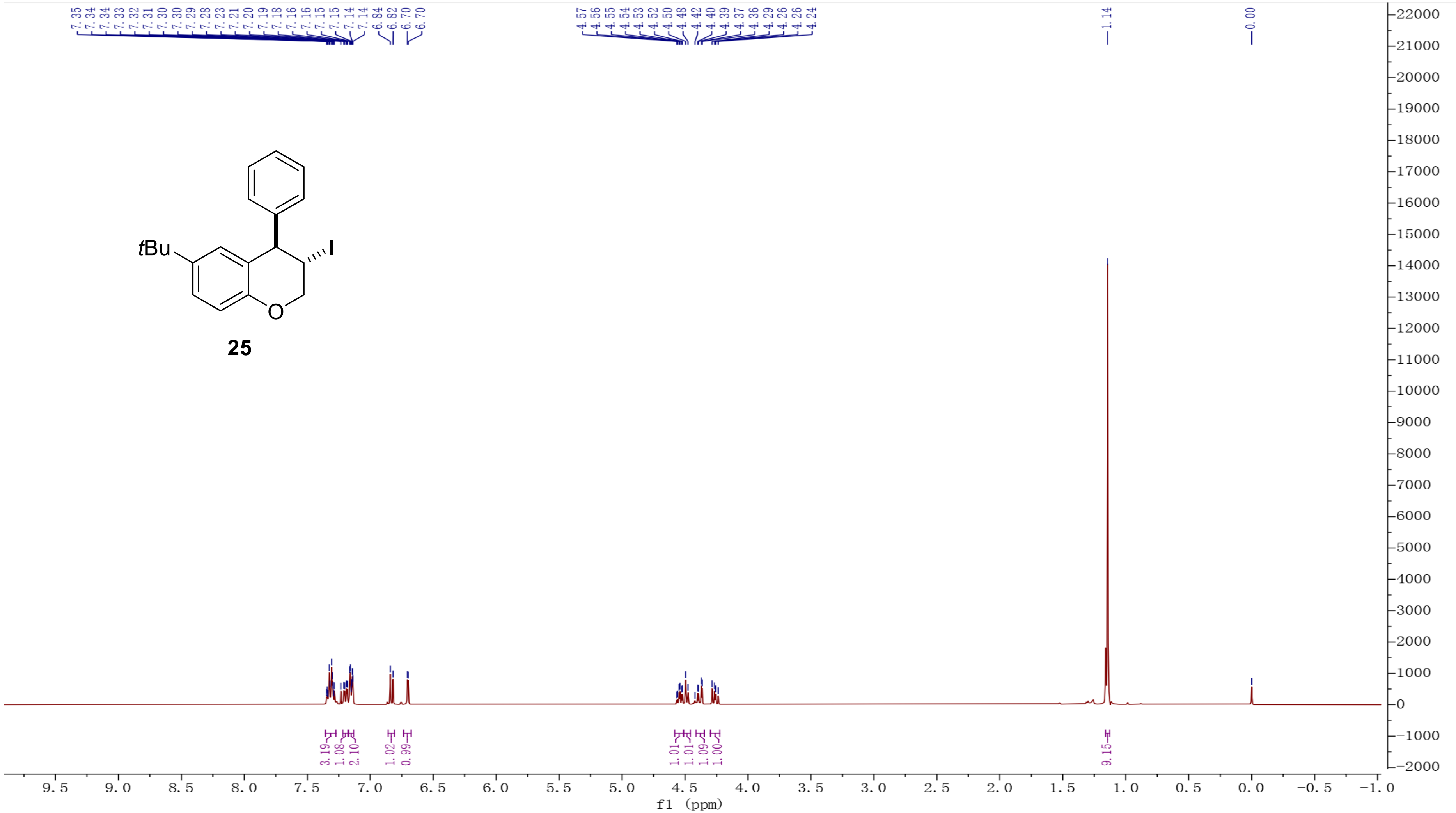

25

Supplementary Figure 73. ^{1}H NMR spectra of compound **25**.

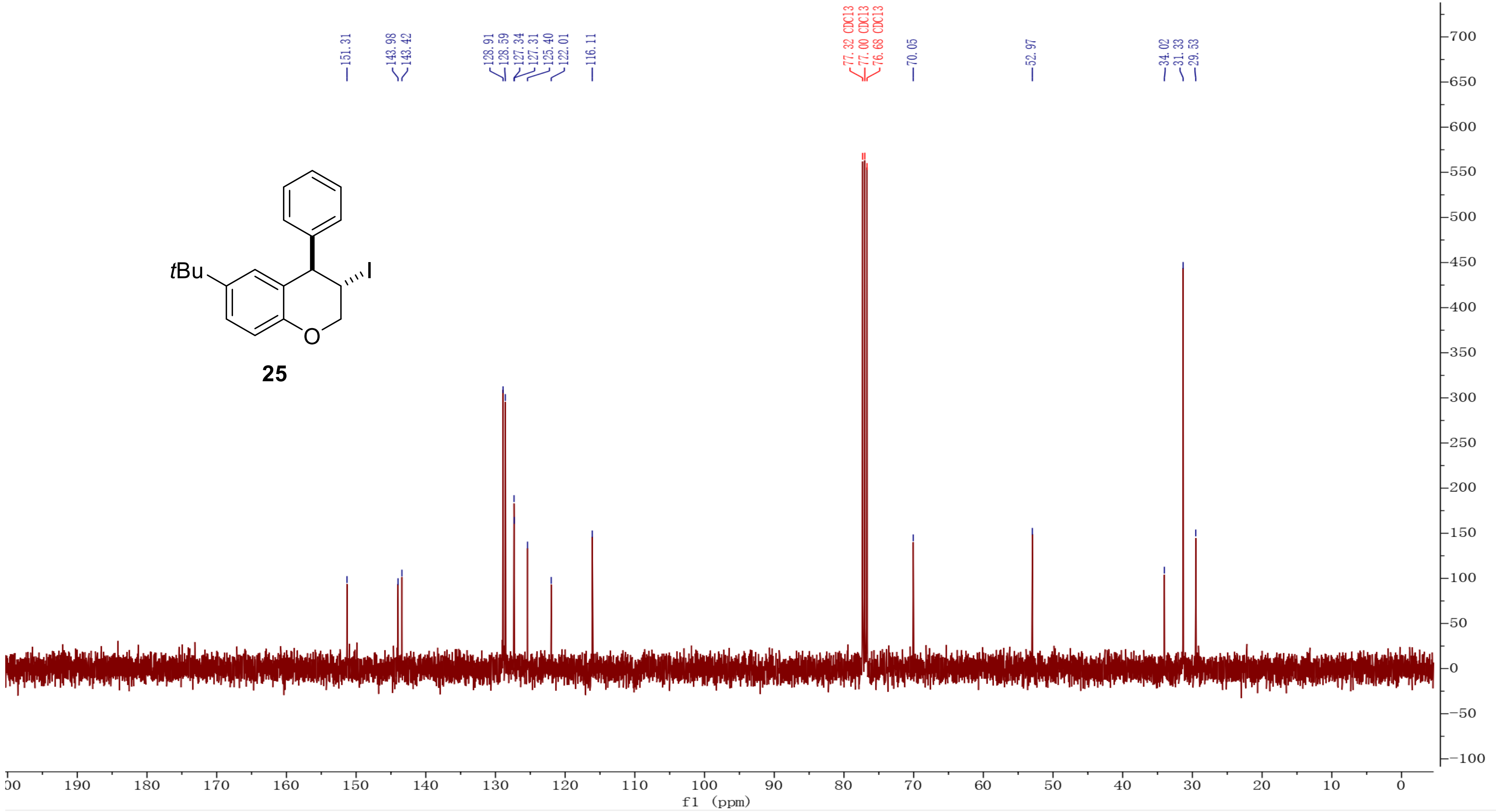

Supplementary Figure 74. ^{13}C NMR spectra of compound **25**.

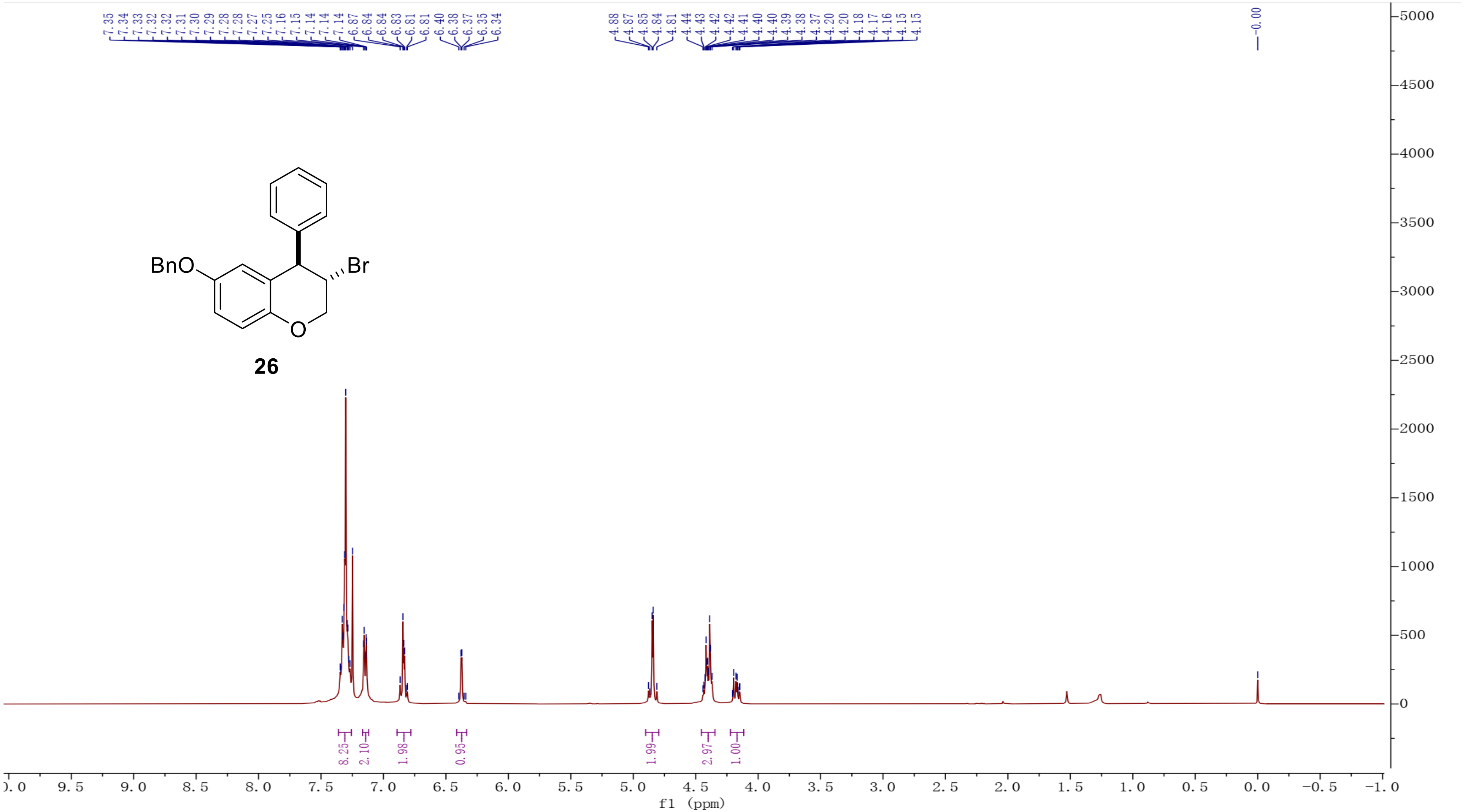

Supplementary Figure 75. ^{1}H NMR spectra of compound **26**.

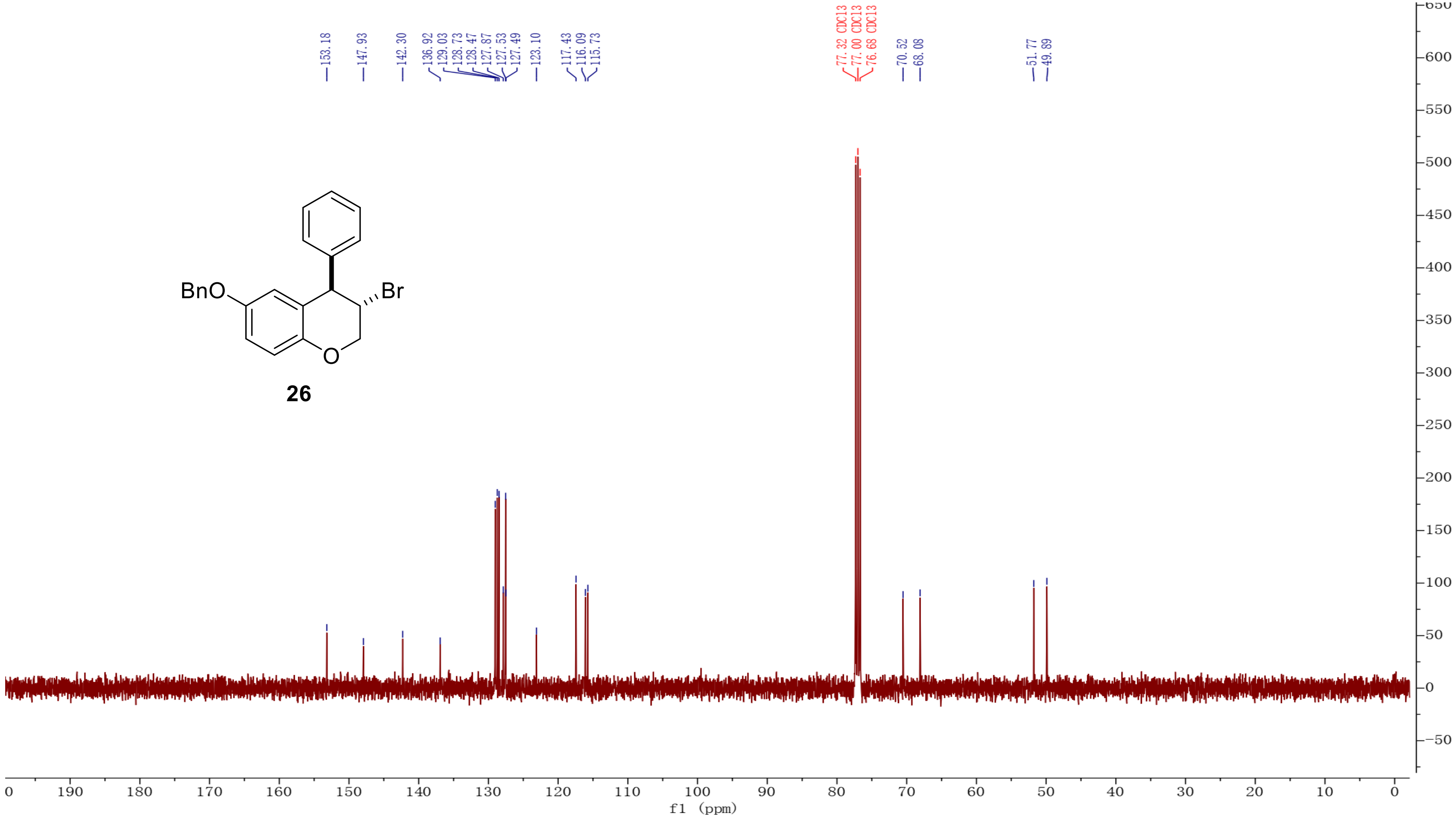

Supplementary Figure 76. ^{13}C NMR spectra of compound **26**.

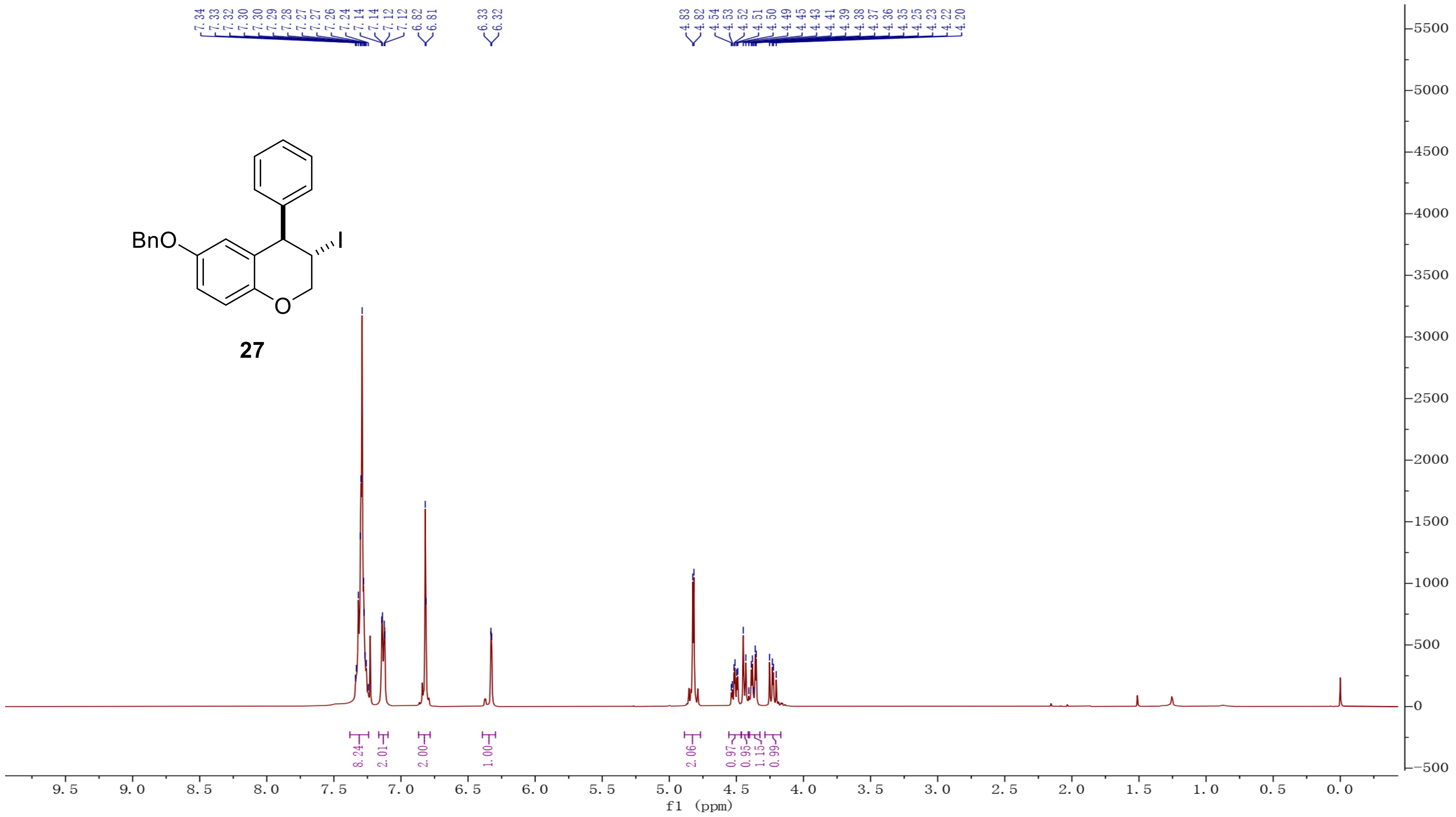

Supplementary Figure 77. ^{1}H NMR spectra of compound **27**.

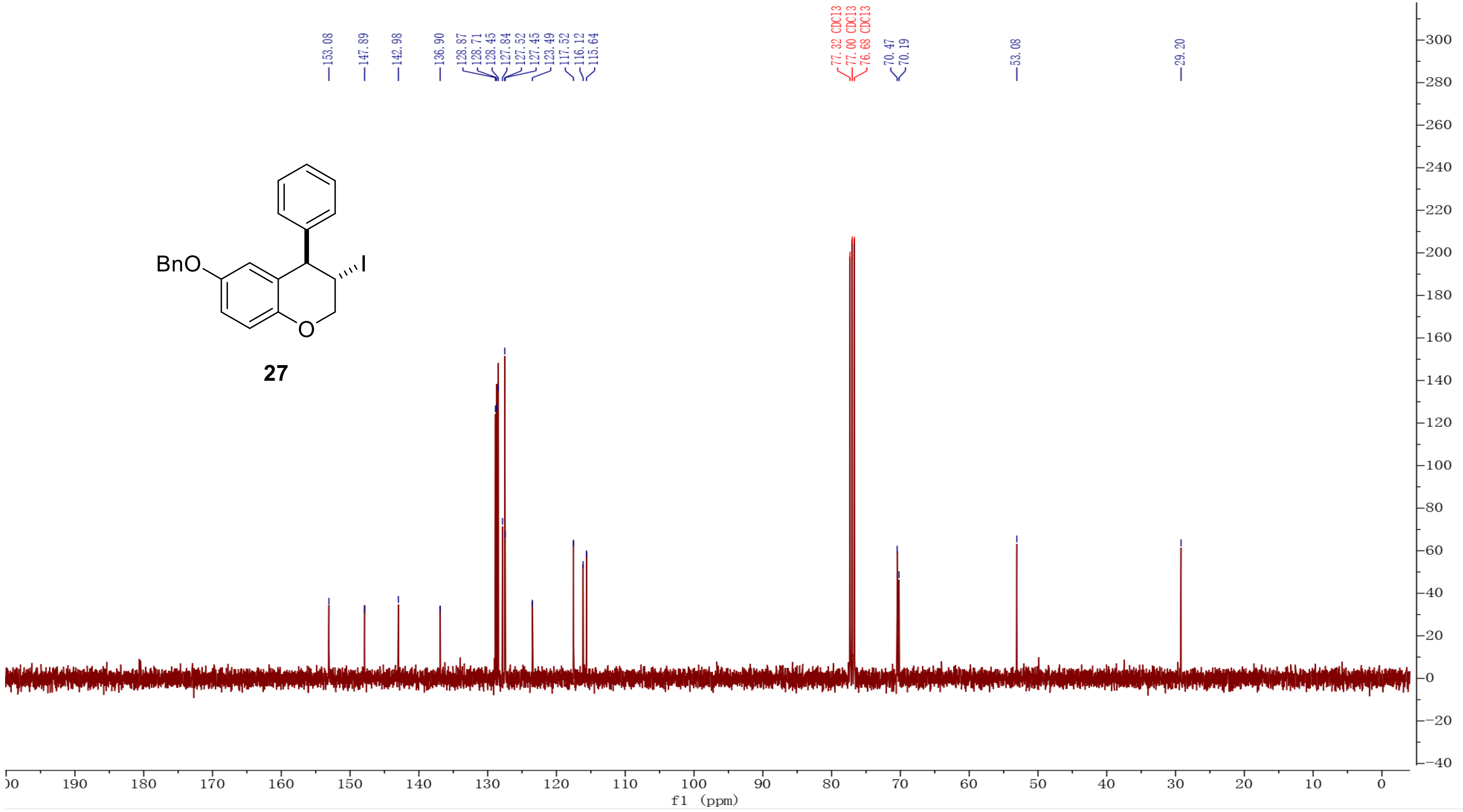

Supplementary Figure 78. ^{13}C NMR spectra of compound **27**.

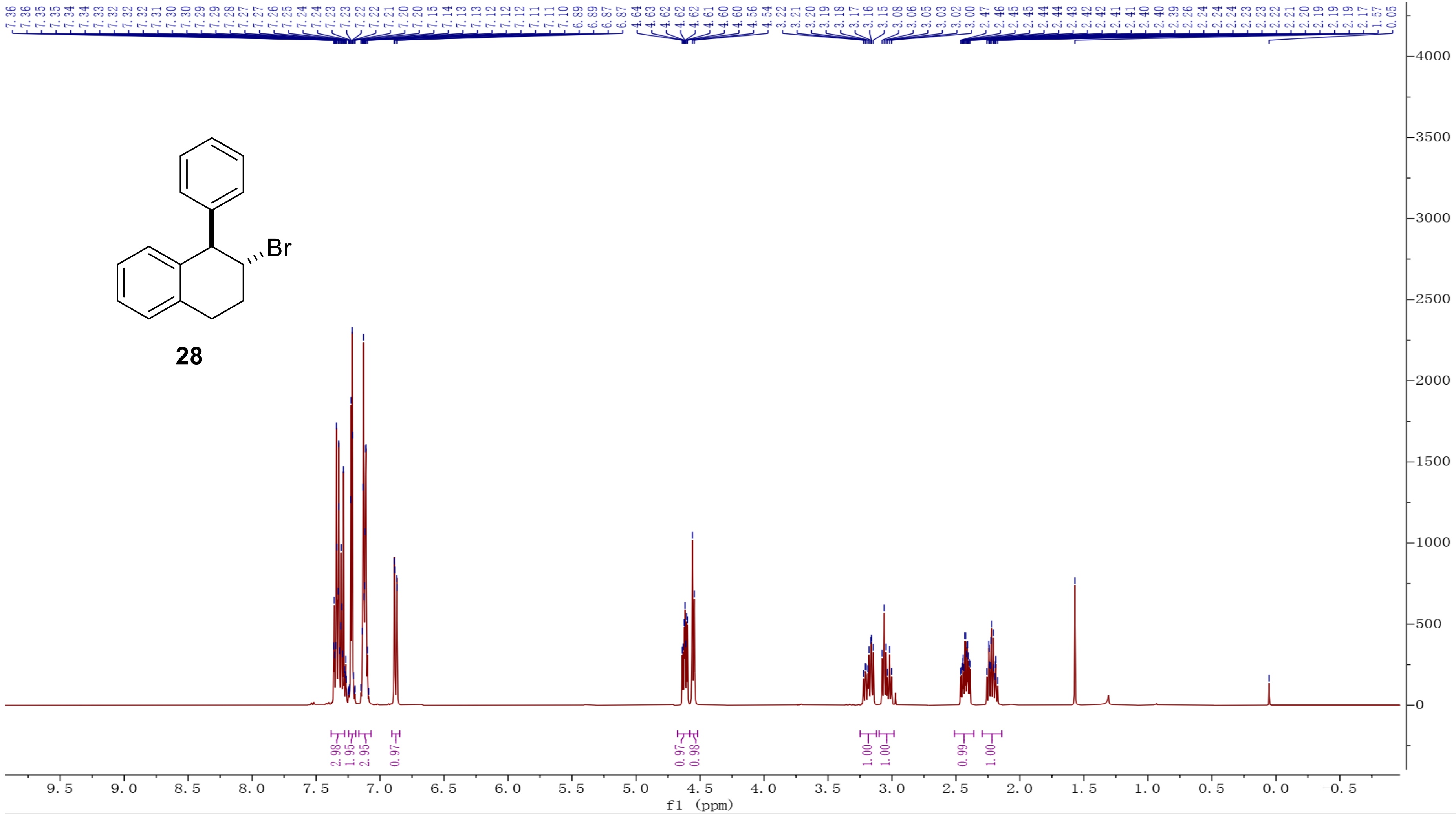

28

Supplementary Figure 79. ^{1}H NMR spectra of compound **28**.

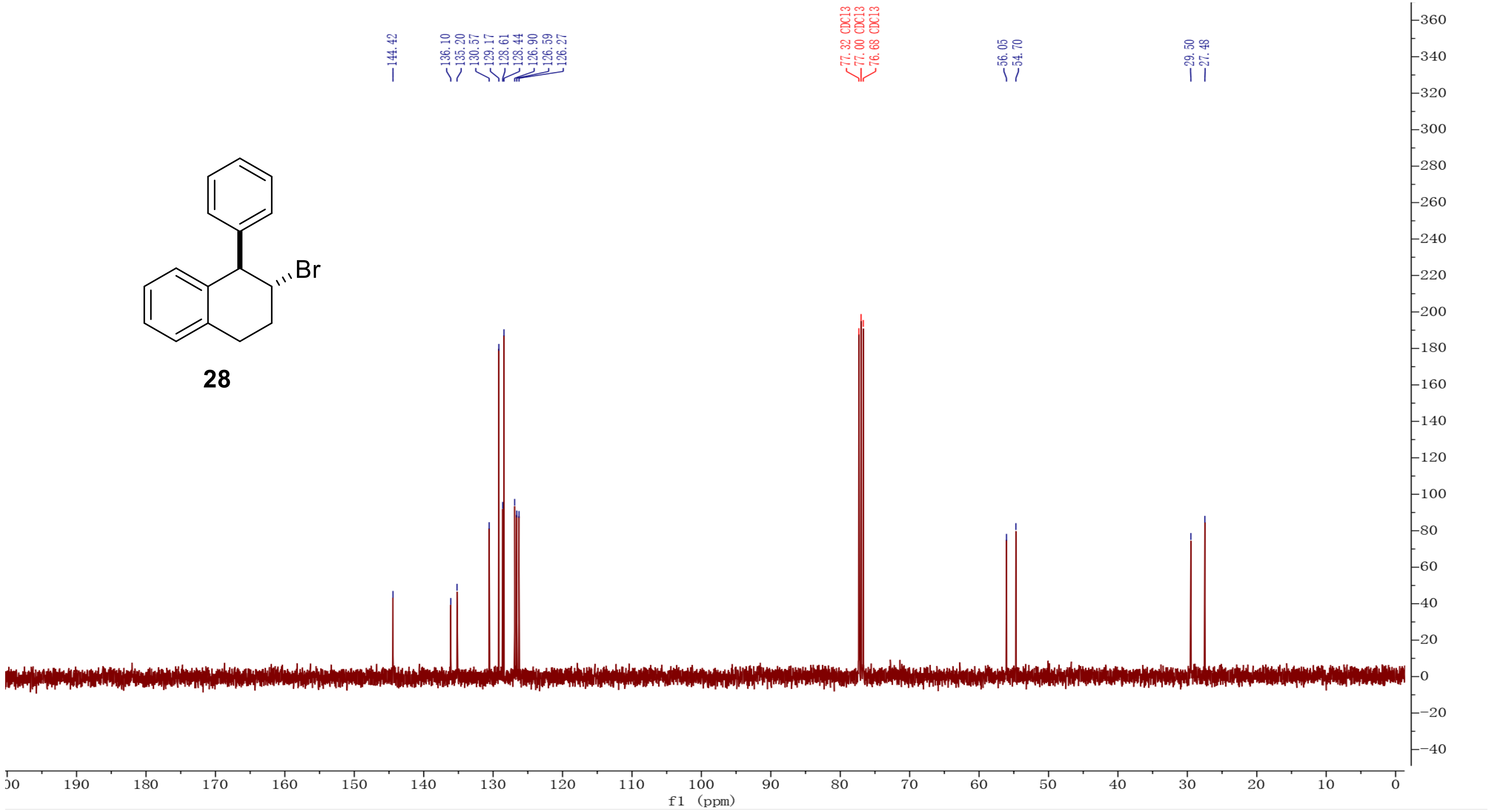

Supplementary Figure 80. ^{13}C NMR spectra of compound **28**.

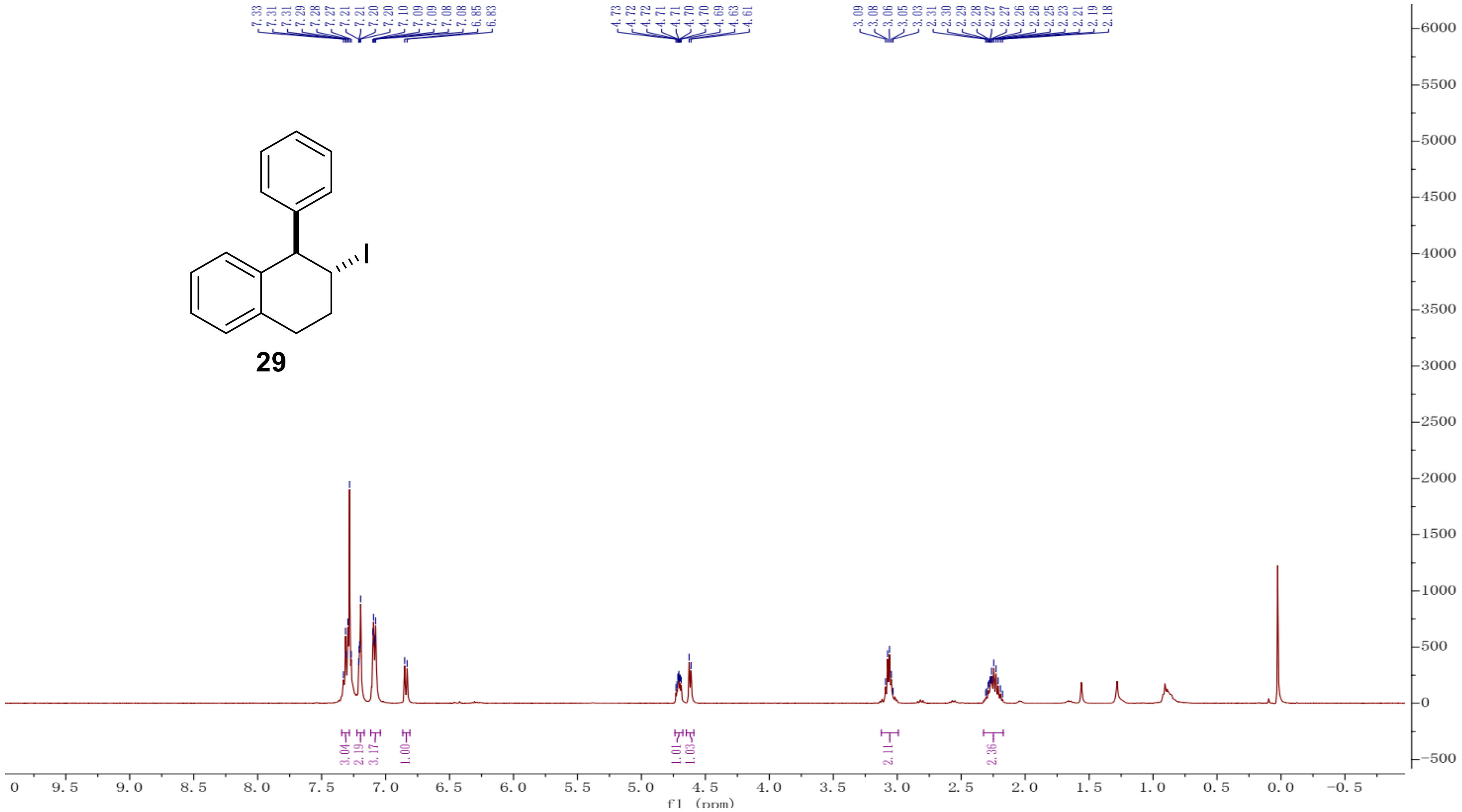

Supplementary Figure 81. ^{1}H NMR spectra of compound **29**.

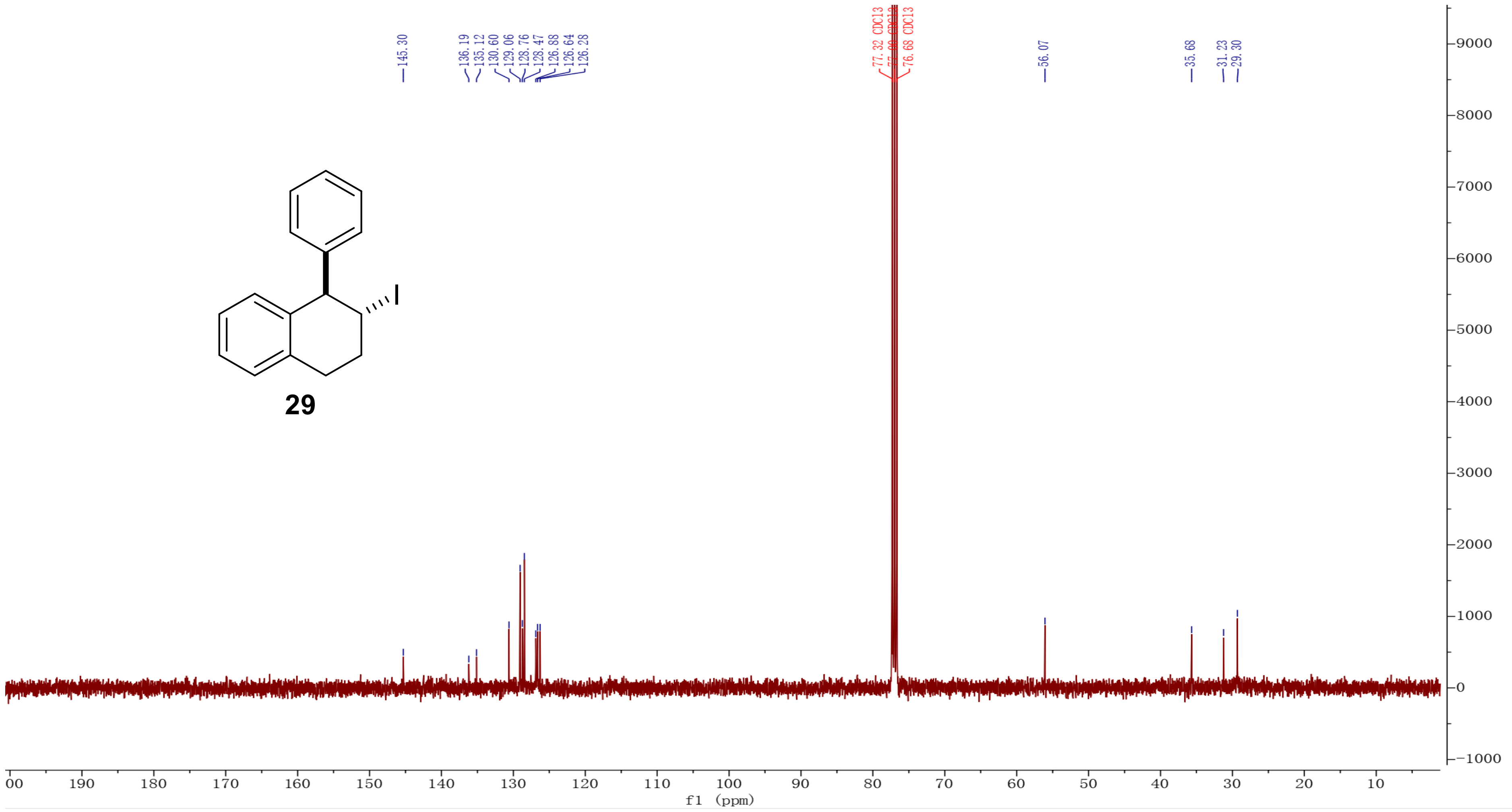

Supplementary Figure 82. ^{13}C NMR spectra of compound **29**.

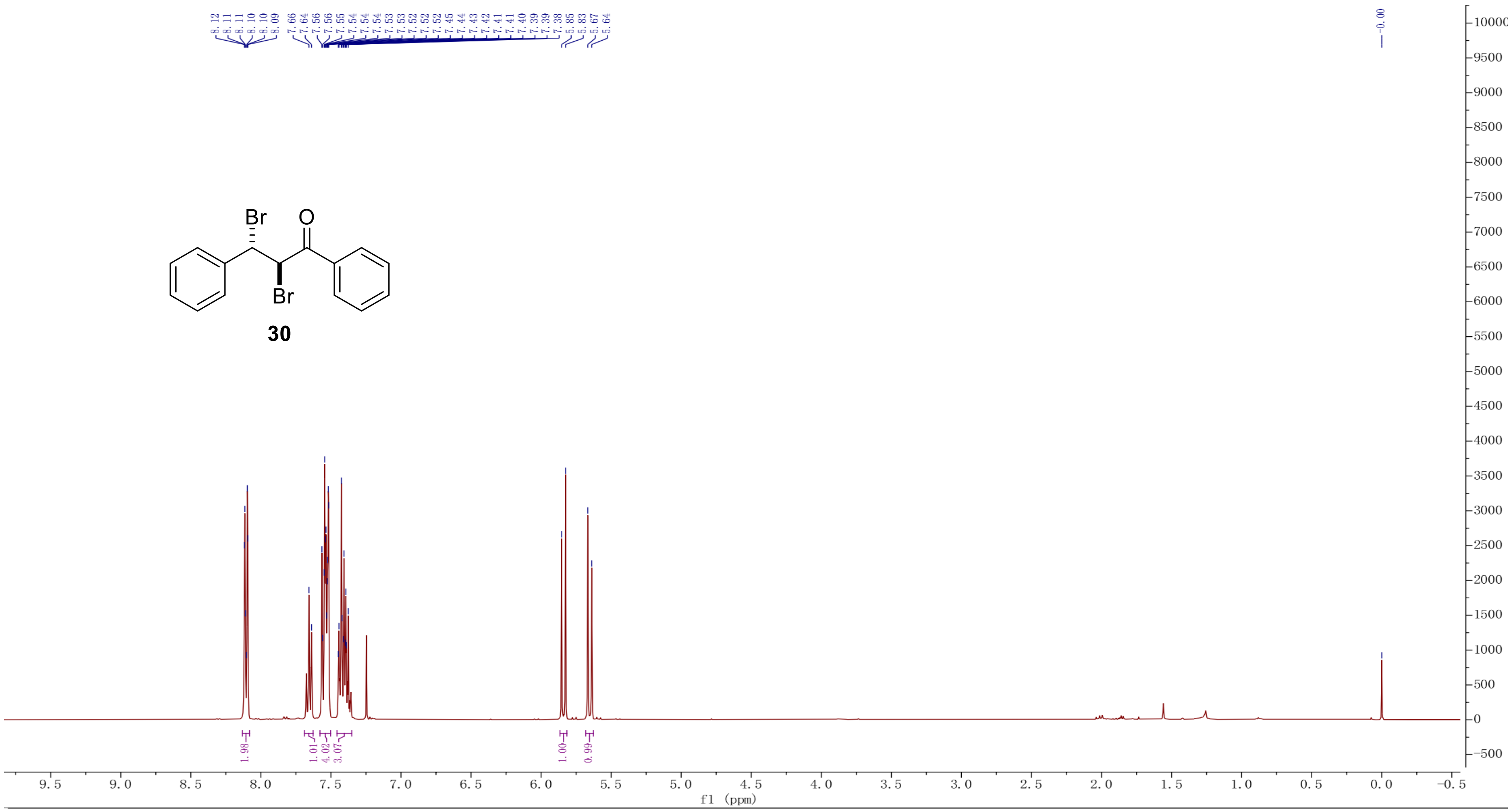

Supplementary Figure 83. ^{1}H NMR spectra of compound **30**.

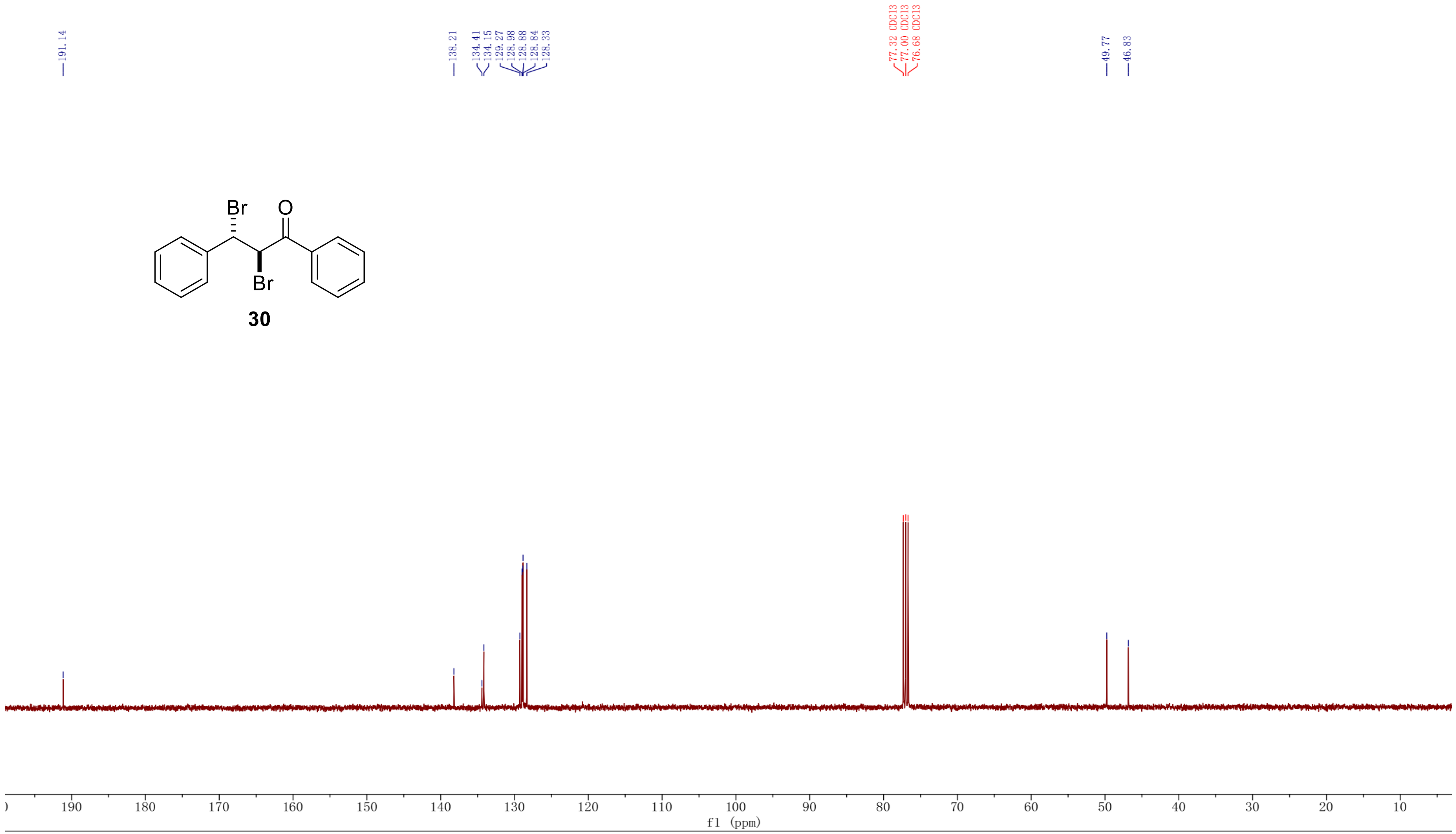

Supplementary Figure 84. ^{13}C NMR spectra of compound **30**.

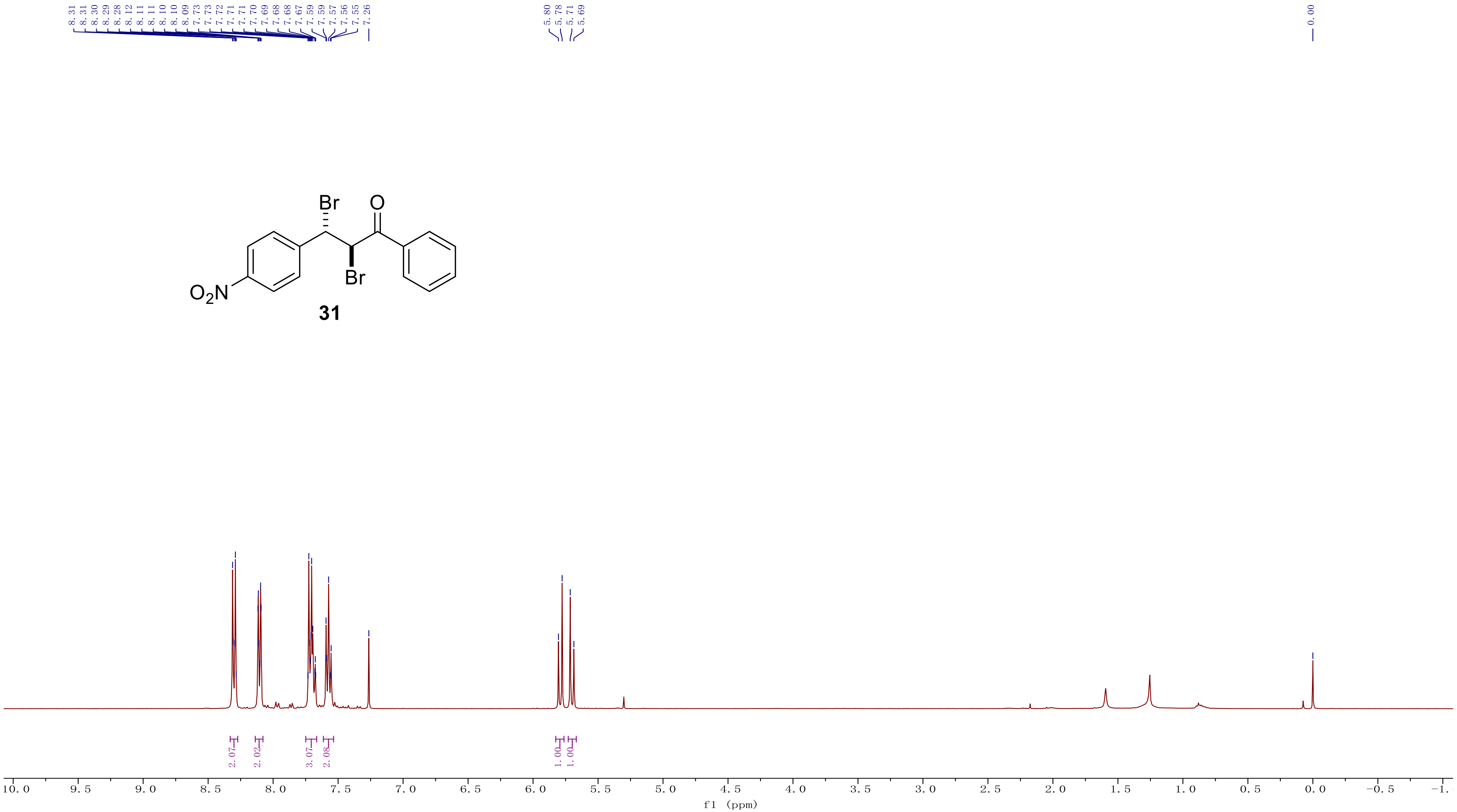

Supplementary Figure 85. ^{1}H NMR spectra of compound **31**.

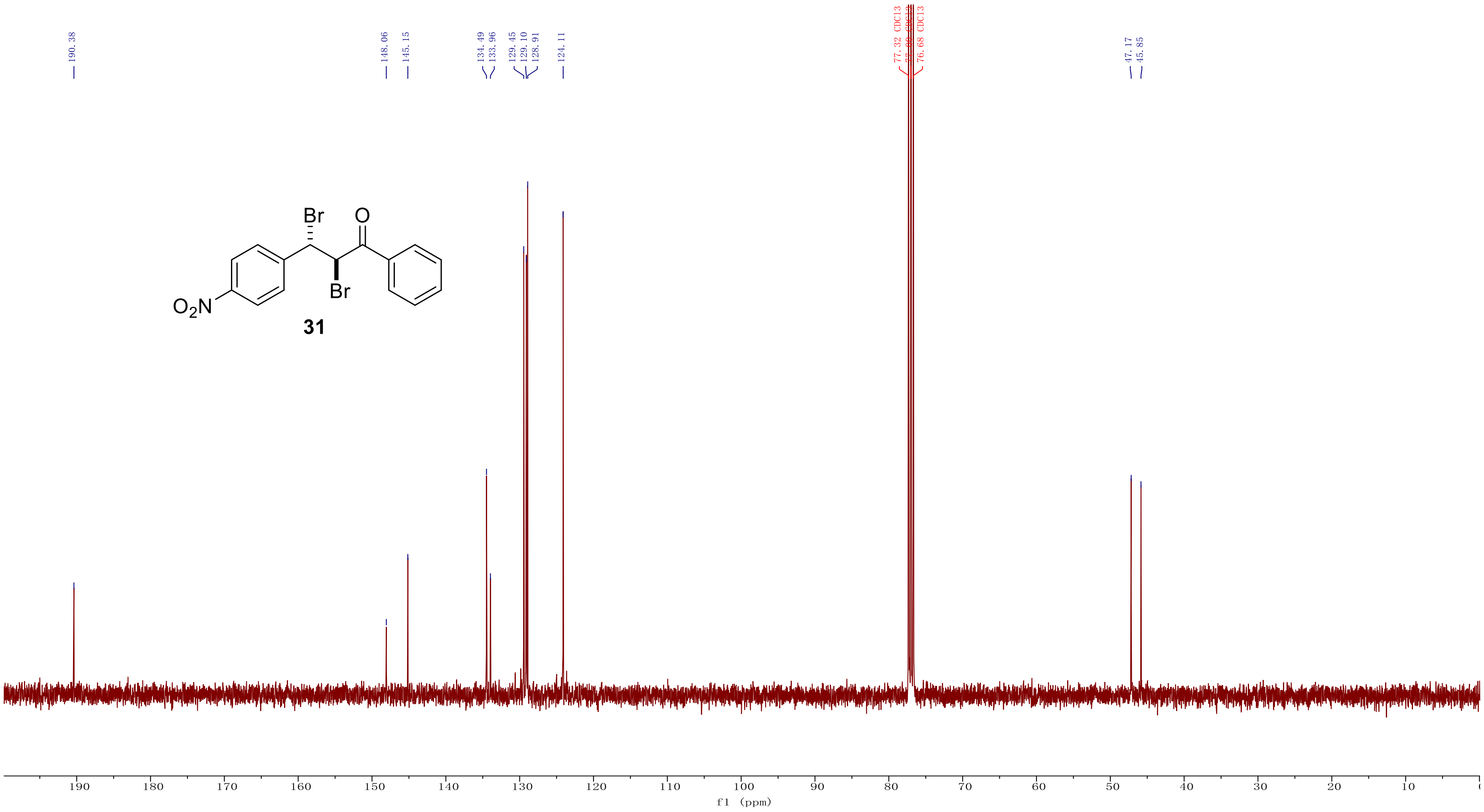

Supplementary Figure 86. ^{13}C NMR spectra of compound **31**.

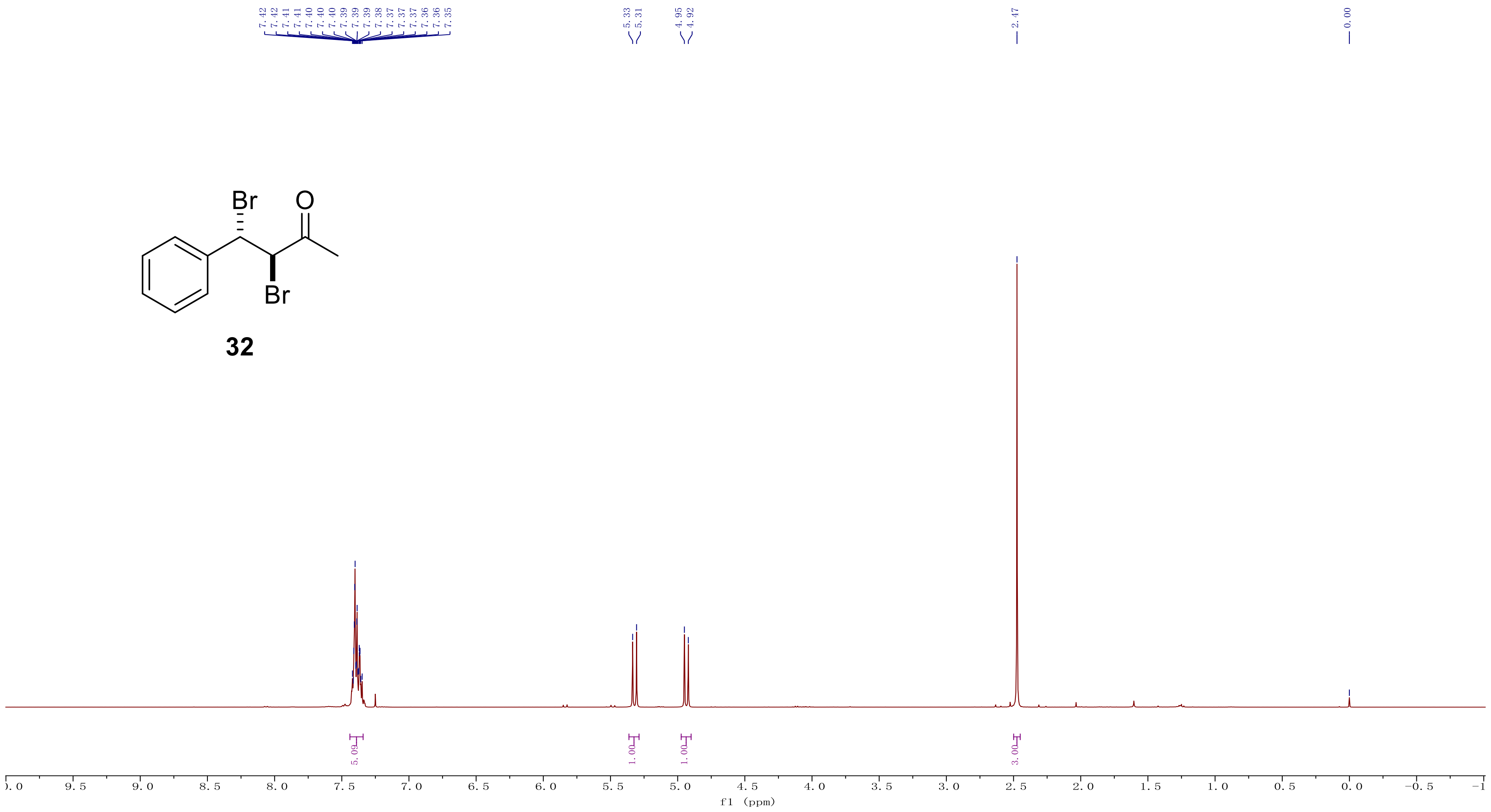

Supplementary Figure 87. ^{1}H NMR spectra of compound **32**.

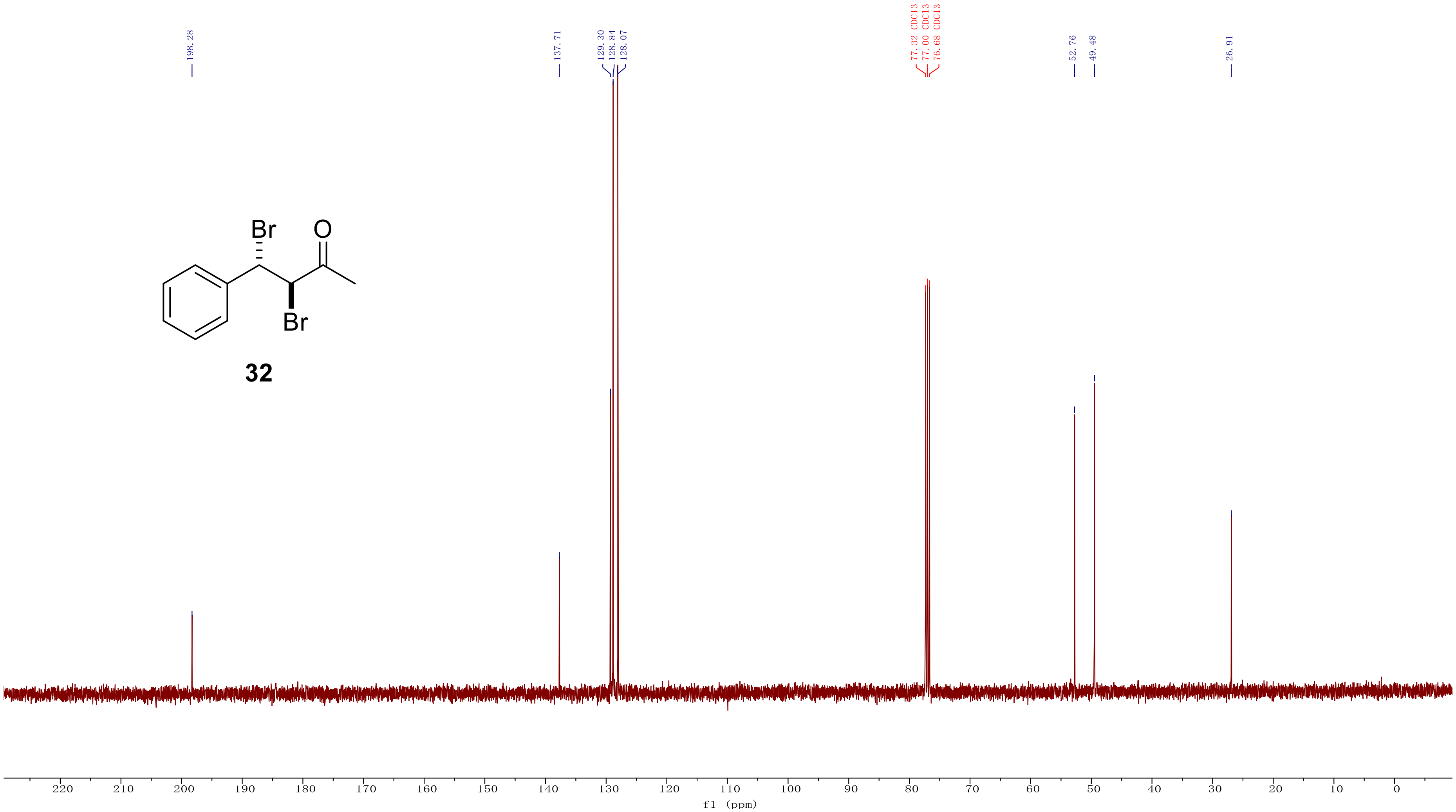

32

Supplementary Figure 88. ^{13}C NMR spectra of compound **32**.

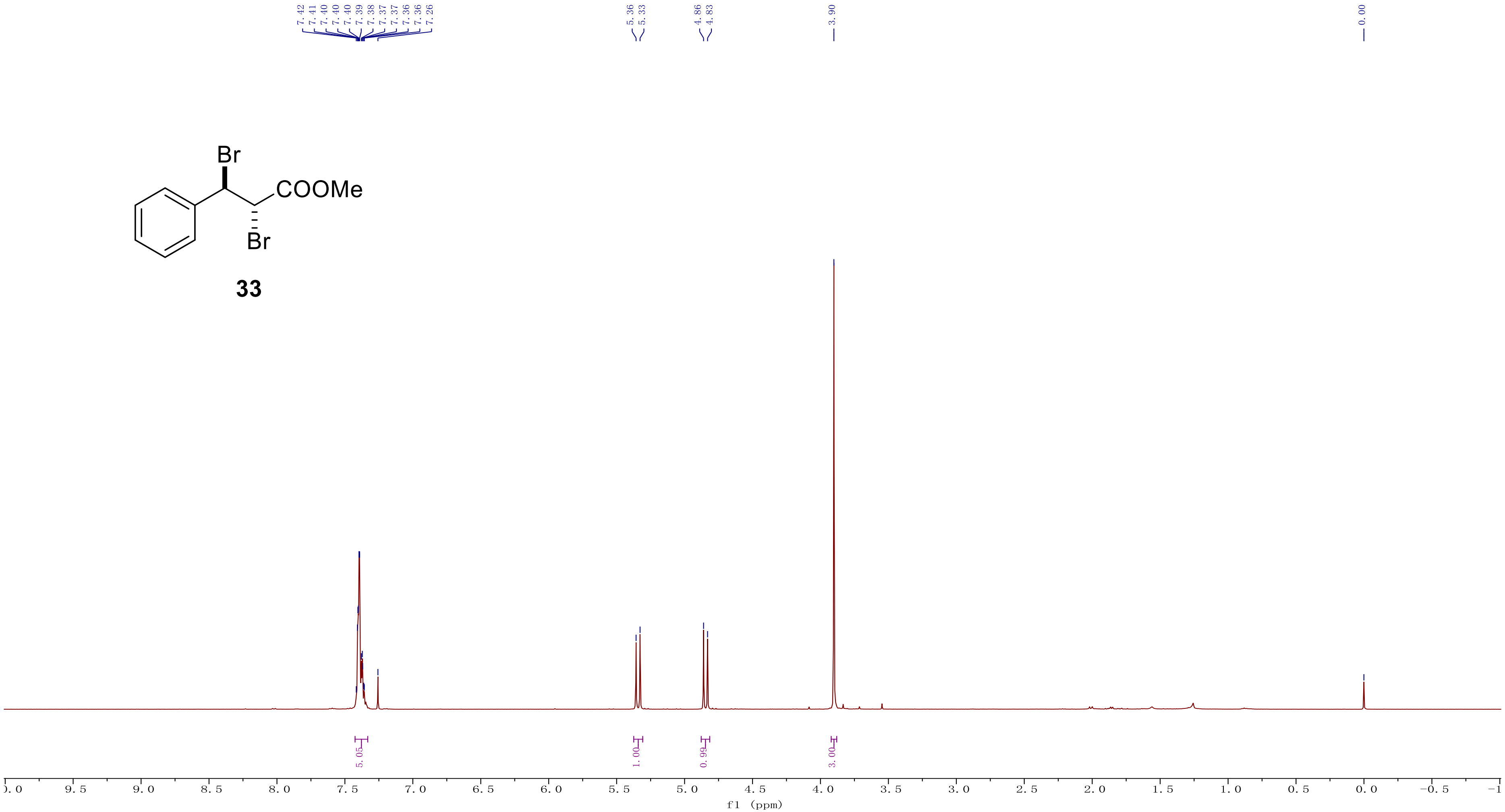

Supplementary Figure 89. ^{1}H NMR spectra of compound **33**.

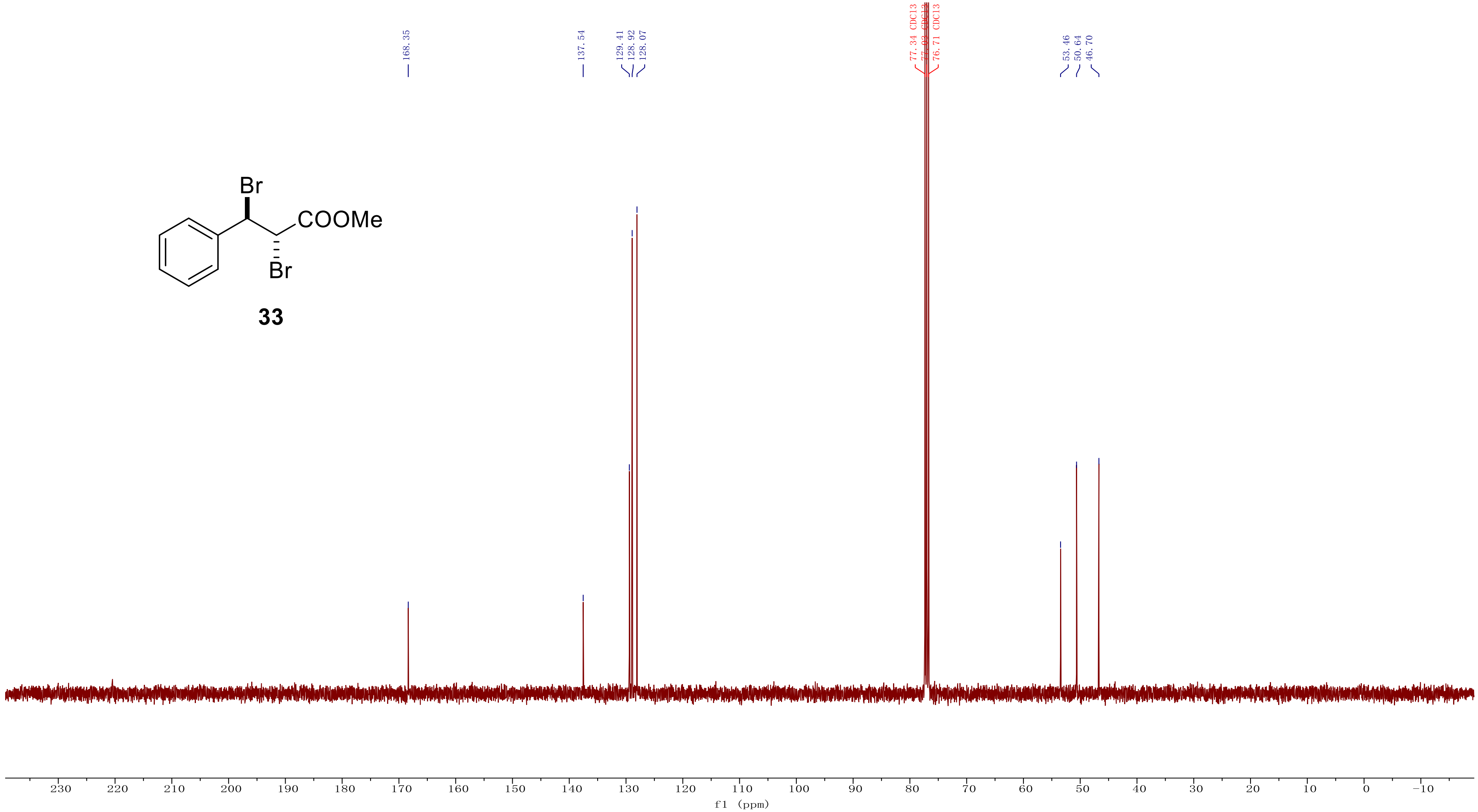

Supplementary Figure 90. ^{13}C NMR spectra of compound **33**.

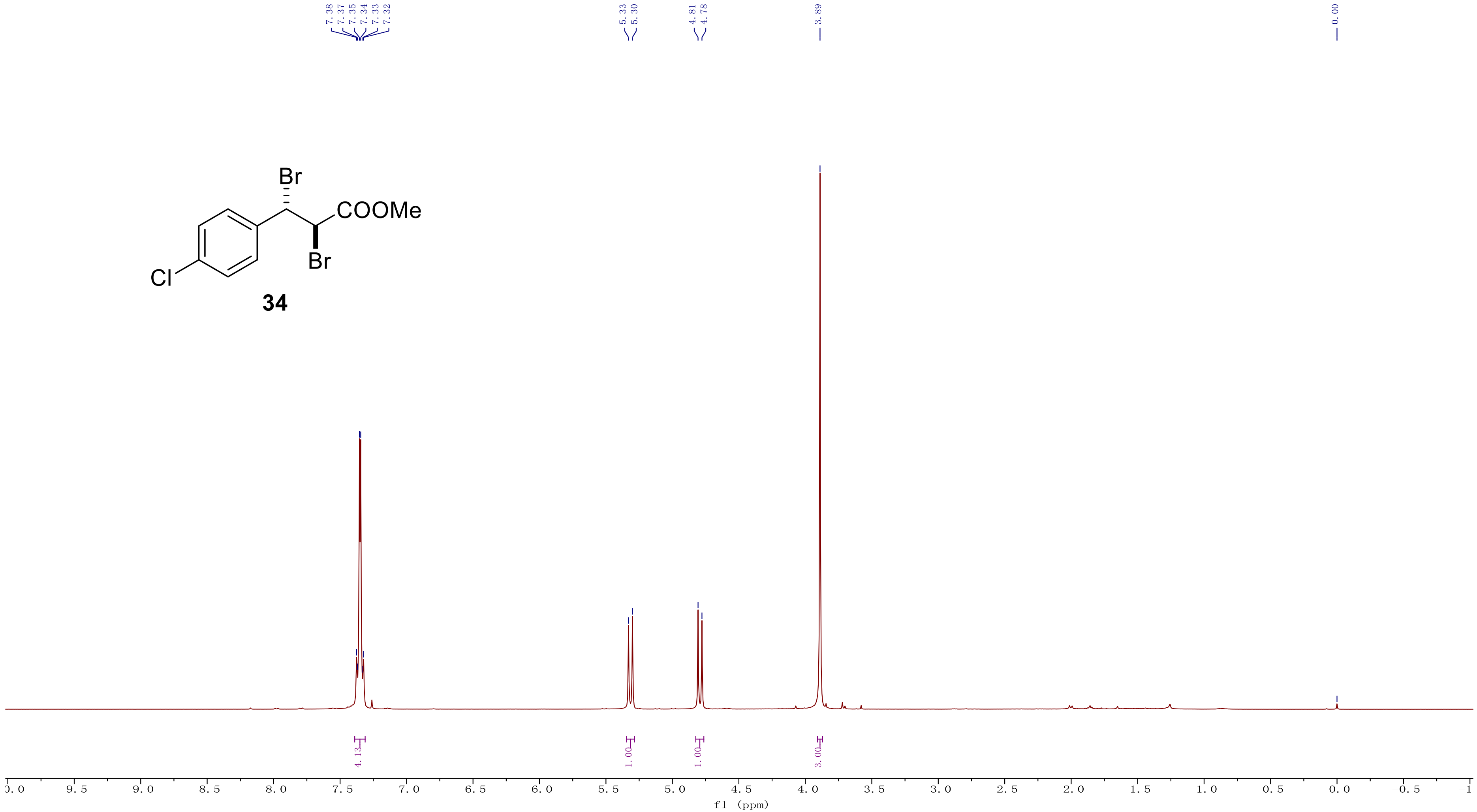

Supplementary Figure 91. ^{1}H NMR spectra of compound **34**.

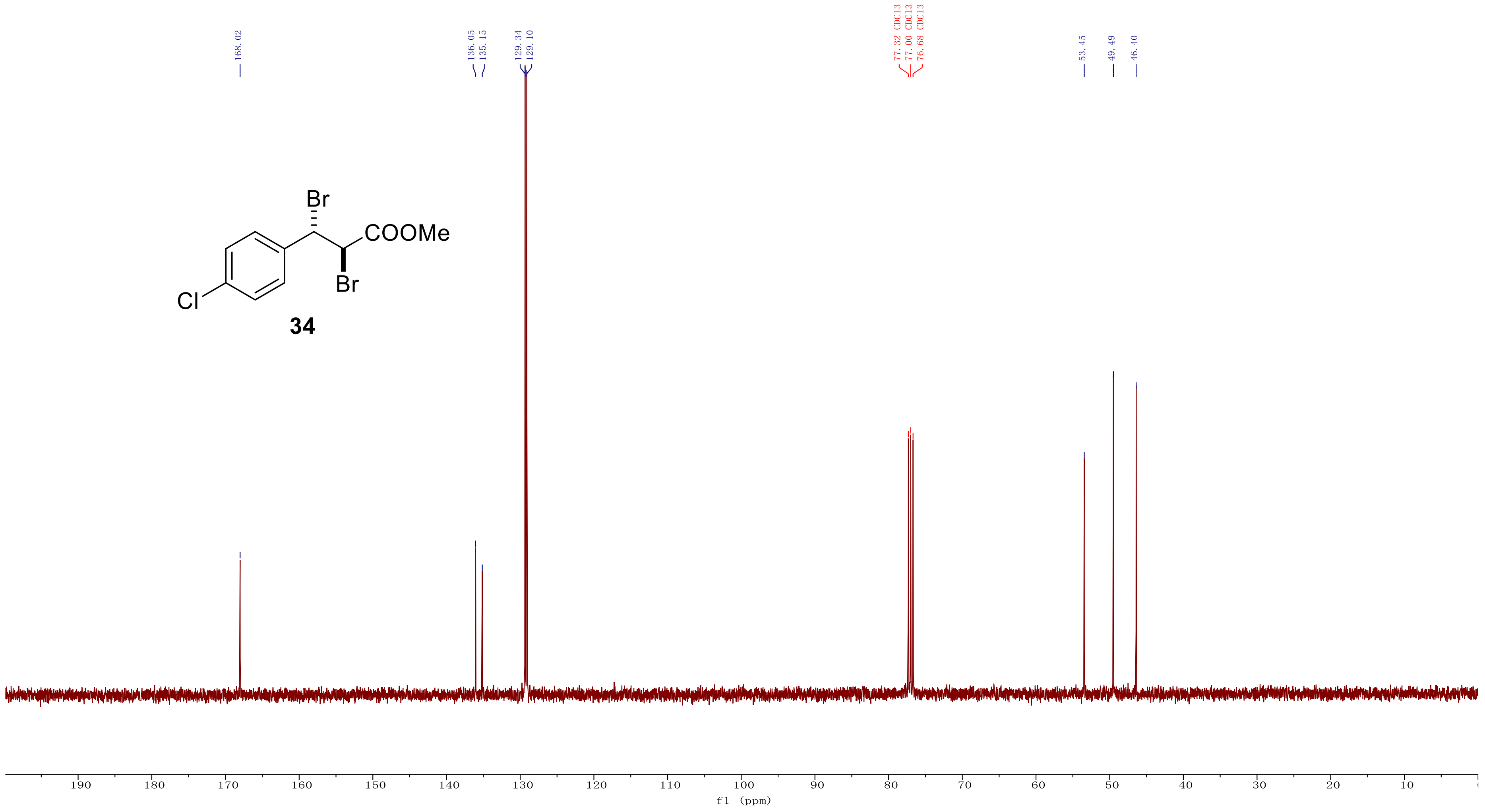

Supplementary Figure 92. ^{13}C NMR spectra of compound **34**.

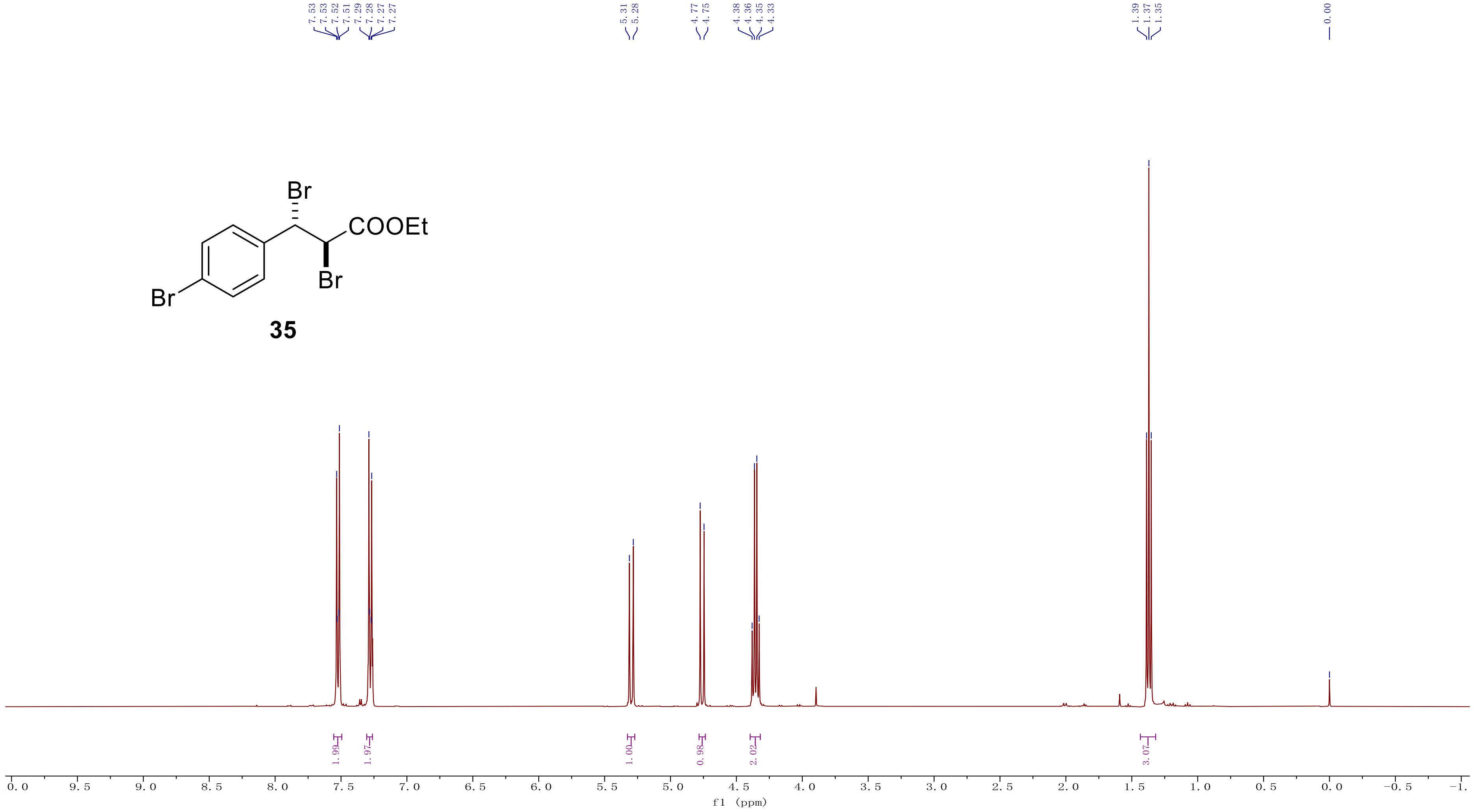

Supplementary Figure 93. ^{1}H NMR spectra of compound **35**.

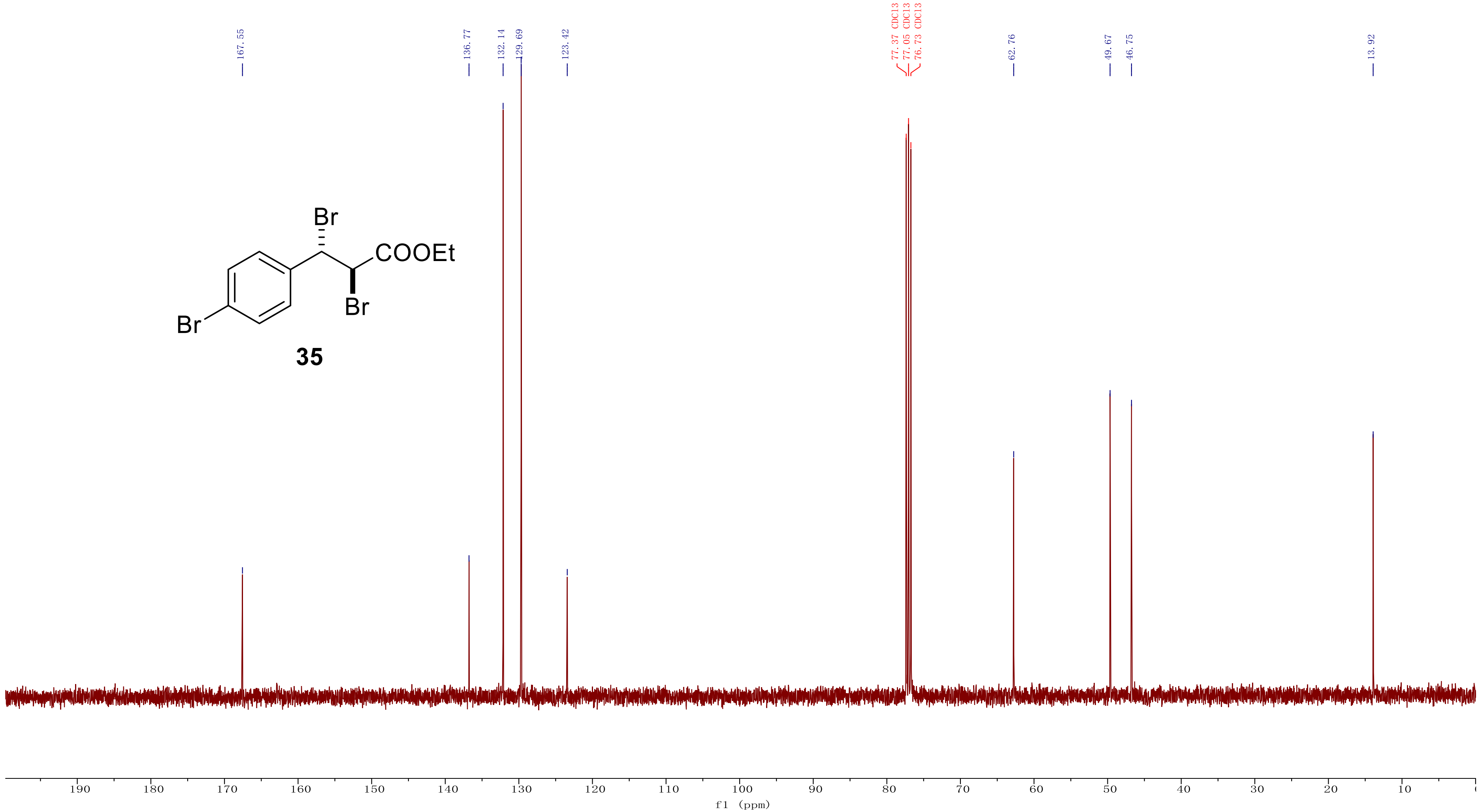

Supplementary Figure 94. ^{13}C NMR spectra of compound **35**.

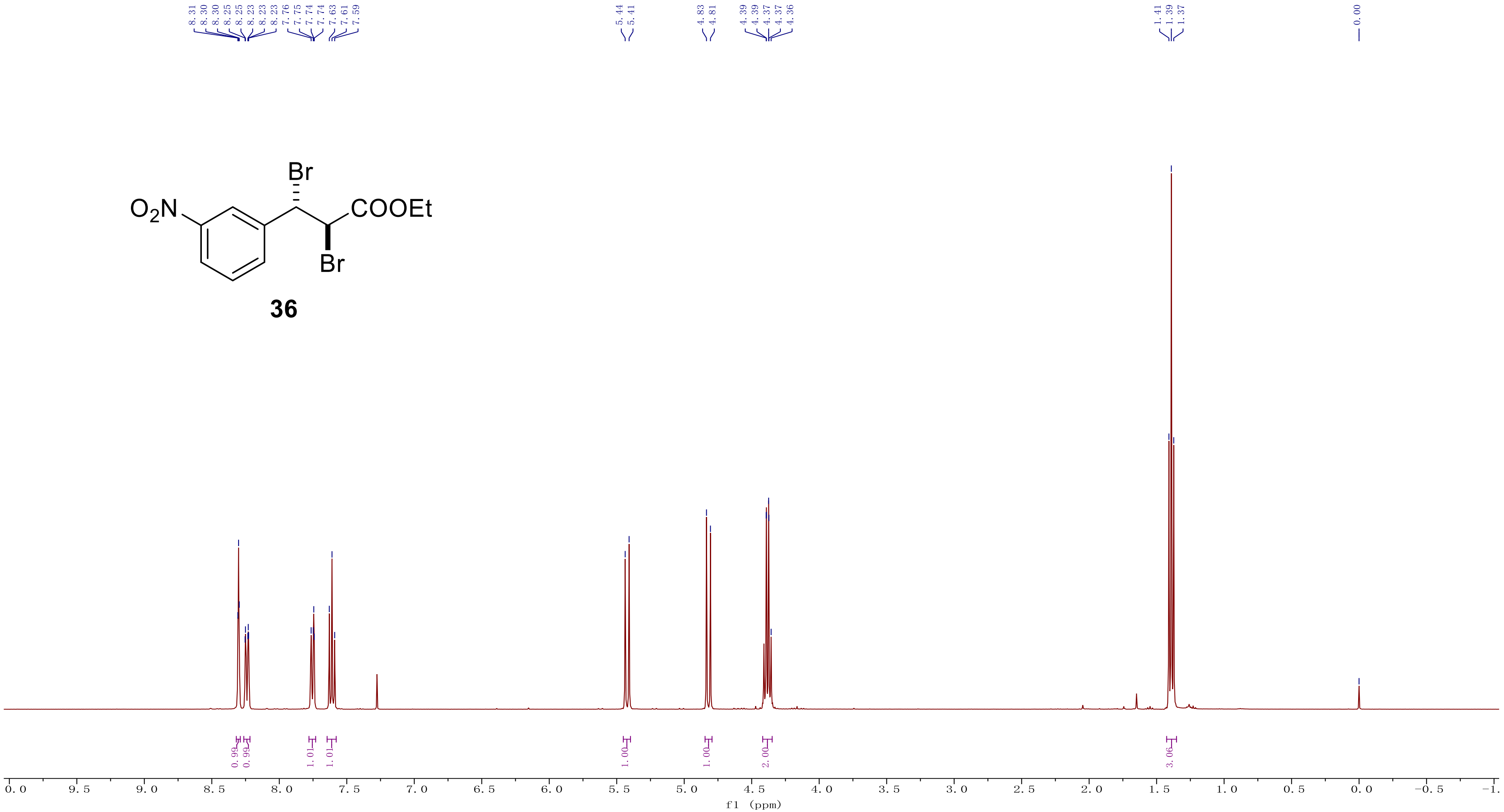

Supplementary Figure 95. ^{1}H NMR spectra of compound **36**.

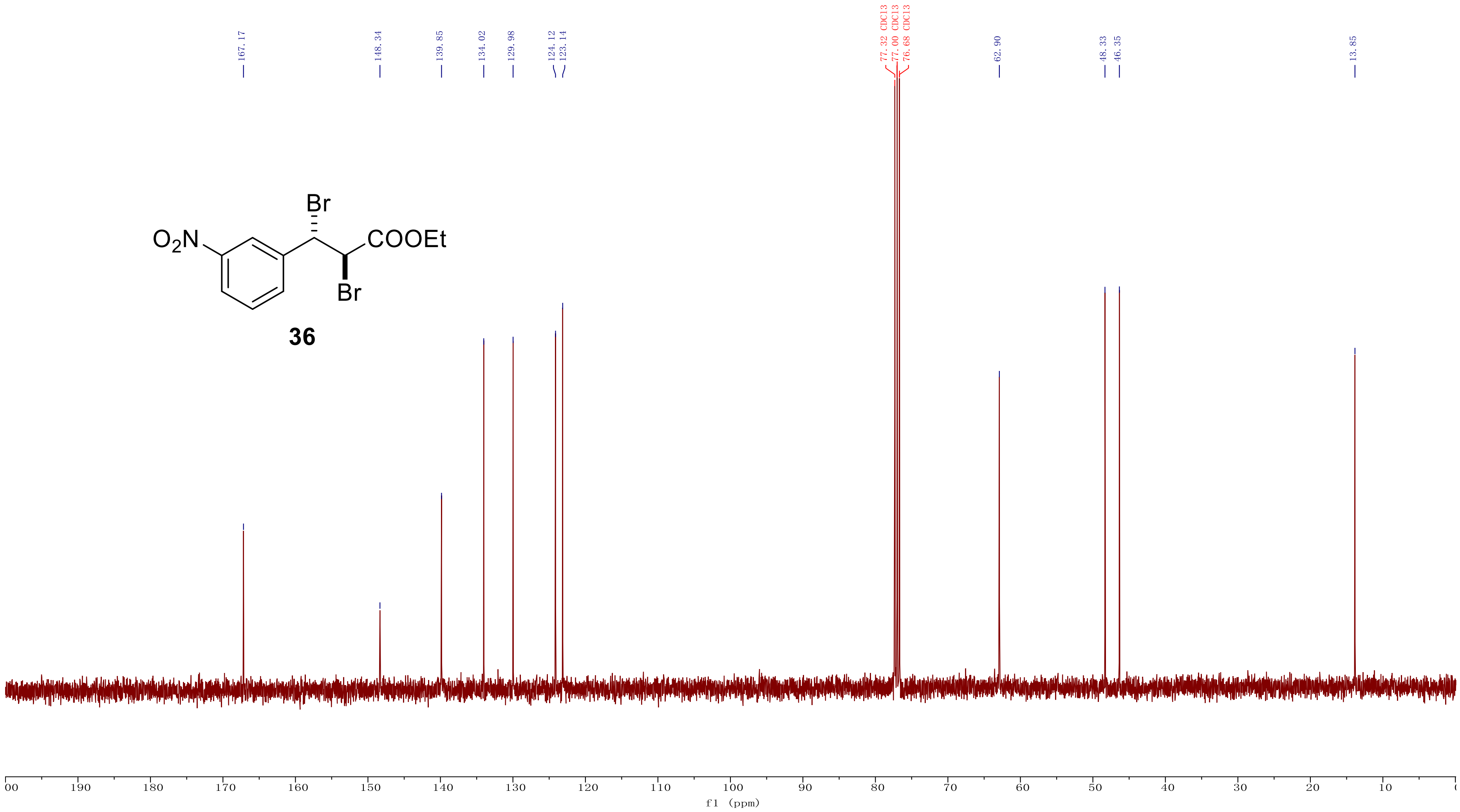

Supplementary Figure 96. ^{13}C NMR spectra of compound **36**.

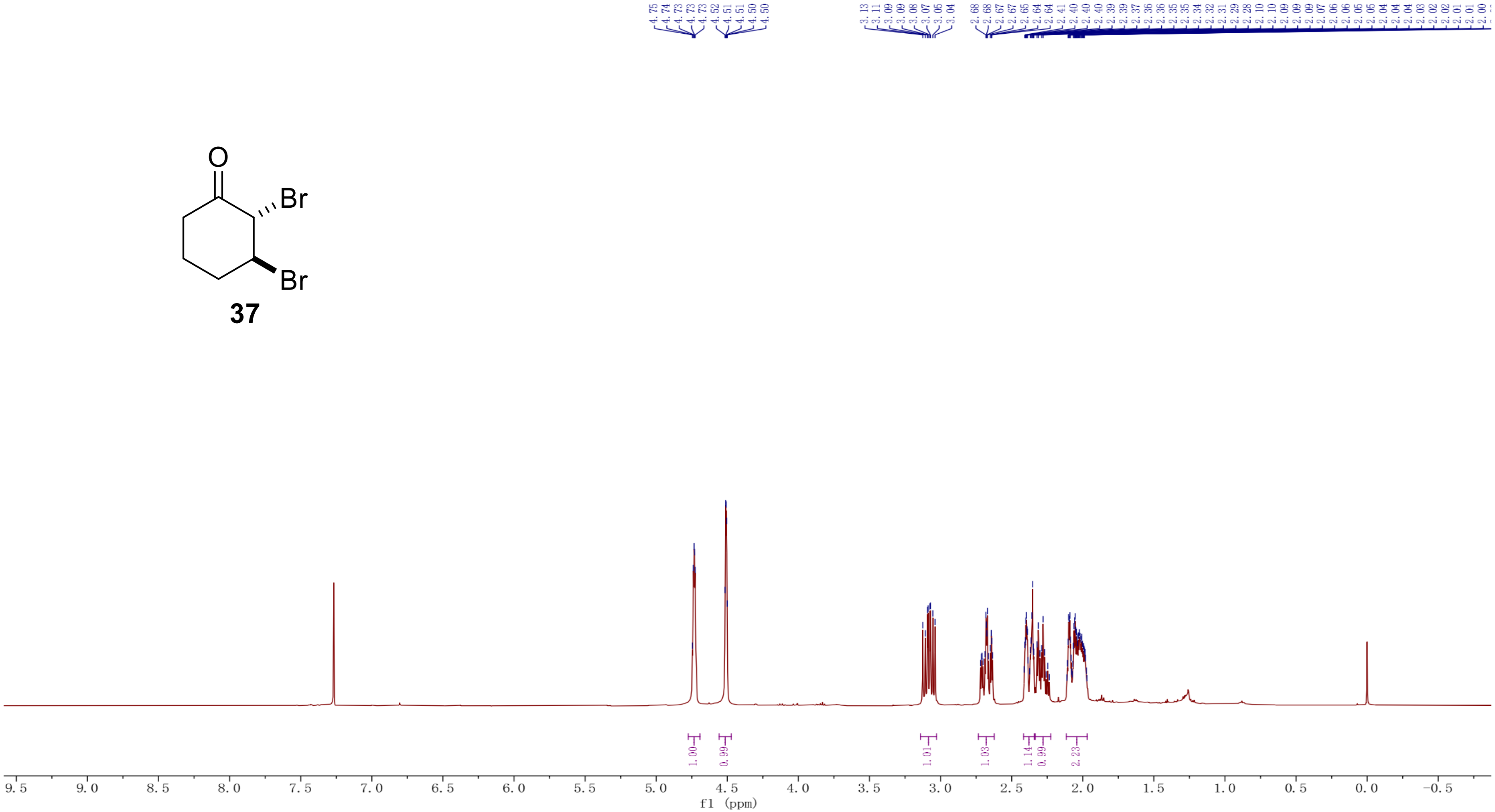

37

Supplementary Figure 97. ^{1}H NMR spectra of compound **37**.

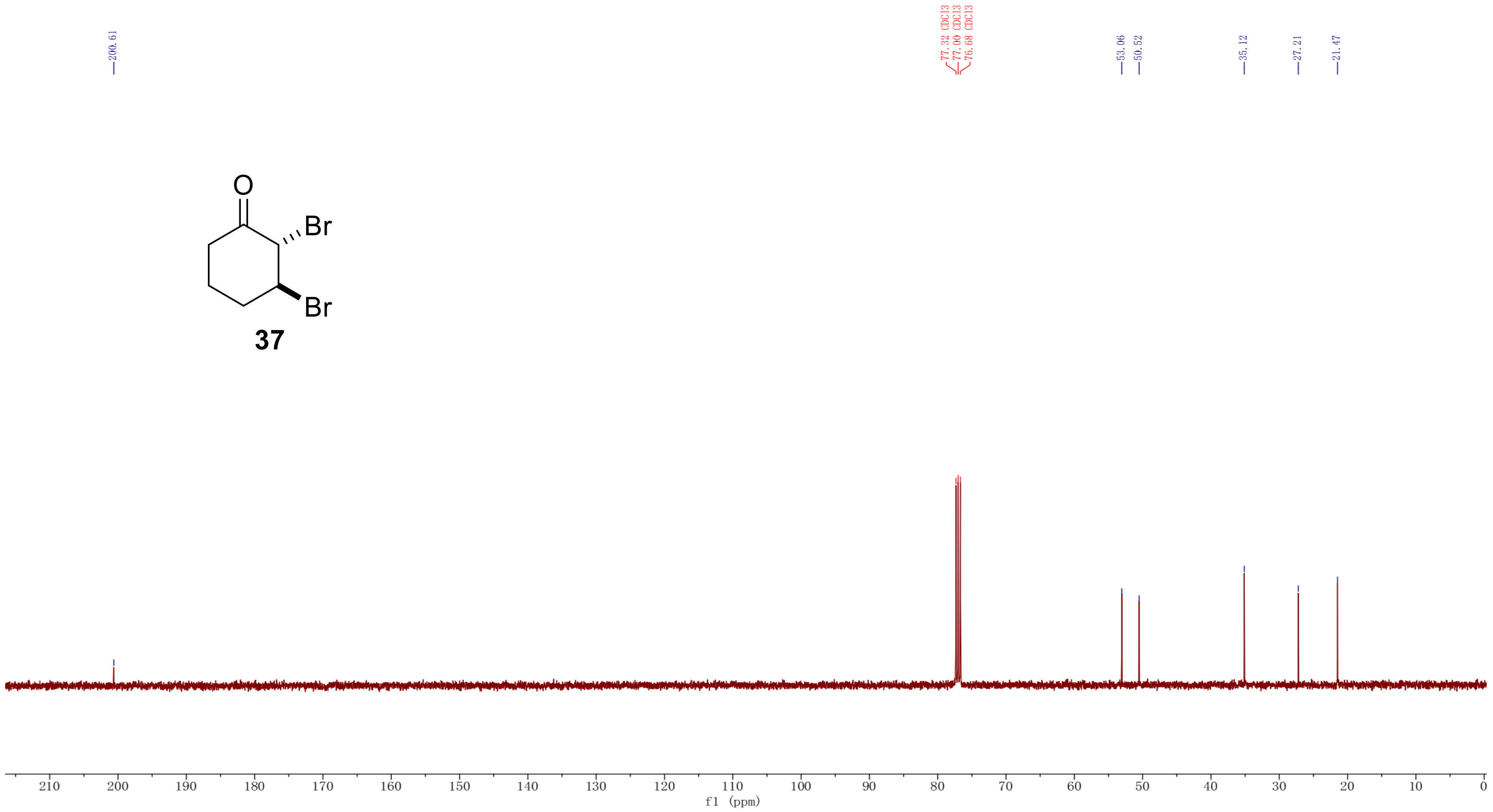

Supplementary Figure 98. ^{13}C NMR spectra of compound **37**.

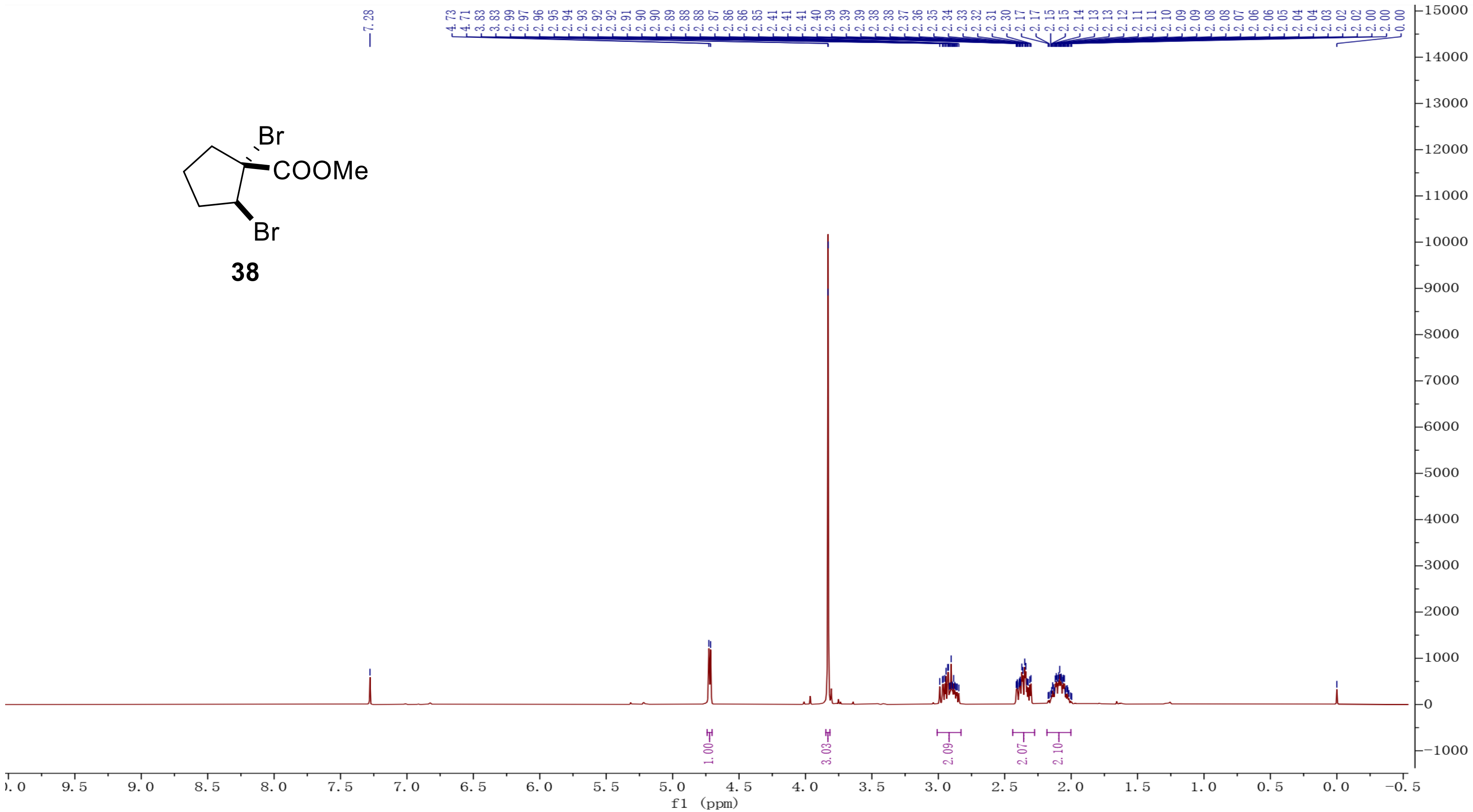

Supplementary Figure 99. ^{1}H NMR spectra of compound **38**.

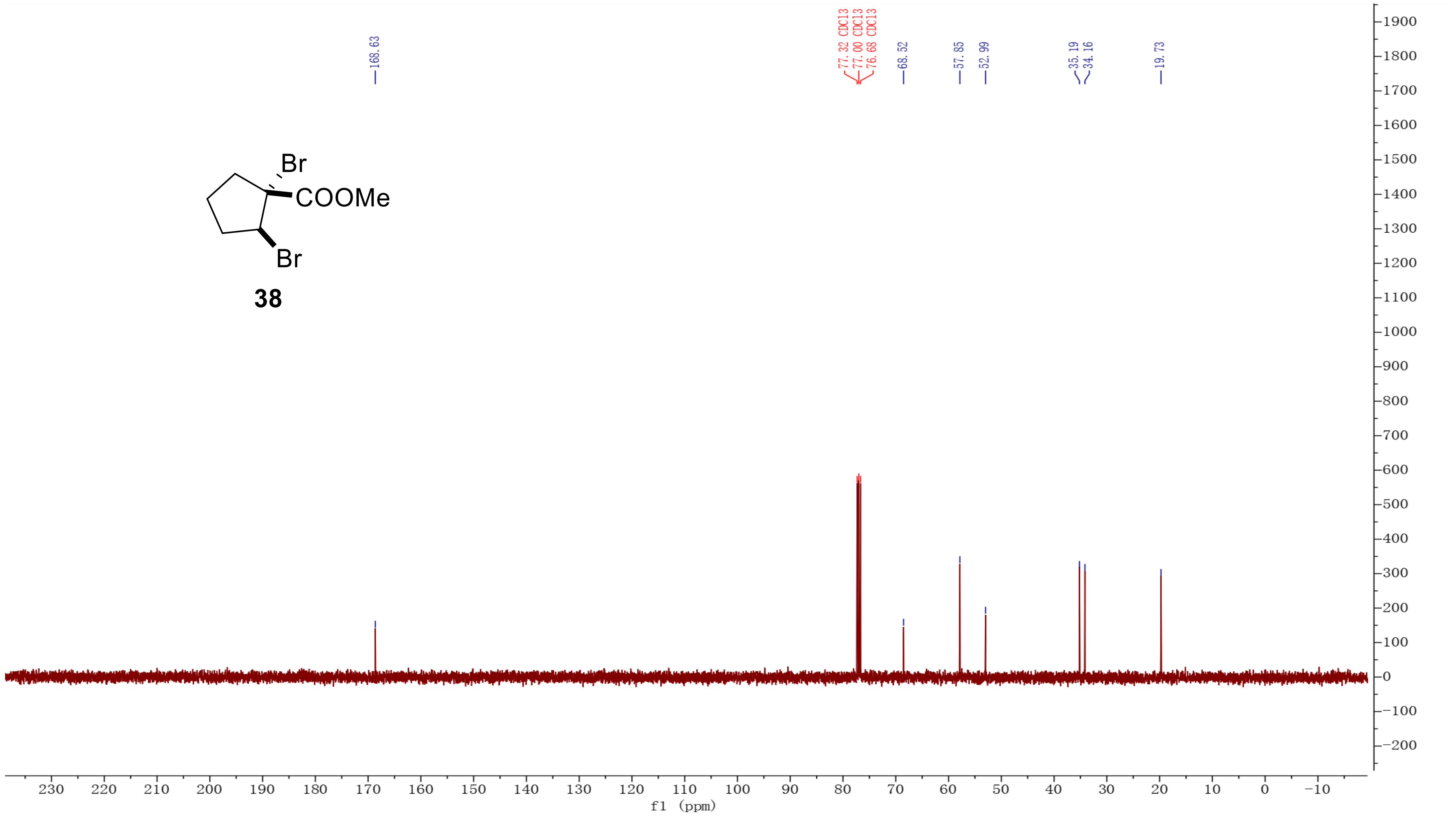

Supplementary Figure 100. ^{13}C NMR spectra of compound **38**.

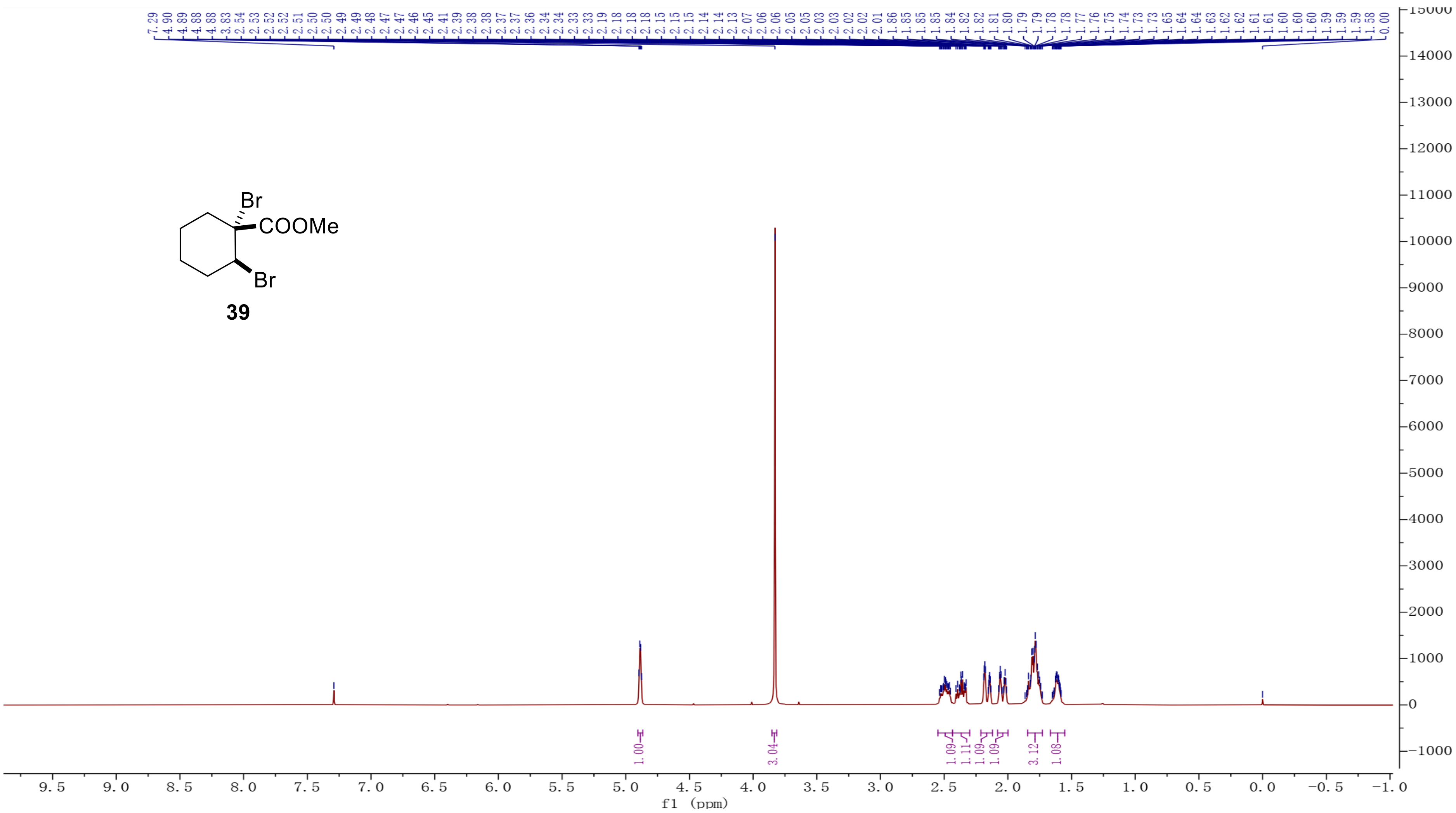

Supplementary Figure 101. ^{1}H NMR spectra of compound **39**.

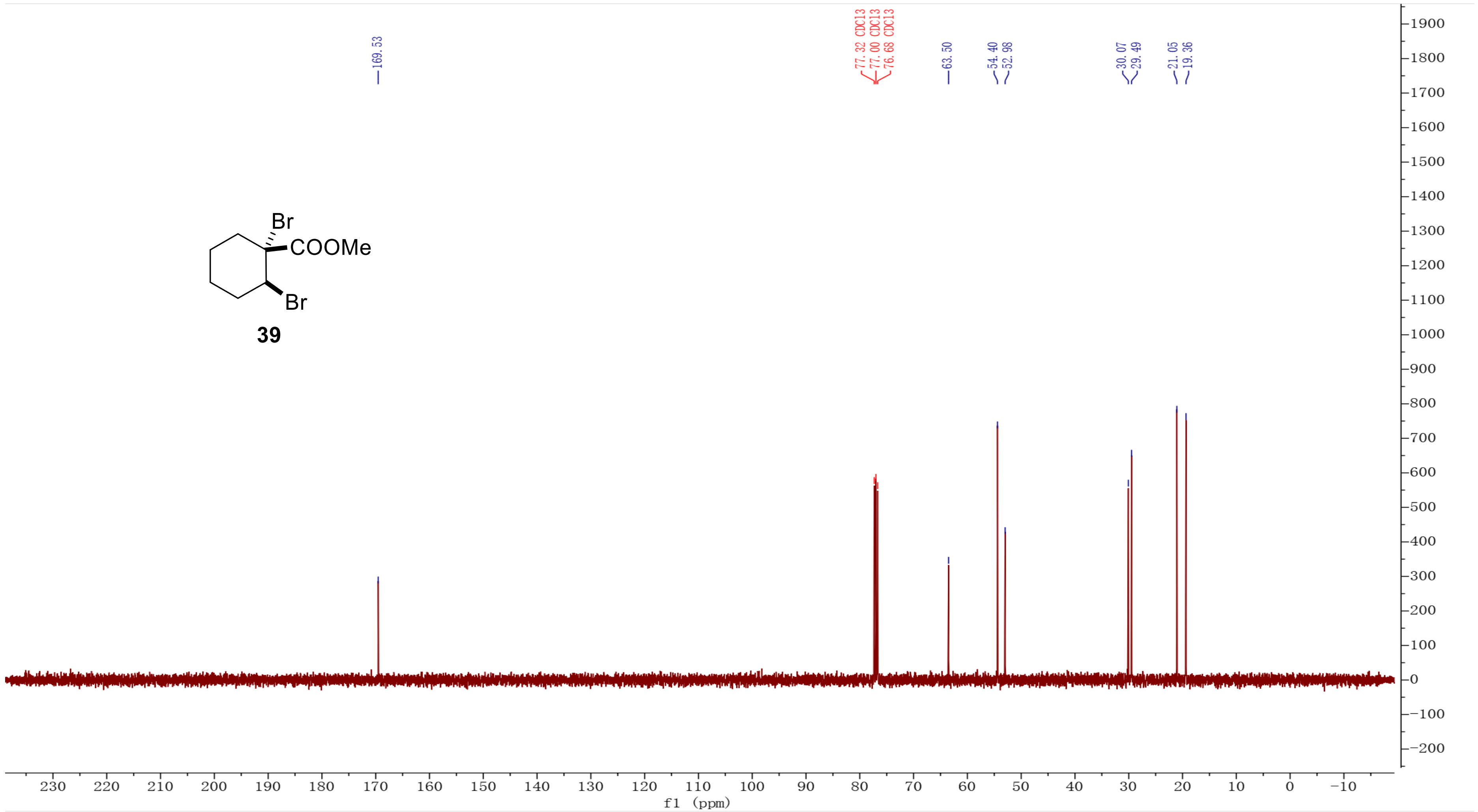

Supplementary Figure 102. ^{13}C NMR spectra of compound **39**.

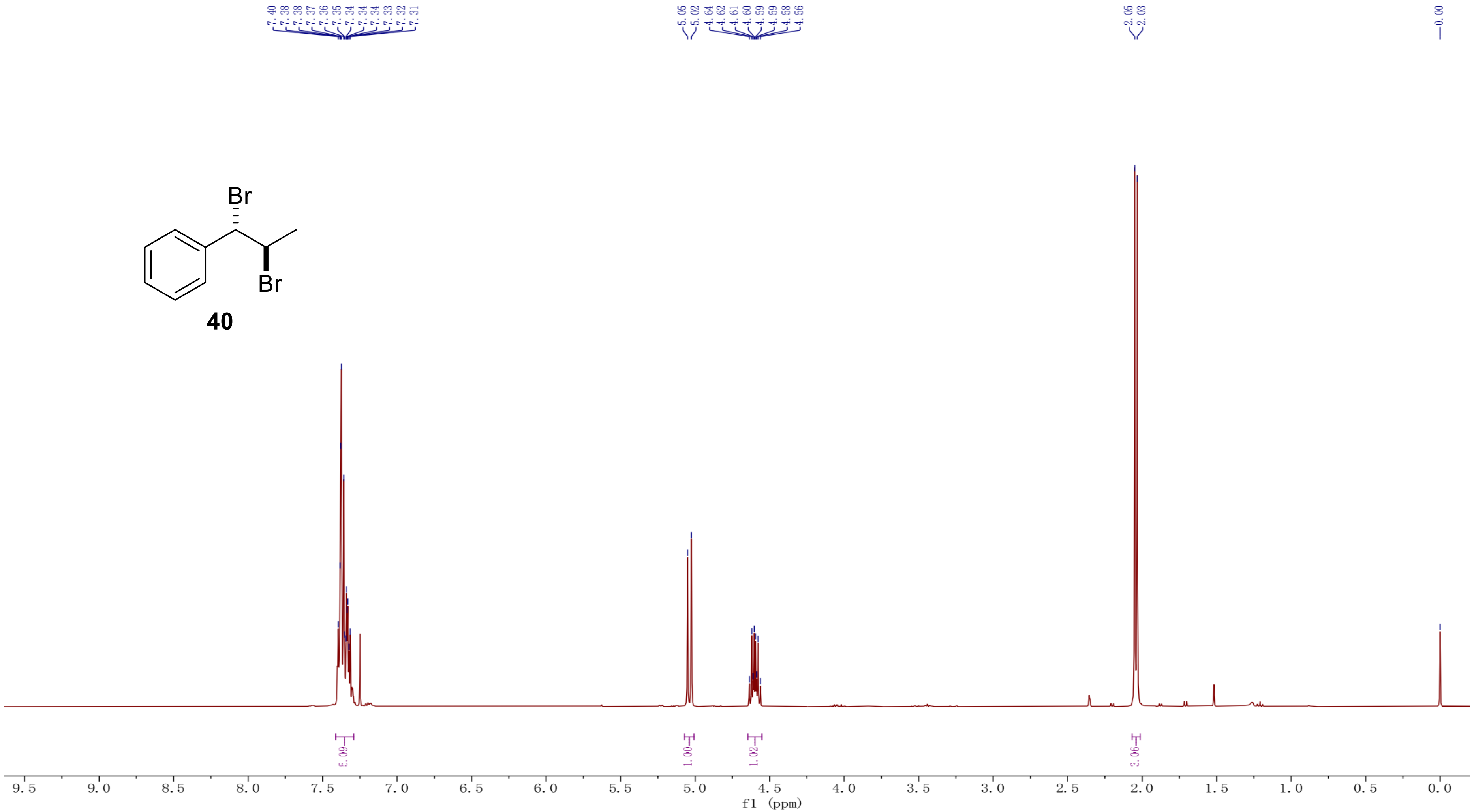

Supplementary Figure 103. ^{1}H NMR spectra of compound **40**.

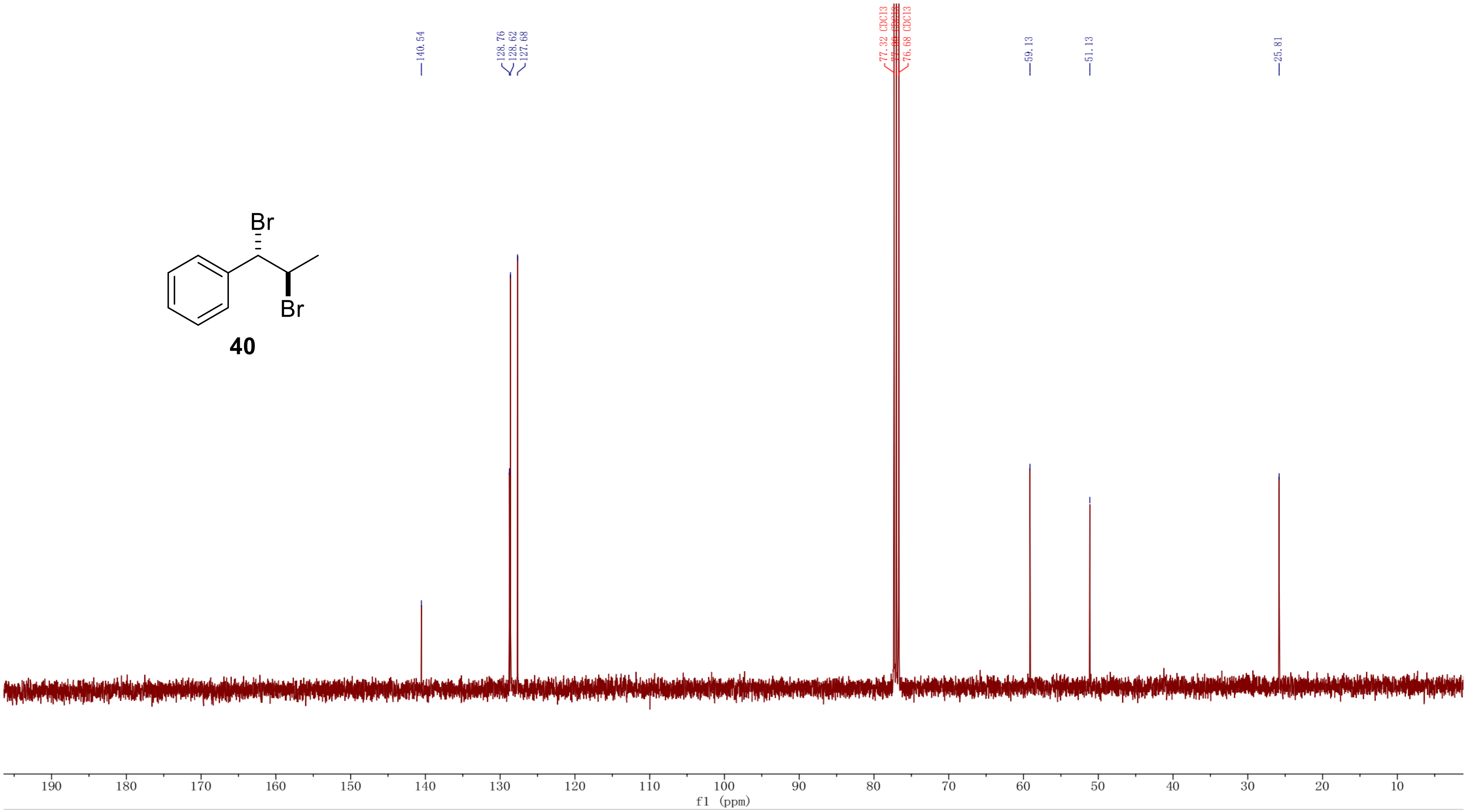

Supplementary Figure 104. ^{13}C NMR spectra of compound **40**.

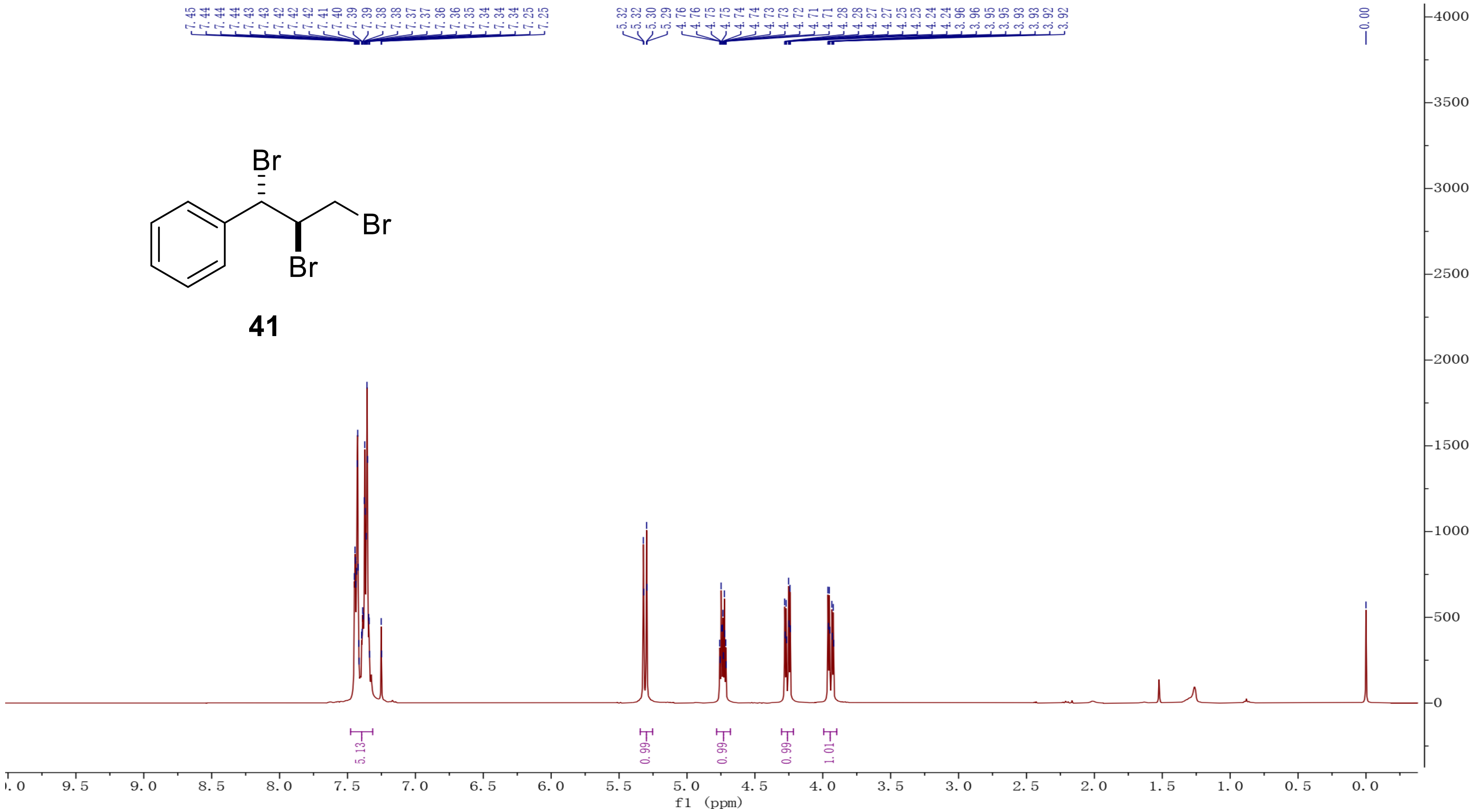

Supplementary Figure 105. ^{1}H NMR spectra of compound **41**.

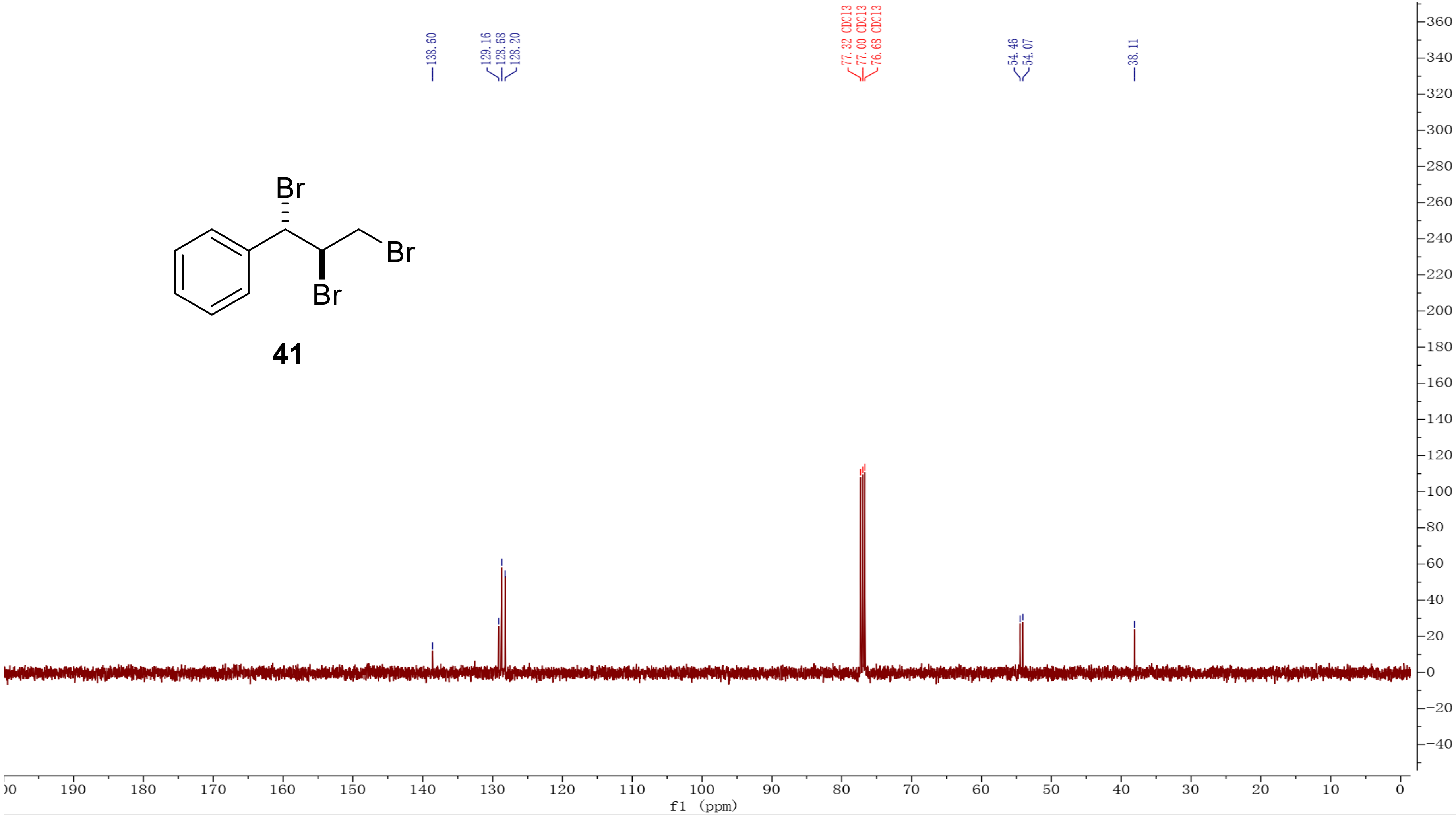

Supplementary Figure 106. ^{13}C NMR spectra of compound **41**.

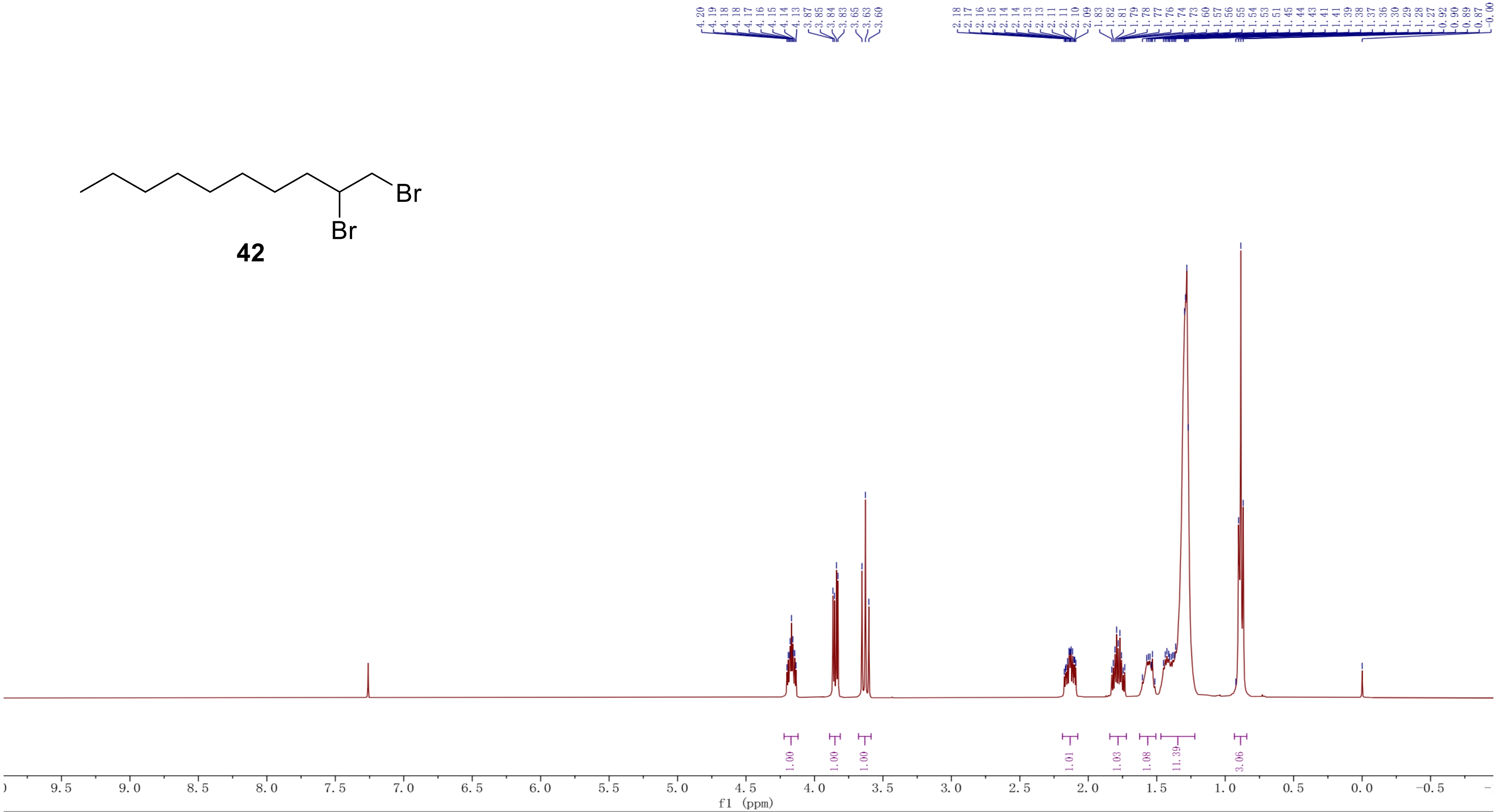

42

Supplementary Figure 107. ^{1}H NMR spectra of compound **42**.

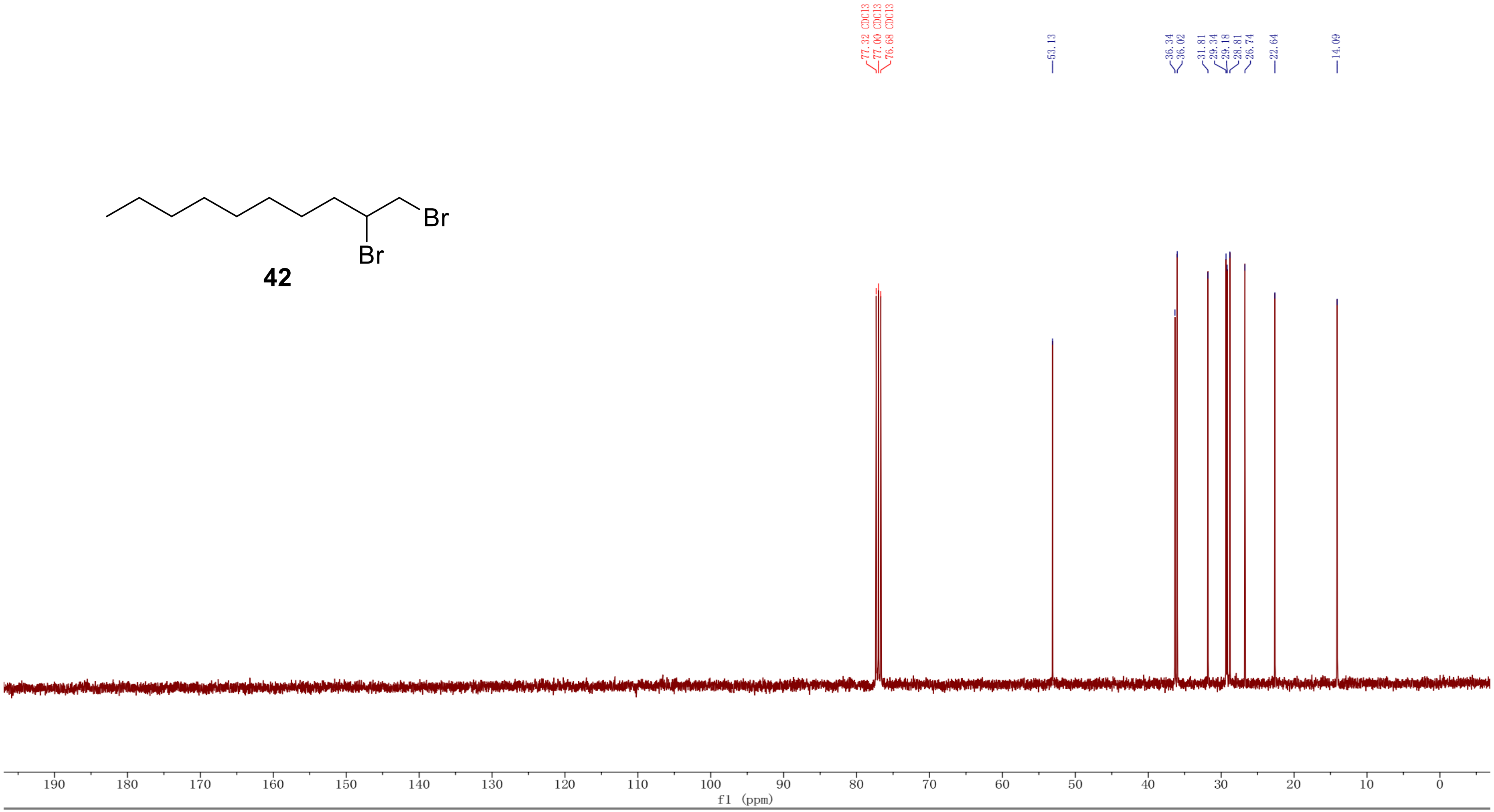

Supplementary Figure 108. ^{13}C NMR spectra of compound **42**.

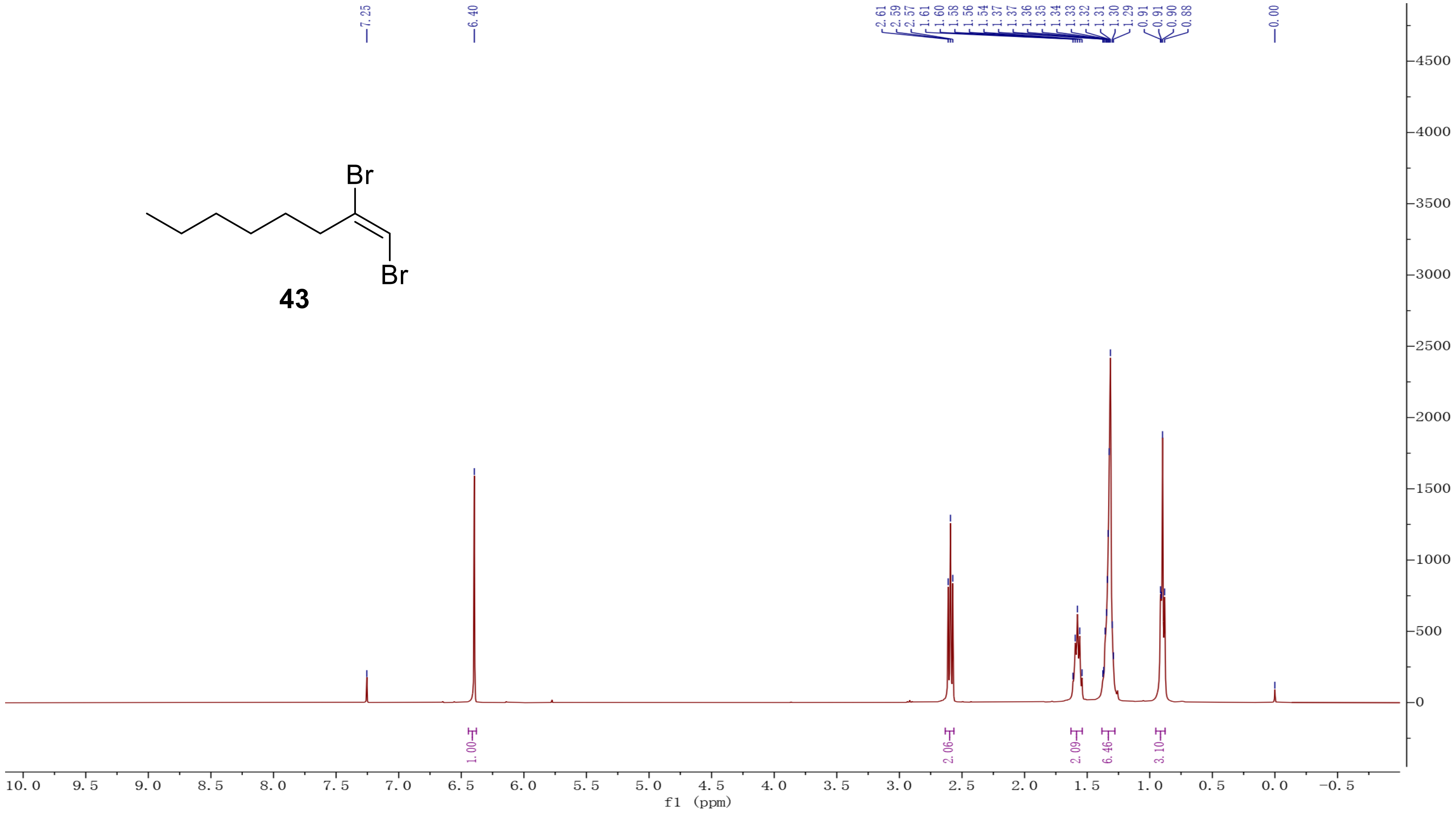

Supplementary Figure 109. ^{1}H NMR spectra of compound **43**.

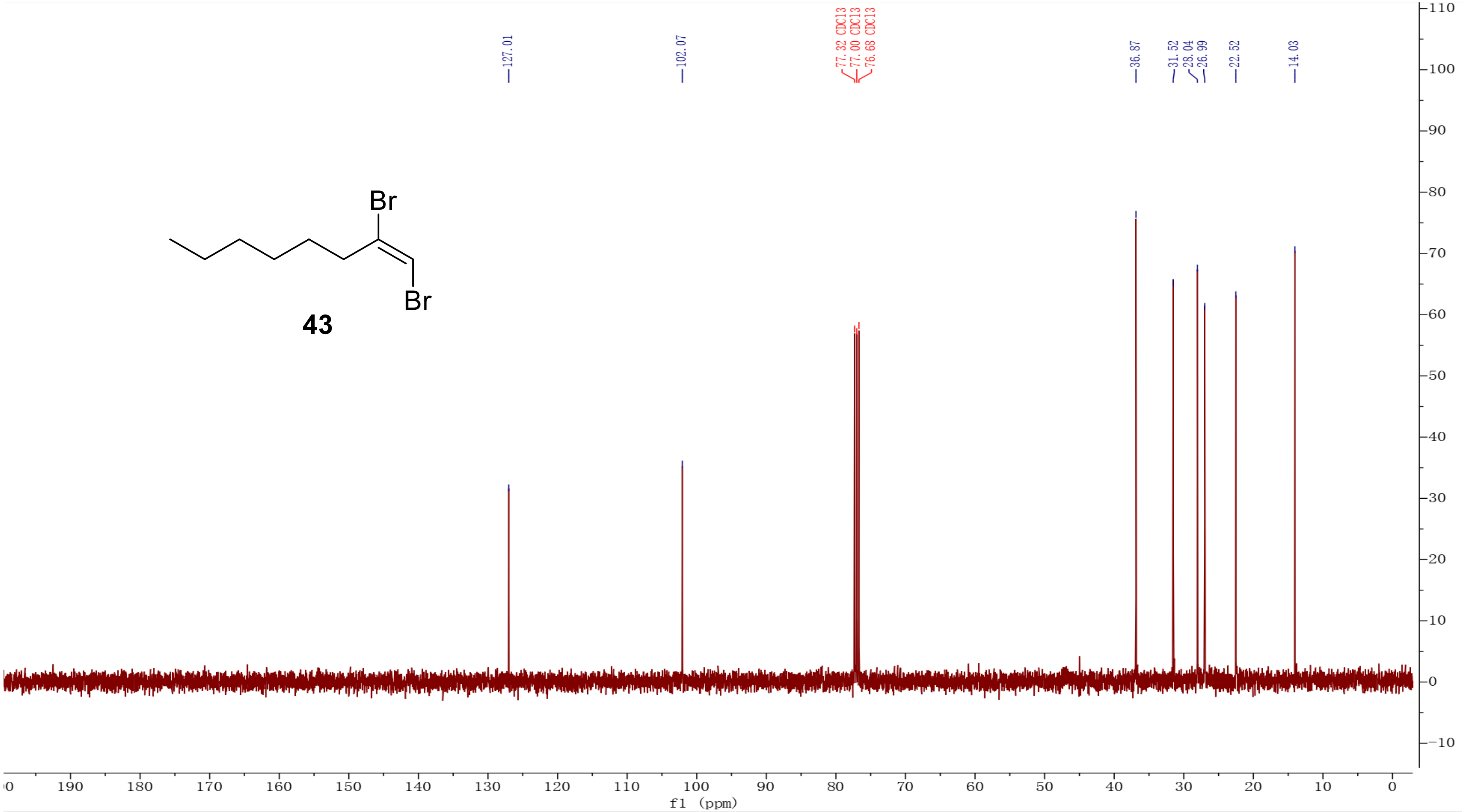

Supplementary Figure 110. ^{13}C NMR spectra of compound **43**.

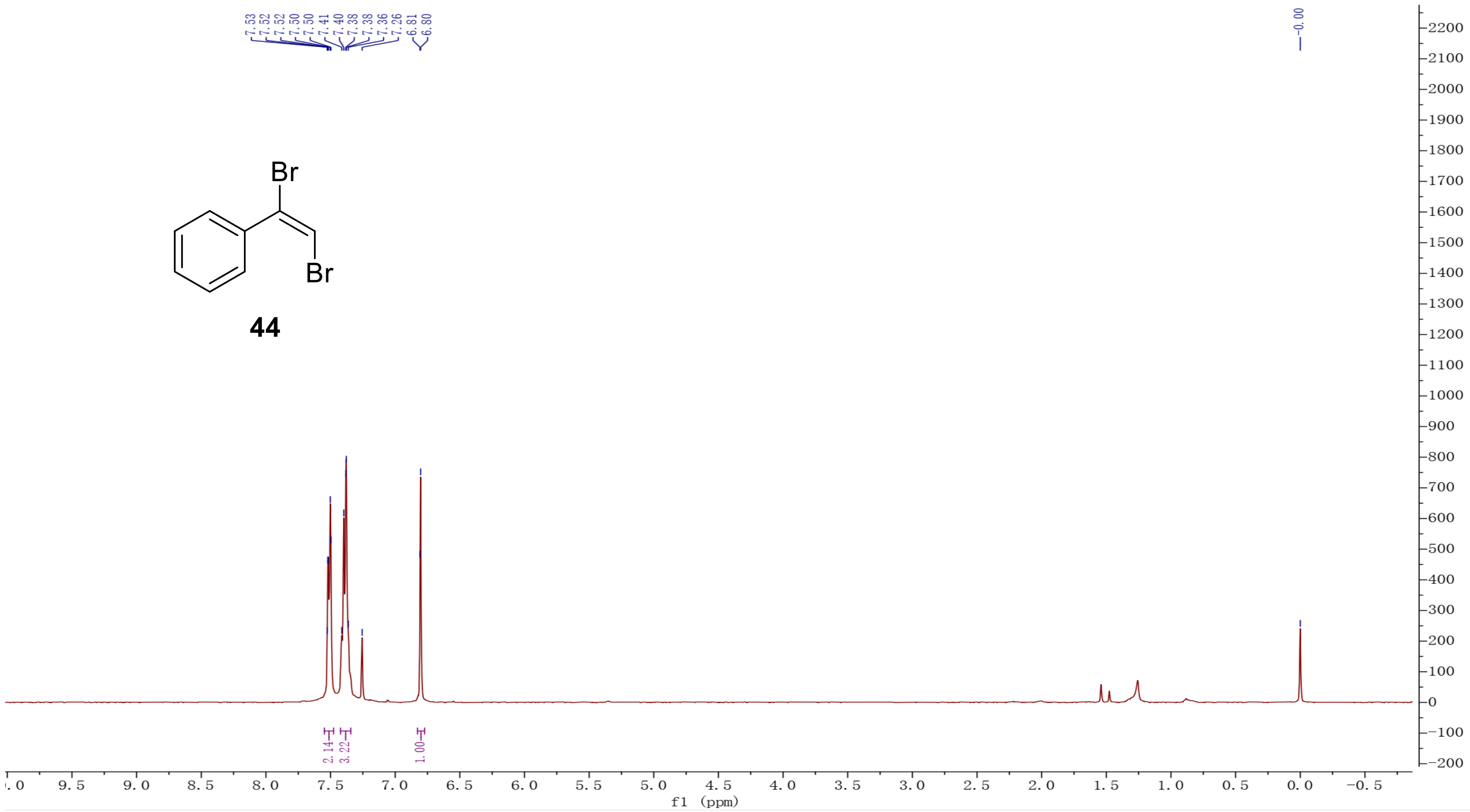

Supplementary Figure 111. ^{1}H NMR spectra of compound **44**.

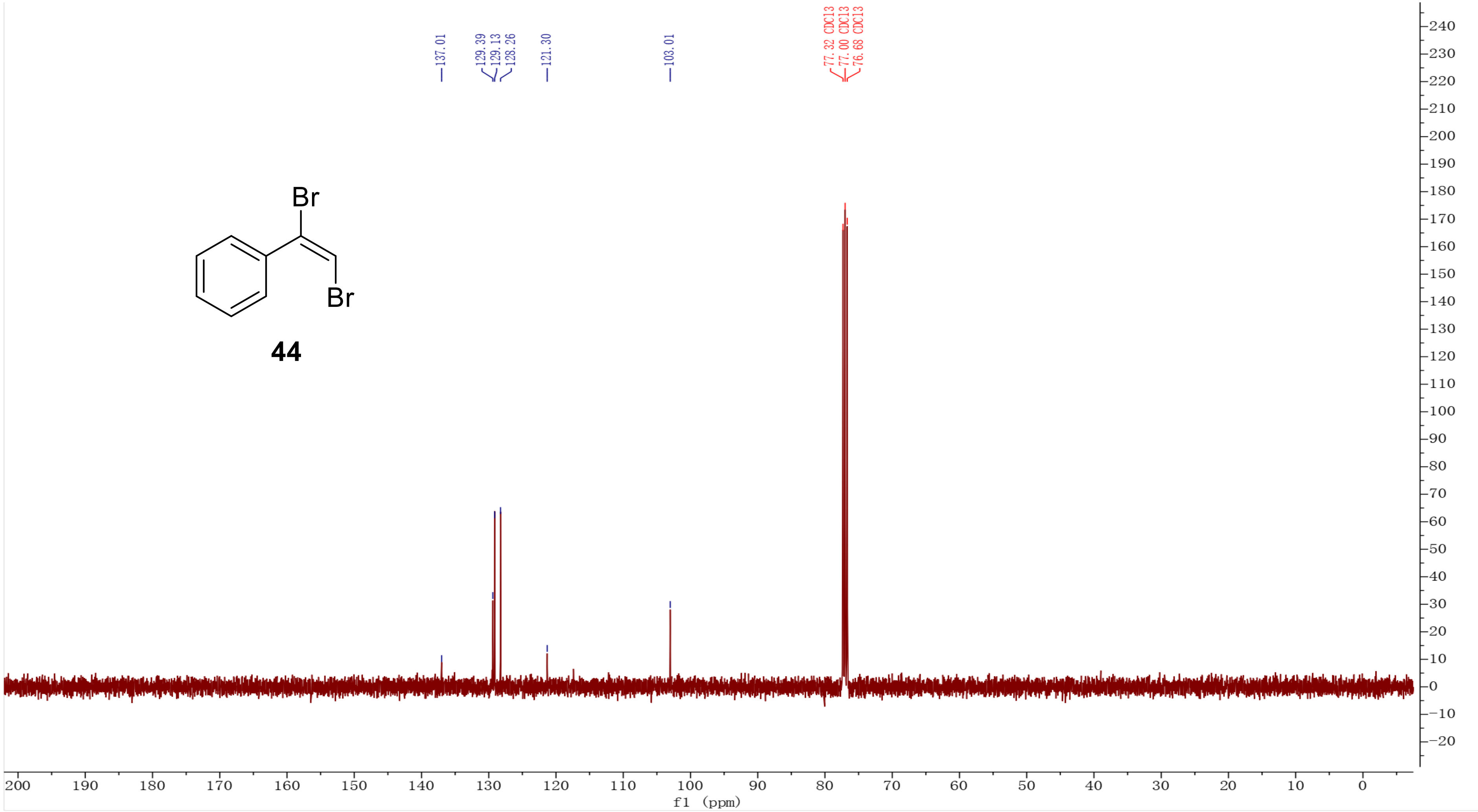

Supplementary Figure 112. ^{13}C NMR spectra of compound **44**.

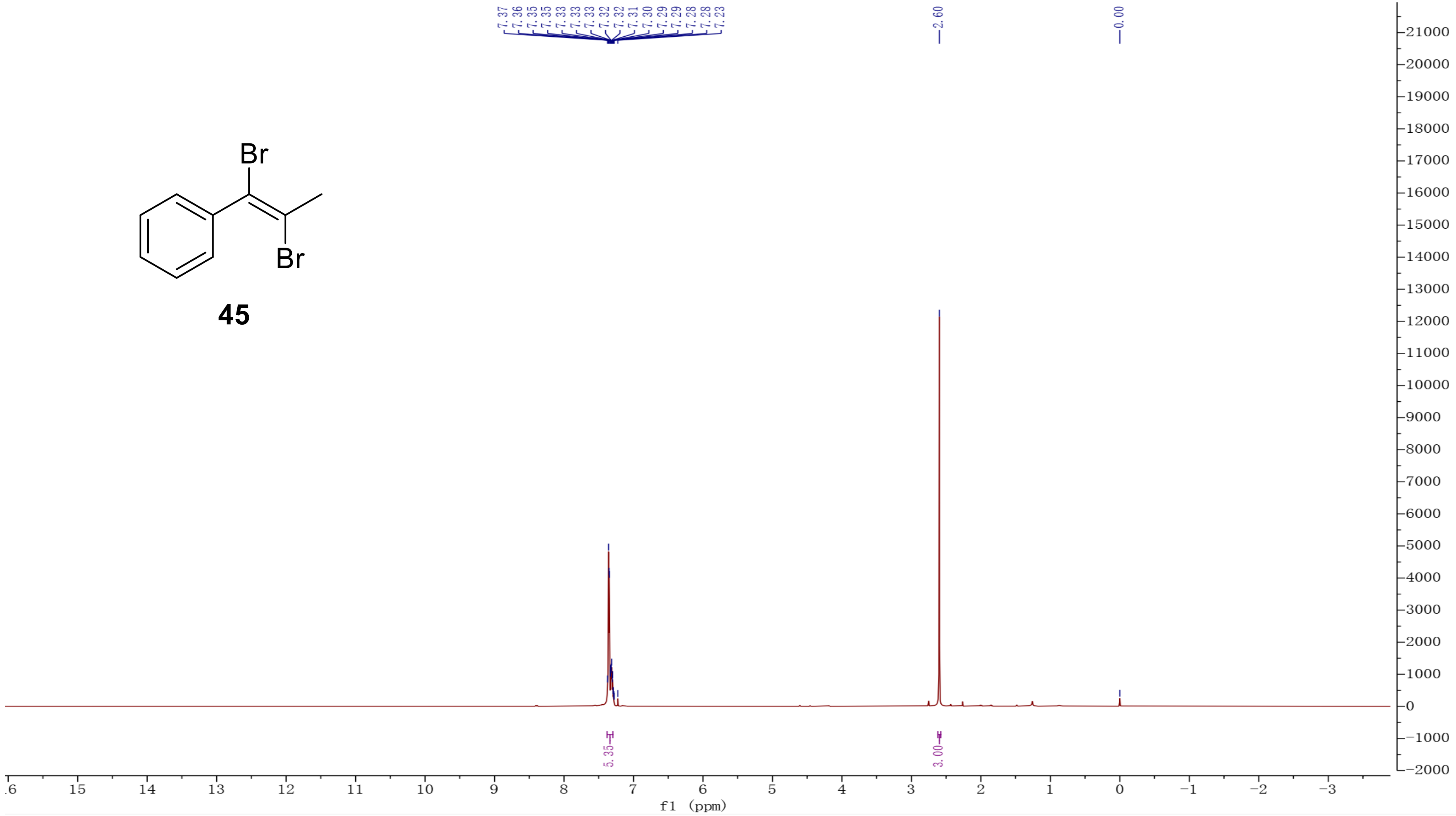

Supplementary Figure 113. ^{1}H NMR spectra of compound **44**.

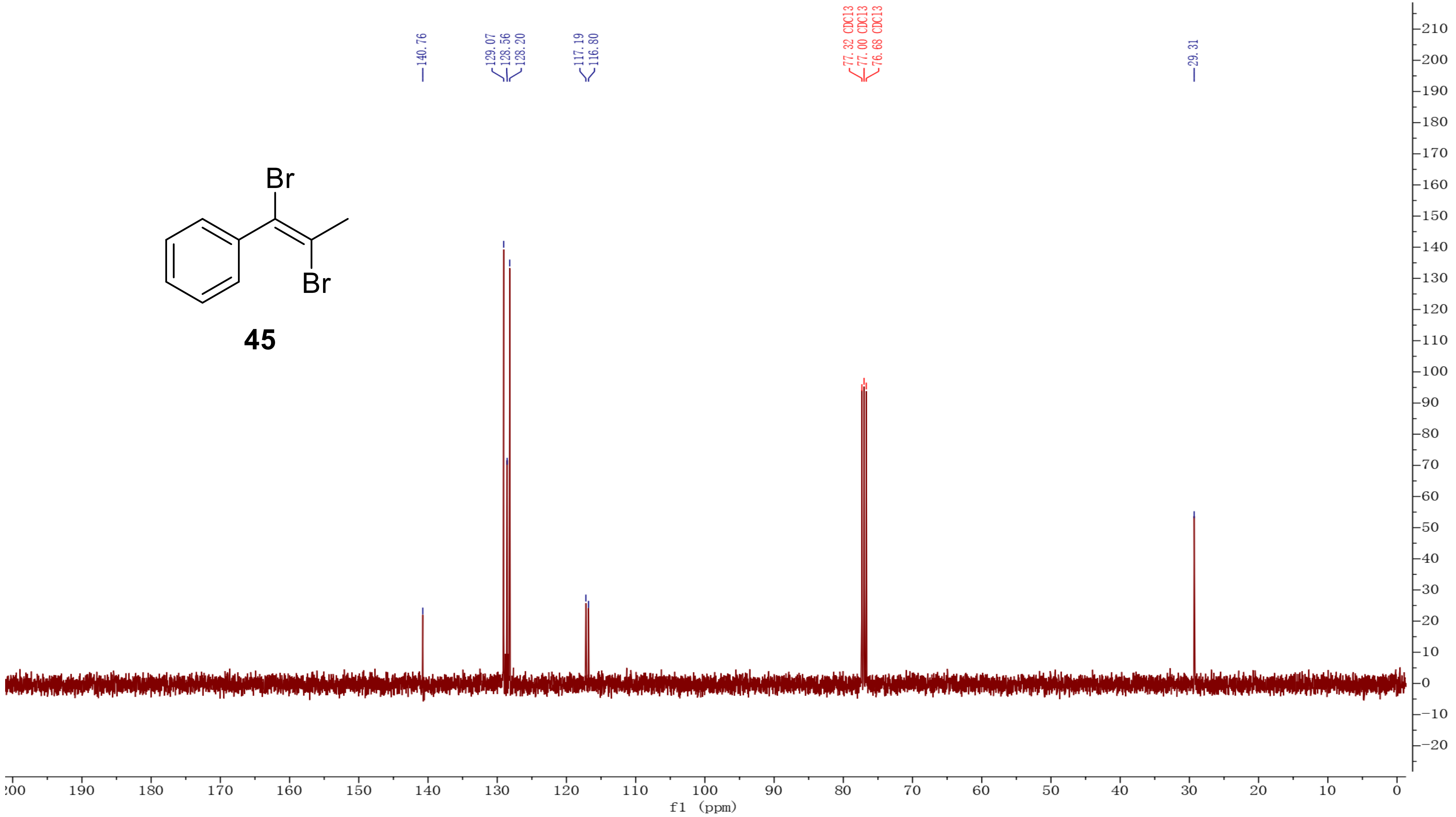

Supplementary Figure 114. ^{13}C NMR spectra of compound **45**.

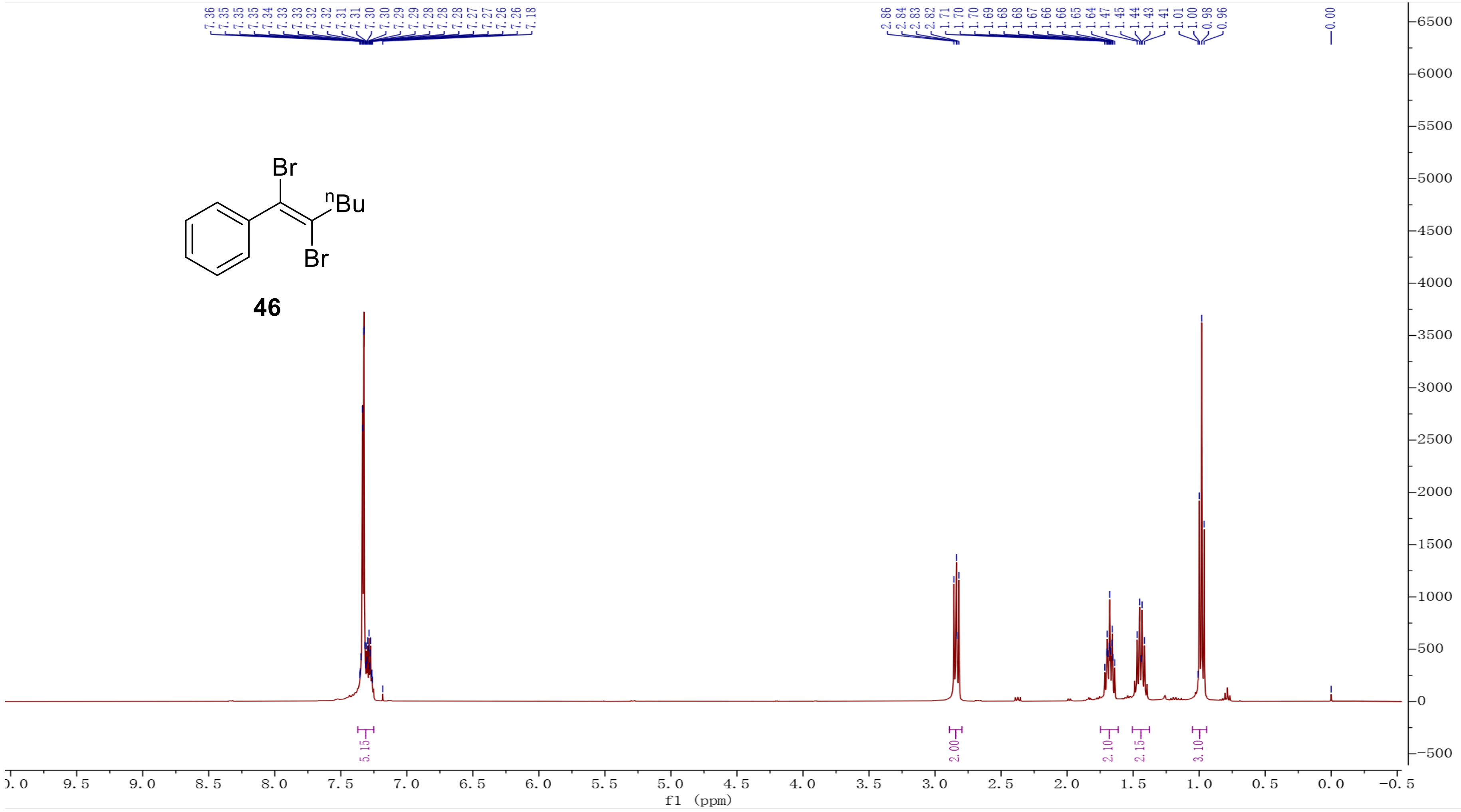

Supplementary Figure 115. ^{1}H NMR spectra of compound **46**.

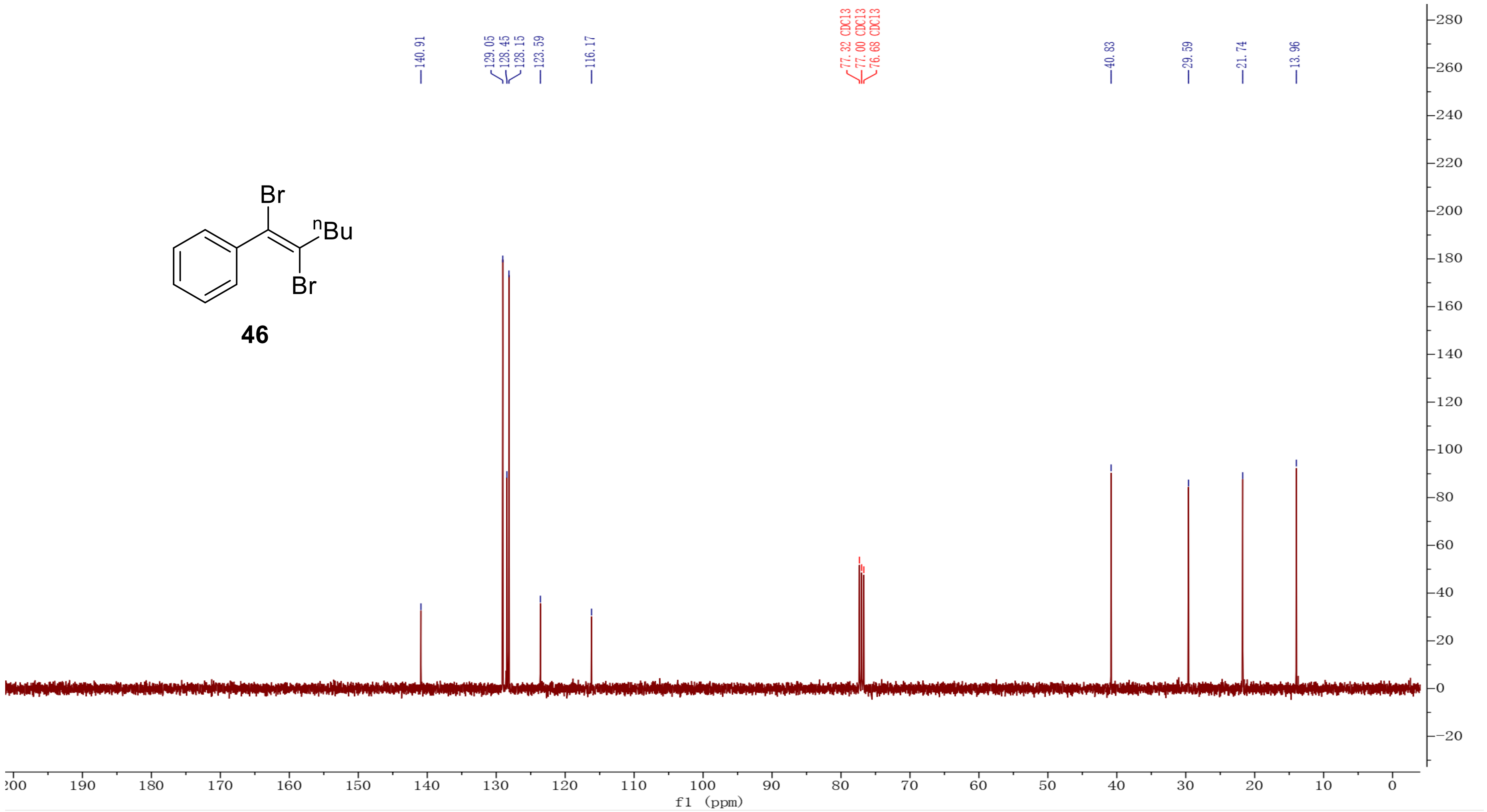

Supplementary Figure 116. ^{13}C NMR spectra of compound **46**.

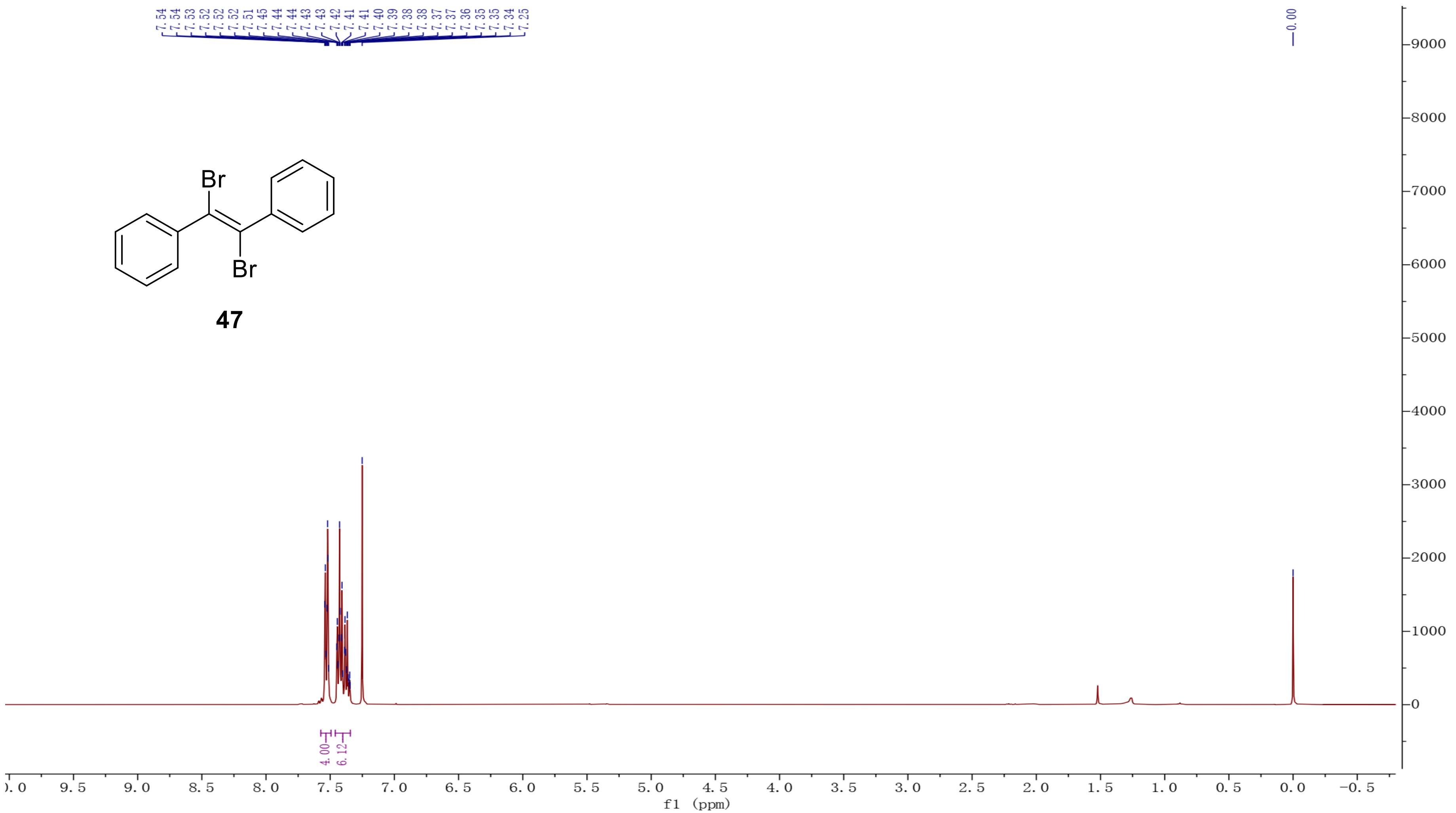

Supplementary Figure 117. ^{1}H NMR spectra of compound **47**.

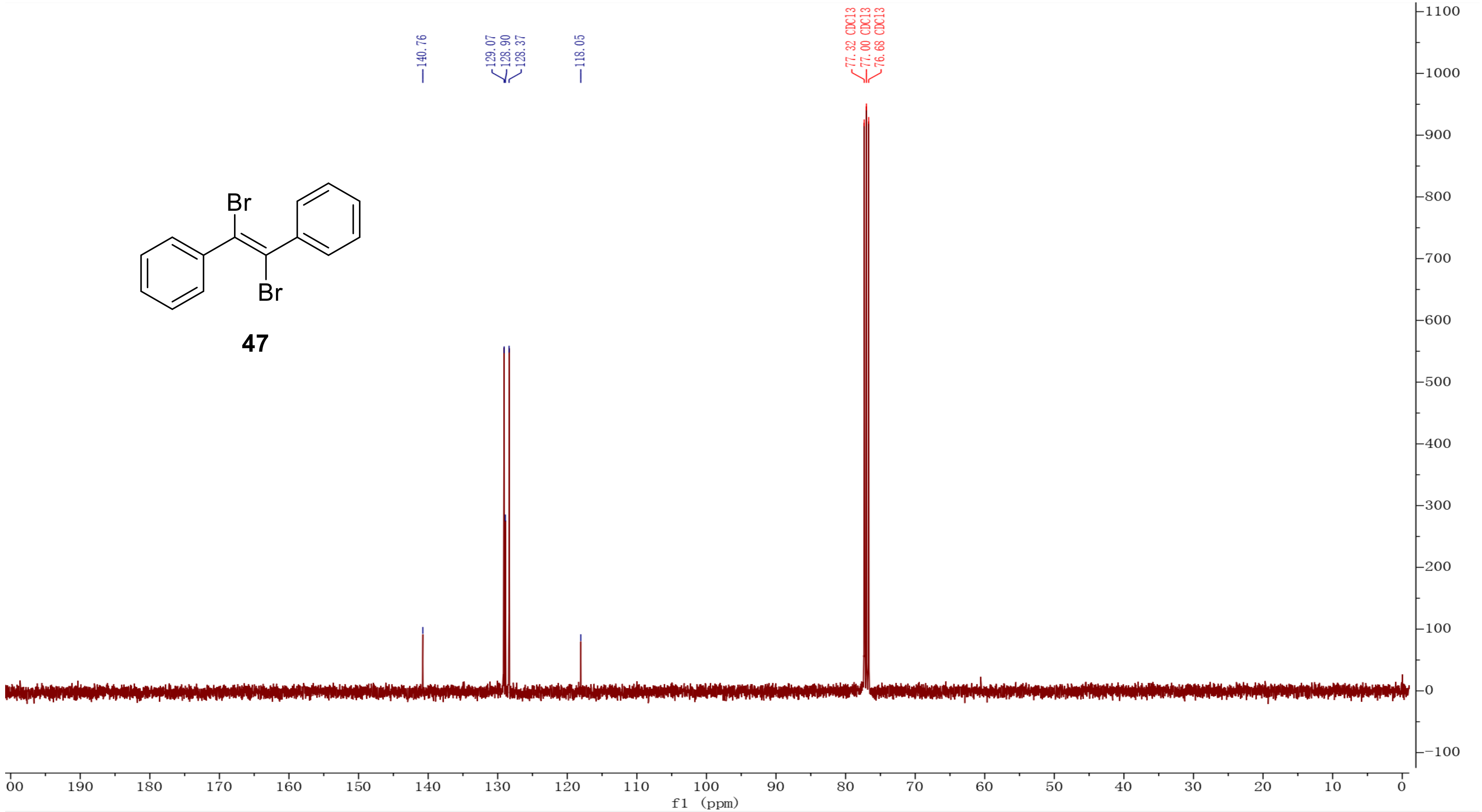

Supplementary Figure 118. ^{13}C NMR spectra of compound **47**.

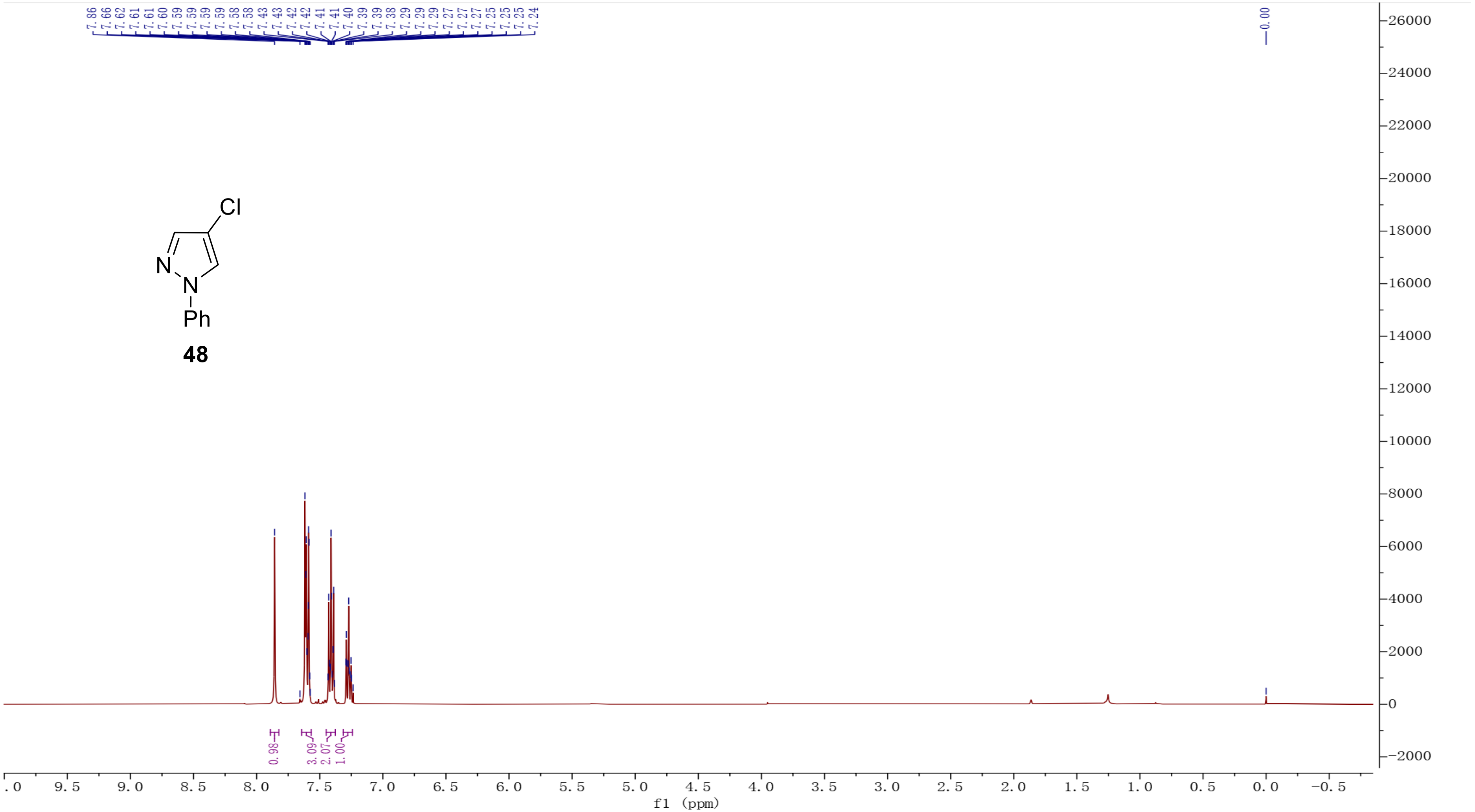

48

Supplementary Figure 119. ^{1}H NMR spectra of compound **48**.

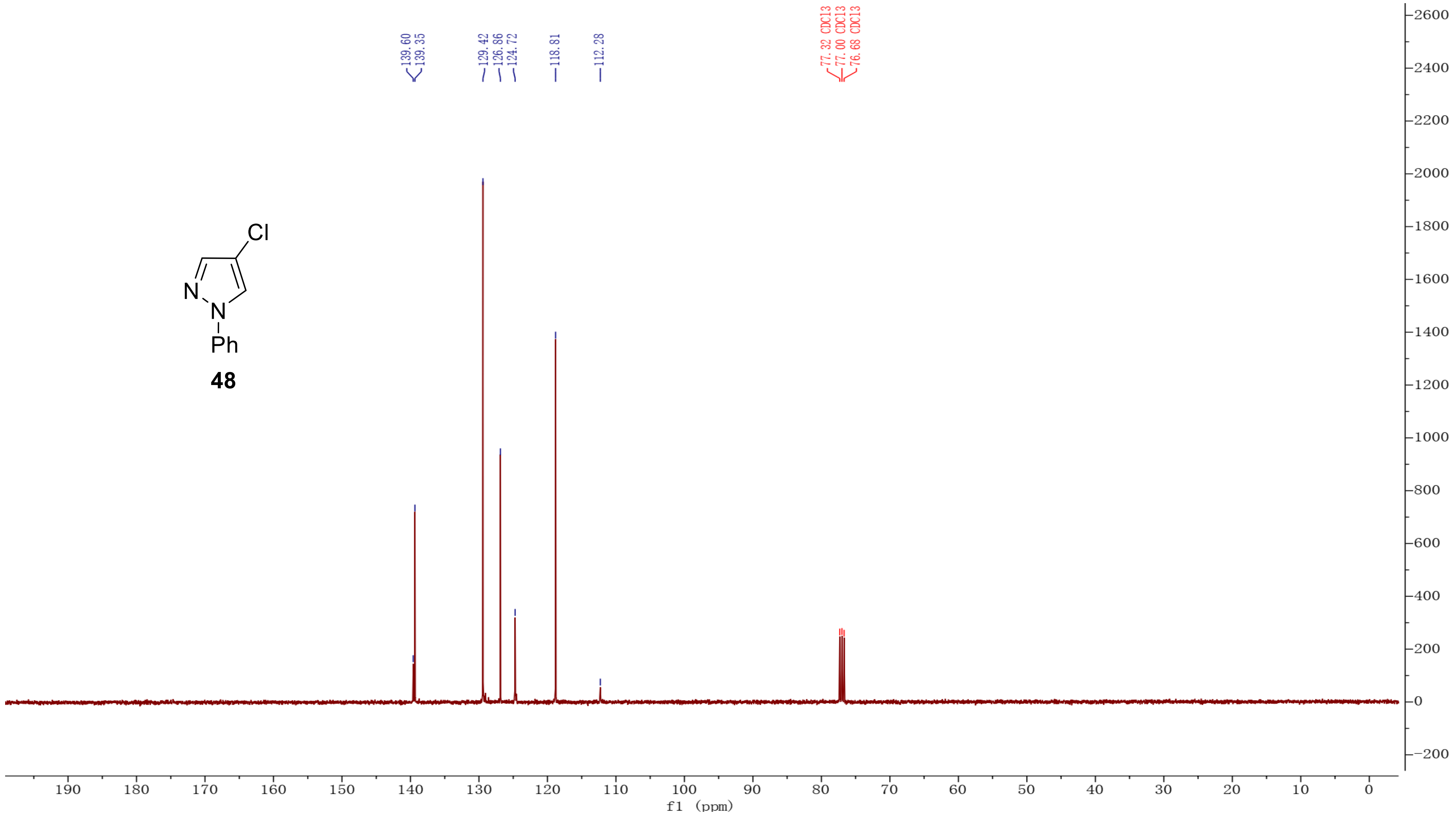

Supplementary Figure 120. ^{13}C NMR spectra of compound **48**.

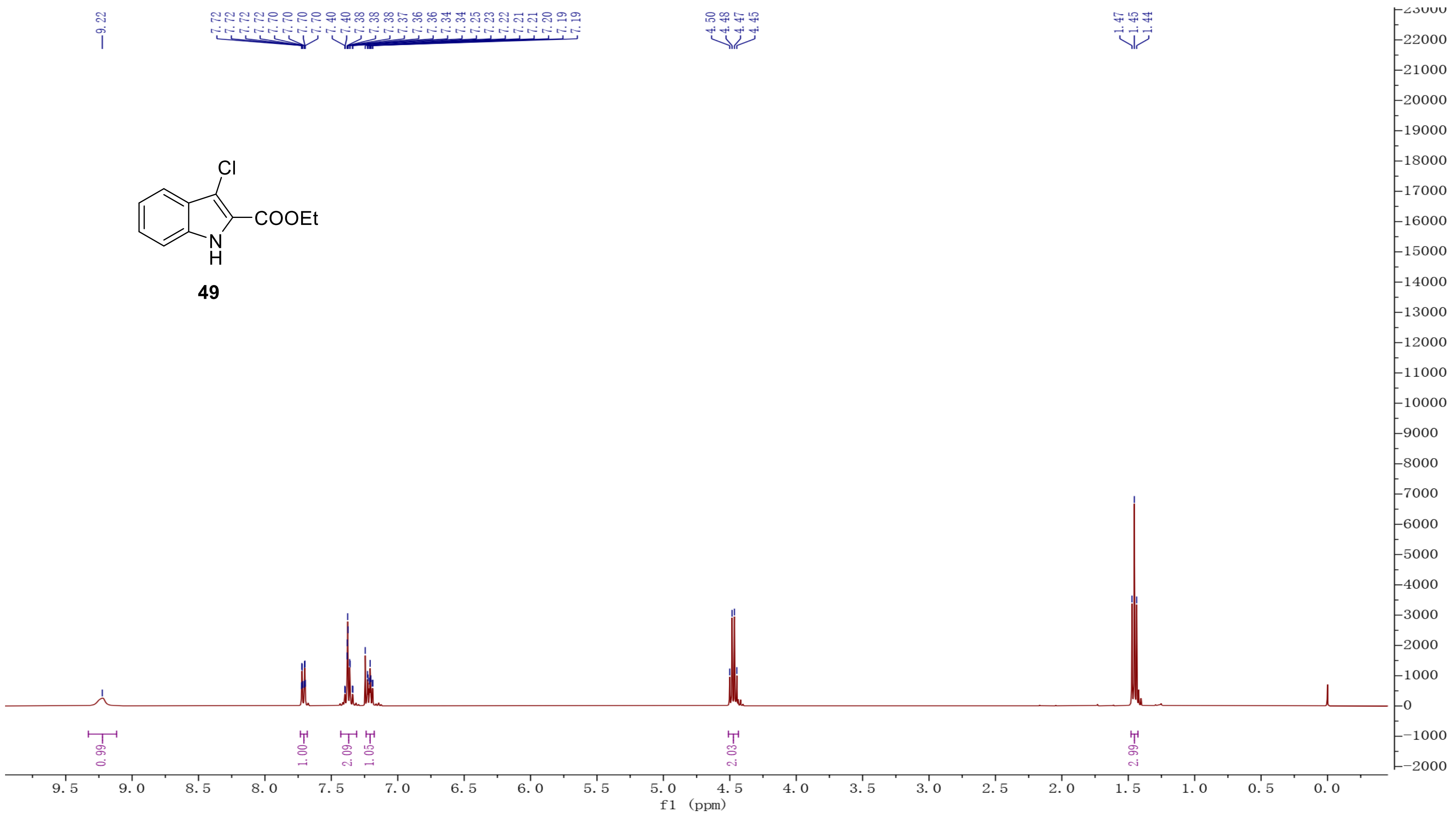

Supplementary Figure 121. ^{1}H NMR spectra of compound **49**.

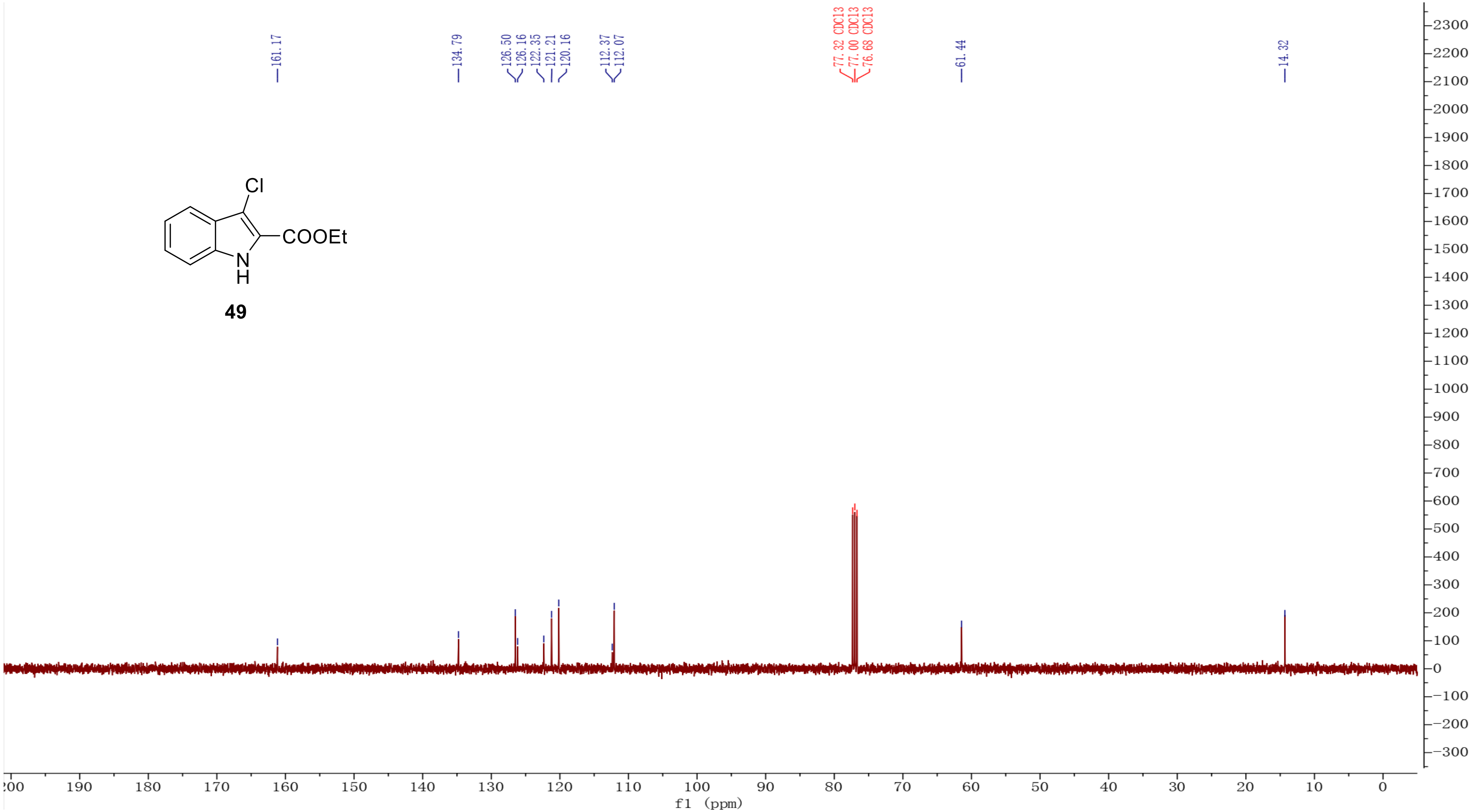

Supplementary Figure 122. ^{13}C NMR spectra of compound **49**.

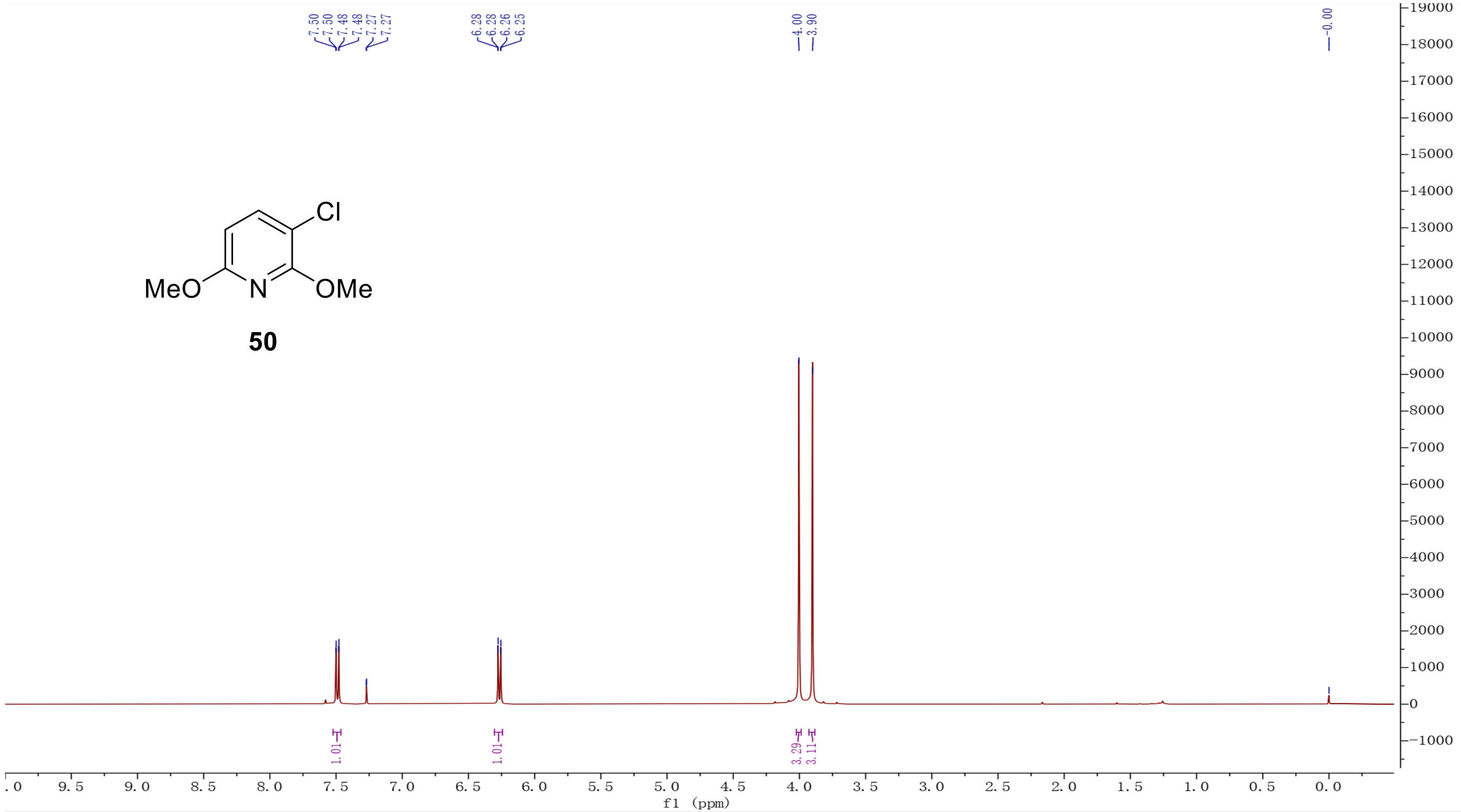

Supplementary Figure 123. ^{1}H NMR spectra of compound **50**.

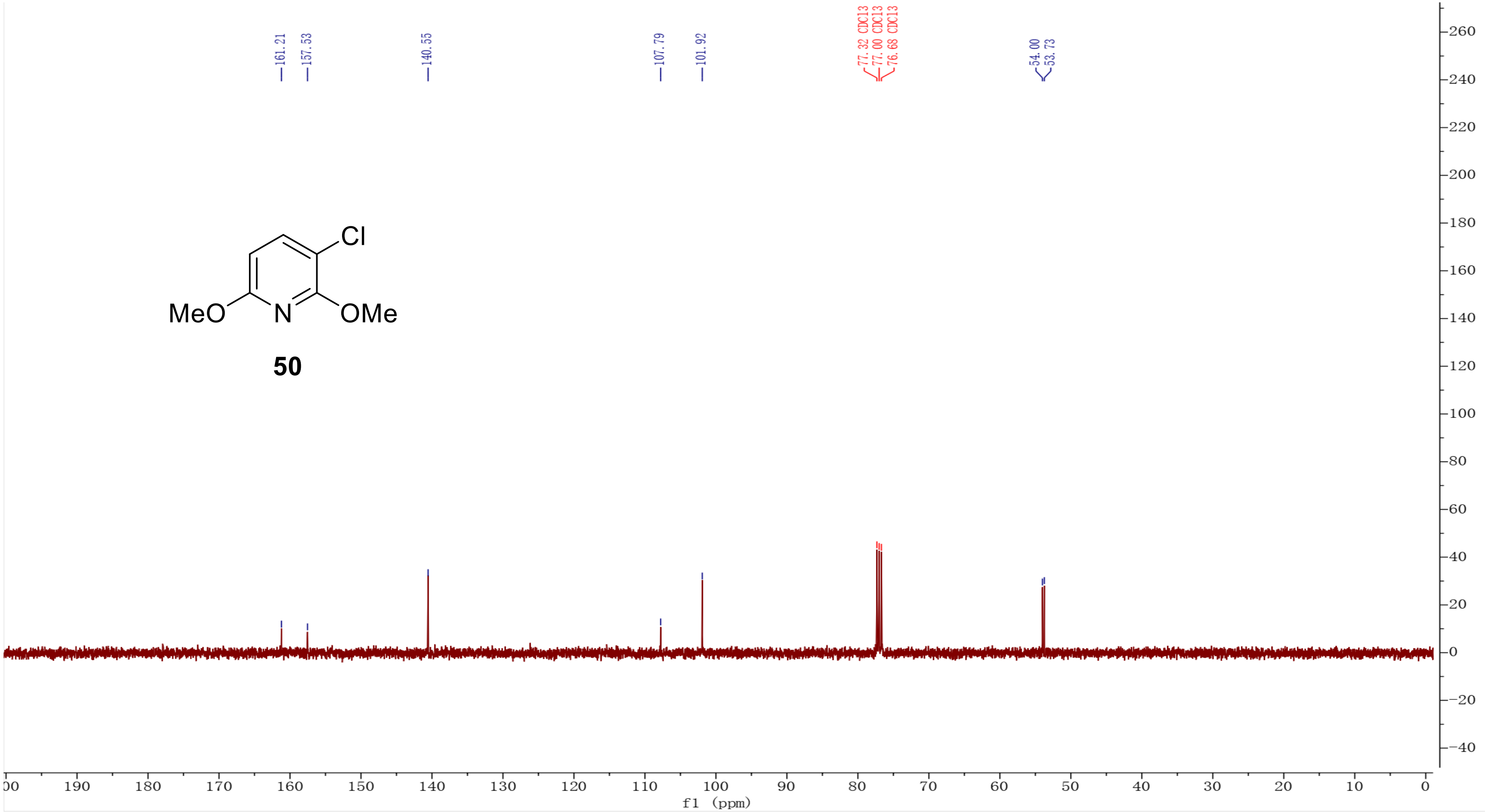

Supplementary Figure 124. ^{13}C NMR spectra of compound **50**.

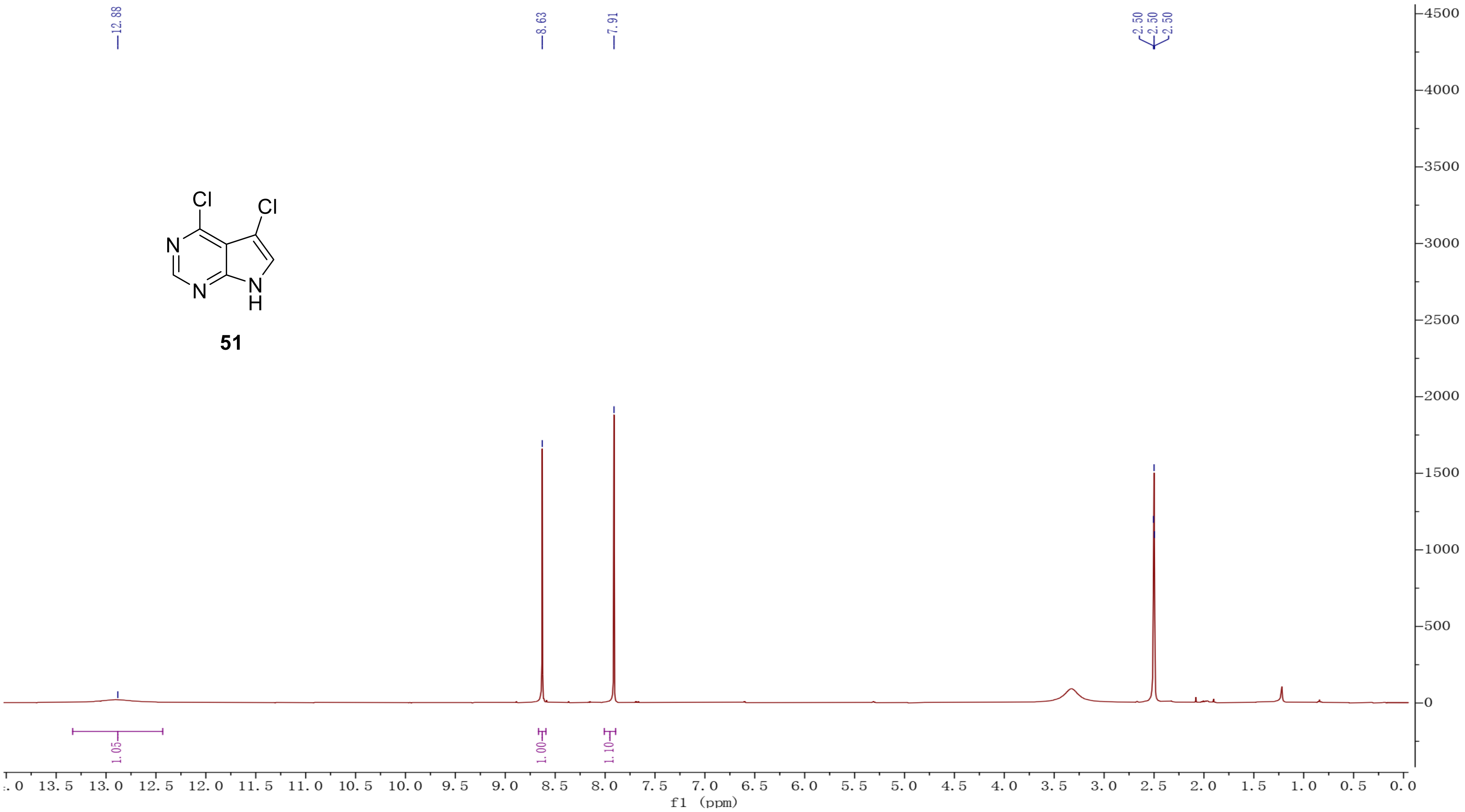

Supplementary Figure 125. ^{1}H NMR spectra of compound **51**.

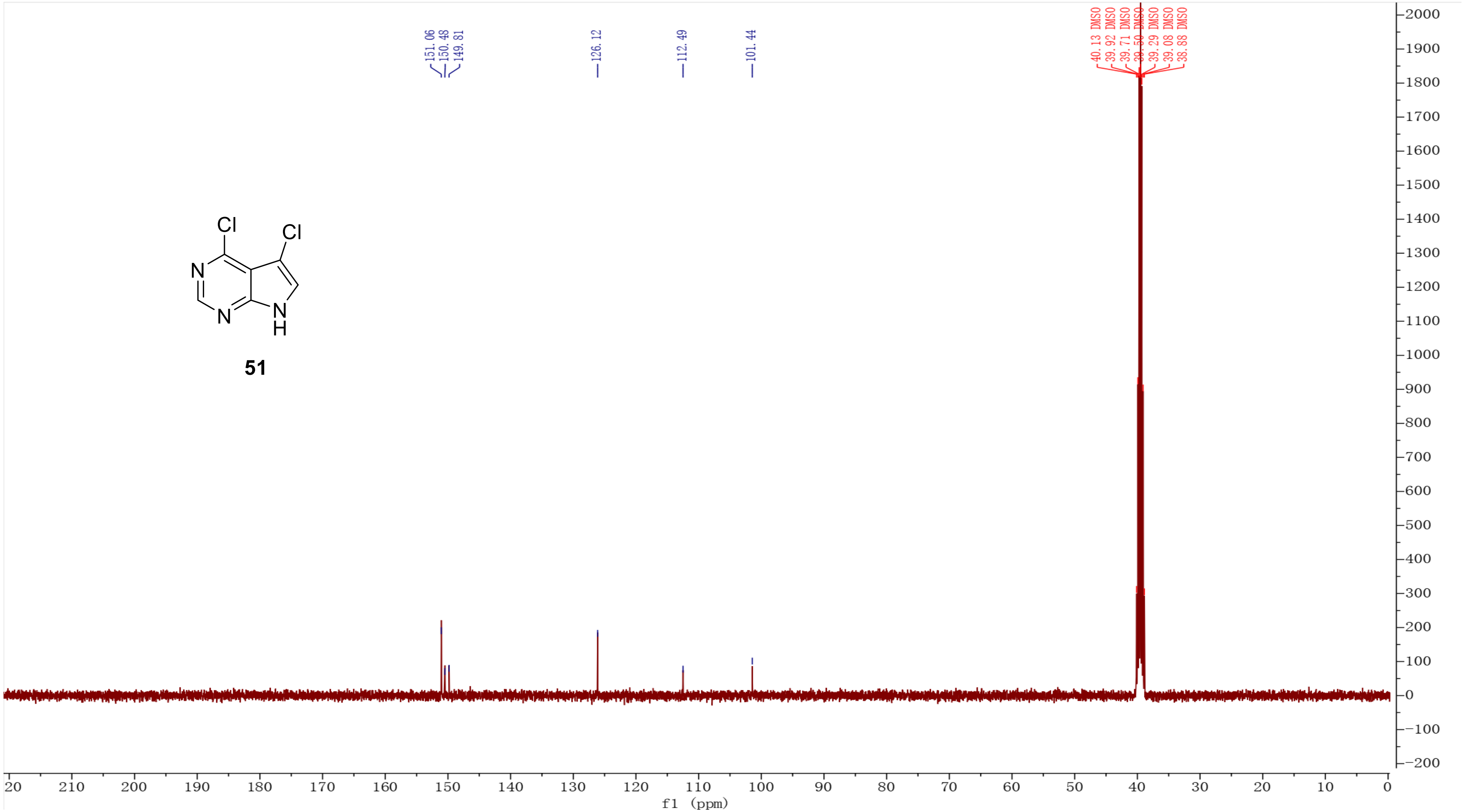

Supplementary Figure 126. ^{13}C NMR spectra of compound **51**.

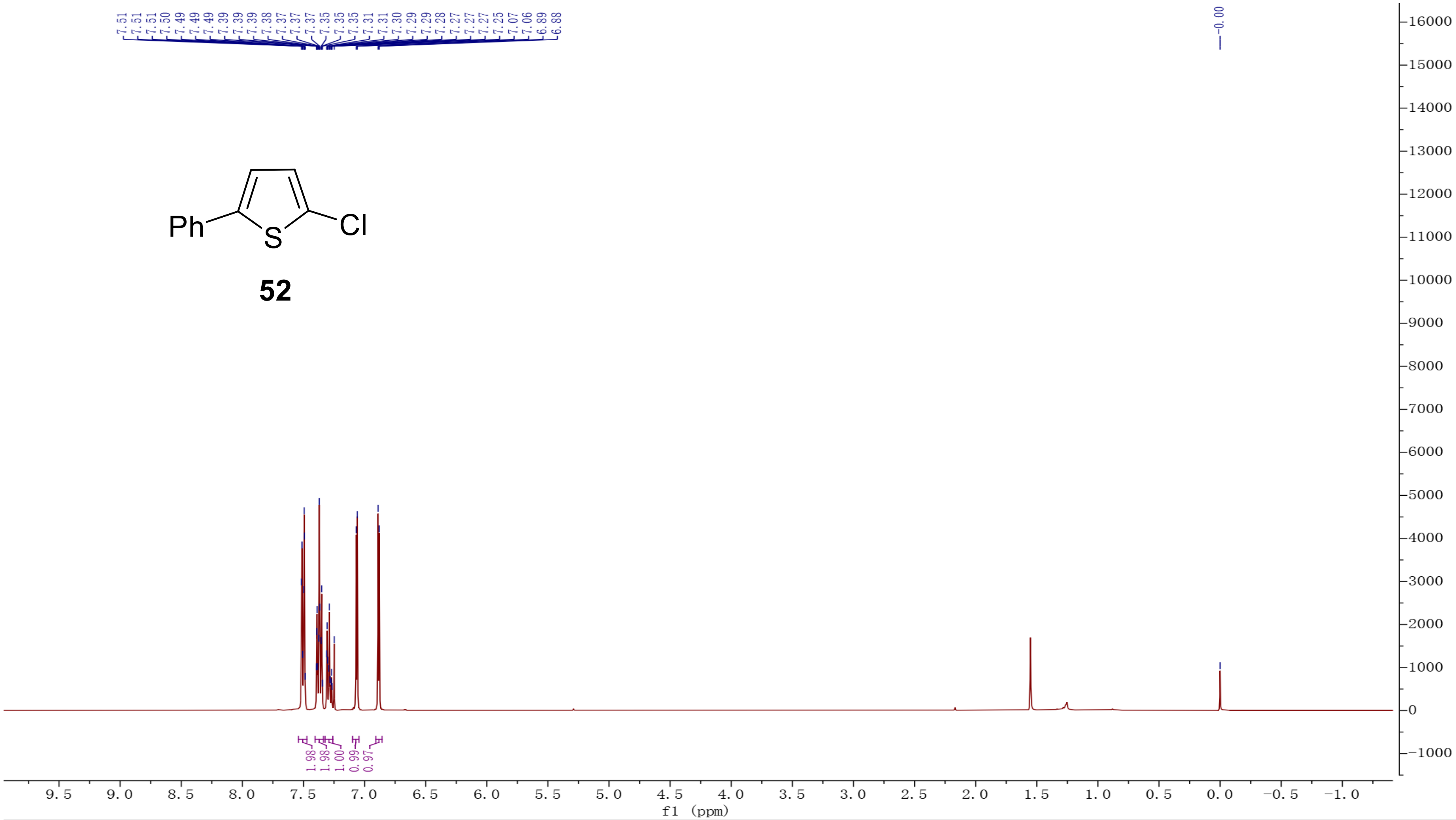

Supplementary Figure 127. ^{1}H NMR spectra of compound **52**.

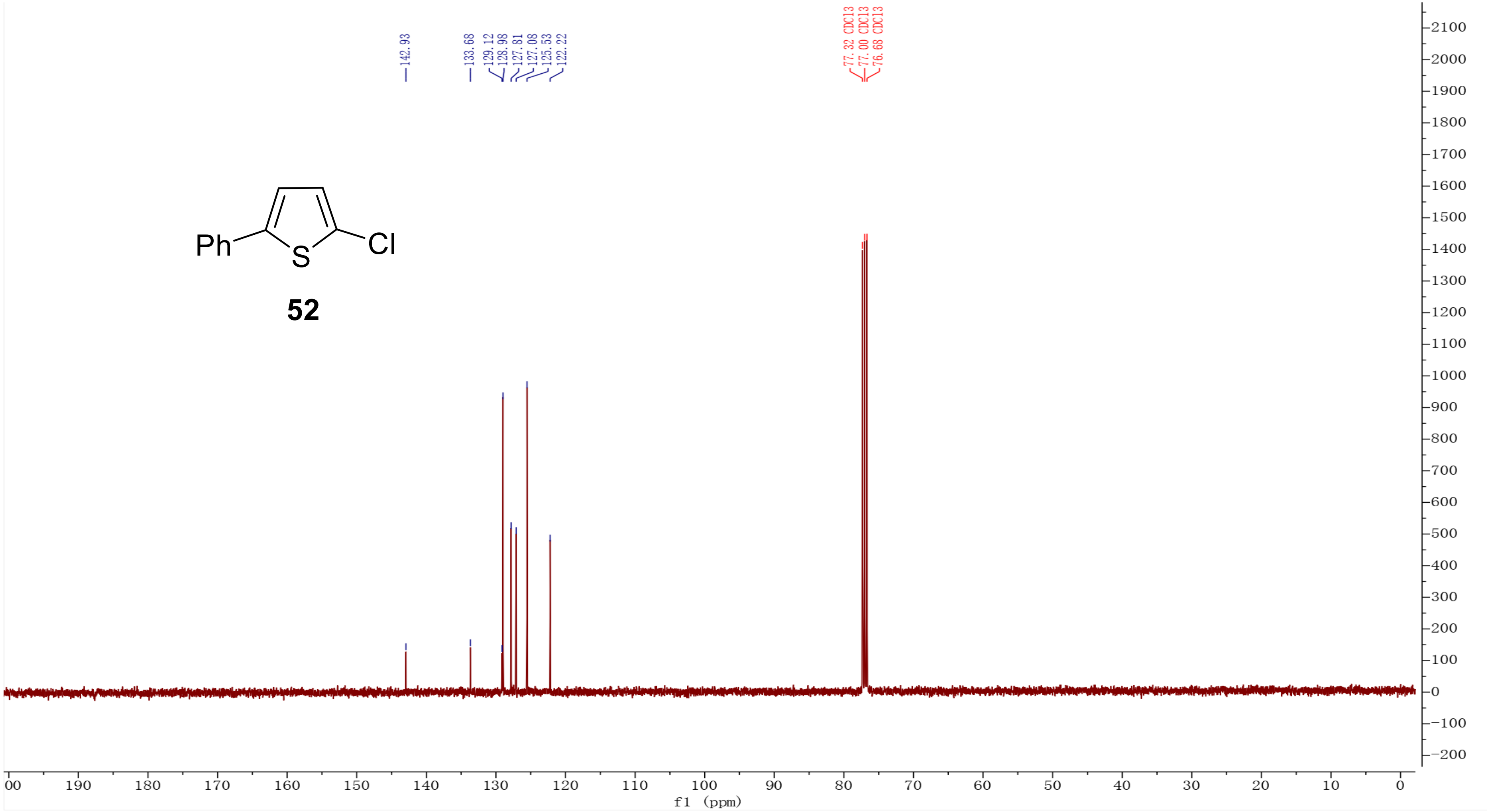

Supplementary Figure 128. ^{13}C NMR spectra of compound **52**.

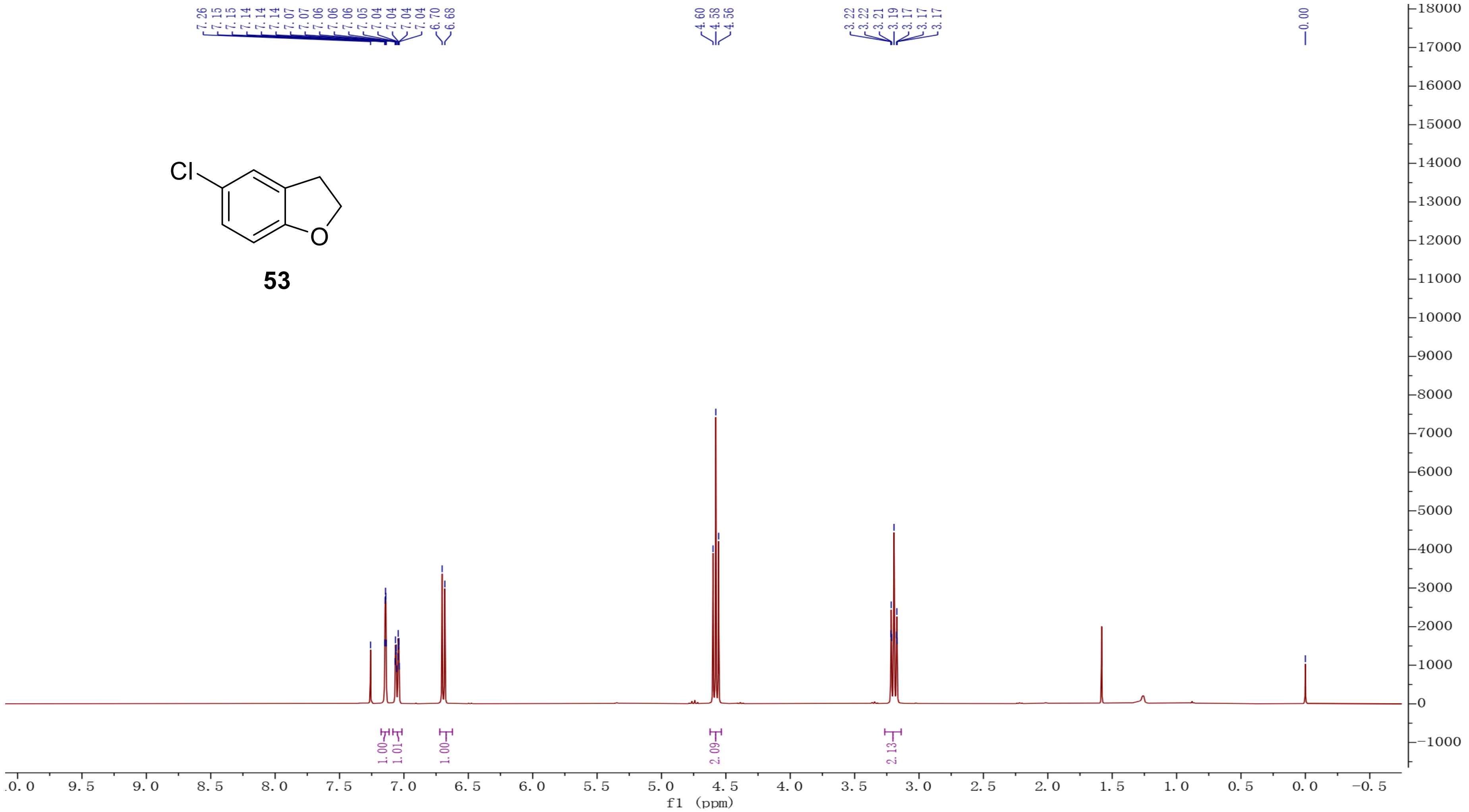

Supplementary Figure 129. ^{1}H NMR spectra of compound **53**.

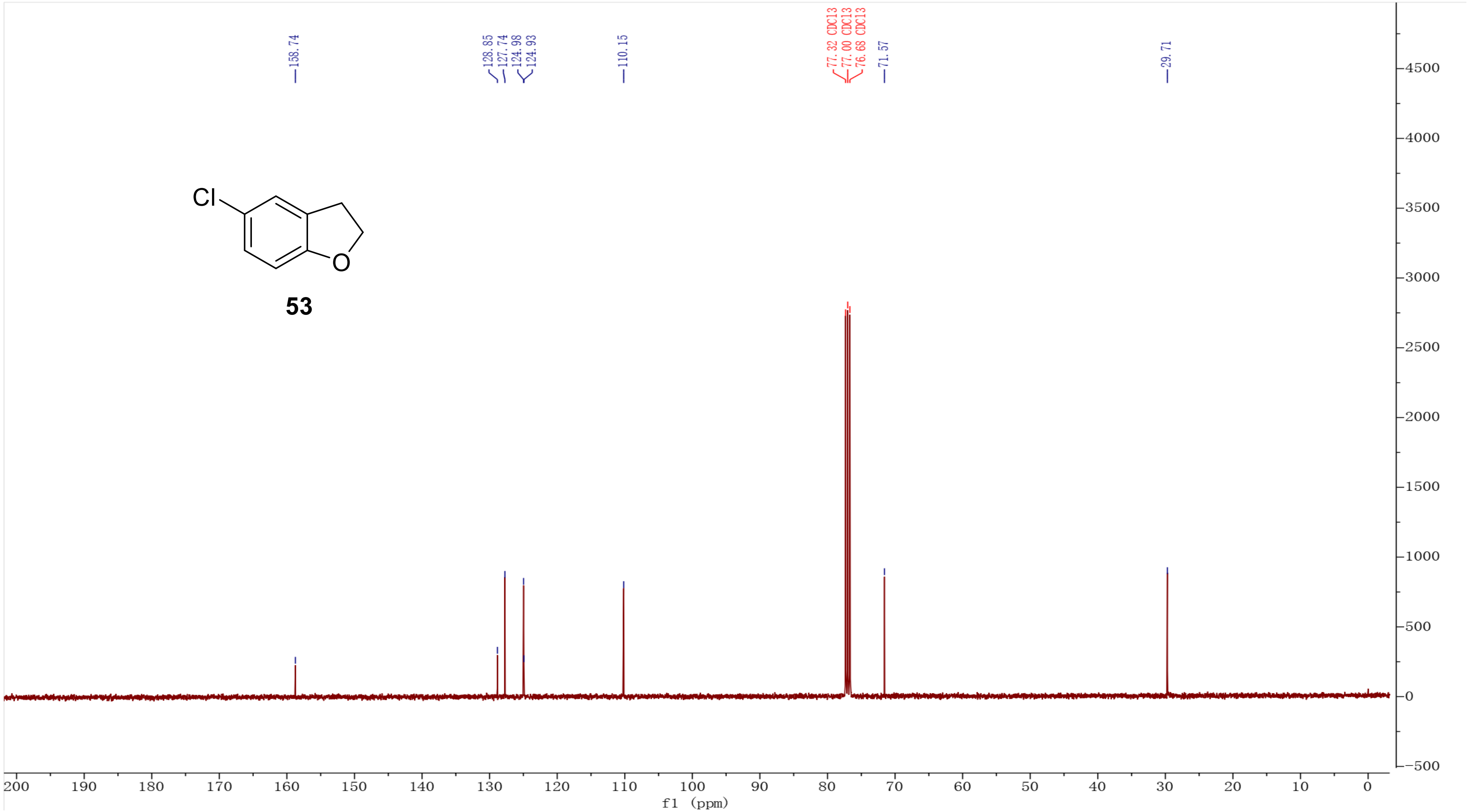

Supplementary Figure 130. ^{13}C NMR spectra of compound **31**.

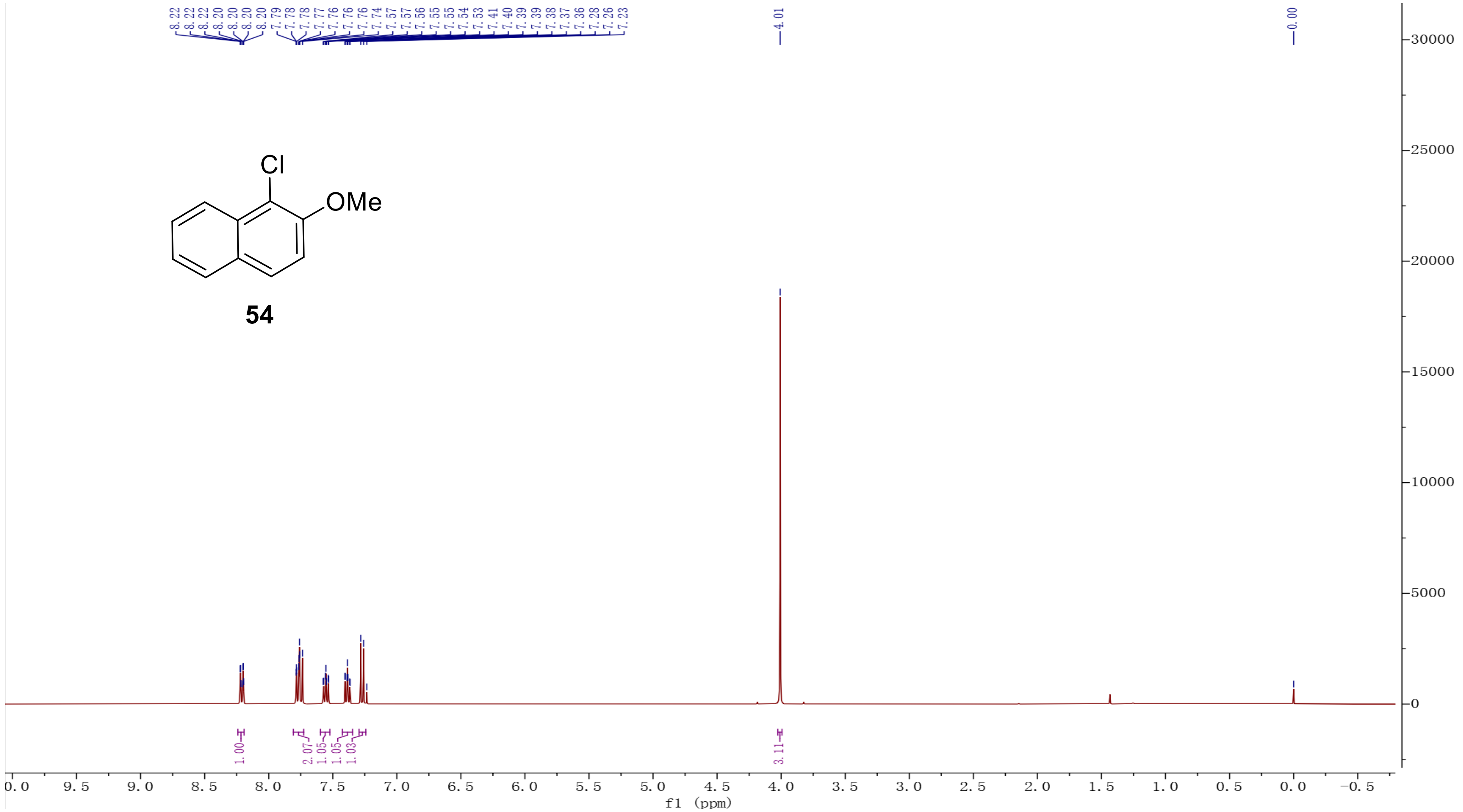

Supplementary Figure 131. ^{1}H NMR spectra of compound **54**.

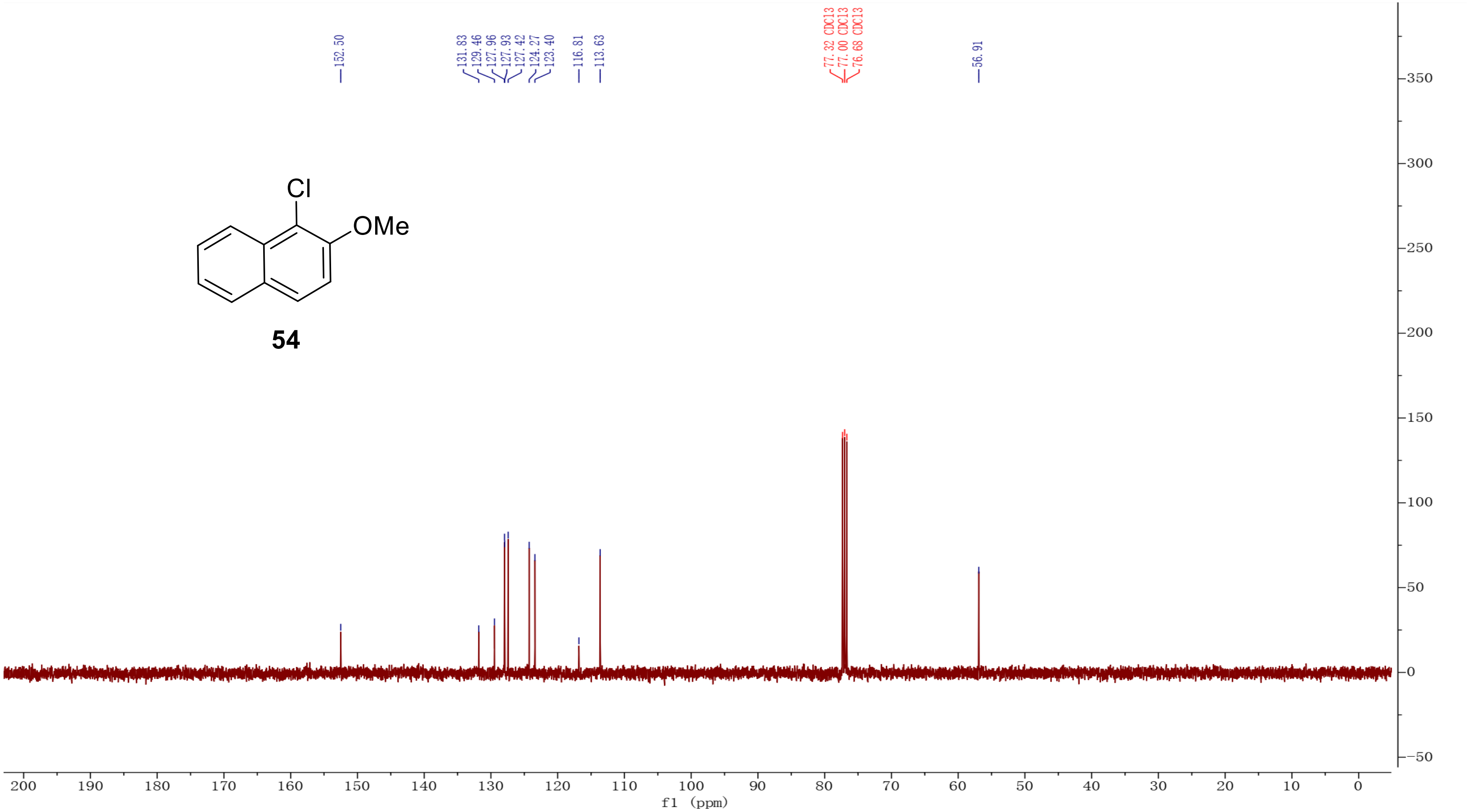

Supplementary Figure 132. ^{13}C NMR spectra of compound **54**.

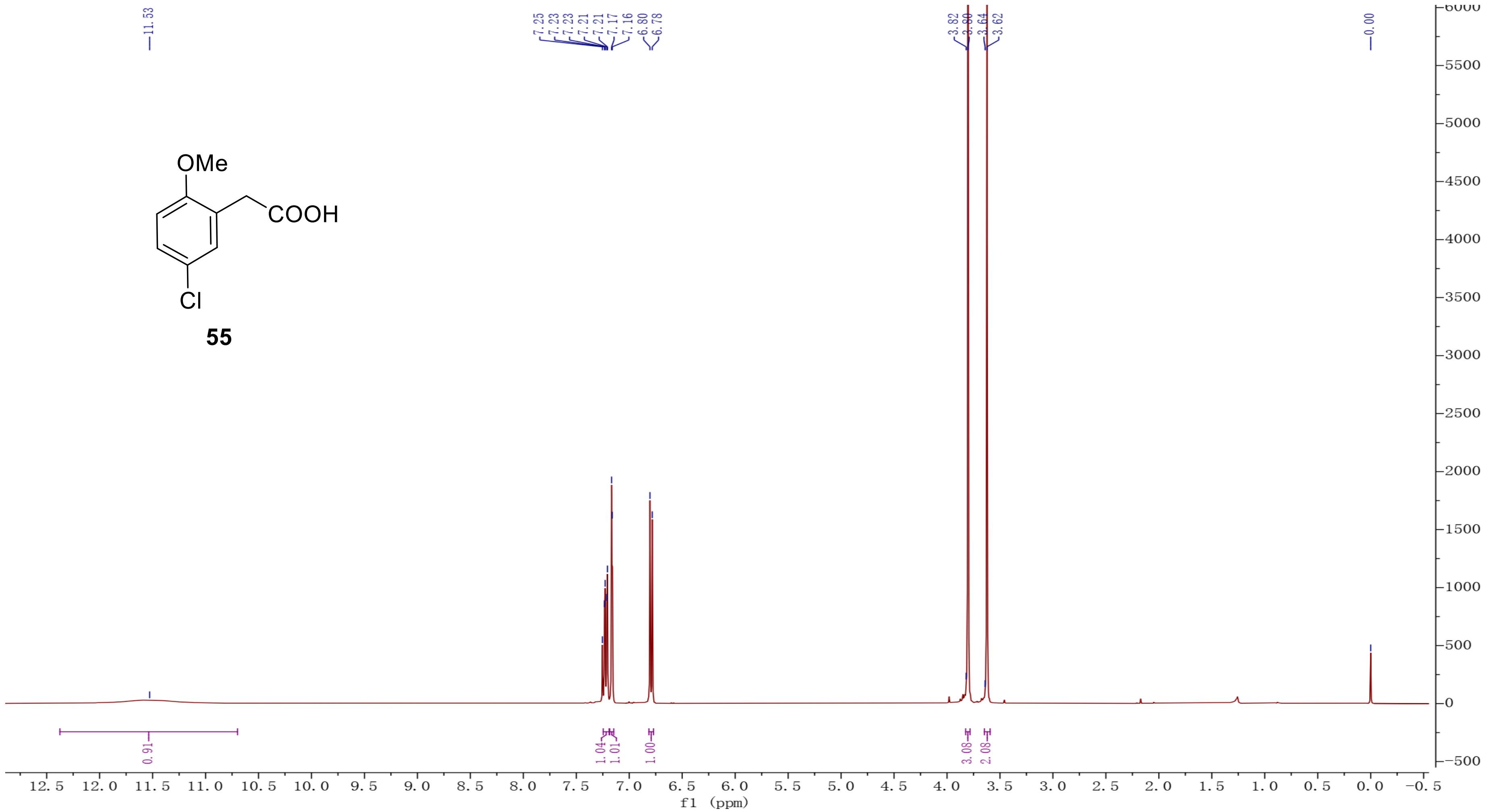

Supplementary Figure 133. ^{1}H NMR spectra of compound **55**.

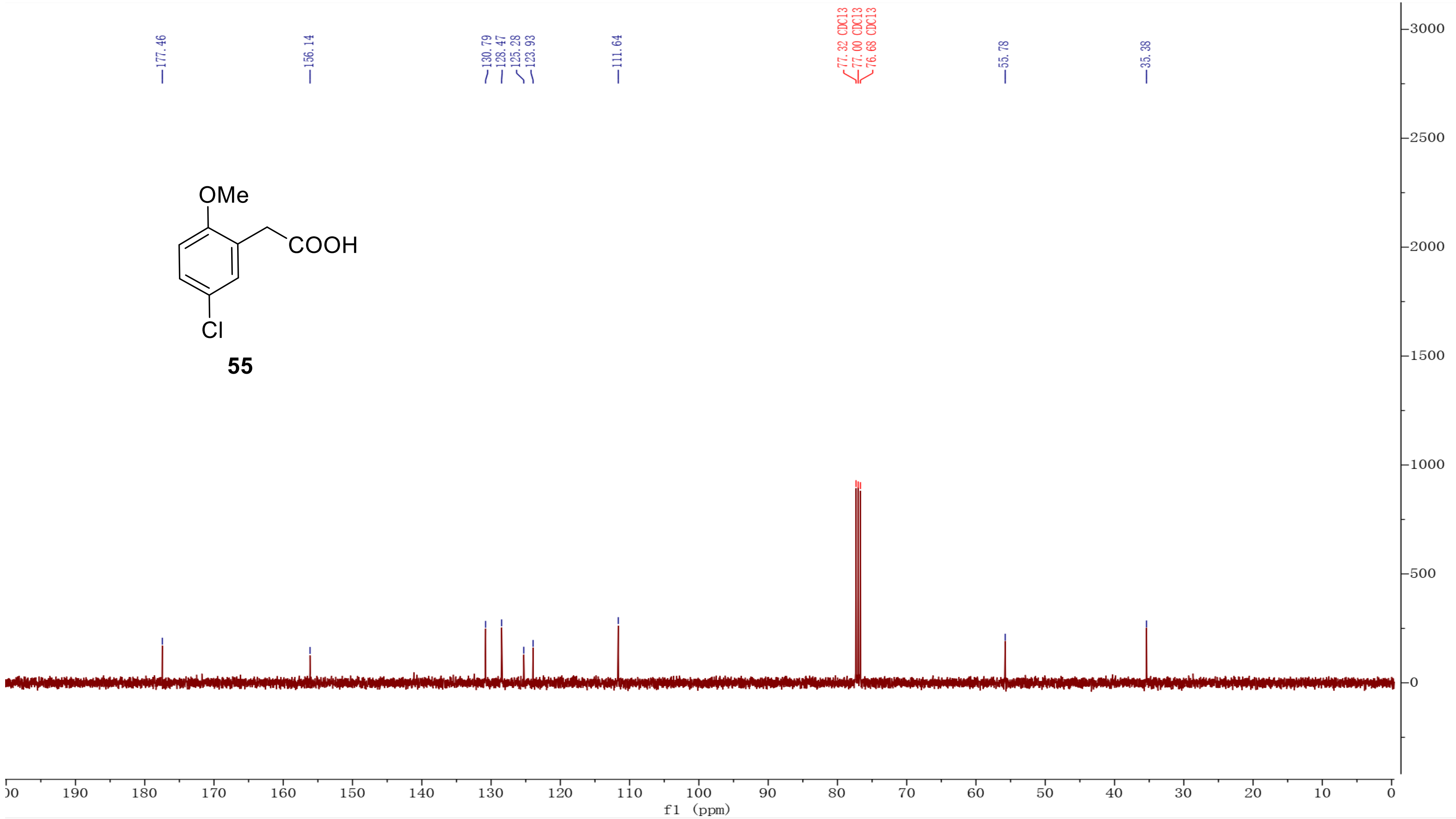

Supplementary Figure 134. ^{13}C NMR spectra of compound **55**.

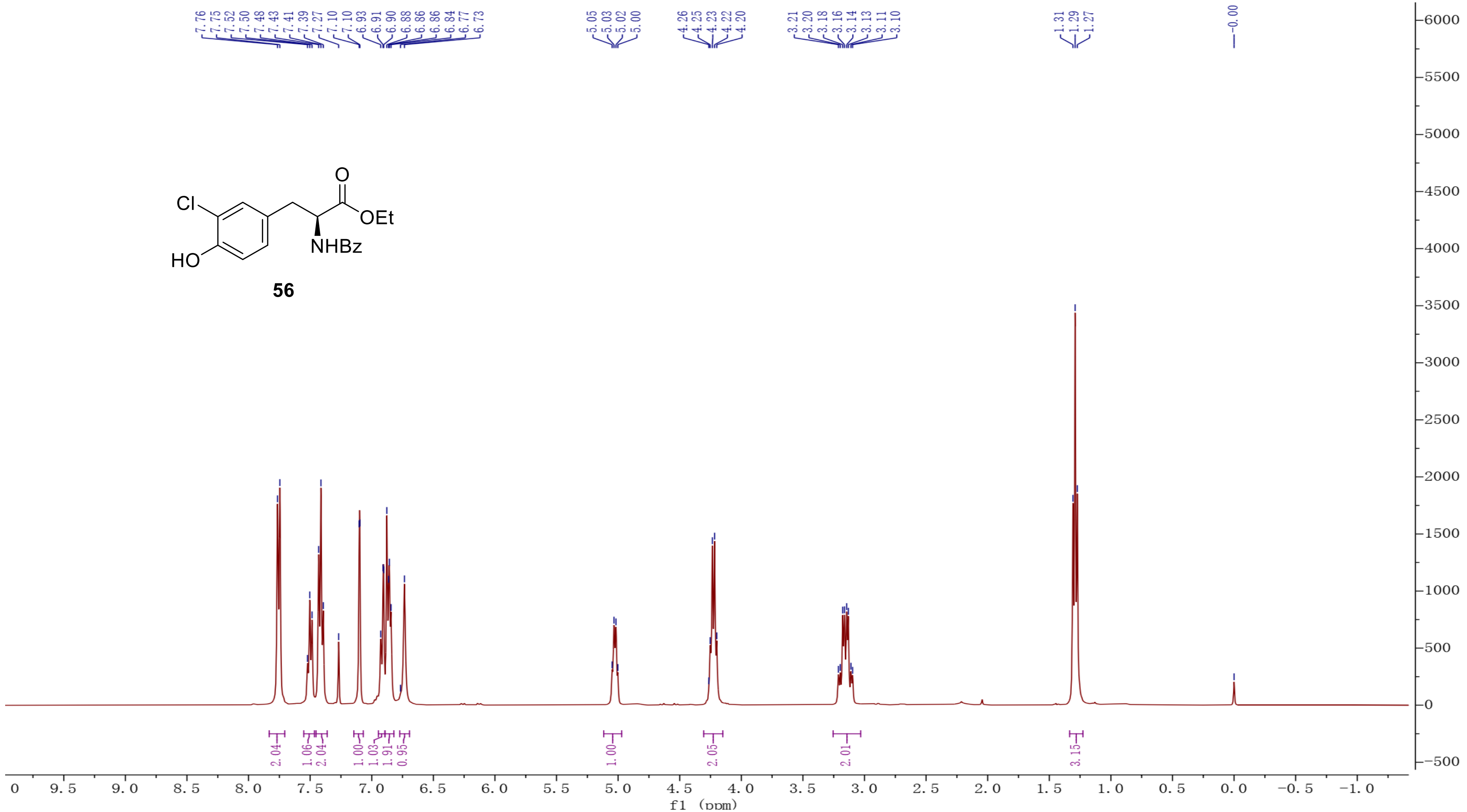

Supplementary Figure 135. ^{1}H NMR spectra of compound **56**.

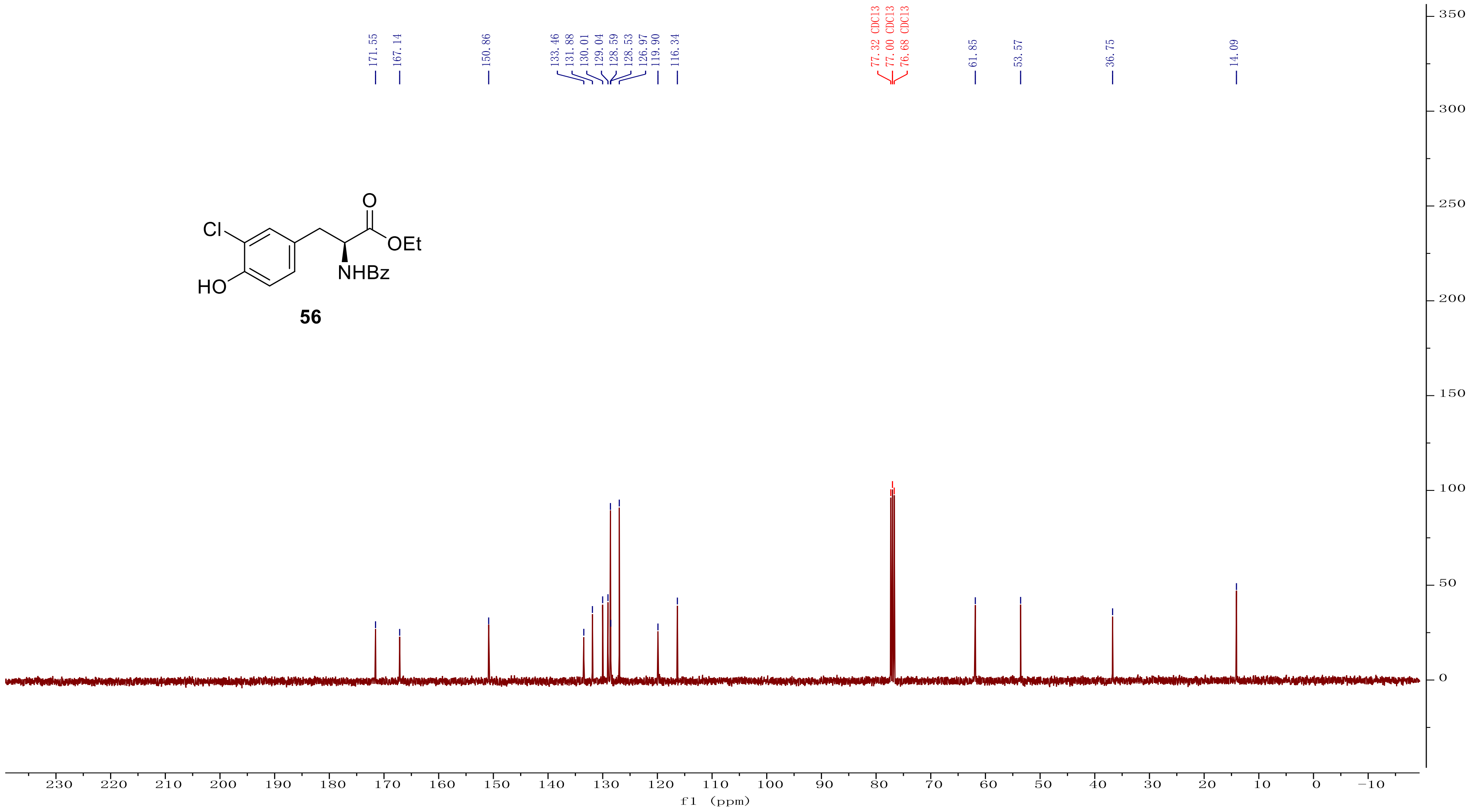

Supplementary Figure 136. ^{13}C NMR spectra of compound **56**.

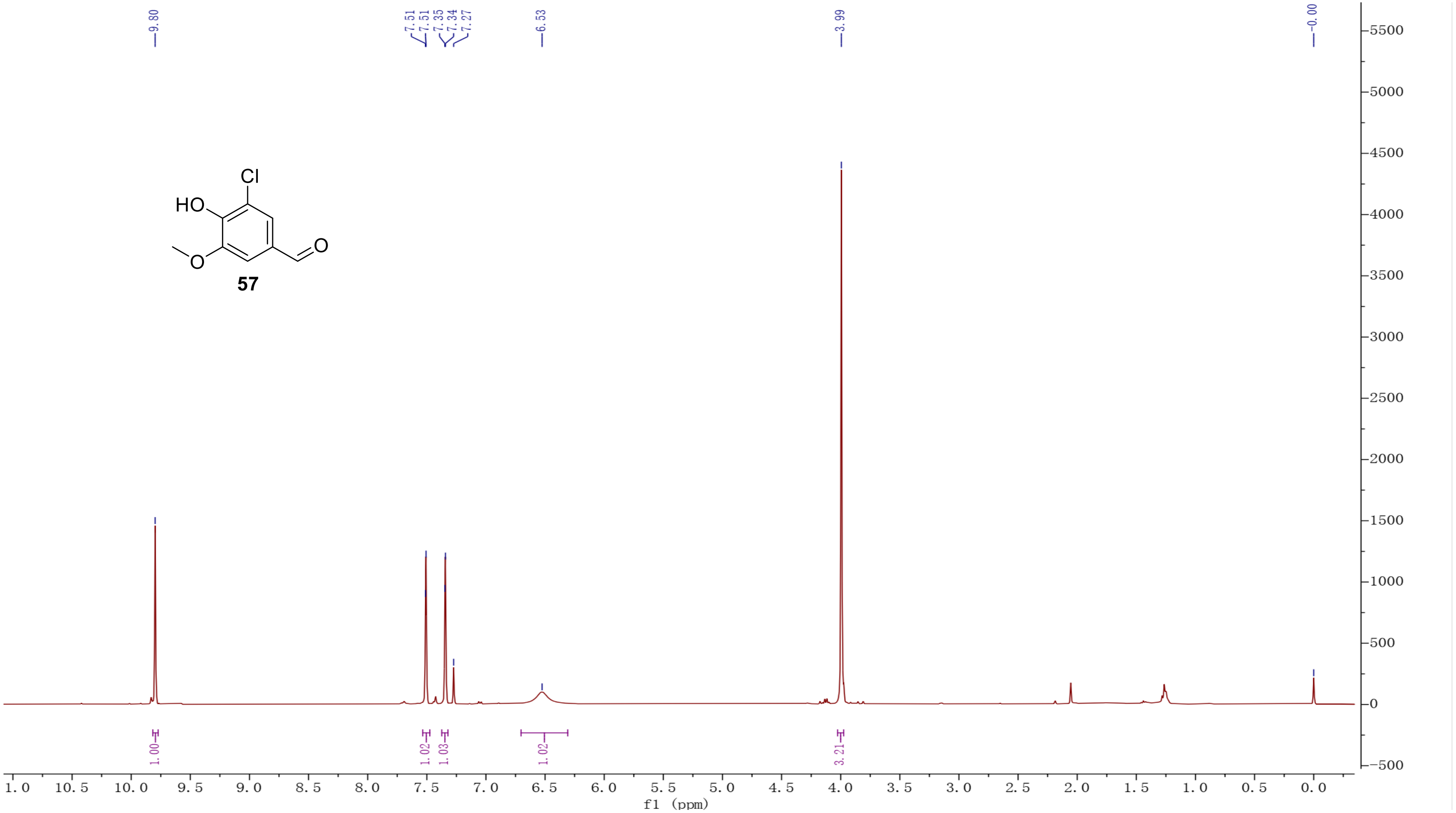

Supplementary Figure 137. ^{1}H NMR spectra of compound **57**.

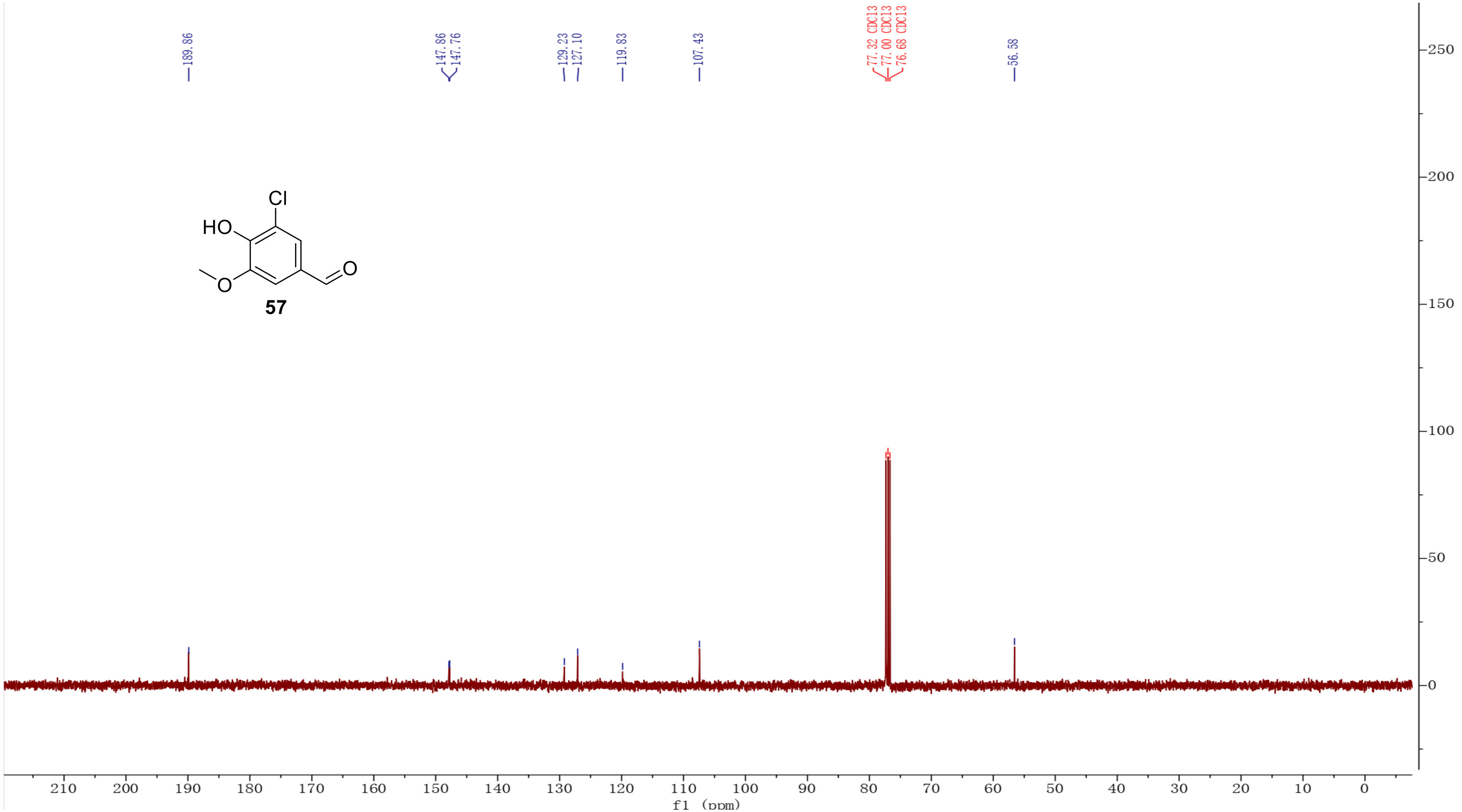

Supplementary Figure 138. ^{13}C NMR spectra of compound **57**.

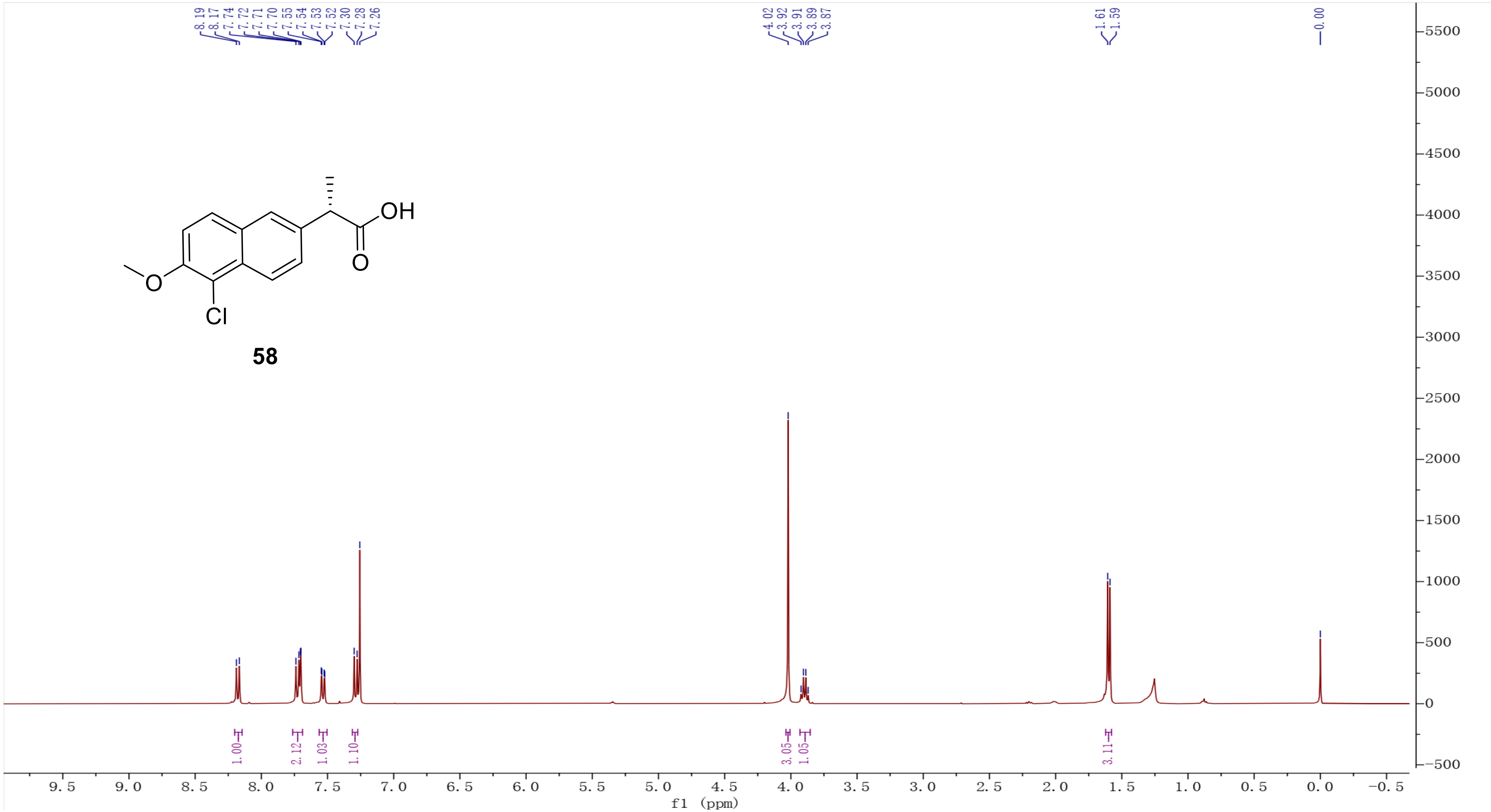

Supplementary Figure 139. ^{1}H NMR spectra of compound **58**.

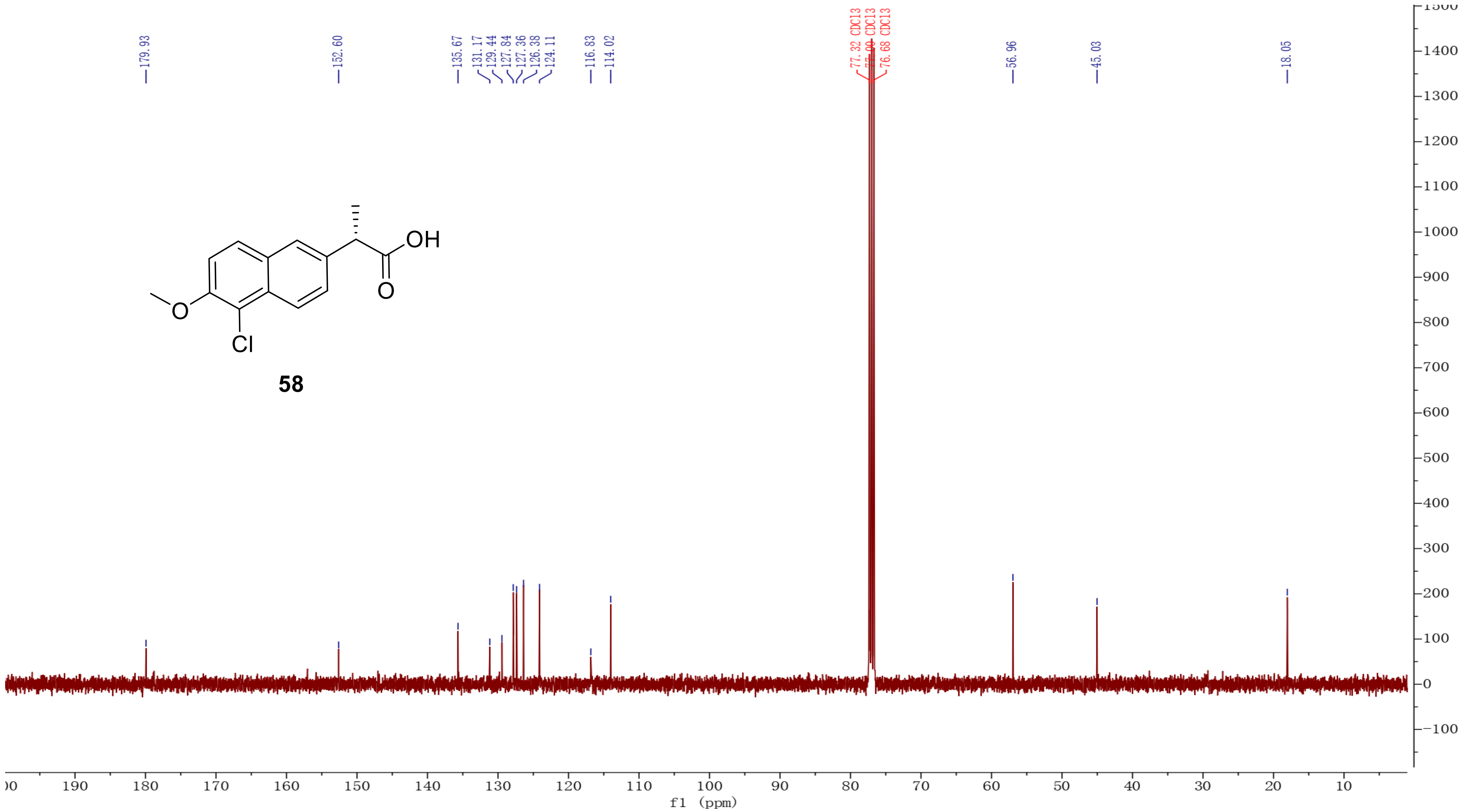

Supplementary Figure 140. ^{13}C NMR spectra of compound **58**.

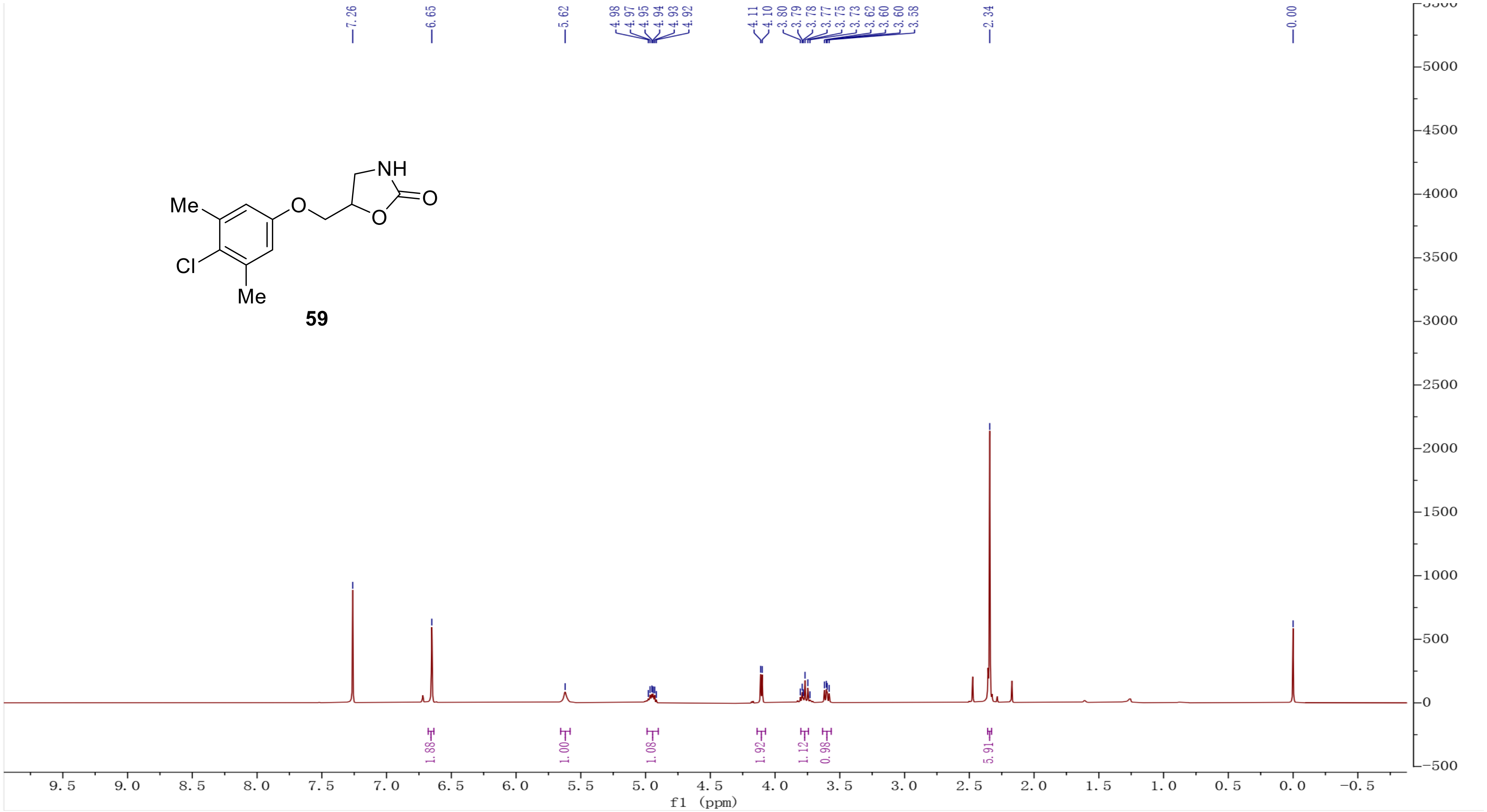

Supplementary Figure 141. ^{1}H NMR spectra of compound **59**.

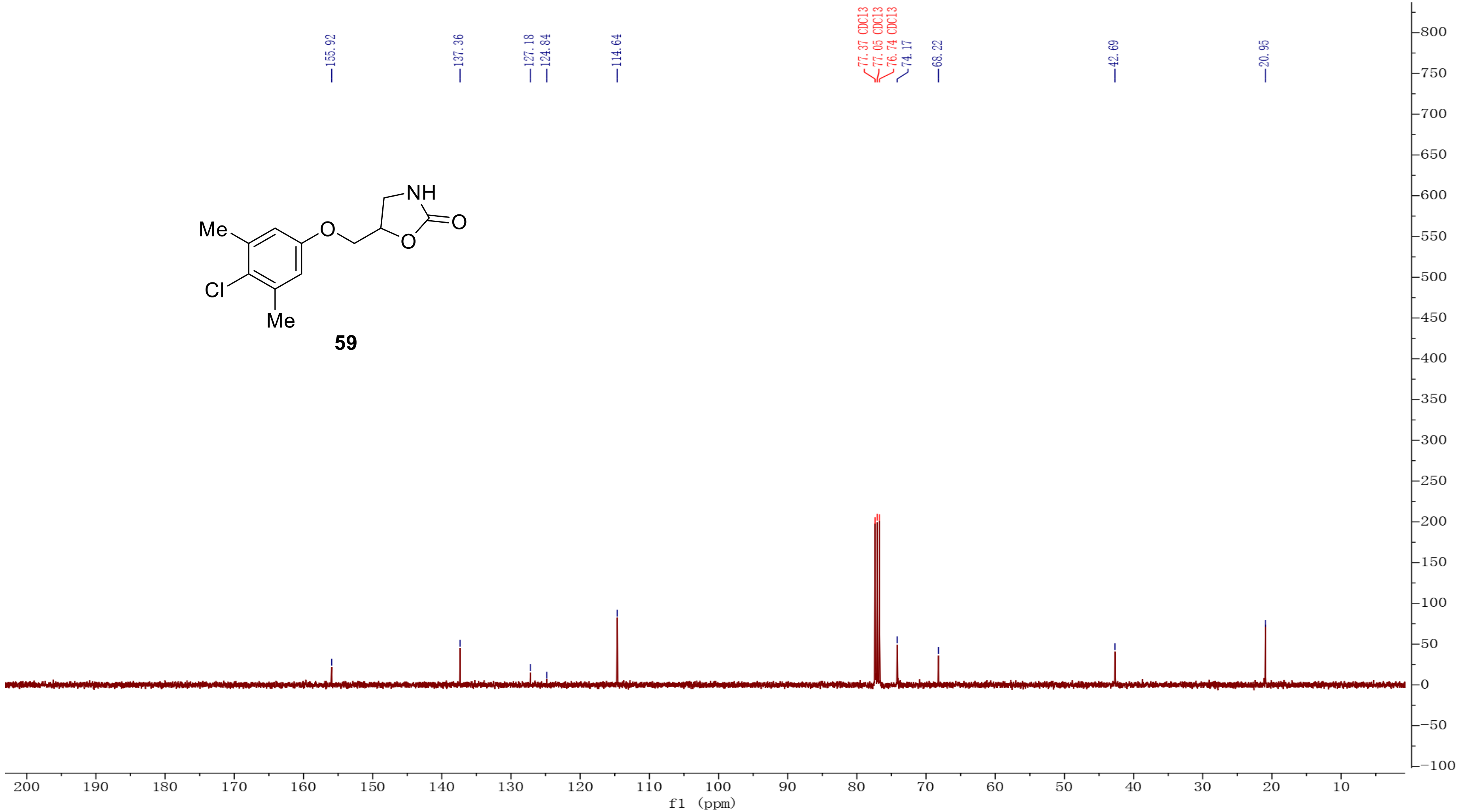

Supplementary Figure 142. ^{13}C NMR spectra of compound **59**.

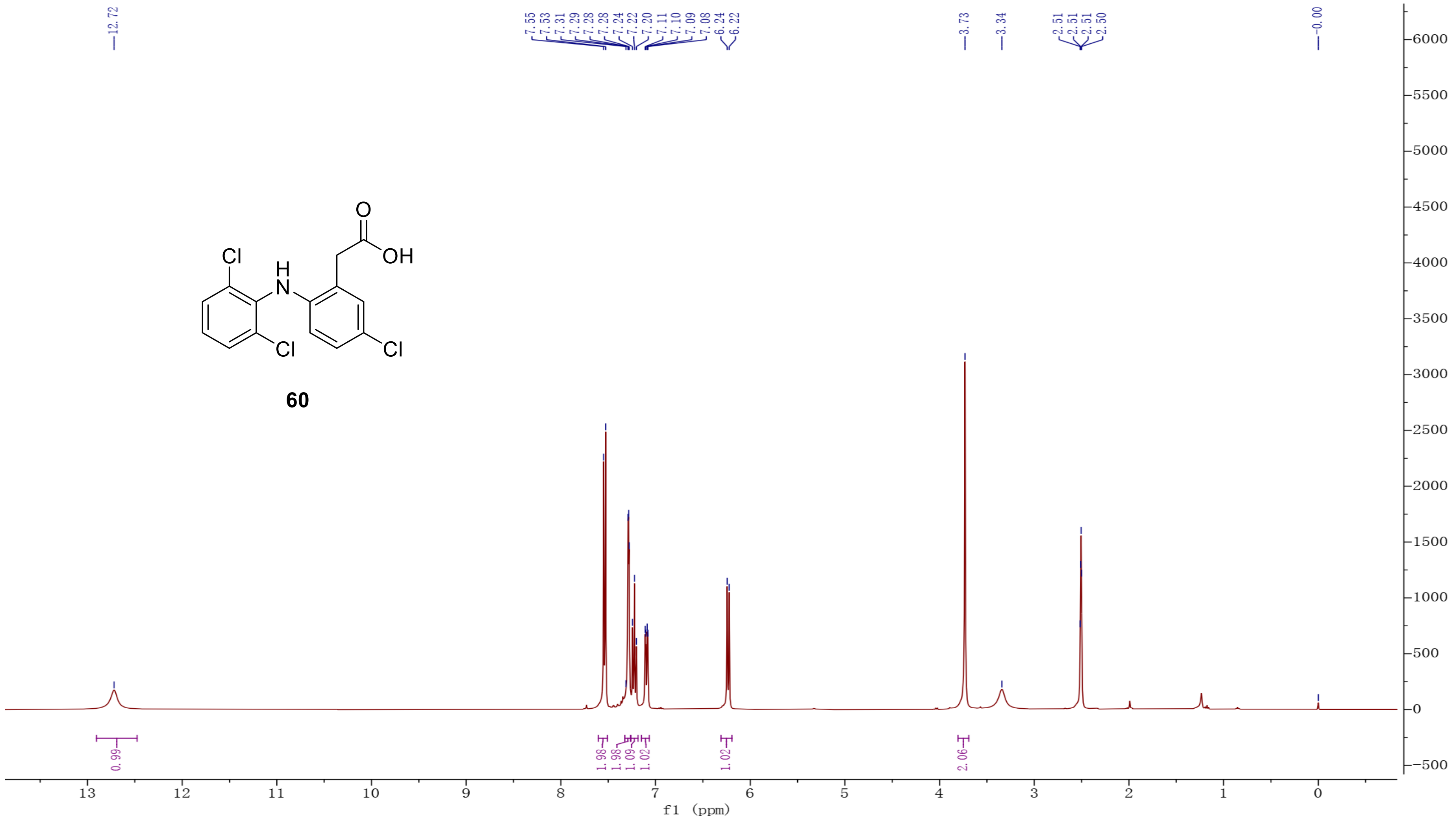

Supplementary Figure 143. ^{1}H NMR spectra of compound **60**.

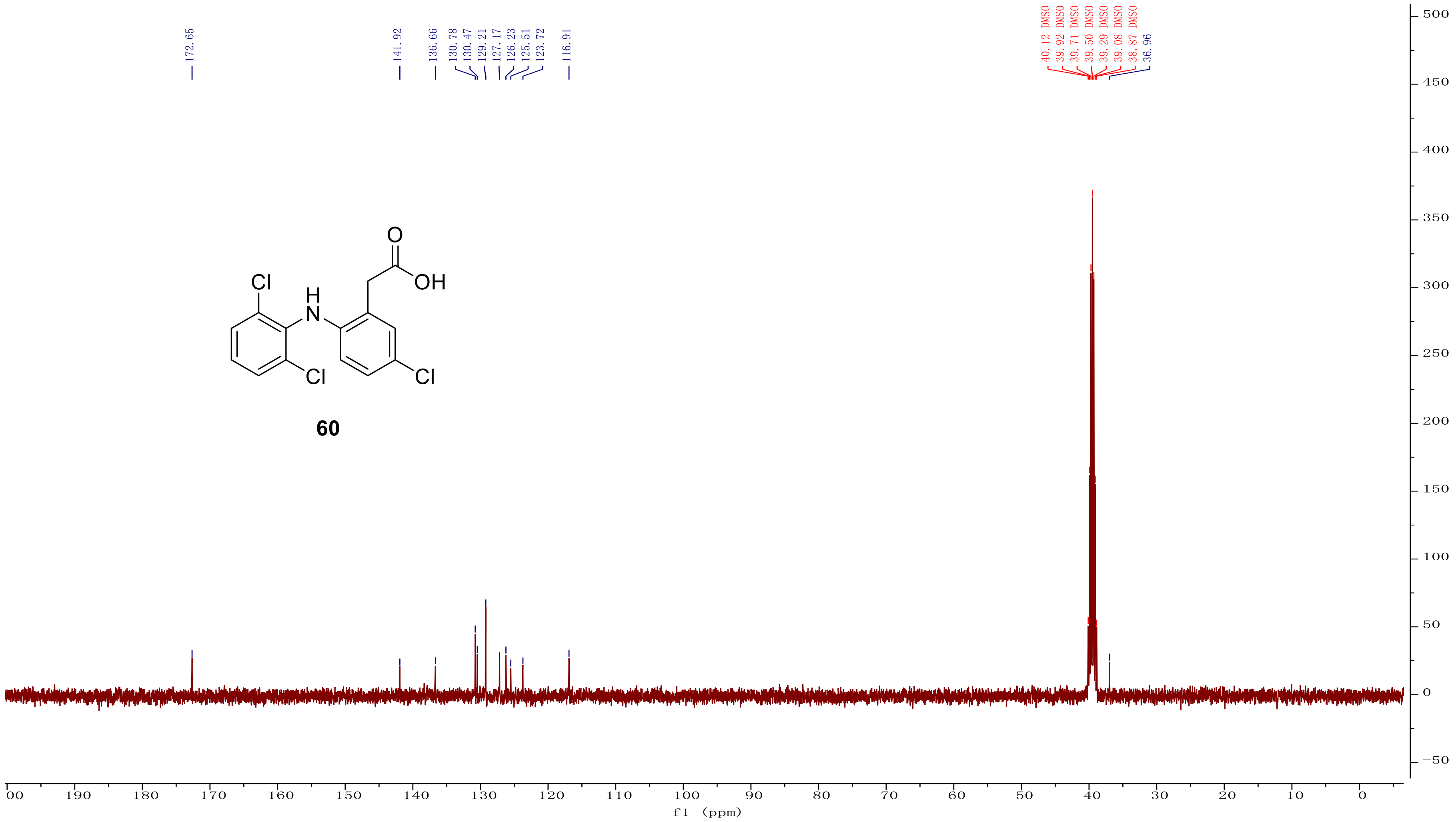

Supplementary Figure 144. ^{13}C NMR spectra of compound **60**.

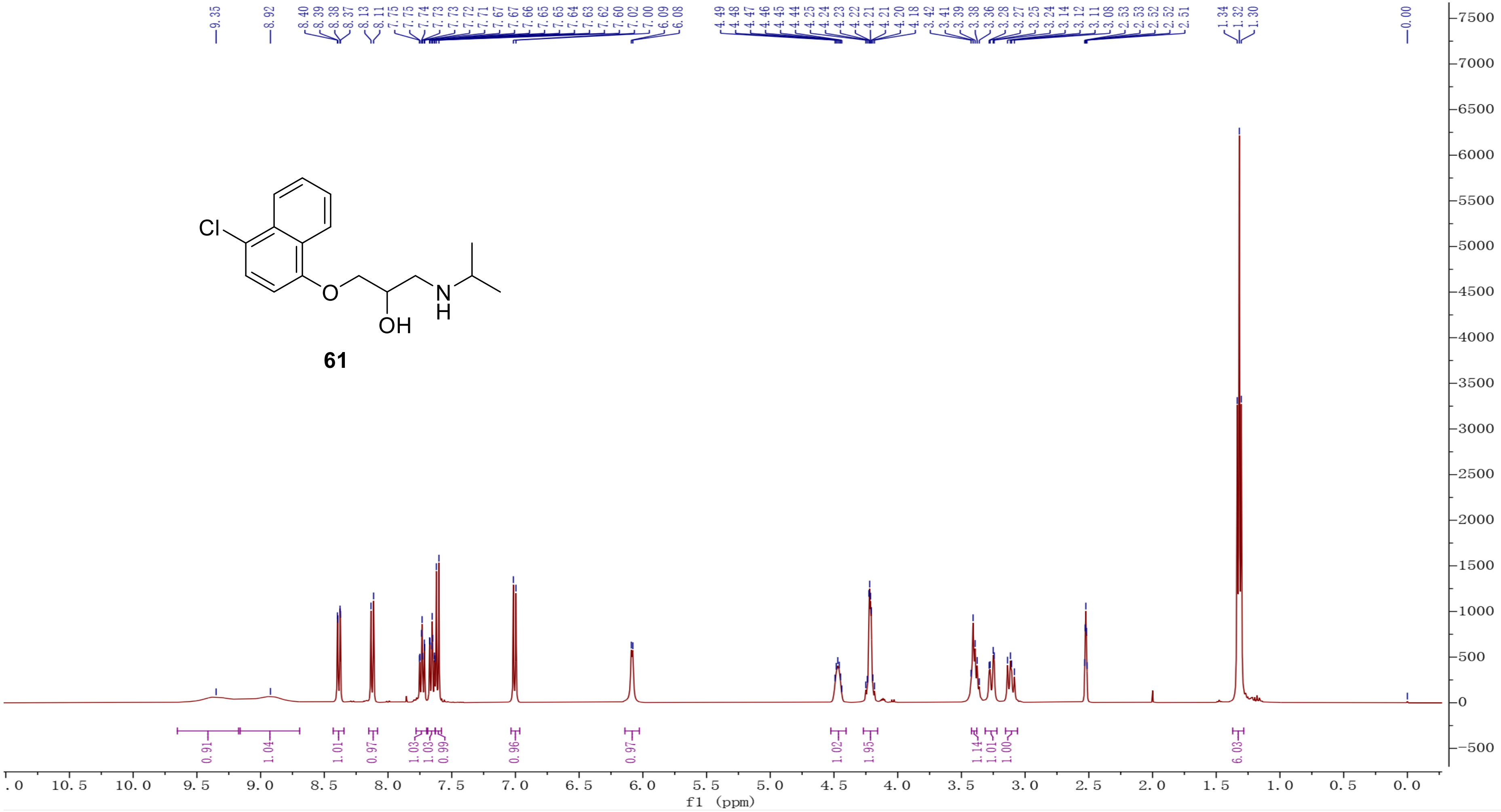

Supplementary Figure 145. ^{1}H NMR spectra of compound **61**.

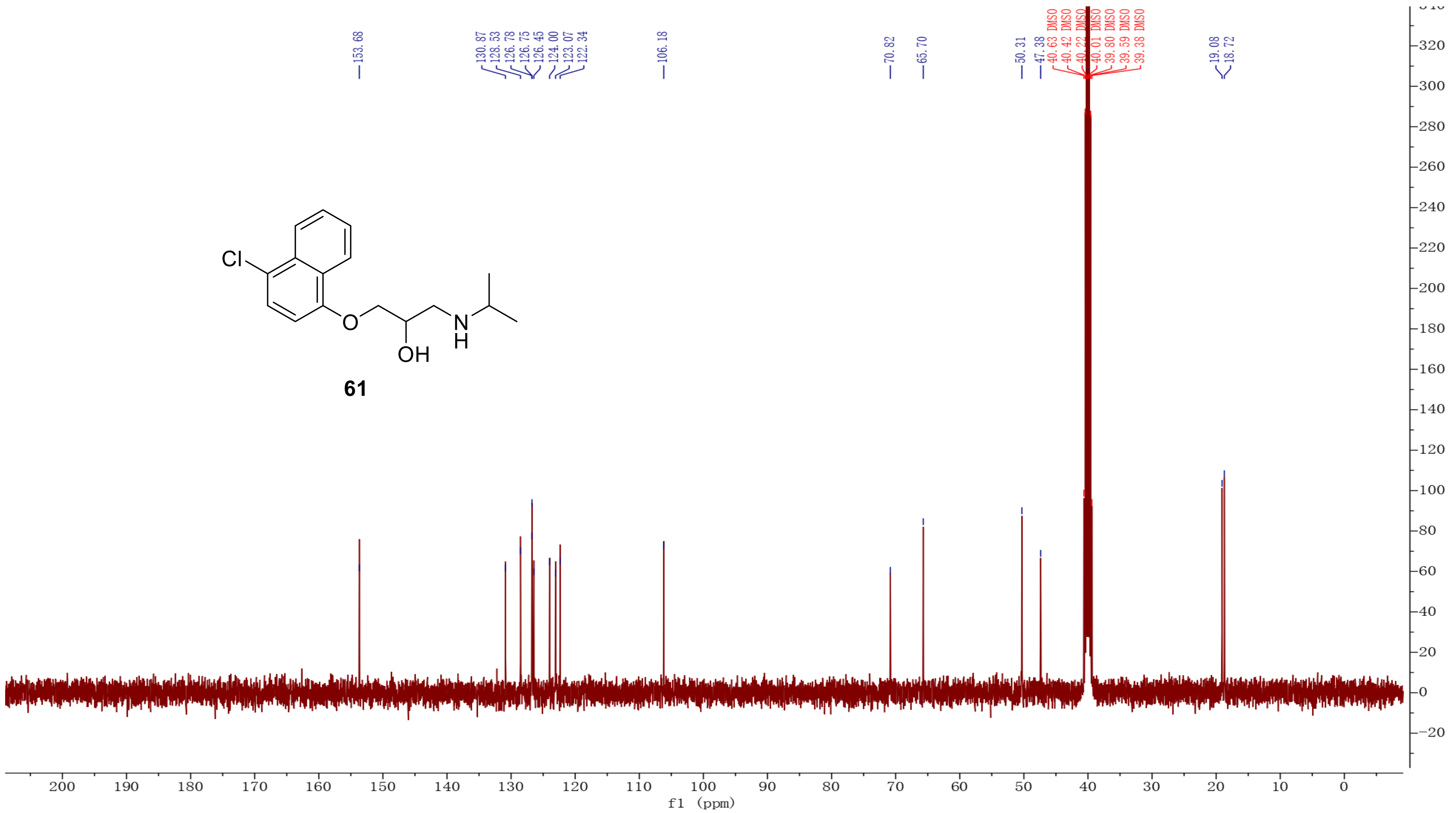

Supplementary Figure 146. ^{13}C NMR spectra of compound **61**.

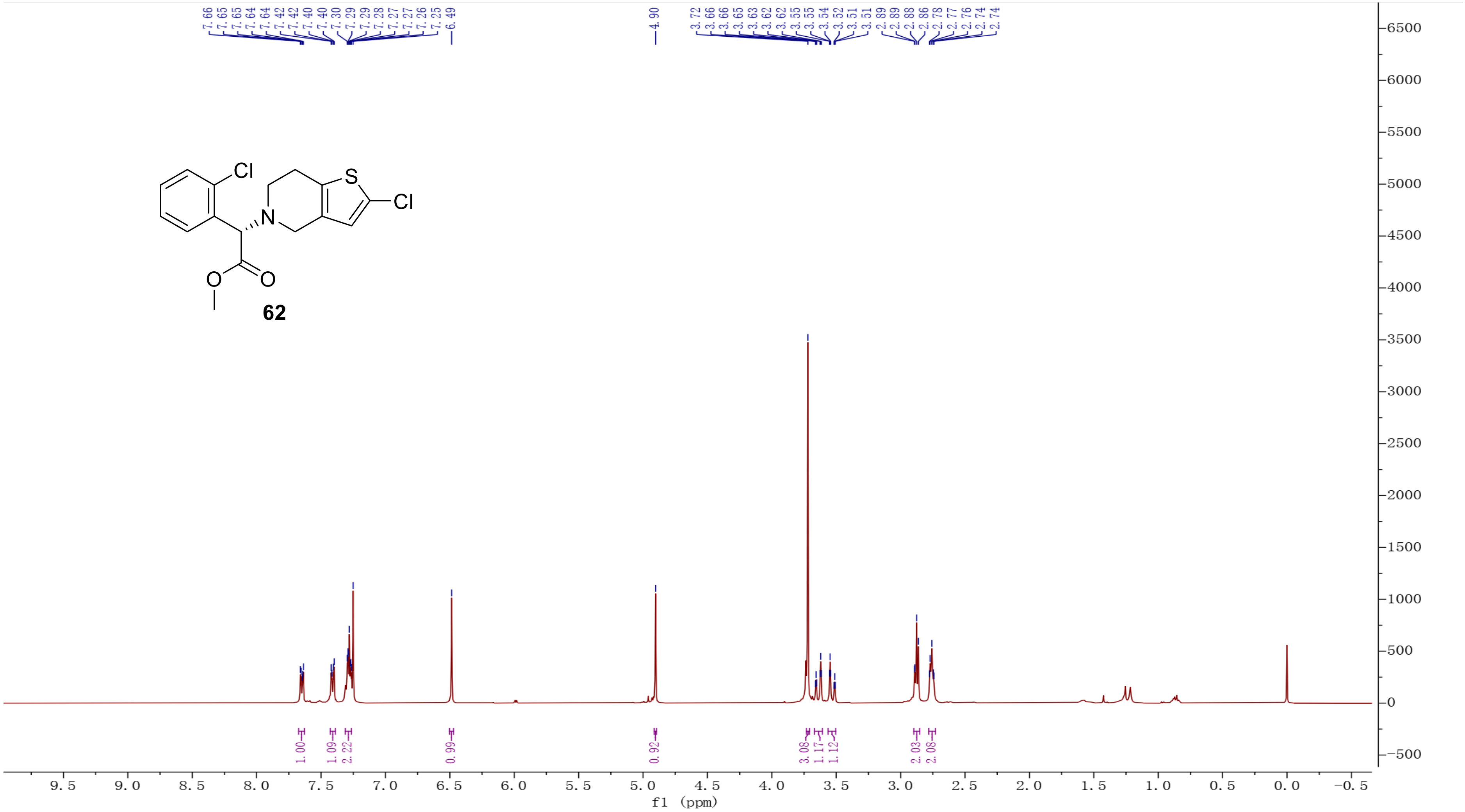

Supplementary Figure 147. ^{1}H NMR spectra of compound **62**.

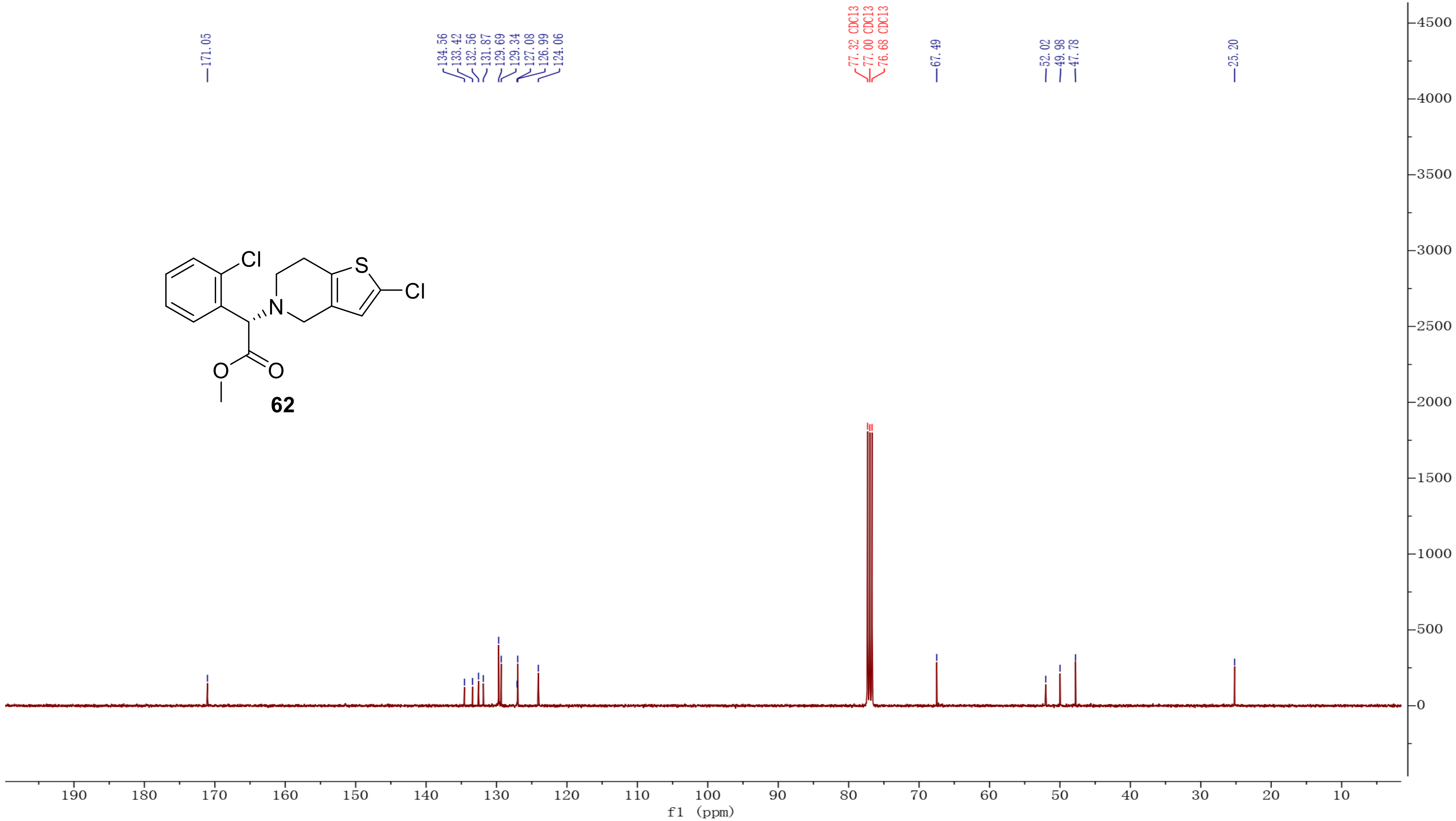

Supplementary Figure 148. ^{13}C NMR spectra of compound **62**.

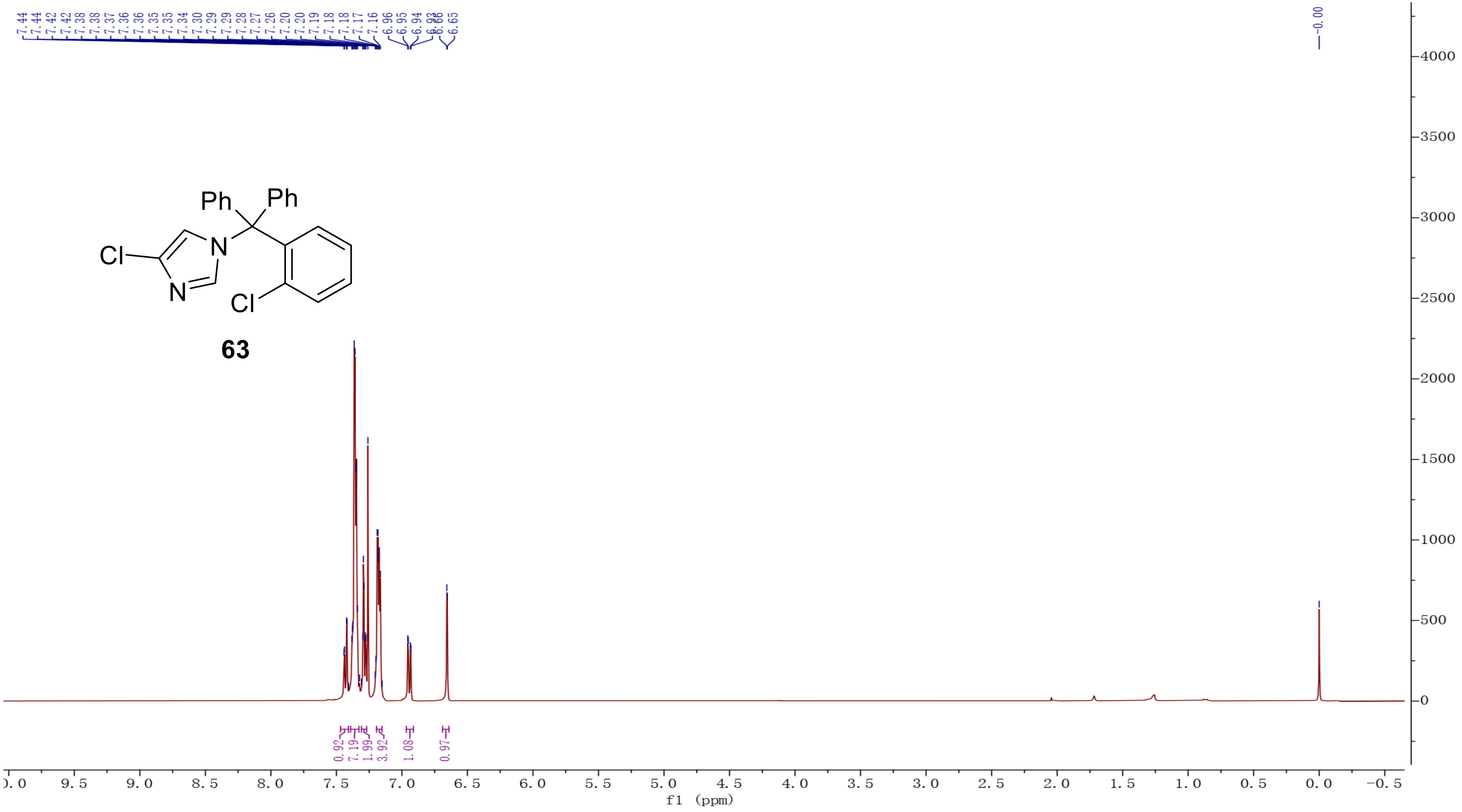

Supplementary Figure 149. ^{1}H NMR spectra of compound **63**.

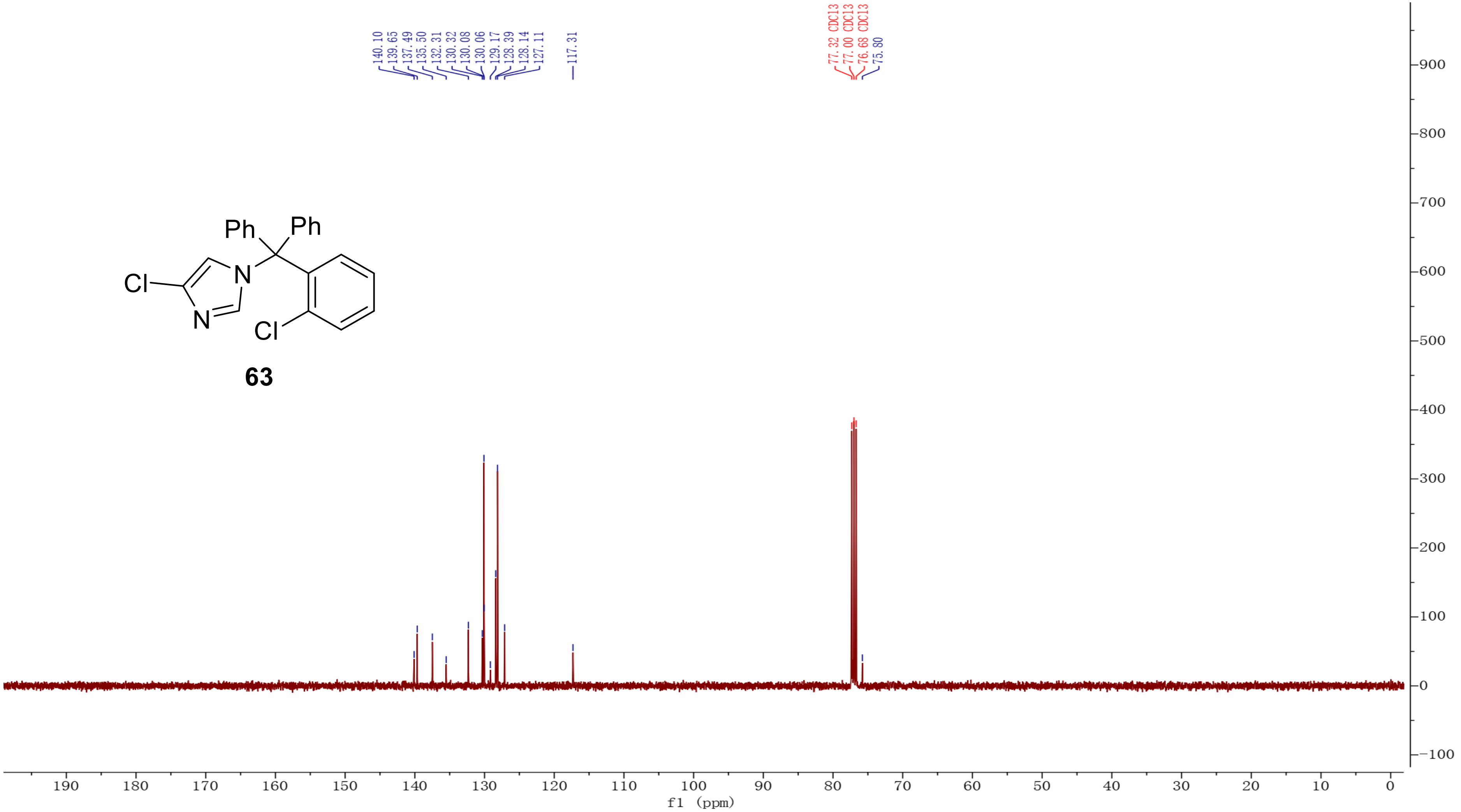

Supplementary Figure 150. ^{13}C NMR spectra of compound **63**.

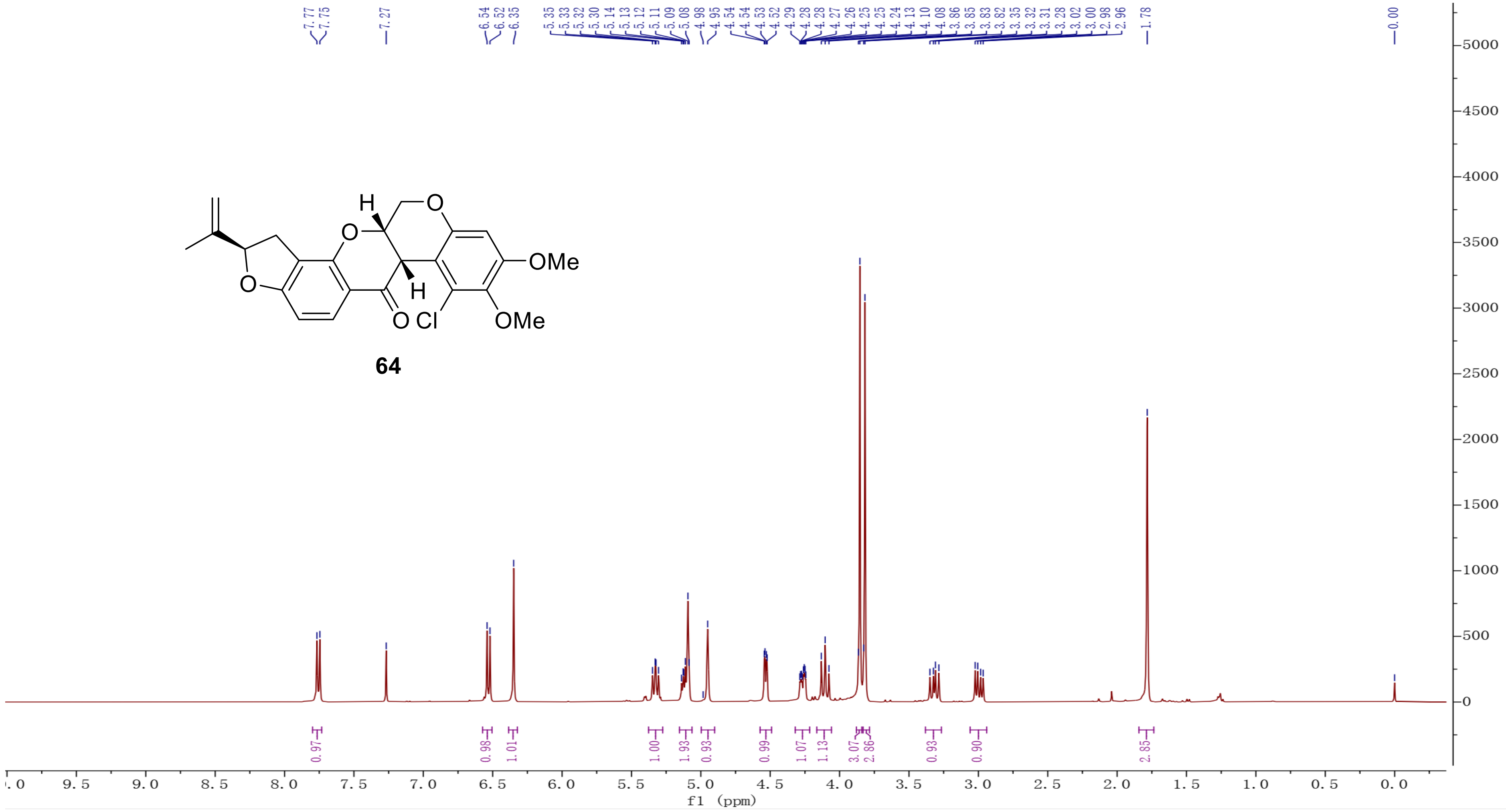

Supplementary Figure 151. ^{1}H NMR spectra of compound **64**.

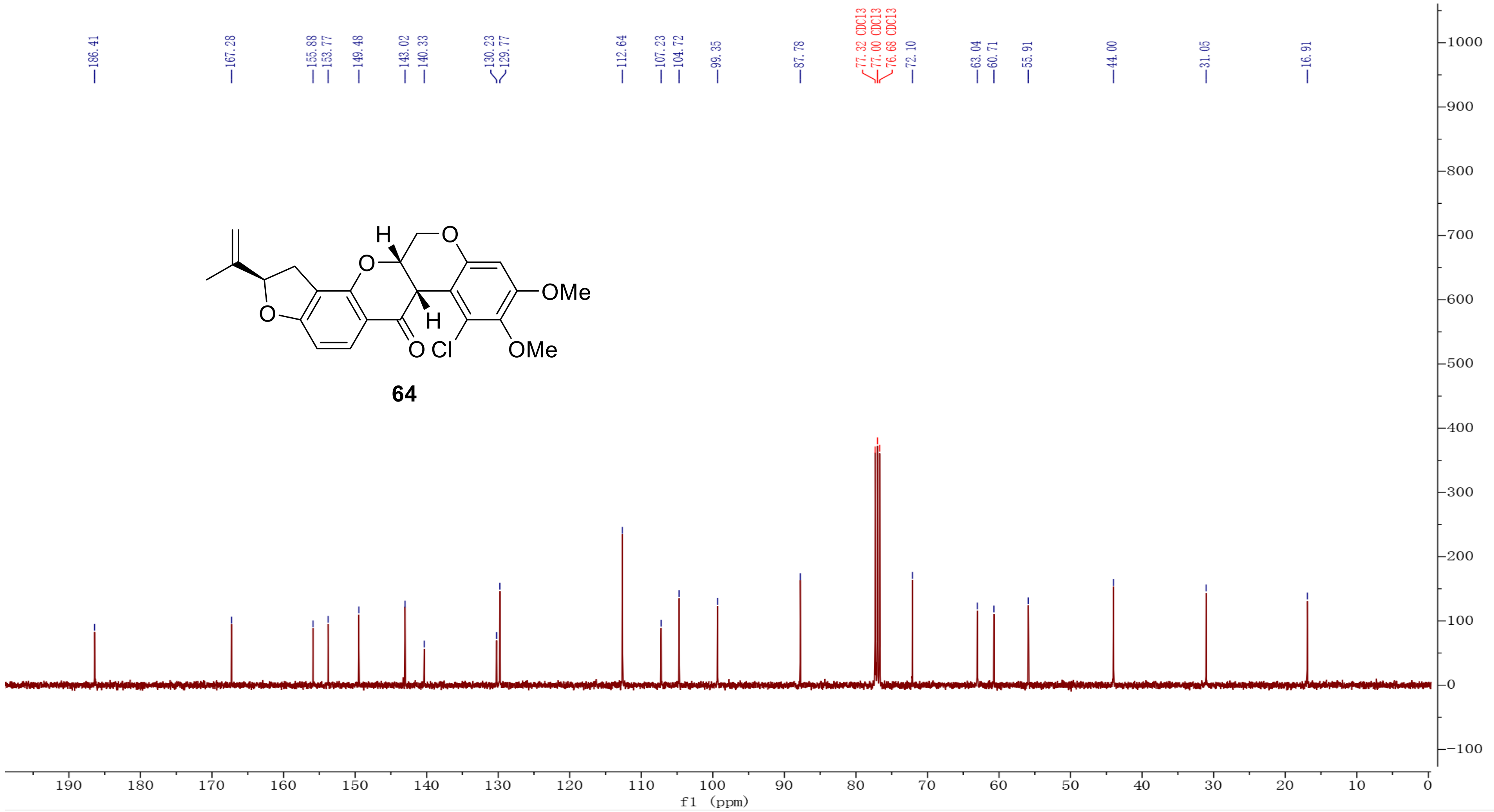

Supplementary Figure 152. ^{13}C NMR spectra of compound **64**.

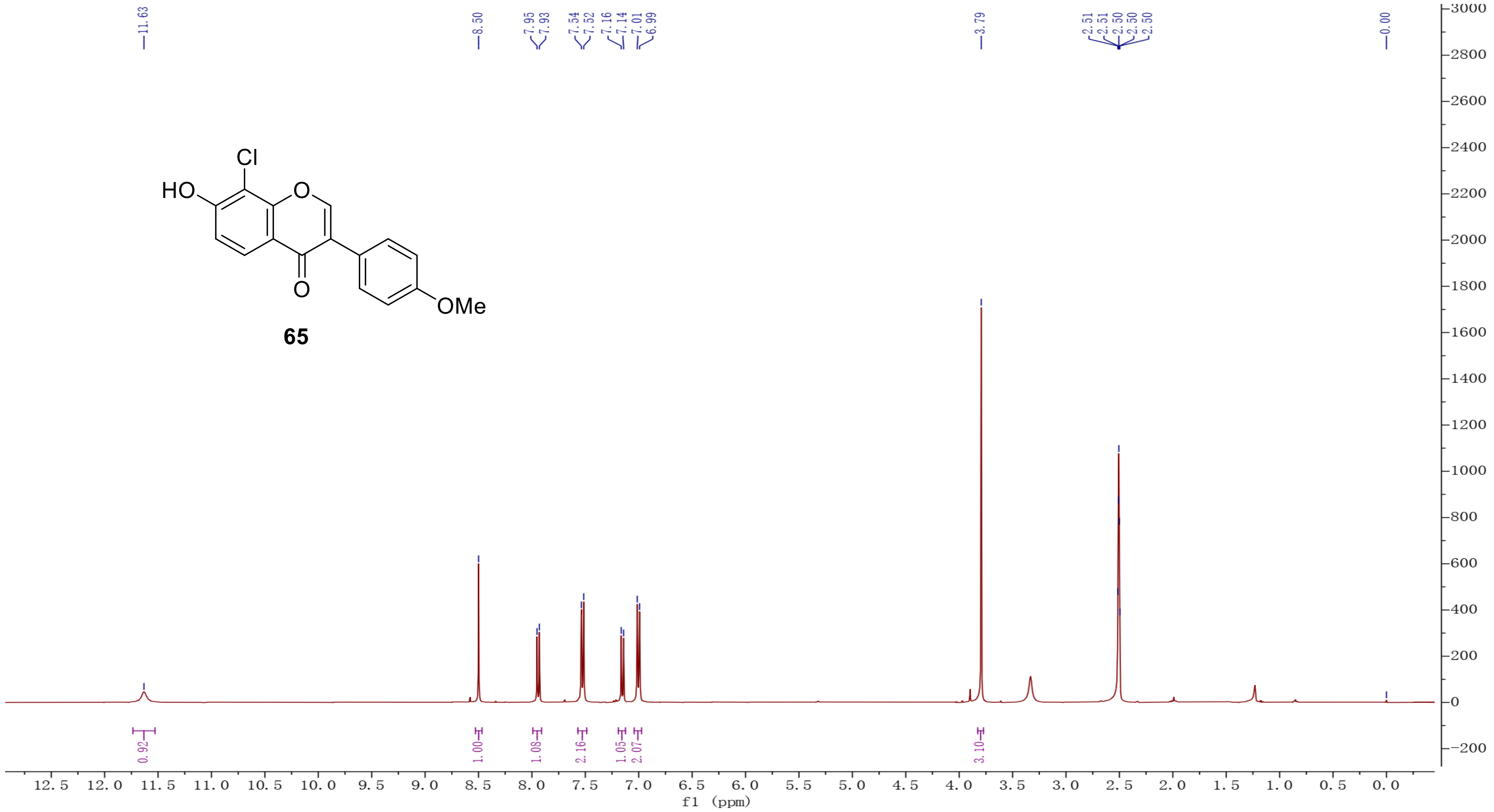

Supplementary Figure 153. ^{1}H NMR spectra of compound **65**.

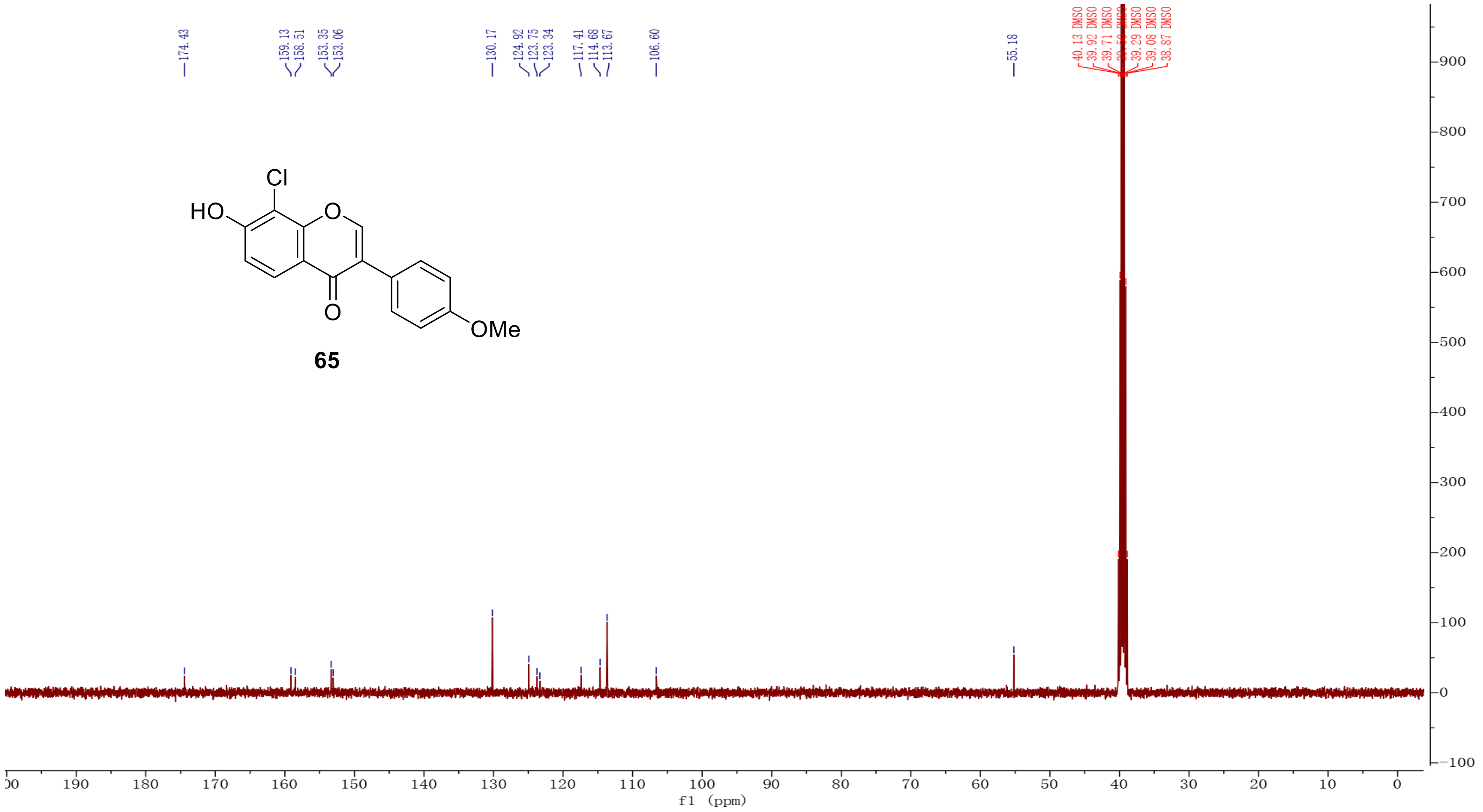

Supplementary Figure 154. ^{13}C NMR spectra of compound **65**.

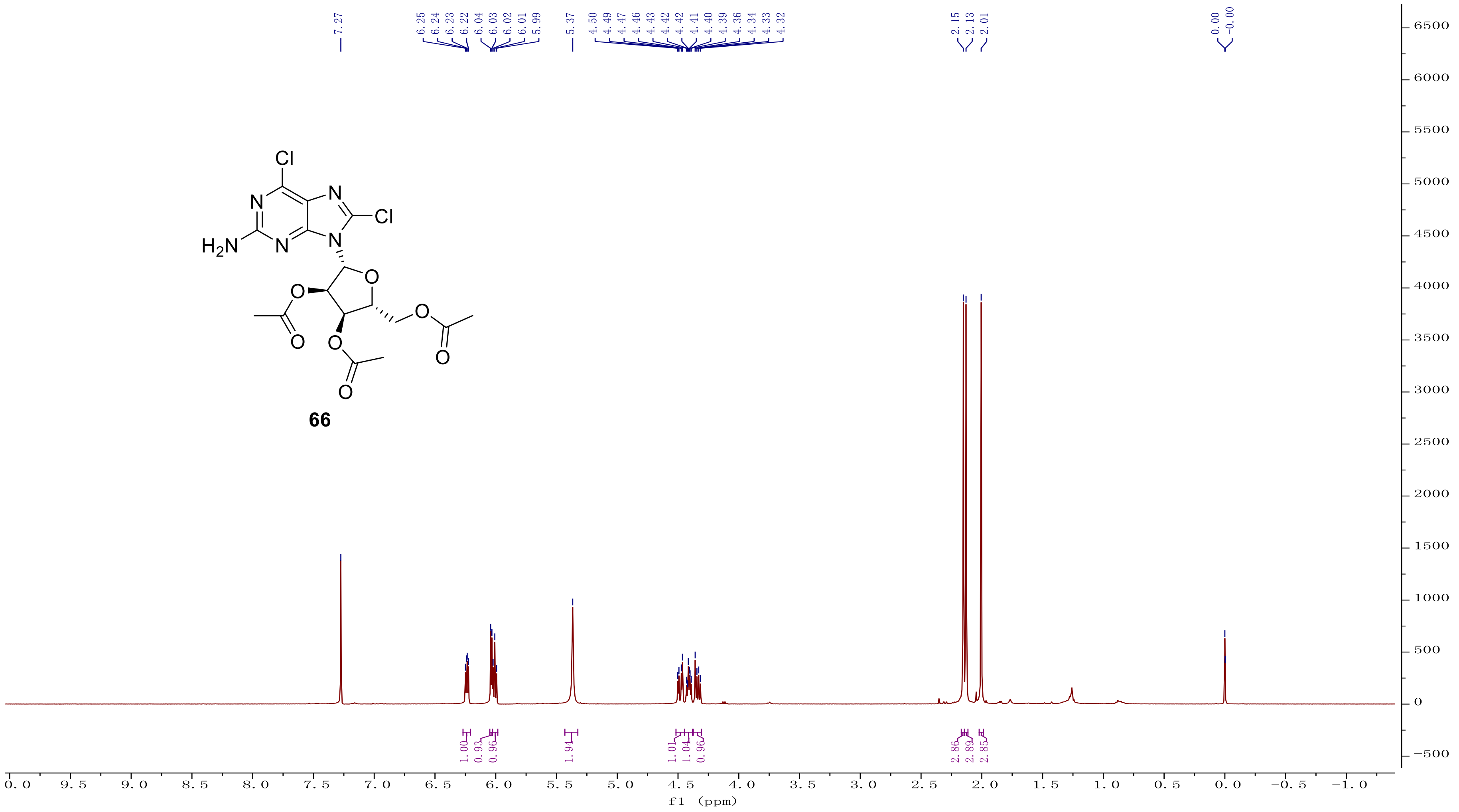

Supplementary Figure 155. ^{1}H NMR spectra of compound **66**.

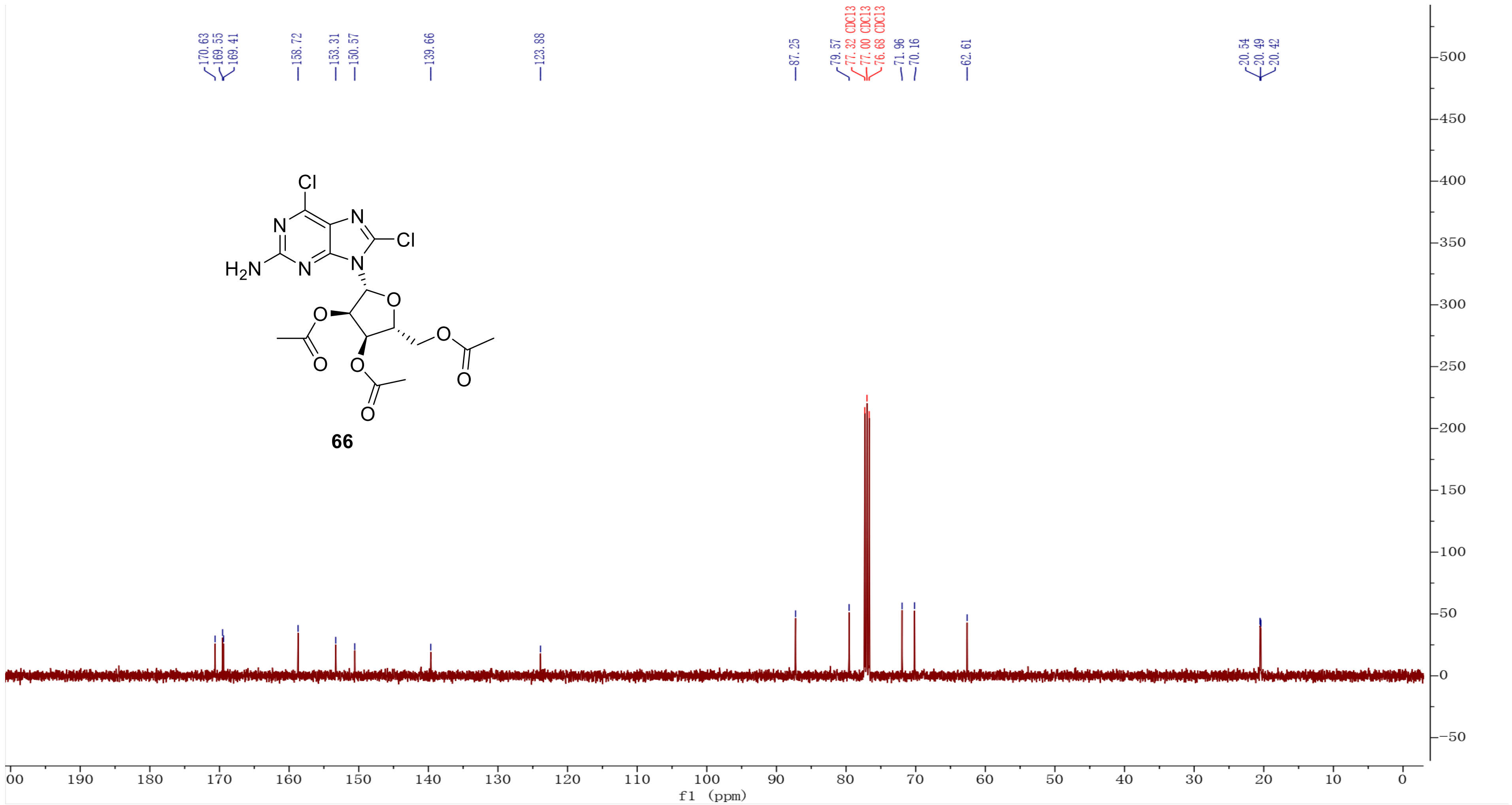

Supplementary Figure 156. ^{13}C NMR spectra of compound **66**.

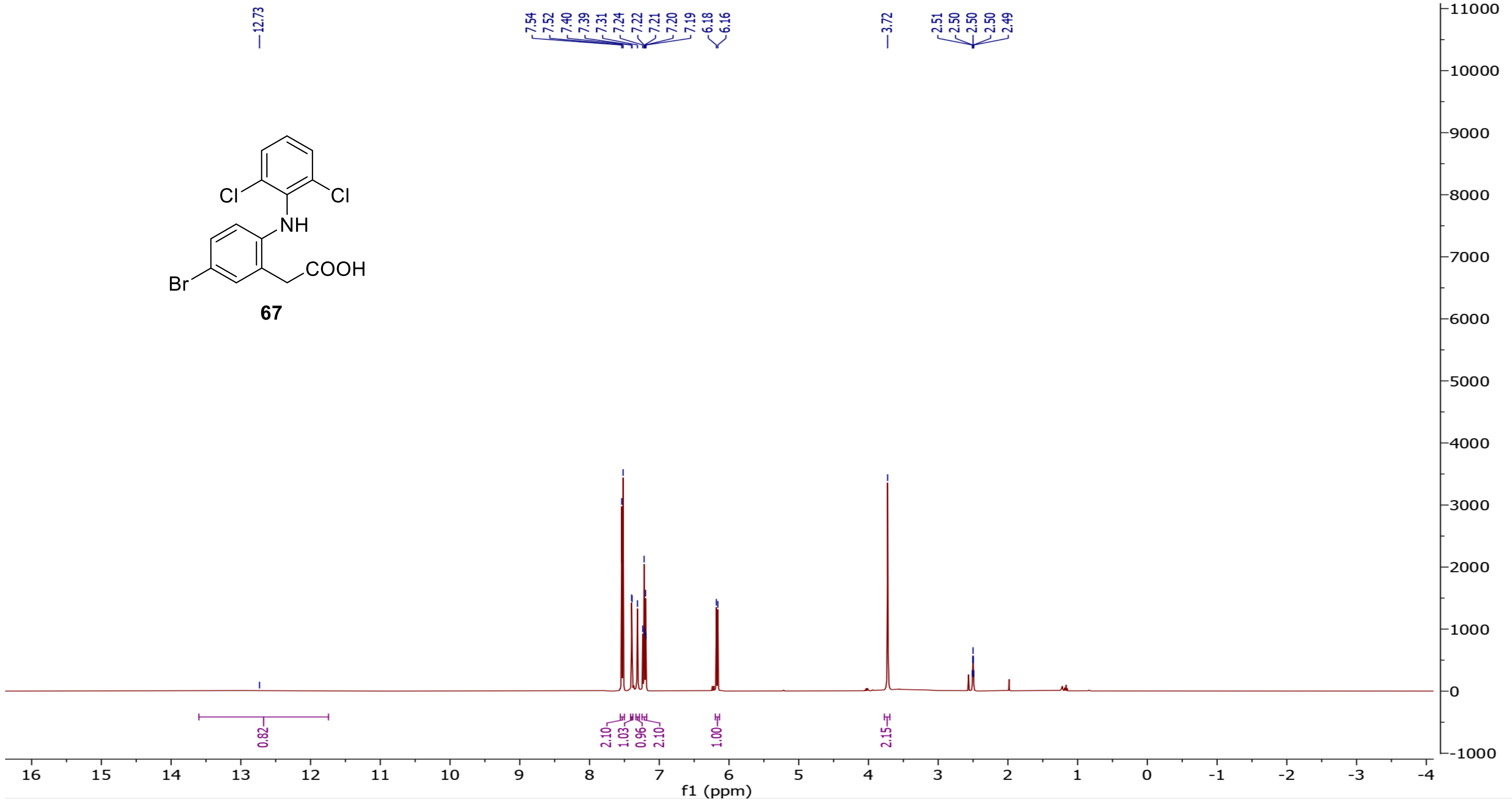

Supplementary Figure 157. ^{1}H NMR spectra of compound **67**.

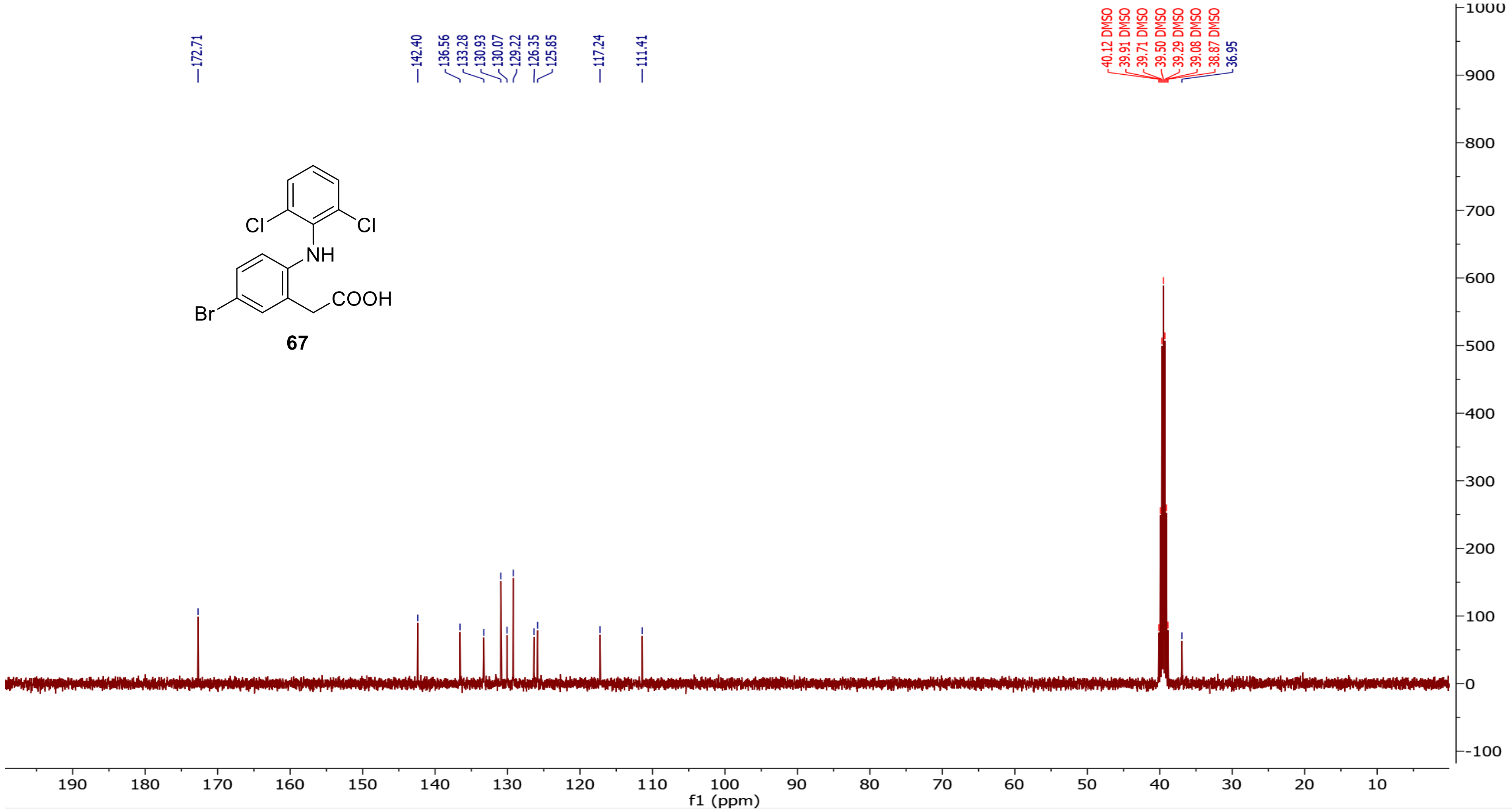

Supplementary Figure 158. ^{13}C NMR spectra of compound **67**.

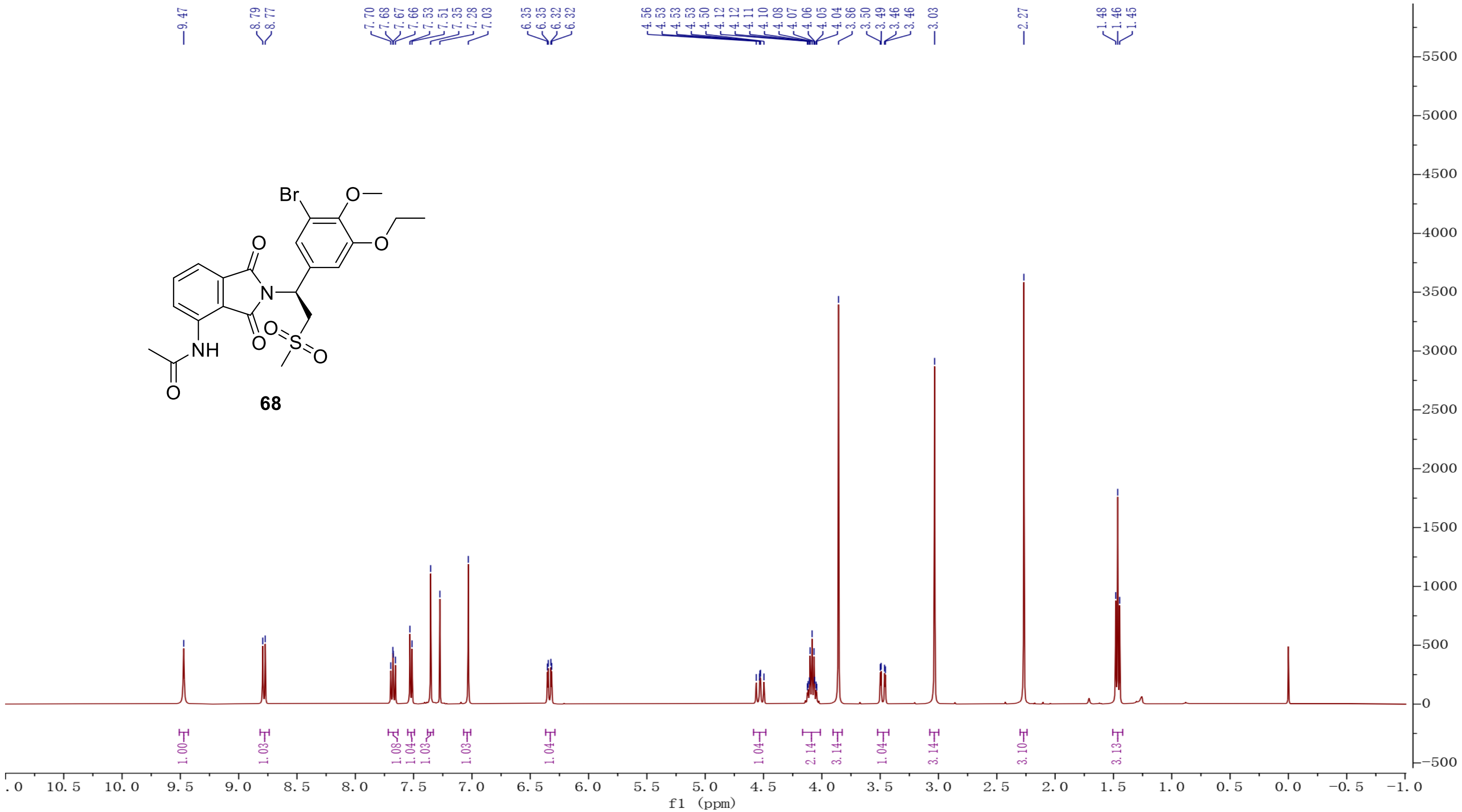

Supplementary Figure 159. ^{1}H NMR spectra of compound **68**.

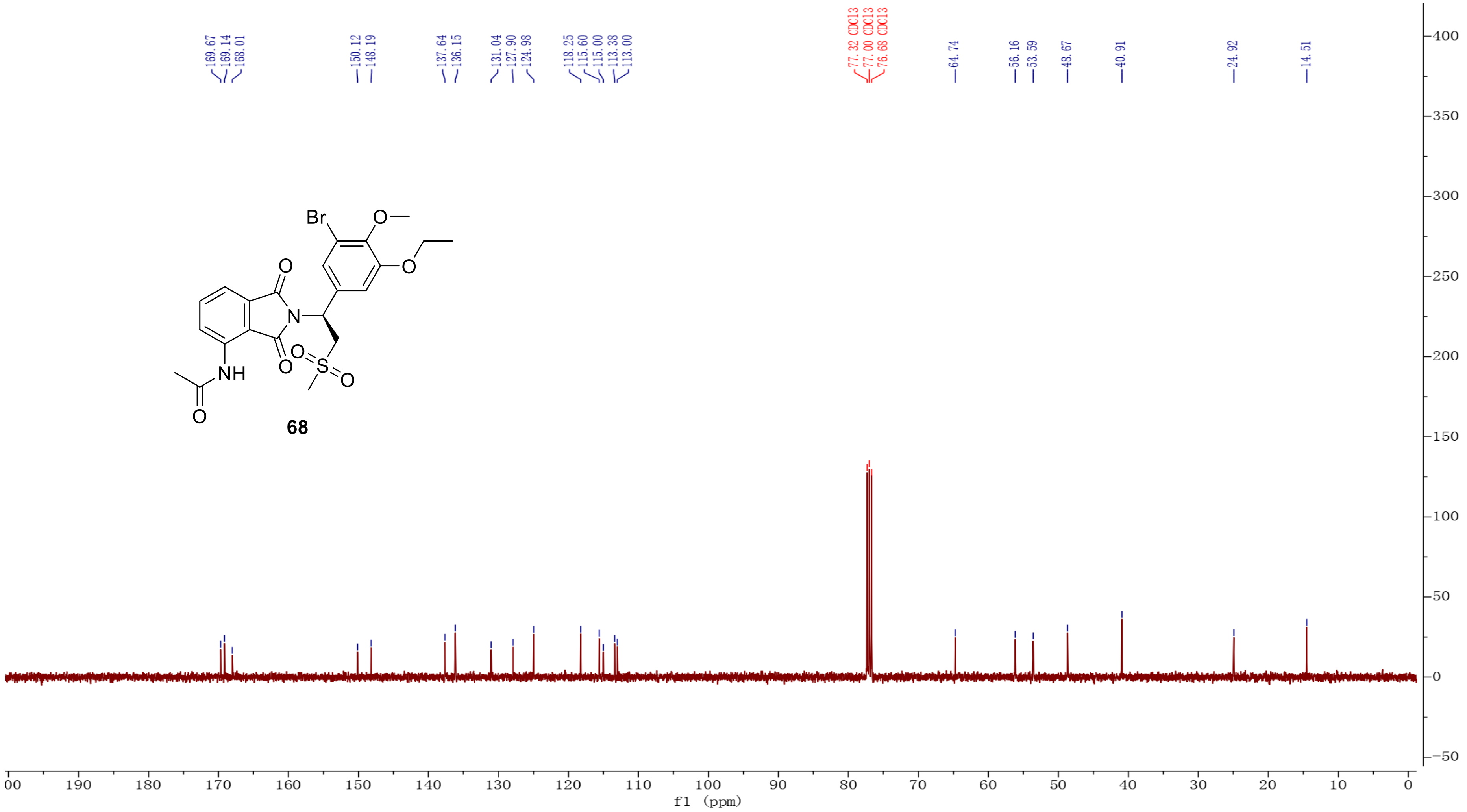

Supplementary Figure 160. ^{13}C NMR spectra of compound **68**.

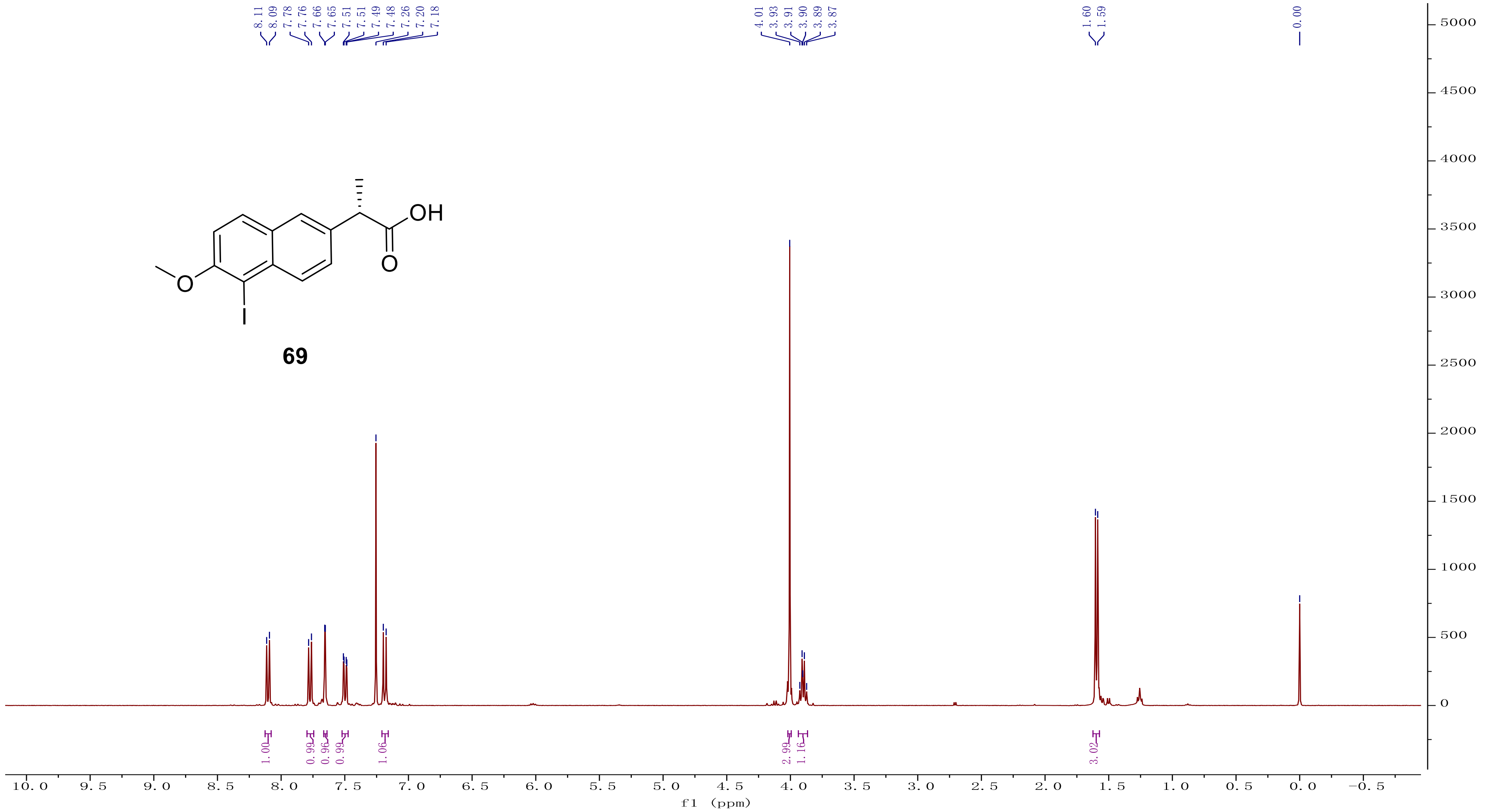

Supplementary Figure 161. ^{1}H NMR spectra of compound **69**.

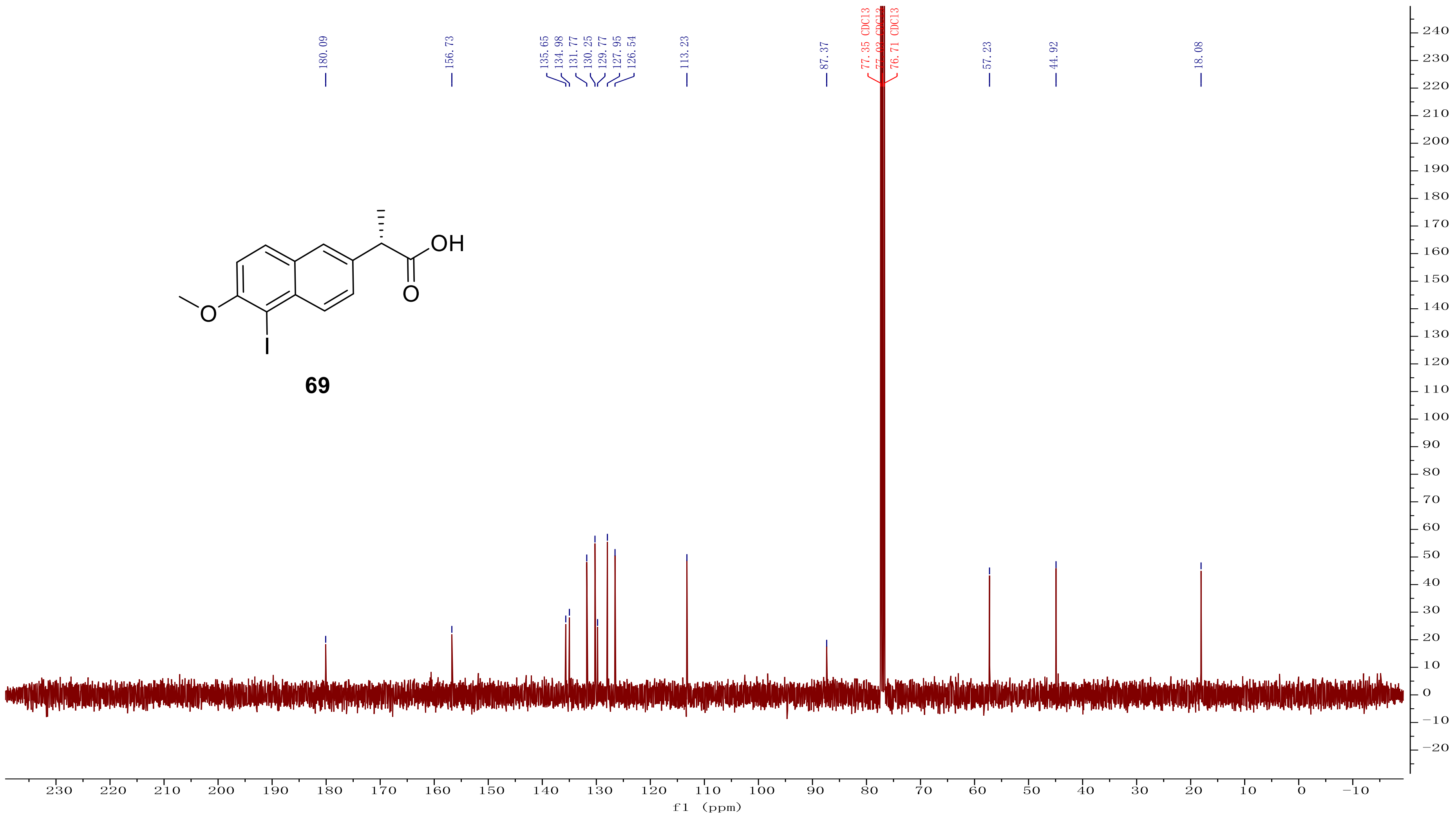

Supplementary Figure 162. ^{13}C NMR spectra of compound **69**.
